# Copper-Catalyzed Decarboxylative Elimination of Carboxylic Acids to Styrenes

**DOI:** 10.1021/acs.joc.2c02705

**Published:** 2023-01-20

**Authors:** Michael P. Stanton, Jessica M. Hoover

**Affiliations:** C. Eugene Bennett Department of Chemistry, West Virginia University, Morgantown, West Virginia 26506, United States

## Abstract

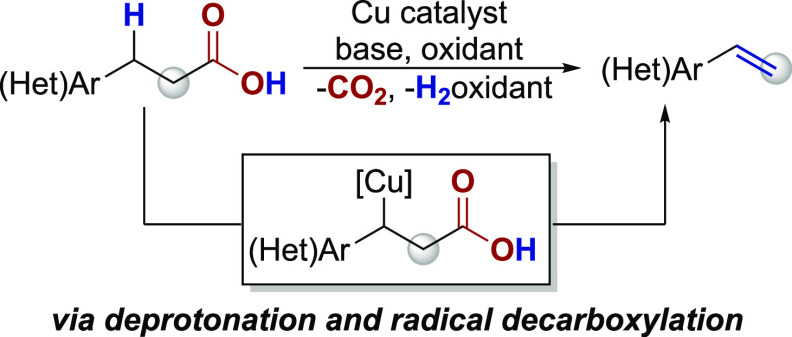

A copper-catalyzed decarboxylative elimination reaction of (hetero)aromatic propionic acids to vinyl (hetero)arenes has been developed. This method furnishes alkenes from carboxylic acids without the need for stochiometric Pb or Ag additives or expensive or specialized photocatalysts. A series of mechanistic experiments indicate that the reaction proceeds via benzylic deprotonation and subsequent radical decarboxylation; a pathway that is distinct from the single-electron-transfer mechanisms implicated in related decarboxylative elimination reactions.

## Introduction

In recent decades, the rising financial, environmental, and societal costs of petroleum and related fossil fuels have highlighted the importance of utilizing renewable feedstocks to access fuels and value-added commodity chemicals.^1^ Biomass-derived materials have emerged as ideal candidates for renewable chemical feedstocks because they are abundant and easy to handle.^2^ These sources, however, are highly oxygenated and the commercial utilization of biobased raw materials requires efficient deoxygenation reactions to access intermediates that are compatible with the existing petrochemical infrastructure.

**Scheme 1:**
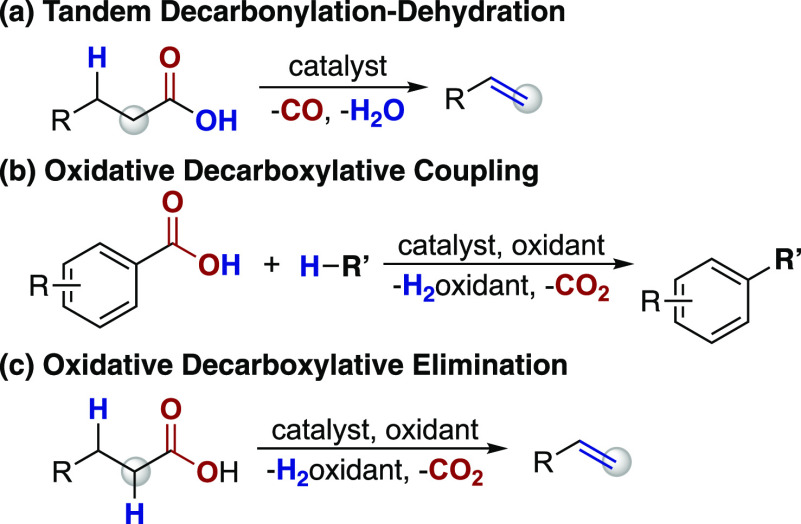
Transition-Metal-Catalyzed Deoxygenation Strategies

Of particular interest are terminal alkenes which are key additives in the manufacturing of plastics and detergents,^3^ and serve as valuable intermediates in the synthesis of fine chemicals and pharmaceuticals. With the appropriate deoxygenation technologies, these important targets could be accessed from bioderived fatty acids.^2^ One approach involves the tandem decarbonylation–dehydration of carboxylic acids (Scheme 1a). These methods, however, typically rely on the inclusion of stoichiometric anhydride activators and often generate isomeric mixtures of alkene products.^4^

Alternatively, decarboxylation reactions represent a deoxygenation strategy with the potential to provide access to a wide variety of value-added products. For example, protodecarboxylation reactions facilitate the conversion of aliphatic^5^ and aromatic^6^ carboxylic acids to the corresponding alkanes and arenes, respectively. Decarboxylative coupling reactions provide deoxygenative alternatives to traditional cross-coupling reactions^7^ for the formation of new C–N,^8^ C–O,^9^ C–S,^10^ C–X,^11^ and C–C bonds from both aliphatic and aromatic carboxylic acids (Scheme 1b).^12^ Decarboxylative elimination reactions of aliphatic carboxylic acids to generate alkenes, however, remain underdeveloped (Scheme 1c).^13^

**Scheme 2:**
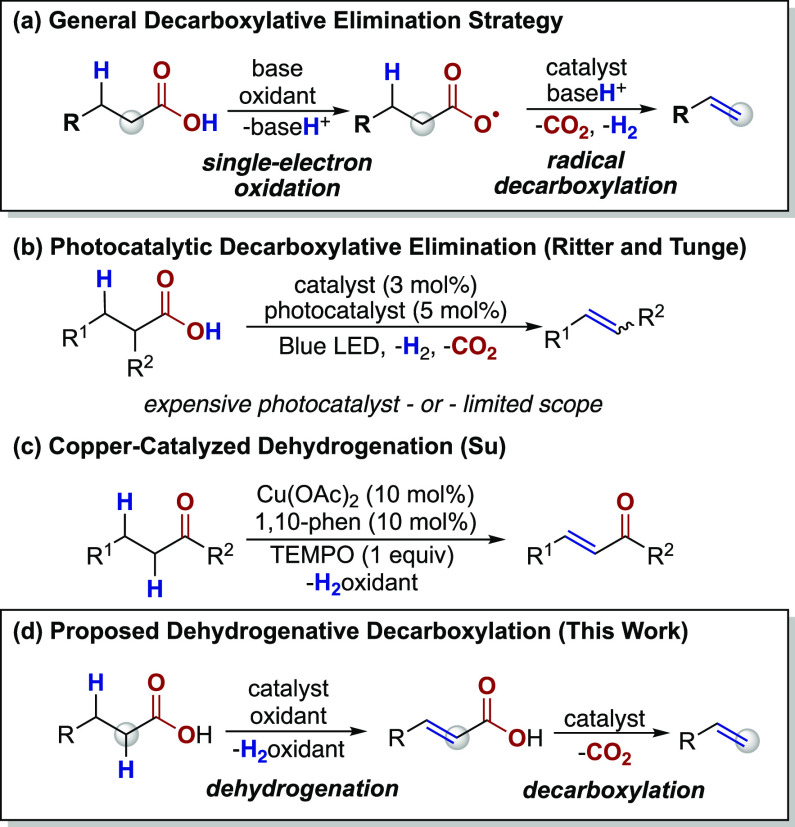
Transition-Metal-Catalyzed Decarboxylation Reactions

Early methods for the decarboxylative elimination of carboxylic acids to alkenes, pioneered by Kochi^14^ in the 1960s, relied on stochiometric amounts of toxic Pb(OAc)_4_^15^ or the use of persulfate salts^5^ as oxidants. Although these procedures represent the first example of this reaction pathway (Scheme 2a), widespread development of these protocols has been hindered by the harsh conditions required. Reactions employing catalytic loadings of Pb or Ag in conjunction with a Cu co-catalyst also facilitate the conversion of carboxylic acids to alkenes, albeit in modest yields and for limited acids.^16^

More recently, photoredox catalysis has emerged as an alternative approach for the decarboxylative elimination of aliphatic carboxylic acids (Scheme 2b).^17^ Ritter and coworkers have developed a dual catalytic approach involving an Ir photocatalyst paired with cobaloxime as a proton-reduction catalyst to enable the decarboxylation of fatty acids to α-olefins without the need for stochiometric additives.^18^ While this protocol provides efficient access to a large scope of alkene products, the cost of the photocatalyst may limit larger-scale applications.

A related strategy has been reported by Tunge and coworkers who used an acridinium organo-photocatalyst and cobaloxime proton-reduction catalyst for the decarboxylative elimination of carboxylic acids to enamides and enecarbamates (Scheme 2b, *R*^2^ = NHAc or NHBoc).^19^ Shortly thereafter, Larionov and coworkers described a similar dual catalyst system composed of acridine and cobaloxime for the efficient decarboxylative elimination of biomass-derived carboxylic acids.^20^ Therefore, to date, all existing methods for the decarboxylative elimination reaction rely on a single-electron-transfer pathway to generate an acyloxy intermediate which can extrude CO_2_, followed by subsequent elimination of the resulting alkyl radical to furnish the alkene product (Scheme 2a).^5,21^ Given the prevalence of copper-catalyzed decarboxylation reactions, we hypothesized that a copper-based dehydrogenation-decarboxylation strategy might eliminate the need for expensive photocatalysts or stoichiometric Pb, while also providing selectivity for the terminal olefin products (Scheme 2d).

To evaluate the feasibility of such a strategy, we envisioned the decarboxylative elimination of hydrocinnamic acids to styrenes as a proof-of-concept reaction. Copper catalysts have established efficiency for the decarboxylation of cinnamic acids, in both protodecarboxylation and decarboxylative coupling reactions.^22,23^ Additionally, Su and coworkers have demonstrated that a Cu(OAc)_2_/TEMPO system facilitates the α,β-dehydrogenation of carbonyl-containing compounds such as aldehydes, ketones, and diesters (Scheme 2c, TEMPO = 2,2,6,6-tetramethylpiperidine-1-oxyl).^24^ The precedent for copper-catalyzed dehydrogenation as well as decarboxylation reactions makes it a good candidate for a new manifold of decarboxylative elimination reactions.

## Results and Discussion

In an effort to identify a Cu catalyst system for the efficient decarboxylative elimination of hydrocinnamic acids to styrenes, we focused our initial study on *para*-nitrohydrocinnamic acid. Employing a Cu(OAc)_2_ catalyst with 2,2′-bipyridine (bpy) as a ligand paired with TEMPO as the oxidant generated 4-nitrostyrene (**2a**) in 13% yield with the dehydrogenation product, 4-nitrocinnamic acid (**3a**), formed in 72% yield (Table 1, entry 1). Substituting Cu(OAc)_2_ with CuOAc resulted in a similar yield of **2a** at 12%; however, a marked increase in 4-nitrocinnamic acid to 81% was observed (Table 1, entry 2). We found the identity of the oxidant to have a dramatic influence on the yield of the decarboxylative elimination product. For example, utilizing di-*tert*-butylperoxide as the oxidant, **2a** was observed in 45% yield (Table 1, entry 5), while MnO_2_ was found to dramatically increase the yield of **2a** to 82% (Table 1, entry 6). Increasing the temperature from 110 °C to 120 °C resulted in a slight decrease in the yield of **2a** to 79%; however, it also afforded improved mass balance and reproducibility for the reaction. Therefore, the optimized catalytic reaction conditions employ 20 mol % CuOAc and bpy, MnO_2_ (2.0 equiv), and LiOAc (2.0 equiv) in DMA at 120 °C for 24 h, to generate the decarboxylative elimination product in 79% yield (Table 1, entry 7). Furthermore, when stoichiometric loadings of CuOAc/bpy are employed in the absence of an additional oxidant (Table 1, entry 8), **2a** is obtained in similar yields to the catalytic conditions, highlighting the importance of Cu to the overall transformation.

**Table 1:**
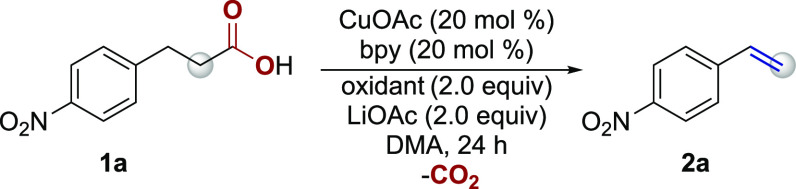
Optimization of Reaction Conditions^a^

entry	[Cu]	oxidant	temp (°C)	yield (%)
1	Cu(OAc)_2_	TEMPO	110	13^b^
2	CuOAc	TEMPO	110	12^c^
3	CuOAc	Na_2_S_2_O_8_	110	0
4	CuOAc	NFSI	110	0
5	CuOAc	*t*BuOO*t*Bu	110	45
6	CuOAc	MnO_2_	110	82
**7**	**CuOAc**	**MnO_2_**	**120**	**79 (62)**^d^
8^e^	CuOAc		120	82
9^f^		MnO_2_	120	<5%

a Reaction conditions: **1a** (0.1 mmol) in DMA (1 mL) under N_2_. Yields obtained from ^1^H NMR spectroscopy with 1,3,5-trimethoxybenzene as the internal standard.

b 72% yield **3a**.

c 81% yield **3a**.

d Isolated yield.

e CuOAc and bpy (1.2 equiv).

f No CuOAc or bpy.

**Scheme 3:**
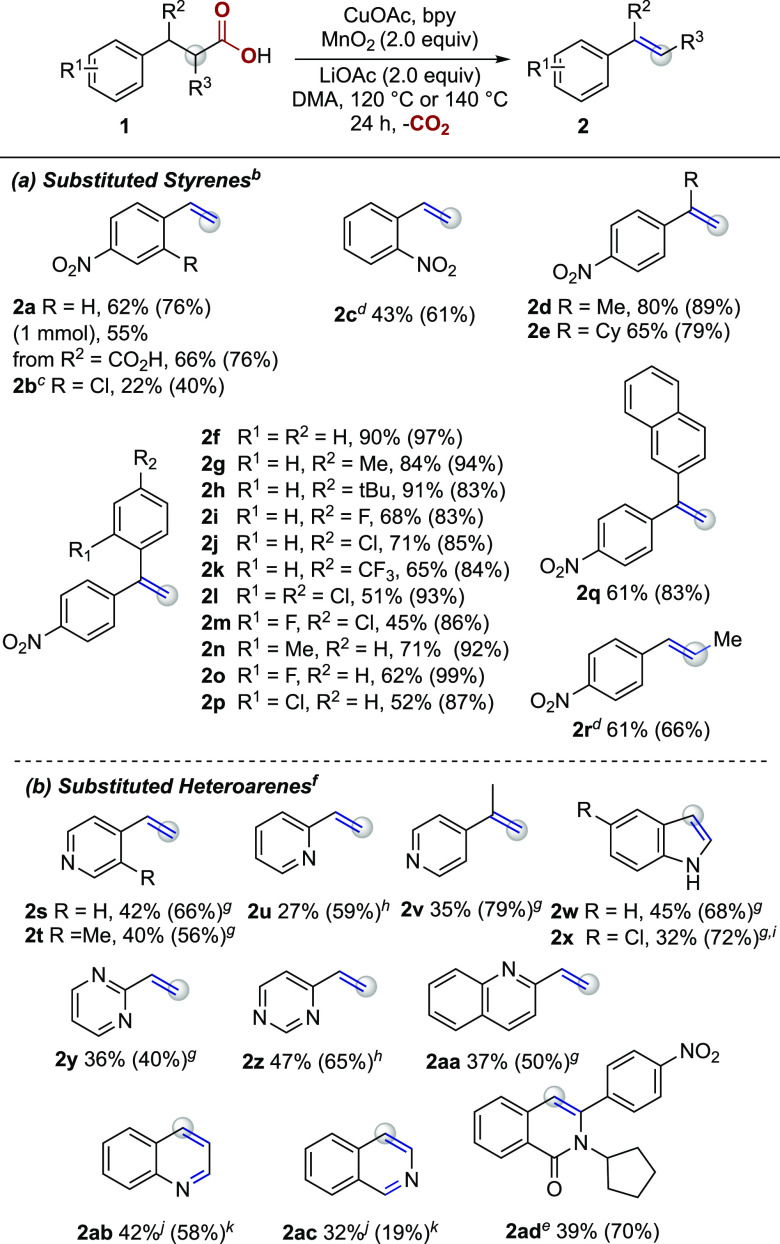
Scope of Substituted Aryl Propionic Acids Isolated yields with ^1^H NMR yields listed in parentheses. Reaction conditions: **1** (0.3 mmol), CuOAc and bpy (20 mol %), MnO_2_ (2.0 equiv), LiOAc (2.0 equiv) in DMA (3 mL) at 120 °C. 90 °C. CuOAc and bpy (1.2 equiv) at 110 °C. 140 °C. Reaction conditions: **1** (0.4 mmol), CuOAc (1.2 equiv), LiOAc (2.0 equiv) in DMA (4 mL) at 140 °C. With bpy (1.2 equiv). With bpy (1.2 equiv) at 120 °C. **1x** was used as the hydrochloride hydrate, LiOAc (3.0 equiv). Reaction conditions: **1** (0.4 mmol), CuOAc (20 mol %), MnO_2_ (4.0 equiv), LiOAc (2.0 equiv) in DMA (4 mL) at 140 °C. With bpy (20 mol %).

After optimizing the reaction conditions, we turned our attention to establishing the scope and limitations of this transformation (Scheme 3). Substitution in the *ortho*-position of the phenyl ring was tolerated with the disubstituted arene **2b** furnishing the corresponding product in moderate yield. Phenylpropionic acids bearing alkyl (**2d**, **2e**) or aryl groups (**2f–2p**) in the benzylic position formed products in good yields. Both electron-donating groups (**2g, 2h, 2n**) and electron-withdrawing groups (**2i–m, 2o, 2p**) were tolerated in the *ortho*- and *para*-positions of the second aromatic ring of 3,3-diphenylpropionic acids. Naphthyl substitution at the benzylic position of 4-nitrohydrocinnamic acid also furnished the corresponding alkene **2q** in 61% yield. Interestingly, 2-(4-nitrophenyl)succinic acid also underwent oxidative decarboxylation to generate **2a** in good yield (66%). Dehydrogenative decarboxylation of 2-methyl-(3-nitrophenyl)propionic acid successfully yielded the internal *trans*-alkene product (**2r**) selectively, while the related photoredox catalytic methods would be expected to generate a mixture of regioisomeric products from this and related substrates.^18^ In all of these cases, however, a nitro substituent is required in either the *ortho*- or *para*-position (Scheme S1), suggesting the possible importance of the resonance-withdrawing groups.

Owing to the prominence of nitrogen-containing heteroarenes in pharmaceutically relevant compounds,^25^ convenient access to vinyl heteroarenes in a single step is a desirable transformation.^26^ Thus, we explored carboxylic acids bearing heterocycles which share the resonance withdrawing feature. Gratifyingly, a number of heteroarene-containing carboxylic acids underwent efficient decarboxylative elimination under conditions with stoichiometric CuOAc/bpy (Scheme 3b). For example, both *ortho*- and *para*-substituted pyridine propionic acids were converted to the corresponding vinyl products in 27% **(2u)** and 42% (**2s**) yields, respectively. Indole products **2w** and **2x** were generated in moderate yields (45 and 32%) from indoline-3-propionic acids. A quinoline-substituted propionic acid was also amenable to the reaction conditions, forming the vinyl product (**2aa**) in 37% yield. Additionally, it was found that 1,2,3,4-tetrahydro-3-quinolinecarboxylic acid (**1ab**) and 1,2,3,4-tetrahydro-3-isoquinolinecarboxylic acid (**1ac**) generated the corresponding quinoline products in moderate yields. Finally, the more structurally complex substrate **1ad** was successfully converted to the *N*-cyclopentyl isoquinolinone, **2ad**, in moderate yield. Given the structural similarities between bpy and the heterocyclic products, isolation of these alkenes on a small scale by silica column chromatography was challenging. To facilitate purification, the products could be generated in the absence of bpy, although in somewhat lower yields (Scheme 3b).

**Scheme 4:**
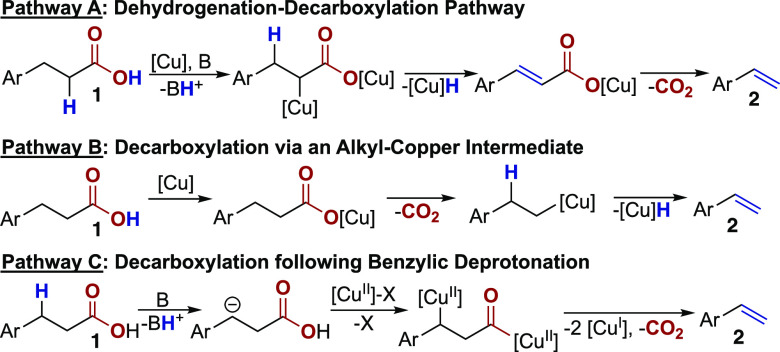
Possible Reaction Pathways for the Copper-Catalyzed Decarboxylative Elimination

We considered three reaction pathways that could account for alkene formation under these conditions (Scheme 4). The first, **Pathway A**, involves an initial dehydrogenation event followed by the decarboxylation of the resulting cinnamic acid derivative to provide the styrene product.^28^ In this pathway, dehydrogenation occurs via deprotonation alpha to the carbonyl.^24,27^ An alternative pathway, **Pathway B**, begins with the decarboxylation of **1** to generate an alkyl-copper species which could undergo elimination to yield the styrene product.^28^ Finally, in **Pathway C**, the initial deprotonation at the benzylic position of **1** would generate the benzylic anion. Recombination of this anion with copper would enable single-electron decarboxylation to furnish the product through a formal dehydrogenation process, without the formation of an α,β-unsaturated acid intermediate.^29^

**Scheme 5:**
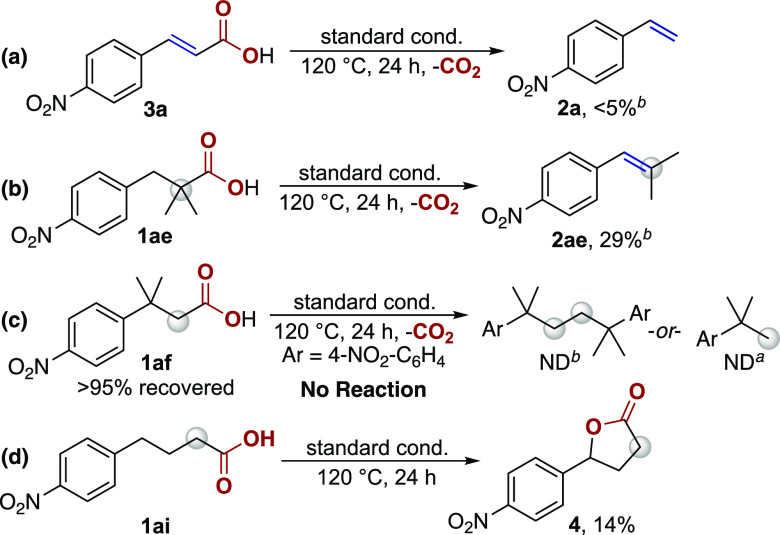
Control Reactions for the Copper-Catalyzed Decarboxylative Elimination Yields obtained from ^1^H NMR spectroscopy with 1,3,5-trimethoxybenzene as an internal standard. All reactions conducted in DMA (1 mL) under a N_2_ atmosphere at 120 °C. Reaction conditions: **1** or **3** (0.1 mmol), CuOAc and bpy (20 mol %), MnO_2_ (2.0 equiv), LiOAc, (2.0 equiv). Reaction conditions: **1ai** (0.1 mmol), CuOAc and bpy (1.2 equiv), LiOAc (2.0 equiv).

Pathway **A** was eliminated with a series of control experiments (Scheme 5). This pathway relies on the efficient decarboxylation of the proposed cinnamic acid intermediate.^29^ The decarboxylation of 4-nitrocinnamic acid (**3a**) under the reaction conditions, however, affords 4-nitrostyrene (**2a**) in <5% yield, with 74% starting material recovered, inconsistent with the intermediacy of 4-nitrocinnamic acid. Furthermore, when positions adjacent to the carbonyl are blocked, such as in 2,2-dimethyl-3-(4-nitrophenyl)propanoic acid (**1ae**), decarboxylative elimination still occurs to yield the corresponding styrene product (**2ae**) in 29% yield. These data indicate a pathway involving deprotonation adjacent to the carbonyl to be unlikely.

The possibility of **Pathway B** was evaluated with the reaction of 3-methyl-3-(4-nitrophenyl)butanoic acid (**1af**, Scheme 5c). In this case, the proposed alkyl-copper intermediate cannot undergo elimination, and instead, oxidative coupling or protodecarboxylation products would be expected, as demonstrated by Kochi and co-workers.^28^ No products derived from decarboxylation are observed and the starting material was recovered in quantitative yield, suggesting that **Pathway B** is inoperative under the reaction conditions. Instead, we favor **Pathway C**. This pathway is consistent with the formation of **2ae** from **1ae**. In addition, the reaction of 4-(4-nitrophenyl)butanoic acid (**1ai**) did not furnish the alkene product, and instead, 4-(4-nitrophenyl)-γ-butyrolactone (**4**, 14% yield) was formed (Scheme 5d).^30^ These data demonstrate the feasibility of a pathway initiated with activation at the benzylic position.

**Scheme 6:**
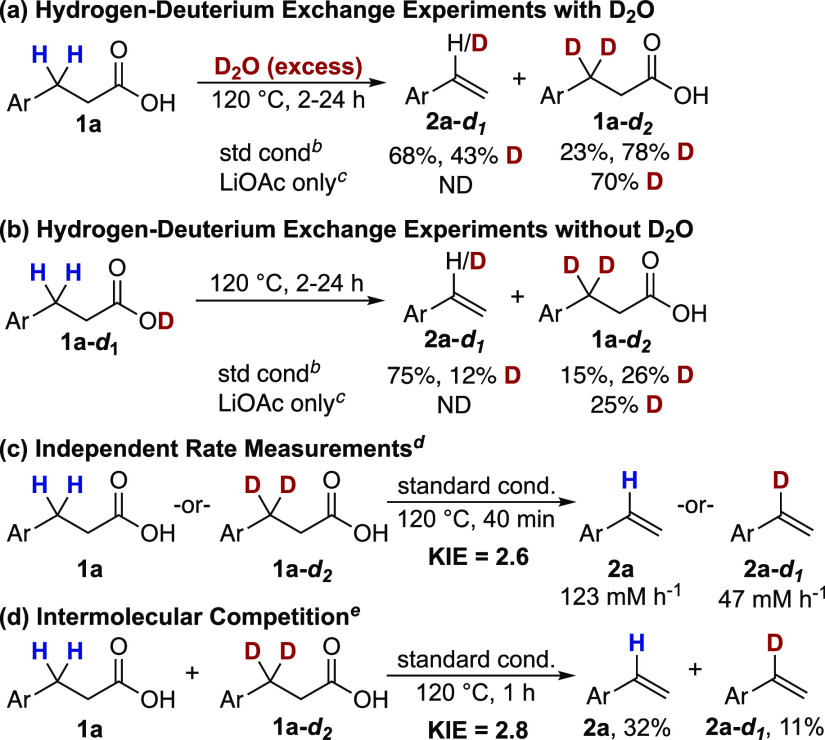
Hydrogen Deuterium Exchange and Kinetic Isotopic Effect Studies Yields obtained from ^1^H NMR spectroscopy with 1,3,5-trimethoxybenzene or methyl 3,5-dinitrobenzoate as an internal standard. Reaction conditions: **1a** (0.10 mmol) and D_2_O (80 equiv) or **1a-*d*_*1*_** (0.15 mmol, no D_2_O), CuOAc and bpy (20 mol %), MnO_2_ (2.0 equiv), LiOAc (2.0 equiv) in DMA (1.0 or 1.5 mL) for 24 h. Reaction conditions: **1a** (0.15 mmol) and D_2_O (80 equiv) or **1a-*d*_*1*_** (0.15 mmol, no D_2_O), LiOAc (2.0 equiv) in DMA (1.5 mL) for 2 h. Reaction conditions: **1a** and **1a-*d*_*2*_** (0.2 mmol), CuOAc and bpy (20 mol %), MnO_2_ (2.0 equiv), LiOAc (2.0 equiv) in DMA (2 mL). Reaction conditions: **1a** and **1a-*d*_*2*_** (0.05 mmol), CuOAc and bpy (0.01 mmol), MnO_2_ (0.1 mmol), LiOAc (0.1 mmol) in DMA (0.5 mL).

Activation of a benzylic C–H bond is also supported by H/D exchange and kinetic isotope effect experiments (Scheme 6). When 80 equivalents of D_2_O were included under otherwise standard reaction conditions, **2a** was obtained with 43% deuterium incorporation in the benzylic position (Scheme 6a). No other positions showed any deuterium incorporation. Additionally, the recovered 4-nitrohydrocinnamic acid starting material exhibited 78% deuterium content at the benzylic position. Reactions including LiOAc in the absence of CuOAc and MnO_2_ provided no styrene product, and only the starting material was recovered with 70% deuterium incorporation into the benzylic position, demonstrating the benzylic activation to occur via deprotonation and highlighting the importance of CuOAc for the formation of styrene. Measurement of the rates of styrene formation revealed much faster product formation in the presence of LiOAc (123 mM h^–1^) than in the absence (5 mM h^–1^), indicating that product-forming deprotonation likely occurs by LiOAc to generate a benzylic anion that is then trapped by copper. The H/D exchange experiments were also conducted in the absence of D_2_O using the mono-deuterated substrate **1a-*d*_1_** (Scheme 6b). Under these conditions, the styrene product was formed with only 12% deuterium incorporation, suggesting decreased reversibility in the absence of D_2_O (see the SI for full details).

**Figure 1:**
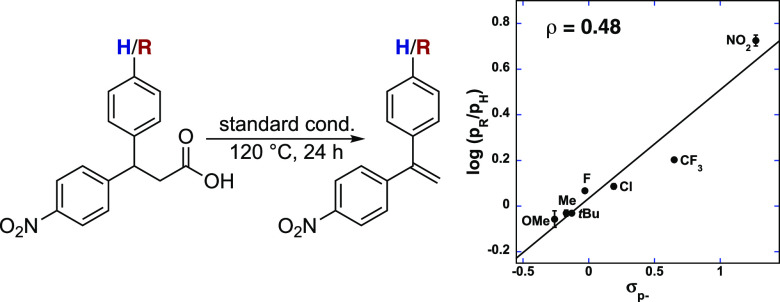
Hammett plot from competition experiments of **1f** and **1g–1k** (0.05 mmol) with CuOAc (0.02 mmol) bpy (0.02 mmol), MnO_2_ (0.2 mmol), LiOAc (0.2 mmol) in DMA (1 mL) for 30 min at 120 °C. Yields obtained from ^1^H NMR spectroscopy with 1,3,5-trimethoxybenzene as an internal standard.

Kinetic isotope effect measurements were obtained from the rate determination of the independent reactions of **1a** and **1a-*d*_2_** (*k*_H_/*k*_D_ = 2.6, Scheme 6c) as well as from the intermolecular competition between **1a** and **1a-*d*_2_** ([*P*_H_]/[*P*_D_] = 2.8, Scheme 6d). Both kinetic isotope effect (KIE) values are consistent with a C–H bond cleavage step that occurs at or before the rate-determining step of the reaction.^31^ Additionally, competition studies of differently *para*-substituted 3,3-diphenylpropionic acids revealed significant dependence on the electronic nature of the substrate. The competition Hammett correlation reveals preferential reaction of electron-deficient substrates (ρ = 0.48) (Figure 1). These data, when combined, support a selective deprotonation at the benzylic position that occurs prior to the decarboxylation step.

**Scheme 7:**
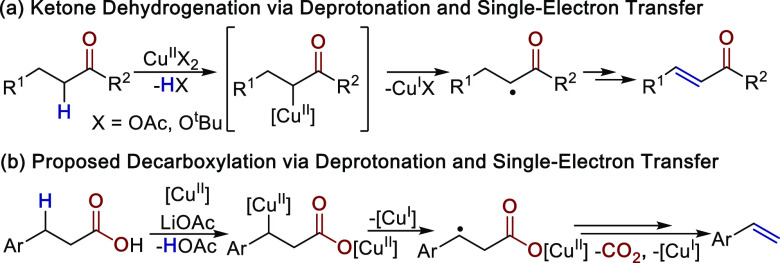
Single-Electron Pathways for Copper-Catalyzed Dehydrogenation and Decarboxylation

In related copper-catalyzed dehydrogenation reactions reported by Su^24^ and Dong,^27^ deprotonation alpha to a carbonyl leads to a proposed copper-enolate species. Both studies suggest that homolysis of the copper-alkyl intermediate would generate a reactive alkyl radical that subsequently generates the product (Scheme 7a). Thus, for our reactions, we imagined that a similar single-electron transfer between the benzylic anion and copper might generate a benzylic radical intermediate capable of undergoing rapid radical decarboxylation to afford the final alkene product (Scheme 7b). This general reaction pathway has been proposed recently for the enzymatic decarboxylation reactions of fatty acids to terminal olefins by cytochrome P450.^32^ Related radical-based decarboxylation steps have also been implicated in the decarboxylative cross-coupling reactions of cinnamic acids.^29^

**Scheme 8:**
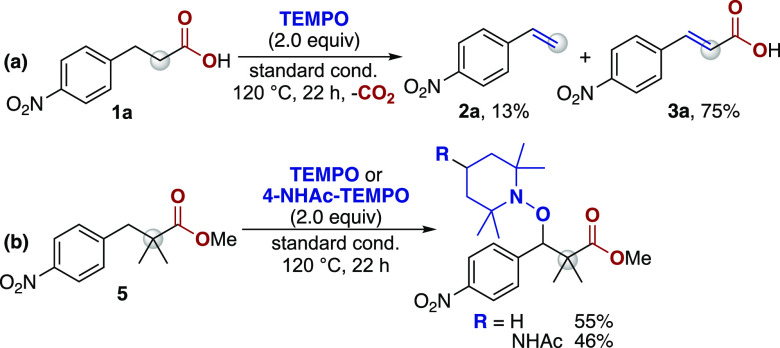
Radical Trapping Experiments Yields obtained from ^1^H NMR spectroscopy with 1,3,5-trimethoxybenzene or methyl 3,5-dinitrobenzoate as the internal standard. Reaction conditions: **1a** or **5** (0.1 mmol), TEMPO (2.0 equiv) or 4-NHAc-TEMPO (2.0 equiv), CuOAc (20 mol %), bpy (20 mol %), MnO_2_ (2.0 equiv), LiOAc (2.0 equiv) in DMA (1 mL) for 22 h at 120 °C.

To probe the possible intermediacy of a benzylic radical, we employed TEMPO as a radical trapping agent. When TEMPO (2.0 equiv) is included under our standard reaction conditions with **1a**, no TEMPO-trapped products were observed, and instead, the dehydrogenation product (**3a**) was generated in 75% yield (Scheme 8a). In contrast, when 2,2-dimethyl-3-(4-nitrophenyl)propanoic acid methyl ester (**5**) is treated with TEMPO or 4-acetamido-TEMPO under the standard reaction conditions, the benzylic TEMPO adducts were formed in 55% and 46% yields, respectively (Scheme 8b). In the absence of CuOAc, only trace amounts of the TEMPO adduct were observed, highlighting the importance of copper in the radical-forming step(s). Overall, these data provide strong support for a radical decarboxylation pathway that is initiated by deprotonation and single-electron transfer to copper to provide the benzylic radical intermediate.

## Conclusions

In conclusion, we have developed a copper-catalyzed decarboxylative elimination reaction of hydrocinnamic acids to generate styrenes. The reaction pathway differs from recently reported dehydrogenative decarboxylation reactions which rely on single-electron oxidation of the carboxylic acid to generate an acyloxy intermediate. Instead, this reaction proceeds via benzylic deprotonation, followed by radical decarboxylation, offering a new reaction manifold for decarboxylative elimination reactions.

## Data Availability

The data underlying this study are available in the published article and its supporting information.

## Supporting Information Available

Experimental details; spectral data; and ^1^H and ^13^C NMR spectra (PDF)
