# Supplementary material for: Copper-Catalyzed Decarboxylative Elimination of Carboxylic Acids to Styrenes
Source: J Org Chem. 2023 Jan 20;88(3):1713–9. doi: 10.1021/acs.joc.2c02705 (PMC10032571; doi:10.1021/acs.joc.2c02705)
Supplement: Supplementary file 1 — jo2c02705_si_001.pdf [file jo2c02705_si_001.pdf]

Supporting Information For

Copper-Catalyzed Decarboxylative Elimination of Carboxylic Acids to Styrenes

Michael P. Stanton and Jessica M. Hoover*
C. Eugene Bennett Department of Chemistry, West Virginia University, Morgantown, WV, 26506-6045
Jessica.Hoover@mail.wvu.edu

Table of Contents **Page**

I.	General Considerations	S2
II.	Optimization of the Catalytic Decarboxylative Elimination Reaction	S2
III.	Optimization of the Stoichiometric Decarboxylative Elimination Reaction	S5
IV.	Extended Scope of Substrates Tested Under Stoichiometric Conditions	S9
V.	Synthesis of Deuterated Substrates	S10
VI.	Mechanistic Studies	S13
	A. Isotopic Labeling Experiments	S13
	B. Kinetic Isotope Effect Experiments	S17
	C. Hammett Competition Experiments	S22
	D. Radical Trapping Experiments	S25
	E. Initial Rate Experiments	S27
VII.	Synthesis of Substrates Arylated at the Benzylic Position	S29
VIII.	Characterization of Hydrocinnamic Acid Starting Materials **(1)**	S31
IX.	General Procedure for the Catalytic Decarboxylative Elimination Reaction **(2)**	S40
X.	Characterization of Decarboxylative Elimination Products **(2)**	S42
XI.	References	S54
XII.	^{1}H and ^{13}C NMR Spectra of Starting Materials **(1)**	S56
XIII.	^{1}H and ^{13}C NMR Spectra of Decarboxylative Elimination Products **(2)**	S76

I. General Considerations

All manipulations were performed using standard Schlenk or glovebox techniques under a nitrogen atmosphere. All solvents (including dry DMA) were purchased from Alfa-Aesar, Fisher, or Cambridge Isotope Laboratories (deuterated solvents) and used as received. DMF and toluene were taken from a Pure Process Technology solvent system where solvent is passed through activated alumina with a pressure of argon.

All NMR spectra were recorded on a Varian INOVA 600 MHz spectrometer or an Agilent 400 MHz spectrometer at ambient temperature. Chemical shifts (δ) are given in parts per million (ppm) and referenced to the residual solvent signal ($CDCl_3$: 7.26 ppm (^1H) and 77.2 ppm (^{13}C), DMSO-d_6: 2.50 ppm (^1H) and 39.5 ppm (^{13}C));[1,2] all coupling constants (*J*) are reported in Hz. High resolution mass spectra were obtained on a Thermofisher Scientific Q Exactive Mass Spectrometer. IR spectra were recorded on a PerkinElmer (Spectrum 100) FT-IR spectrometer. Column chromatography was performed using Silicycle Silica Flash P60 silica gel.

II. Optimization of the Catalytic Decarboxylative Elimination Reaction

Representative Procedure for the Optimization of the Catalytic Decarboxylative Elimination Reaction. On the benchtop, to an oven dried 4-mL vial with a stir bar was added 4-nitrohydrocinnamic acid **1a** (19.5 mg, 0.100 mmol), CuOAc (2.4 mg, 0.020 mmol), 2,2'-bipyridine (3.1 mg, 0.020 mmol), MnO_2 (17.6 mg, 0.200 mmol) and LiOAc (13.2 mg, 0.200 mmol). The reaction vial was then taken into a nitrogen-filled glovebox where dry DMA (1.0 mL) was added, and the vial was sealed under nitrogen with a PTFE-lined cap and wrapped with electrical tape. The vial was removed from the glovebox, transferred to a pre-heated aluminum block, and stirred for 24 h at 120 °C. Upon completion, the reaction vial was cooled to room temperature. The solution was diluted with ethyl acetate (15 mL) and transferred to a separatory funnel, following which 1 M HCl (100 mL) was added, and the organic layer was extracted with ethyl acetate (3 x 30 mL). The combined organic layers were dried over Na_2SO_4 and gravity filtered through filter paper. The solvent was removed under reduced pressure and a 1,3,5-trimethoxybenzene (5.0 mg, 0.030 mmol) NMR standard was added to the sample for ^1H NMR analysis.

Table S1. Optimization of the Copper Loading and Oxidant under Catalytic Conditions

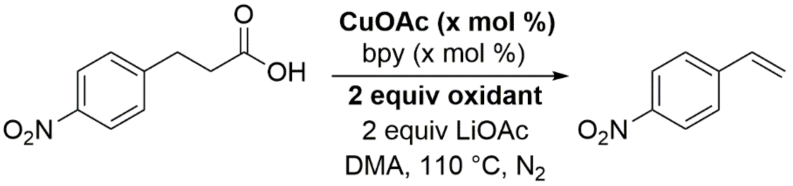

entry	CuOAc loading (mol %)	oxidant	yield **2a** (%)[a]
1	20	-	13
2	120	-	82
3	20	NFSI	0
4	20	$Na_2S_2O_8$	0
5	20	*t*BuOO*t*Bu	45
6	120	*t*BuOO*t*Bu	75
7	10	MnO_2	48
8	20	MnO_2	83

[a]Reaction conditions: **1a** (0.100 mmol) in DMA (1 mL). ^{1}H NMR yield determined from integration using 1,3,5-trimethoxybenzene as an internal standard.
NFSI = *N*-fluorobenzenesulfonimide

Table S2. Optimization of the Reaction Temperature under Catalytic Conditions

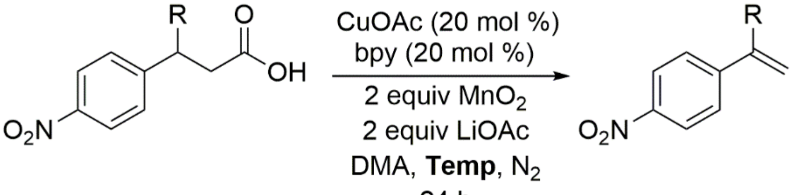

entry	substrate (R= H or CH_3)	temperature (°C)	product yield (%)[a]	starting material recovered (%)[a]
1	H (**1a**)	110	83	<5 %
2	CH_3 (**1b**)	110	72	23
3	H (**1a**)	120	72	19
4	CH_3 (**1b**)	120	79	16

[a]Reaction conditions: **1a or 1d** (0.100 mmol) in DMA (1 mL). ^{1}H NMR yield determined from integration using 1,3,5-trimethoxybenzene as an internal standard.

Table S3. Optimization of the Base under Catalytic Conditions

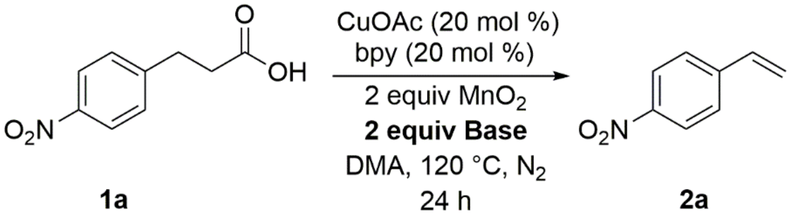

entry	base (2 equiv)	yield **2a** (%)[a]	**1a** recovered (%)[a]
1	-	53	36
2	LiOAc	72	19
3	NaOAc	71	22
4	KOAc	82	0
5	Li_2CO_3	40	44
6	K_2CO_3	60	0
7	Na_2CO_3	76	0
8	DMAP	64	34

[a]Reaction conditions: **1a** (0.100 mmol) in DMA (1 mL). ^1H NMR yield determined from integration using 1,3,5-trimethoxybenzene as an internal standard. (Despite KOAc giving higher product yield, LiOAc was carried forward due to the better overall mass balance observed.)

Table S4. Optimization of the Copper Catalyst under Catalytic Conditions

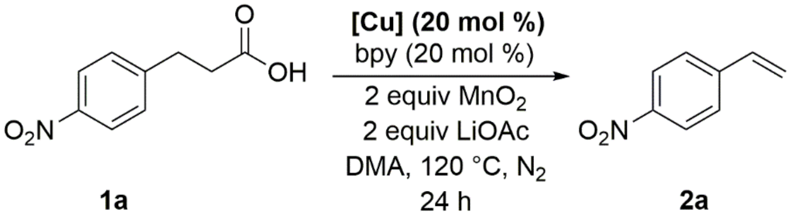

entry	[Cu] (20 mol %)	yield **2a** (%)[a]	**1a** recovered (%)[a]
1	CuI	31	44
2	CuBr•DMS	48	43
3	CuOAc	76	0
4	$Cu(OTf)_2$	61	26
5	$Cu(OAc)_2$	78	0

[a]Reaction conditions: **1a** (0.100 mmol) in DMA (1 mL). ^1H NMR yield determined from integration using 1,3,5-trimethoxybenzene as an internal standard. DMS = dimethyl sulfide

III. Optimization of the Stochiometric Decarboxylative Elimination Reaction

Representative Procedure for the Optimization of the Stochiometric Decarboxylative Elimination Reaction. On the benchtop, to an oven dried 4-mL vial with a stir bar was added 4-nitrohydrocinnamic acid **1a** (19.5 mg, 0.100 mmol), CuOAc (14.7 mg, 0.120 mmol), 2,2'-bipyridine (18.7 mg, 0.120 mmol), and LiOAc (13.2 mg, 0.200 mmol). The reaction vial was then taken into a nitrogen filled glovebox where dry DMA (1.0 mL) was added, and the vial was sealed under nitrogen with a PTFE-lined cap wrapped with electrical tape. The vial was then removed from the glove box, transferred to a pre-heated aluminum block and the reaction mixture was stirred for 24 h at 110 °C. Upon completion, the reaction vial was cooled to room temperature. A 100-µL aliquot was taken from the reaction mixture and added to a Pasteur pipette containing a cotton ball and ~2 cm silica plug. $CDCl_3$ (0.7 mL) was added to the Pasteur pipette to elute the sample into an NMR tube for analysis. A 1,3,5-trimethoxybenzene (1.0 mg, 0.0059 mmol) NMR standard was added directly to the tube and the sample was analyzed by ^1H NMR spectroscopy to obtain reaction yields.

Table S5. Optimization of the Stochiometric Copper Reaction Conditions

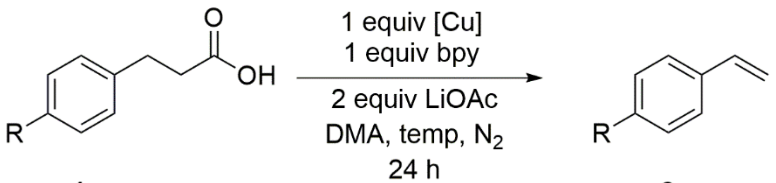

1 **2**

entry	R	[Cu]	temp (°C)	yield **2** (%)[a]
1[b]	H	$Cu(OAc)_2$	140	4
2[b]	*t*-Bu	$Cu(OAc)_2$	140	11
3[b]	NO_2	$Cu(OAc)_2$	140	30
4	NO_2	$Cu(OAc)_2$	140	50
5	NO_2	$Cu(OAc)_2$	110	68
6	NO_2	$Cu(OTf)_2$	110	72
7	NO_2	$CuCl_2$	110	0
8	NO_2	CuOAc	110	77
**9**[c]	**NO_2**	**CuOAc**	**110**	**82**
10	NO_2	-	110	0

[a]Reaction conditions: **1** (0.1 mmol) in DMA (1 mL). ^1H NMR yield with 1,3,5-trimethoxybenzene as internal standard. [b] 2 equiv TEMPO used.[c]1.2 equiv [Cu] and bpy used.

Table S6. Optimization of the Solvent under Stoichiometric Conditions

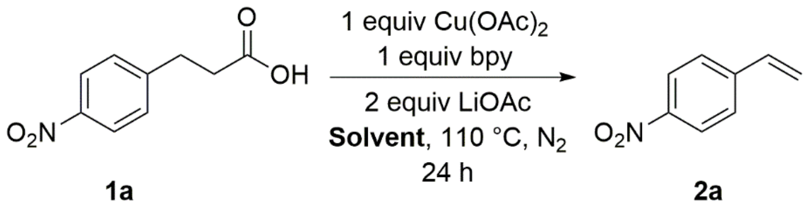

entry	solvent	yield **2a** (%)[a]
1	toluene	0
2	MeCN	3
3	NMP	16
4	DMSO	58
**5**	**DMF**	**73**
**6**	**DMA**	**70**

[a]Reaction conditions: **1a** (0.1 mmol) in DMA (1 mL). ^{1}H NMR yield determined from a reaction mixture aliquot by integration using 1,3,5-trimethoxybenzene as an internal standard.

Table S7. Optimization of the Reaction Atmosphere under Stoichiometric Conditions

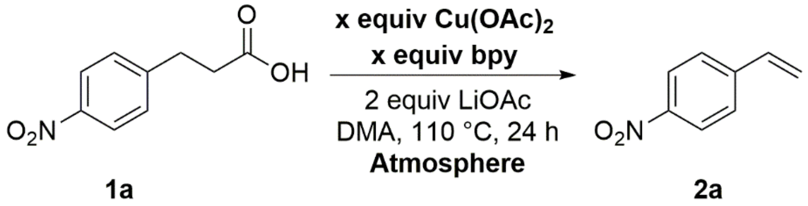

entry	Cu loading (equiv)	atmosphere	yield **2a** (%)[a]
1	0.2	air	2
2	1	air	32
3	0.2	O_2	0
4	1	O_2	0
5	0.2	N_2	17
**6**	**1**	$\mathbf{N_2}$	**68**

[a]Reaction conditions: **1a** (0.1 mmol) in DMA (1 mL). ^{1}H NMR yield determined from a reaction mixture aliquot by integration using 1,3,5-trimethoxybenzene as an internal standard.

Table S8. Optimization of the Reaction Temperature under Stoichiometric Conditions

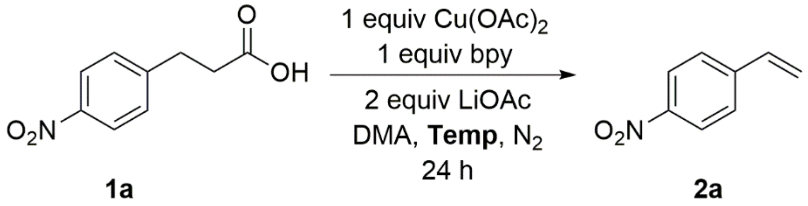

entry	temp (°C)	yield **2a** (%)[a]
1	80	47
2	110	70
**3**	**120**	**78**
4	140	50

[a]Reaction conditions: **1a** (0.1 mmol) in DMA (1 mL). ^{1}H NMR yield determined from a reaction mixture aliquot by integration using 1,3,5-trimethoxybenzene as an internal standard.

Table S9. Optimization of the Ligand under Stoichiometric Conditions

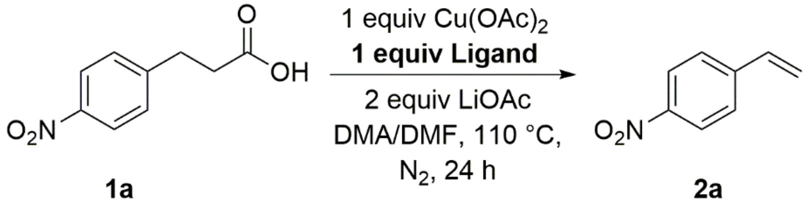

entry	solvent	ligand	yield **2a** (%)[a]
**1**	**DMA**	**bpy**	**70**
2	DMF	bpy	64
3	DMA	1,10-phen	68
4	DMF	1,10-phen	57
**5**	**DMA**	**DABCO**	**76**
6	DMF	DABCO	68
7	DMA	DMAP	70
8	DMF	DMAP	64
9	DMA	PPh_3	55
10	DMA	diazafluorenone	59

[a]Reaction conditions: **1a** (0.1 mmol) in DMA (1 mL) or DMF (1 mL). ^{1}H NMR yield determined from a reaction mixture aliquot by integration using 1,3,5-trimethoxybenzene as an internal standard. DABCO = 1,4-diazabicyclo[2.2.2]octane, DMAP = 4-dimethylaminopyridine.

Table S10. Optimization of Copper Source with bpy under Stochiometric Conditions

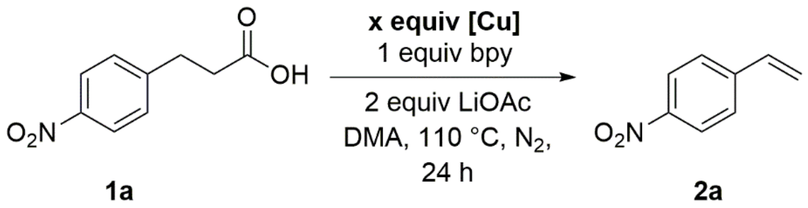

entry	Cu source	Cu loading (equiv)	yield **2a** (%)[a]
1	$Cu(OAc)_2$	1	70
2	$Cu(OTf)_2$	1	76
3	$CuCl_2$	1	0
4	Cu(OTf)	1	60
5	CuCl	1	28
6	CuI	1	20
7	Cu(OAc)	1	77
**8**	**Cu(OAc)**	**1.2**	**84**

[a]Reaction conditions: **1a** (0.1 mmol) in DMA (1 mL). ^{1}H NMR yield determined from a reaction mixture aliquot by integration using 1,3,5-trimethoxybenzene as an internal standard.

Table S11. Optimization of Copper Source with DABCO under Stoichiometric Conditions

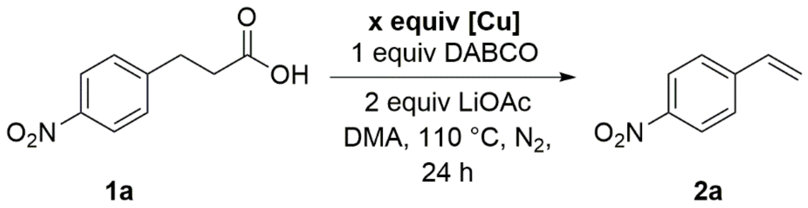

entry	Cu source	Cu loading (equiv)	yield **2a** (%)[a]
**1**	**$Cu(OAc)_2$**	**1**	**76**
2	$Cu(OTf)_2$	1	37
3	$CuCl_2$	1	6
4	Cu(OTf)	1	33
5	CuCl	1	29
6	CuI	1	20
7	Cu(OAc)	1	63

[a]Reaction conditions: **1a** (0.1 mmol) in DMA (1 mL). ^{1}H NMR yield determined from a reaction mixture aliquot by integration using 1,3,5-trimethoxybenzene as an internal standard. DABCO = 1,4-diazabicyclo[2.2.2]octane.

Table S12. Optimization of Base Loading under Stoichiometric Conditions

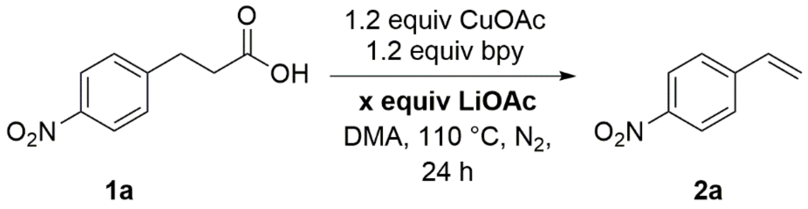

entry	LiOAc loading (equiv)	yield **2a** (%)[a]
1	0	73
2	1	71
**3**	**2**	**82**
4	3	71

[a]Reaction conditions: **1a** (0.1 mmol) in DMA (1 mL). ^{1}H NMR yield determined from a reaction mixture aliquot by integration using 1,3,5-trimethoxybenzene as an internal standard.

IV. Extended Scope of Substrates Tested Under Stoichiometric Conditions

Scheme S1. Scope of Substituted Phenyl Propionic Acids[a]

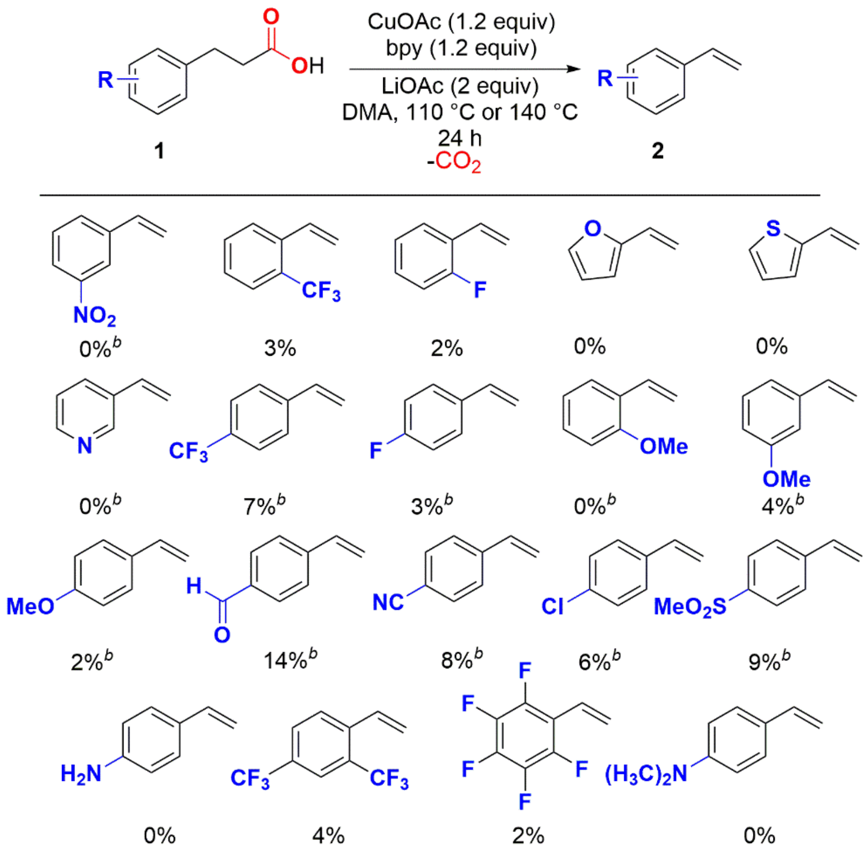

[a]Reaction conditions: **1** (0.100 mmol) in DMA (1 mL). ^{1}H NMR yield determined from a reaction mixture aliquot by integration using 1,3,5-trimethoxybenzene as an internal standard. [b]Reaction conducted at 140 °C.

V.Synthesis of Deuterated Substrates

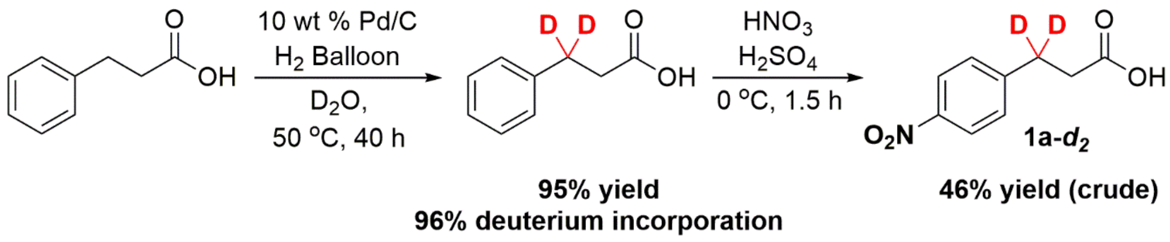

Benzylic Deuteration of Hydrocinnamic Acid. For selective deuteration at the benzylic position a procedure by Sajiki and coworkers was followed.[3] To a test tube fit with a stir bar, hydrocinnamic acid (1.51 g, 10.1 mmol) was added along with 10% by weight Pd/C (150 mg). The tube was then sealed with a septum and evacuated with a needle. D_2O (10 mL, sparged with nitrogen prior to use) was then added via syringe. Next, an H_2 balloon attached to a syringe and needle was inserted through the septum and the reaction was stirred for 40 h at 50 °C. Upon completion, the reaction was cooled to room temperature. The mixture was diluted with DI water (20 mL) and passed through a celite pad by washing with ethyl acetate (200 mL) and DI water (100 mL). The eluents were transferred to a separatory funnel and extracted with ethyl acetate (3 x 40 mL). The organic layer was dried over Na_2SO_4, filtered, and solvent was removed under reduced pressure affording the deuterated product in 95% yield with 96% deuterium incorporation.

Nitration of Deuterated Hydrocinnamic Acid. The deuterated hydrocinnamic acid substrate (1.43 g, 9.40 mmol) was added to a 125-mL flask along with a stir bar. The flask was then placed in an ice bath where concentrated H_2SO_4 (2 mL) was added dropwise to the flask over 15 minutes. Next concentrated HNO_3 (0.75 mL) was added dropwise to the flask over 15 minutes, after which the mixture was stirred for an additional 1 h in the ice bath. Upon completion, the reaction was quenched with ice cold water (100 mL), affording a white solid. The solid was then filtered, washed with copious amounts of water (~200 mL), and dried under vacuum. The solid was a mixture of nitrated products (*para*-substituted, *ortho*-substituted, *di*-substituted) which was used below without further purification (0.86 g, crude yield = 46 %).

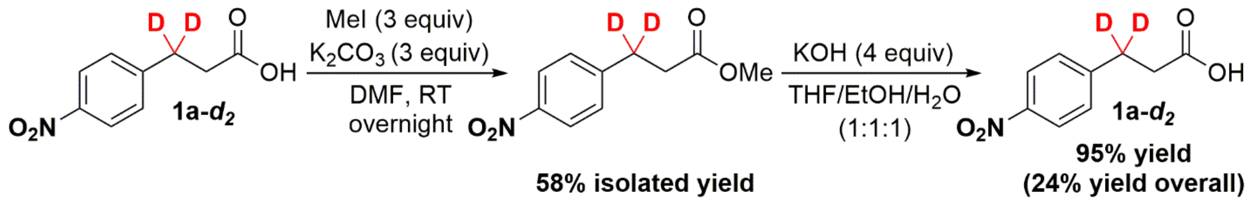

Esterification of Nitration Products. Due to the difficulty of separating the mixture of carboxylic acids obtained by nitration, the products were converted to the corresponding methyl esters prior to purification. A 100-mL round bottom flask fitted with a stir bar was charged with the crude mixture of nitrated products (0.86 g), K_2CO_3 (1.81 g, 13.1 mmol), MeI (1.86 g, 13.1 mmol), and anhydrous DMF (10 mL). The reaction was stirred under air at room temperature overnight. After completion, brine (200 mL) was added and the resulting mixture was extracted with ethyl acetate (3 x 30 mL). The organic layers were dried over Na_2SO_4, filtered, and the solvent was removed under vacuum. The residue was purified via silica gel column chromatography (hexanes: ethyl acetate, 12:1 v/v) to afford the corresponding *para*-nitro-substituted methyl ester of hydrocinnamic acid (0.45 g, 58 % isolated yield after column chromatography).

Hydrolysis of Methyl 4-Nitrohydrocinnamate. A 100-mL round bottom flask was charged with the methyl 4-nitrohydrocinnamate (450 mg, 2.1 mmol), THF/EtOH/H_2O (12 mL total, 1:1:1 by volume), and KOH (4 equiv, 480 mg, 8.5 mmol). The mixture was stirred at room temperature and monitored by TLC until all the starting material was consumed. The reaction was acidified with 2 M HCl until a pH of 2 was achieved, as determined by pH paper. The acidification was accompanied by formation of a precipitate. The reaction mixture was transferred to a separatory funnel and extracted with ethyl acetate (3 x 50 mL), dried over anhydrous Na_2SO_4 and filtered. The solvent was removed under reduced pressure, furnishing 4-nitrohydrocinnamic acid deuterated at the benzylic position with 96% D incorporation (0.40 g, 2.0 mmol, 95% yield, 24% yield starting from unsubstituted hydrocinnamic acid). The product was used without further purification.

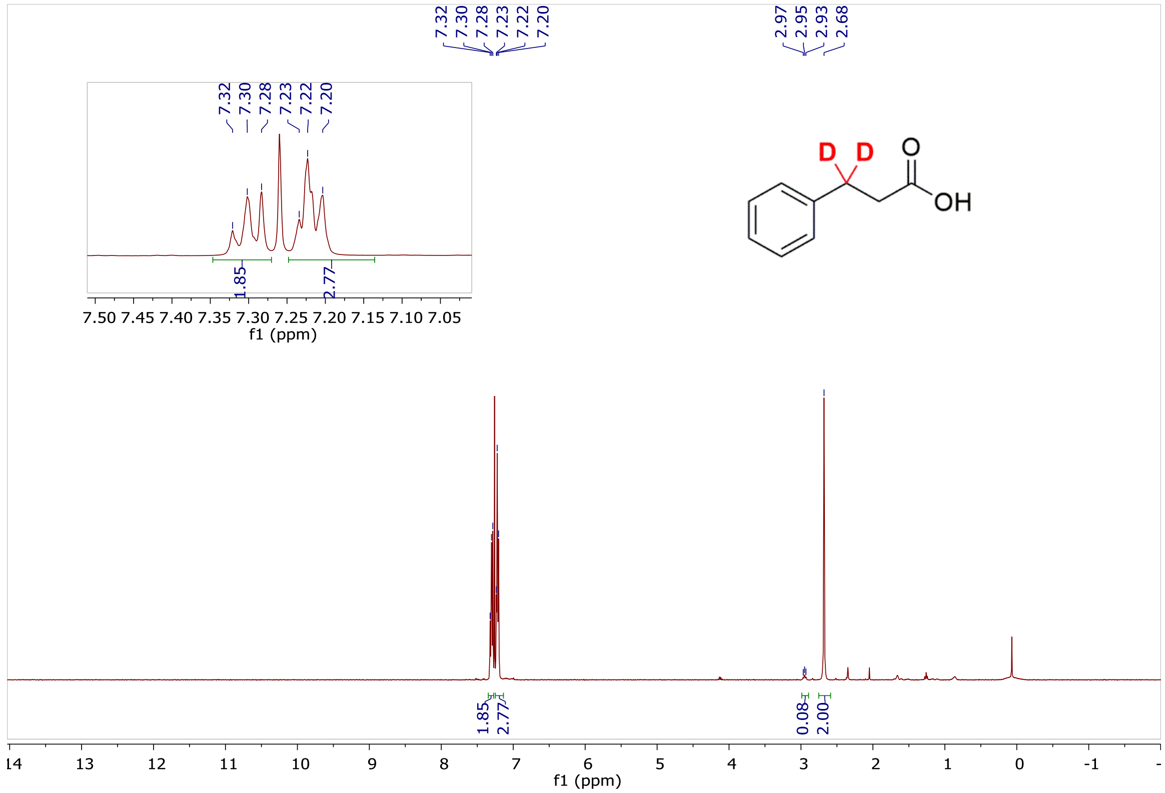

Figure S1. ^1H NMR spectrum of the hydrocinnamic acid product (**1a-*d*$_2$**) after the deuteration reaction. The triplet (2.95 ppm) integrating to 0.08 represents the remaining H atoms at the benzylic position.

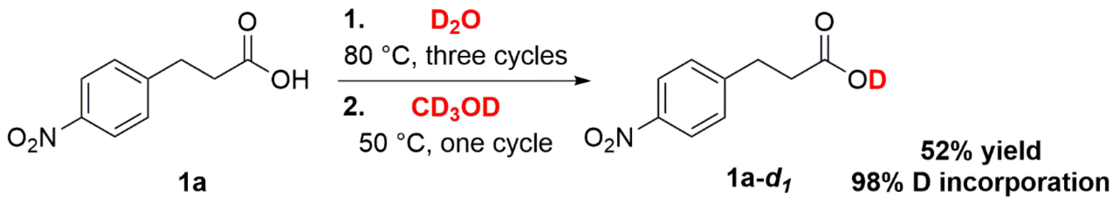

Synthesis of 4-Nitrohydrocinammic Acid 1a-*d*$_1$ (RCO$_2$D). 4-Nitrophenylpropionic acid (488 mg, 2.50 mmol) was stirred in D_2O (5 mL) at 80 °C for two hours in a 20 mL scintillation vial. After the allotted time, the product was extracted with $CDCl_3$ (5 mL x 2, solvent dried over activated 4 Å molecular sieves for 2 hours). Any remaining D_2O was decanted off the organic layer. The organic layers were then concentrated, and the process was repeated two additional times. Next, the product was heated at 70 °C under vacuum to remove remaining D_2O. The product (a yellow solid) was then transferred to a 50-mL 3-neck round bottom Schlenk flask. After evacuating and backfilling the flask with N_2, CD_3OD (3 mL) was added and the mixture was stirred at 50 °C for 2 hours. After the allotted time, the solvent was removed by vacuum and the product was sealed under a nitrogen atmosphere and transferred to a nitrogen-filled glovebox. The product was characterized in dry DMSO-*d*$_6$ (dried over 4 Å molecular sieves). 4-Nitrophenylpropionic acid **1a-*d*$_1$** was synthesized in 52% yield (254.8 mg, 1.299 mmol) with 98% D incorporation as a yellow solid.

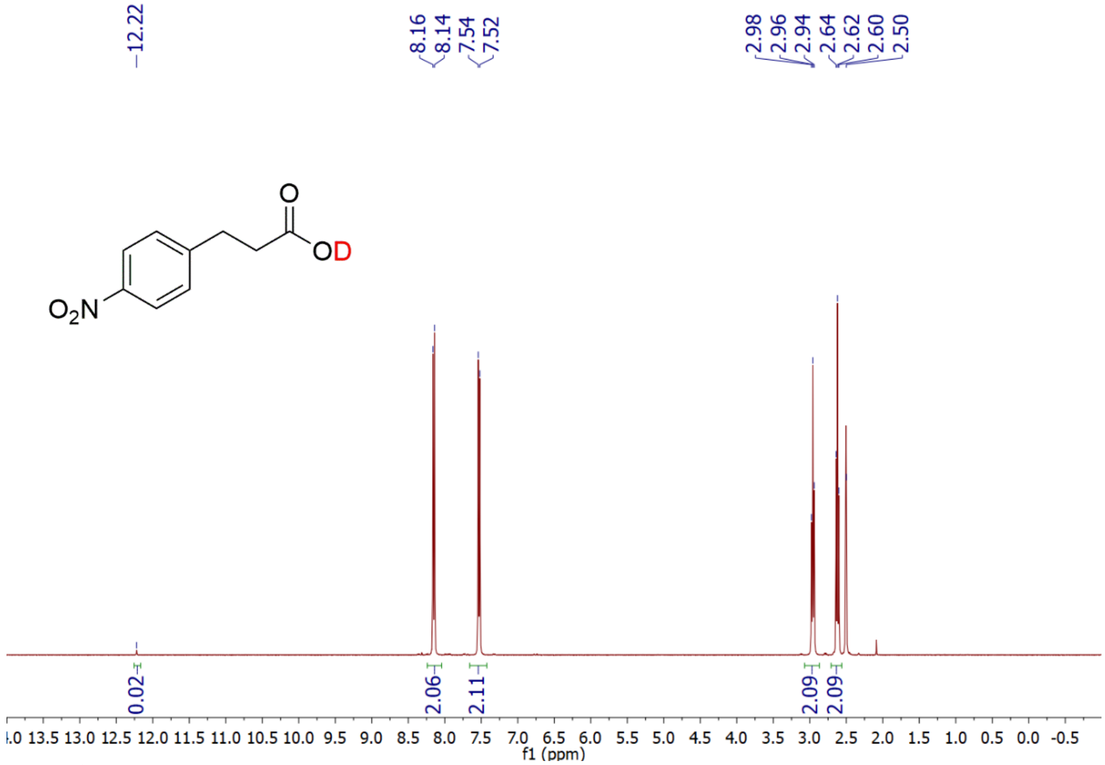

Figure S2. ^{1}H NMR spectrum of deuterated 4-nitrohydrocinnamic acid (**1a-*d*$_1$**) in dry DMSO-*d*$_6$.

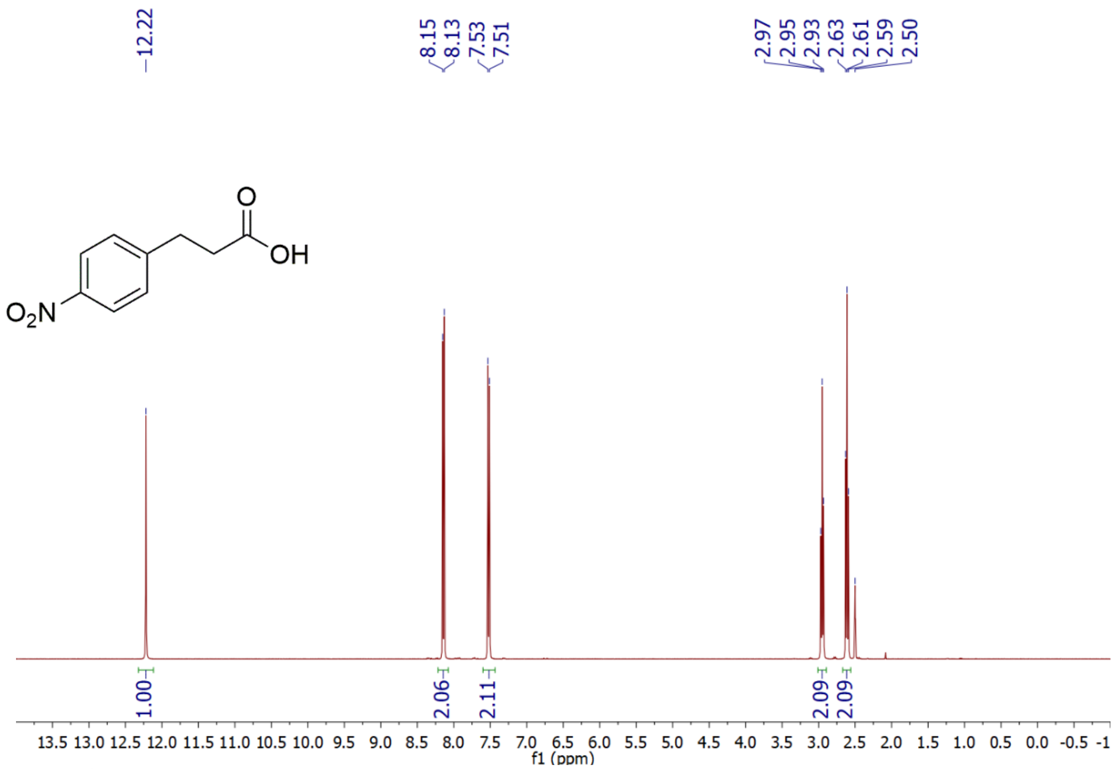

Figure S3. ^{1}H NMR spectrum of 4-nitrohydrocinnamic acid (**1a**) in dry DMSO-d_6.

VI. Mechanistic Studies

A. Isotopic Labeling Experiments

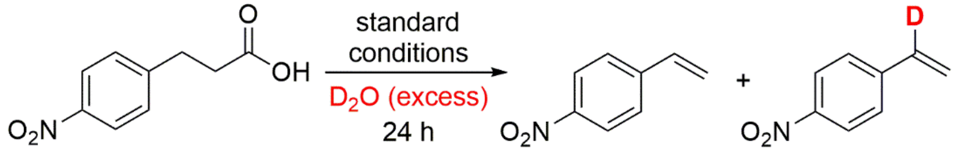

1a **2a** (39%) **2a-d_1** (29%)

Procedure for the Hydrogen-Deuterium Exchange Experiment with D_2O. On the benchtop, to an oven dried 4-mL vial was added a stir bar, 4-nitrohydrocinnamic acid **1a** (19.5 mg, 0.100 mmol), CuOAc (2.5 mg, 0.020 mmol), 2,2'-bipyridine (3.4 mg, 0.020 mmol), MnO_2 (17.4 mg, 0.200 mmol) and LiOAc (13.2 mg, 0.200 mmol). The reaction vial was then taken into a nitrogen-filled glovebox where dry DMA (1 mL) was added, and the vial was sealed under nitrogen with a septum wrapped with electrical tape. The vial was removed from the glovebox and 3 drops (~80 equiv) of D_2O were added *via* syringe (prior to addition, the D_2O was sparged with N_2 for 5 minutes). The vial was transferred to a pre-heated aluminum block and stirred for 24 h at 120 °C. Upon completion, the reaction vial was cooled to room temperature. The solution was diluted with ethyl acetate (15 mL) and transferred to a separatory funnel along with 2 M HCl (100 mL). The aqueous layer was extracted with ethyl acetate (3 x 30 mL). The combined organic layers were washed with brine (60 mL), dried over Na_2SO_4 and filtered. The solvent was removed under

reduced pressure. A 1,3,5-trimethoxybenzene (5.0 mg, 0.30 mmol) NMR standard was added to the sample for ^{1}H NMR analysis and the ^{1}H NMR yield was obtained by integration. The corresponding product was obtained as a mixture of **2a** and **2a-*d₁*** in 39% and 29% yields respectively (as determined by ^{1}H NMR spectroscopy) and recovered 4-nitrohydrocinnamic acid starting material (23 % yield) also showed 78% deuterium content at the benzylic position.

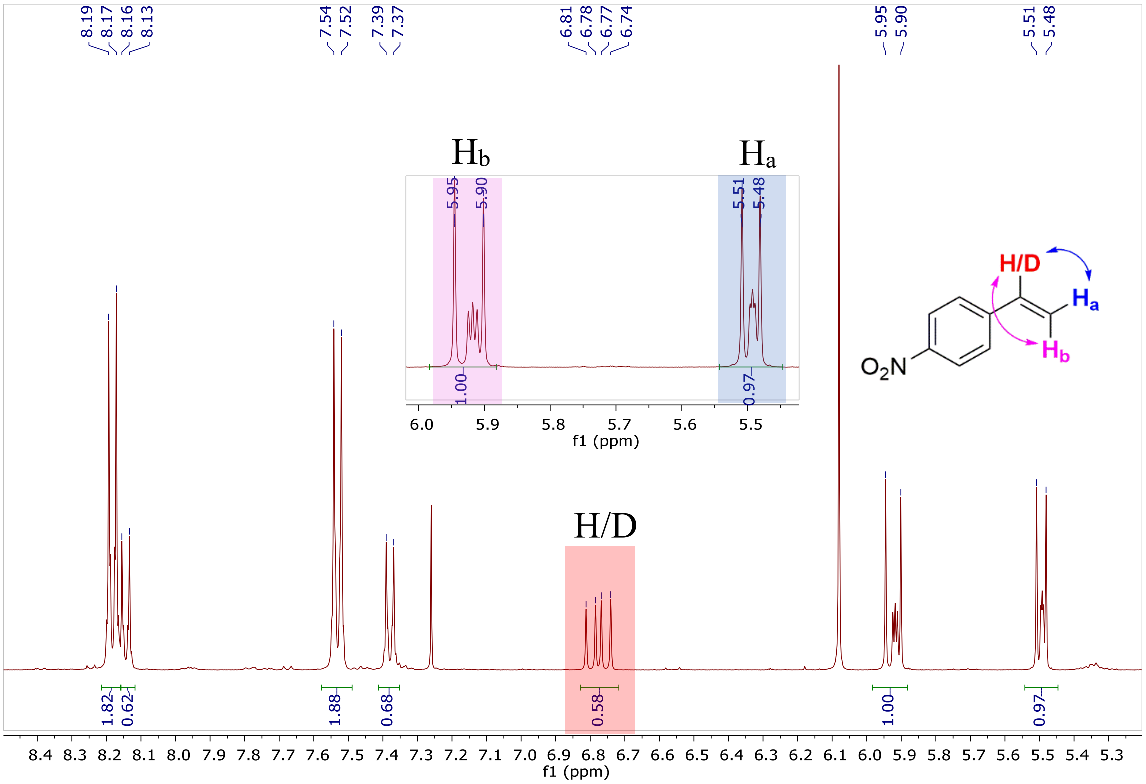

Figure S4. ^{1}H NMR spectrum of a 100 μL aliquot taken from the reaction mixture of the hydrogen-deuterium exchange experiment above. The blue triplet represents $\mathbf{H_a}$ (*cis* to H/D). The pink triplet corresponds to $\mathbf{H_b}$ (*trans* to H/D).

General Procedure for Hydrogen-Deuterium Exchange Control Reactions with 1a-*d*$_2$. An oven-dried 25-mL Schlenk flask (pear shape) fitted with a stir bar was charged with 4-nitrohydrocinnamic acid **1a** (29.2 mg, 0.150 mmol), and the appropriate masses of other reactants based on the control reaction being tested. The flask was then sealed with a septum and electrical tape, evacuated, and backfilled with nitrogen three times, after which dry DMA (1.5 mL) was added via syringe. 5 drops of D_2O were added *via* syringe. (Prior to addition, the D_2O was sparged with N_2 for 5 minutes.) The flask was then added to a pre-heated oil bath and stirred for 2 h at 120 °C. Upon completion, the reaction vessel was cooled to room temperature. The solution was diluted with ethyl acetate (30 mL) and transferred to a separatory funnel along with 1 M HCl (400 mL). The layers were separated, and the aqueous layer was extracted with ethyl acetate (3 x 30 mL). The combined organic layers were washed with brine (~250 mL), dried over Na_2SO_4 and gravity filtered into a round bottom flask. The solvent was removed under reduced pressure and a 1,3,5-trimethoxybenzene (5.00 – 9.00 mg) NMR standard was added to the sample for ^1H NMR analysis. A ^1H NMR spectrum of the crude residue was obtained in 0.7 mL $CDCl_3$ with 3 drops DMSO-d_6 and the yield was obtained by integration.

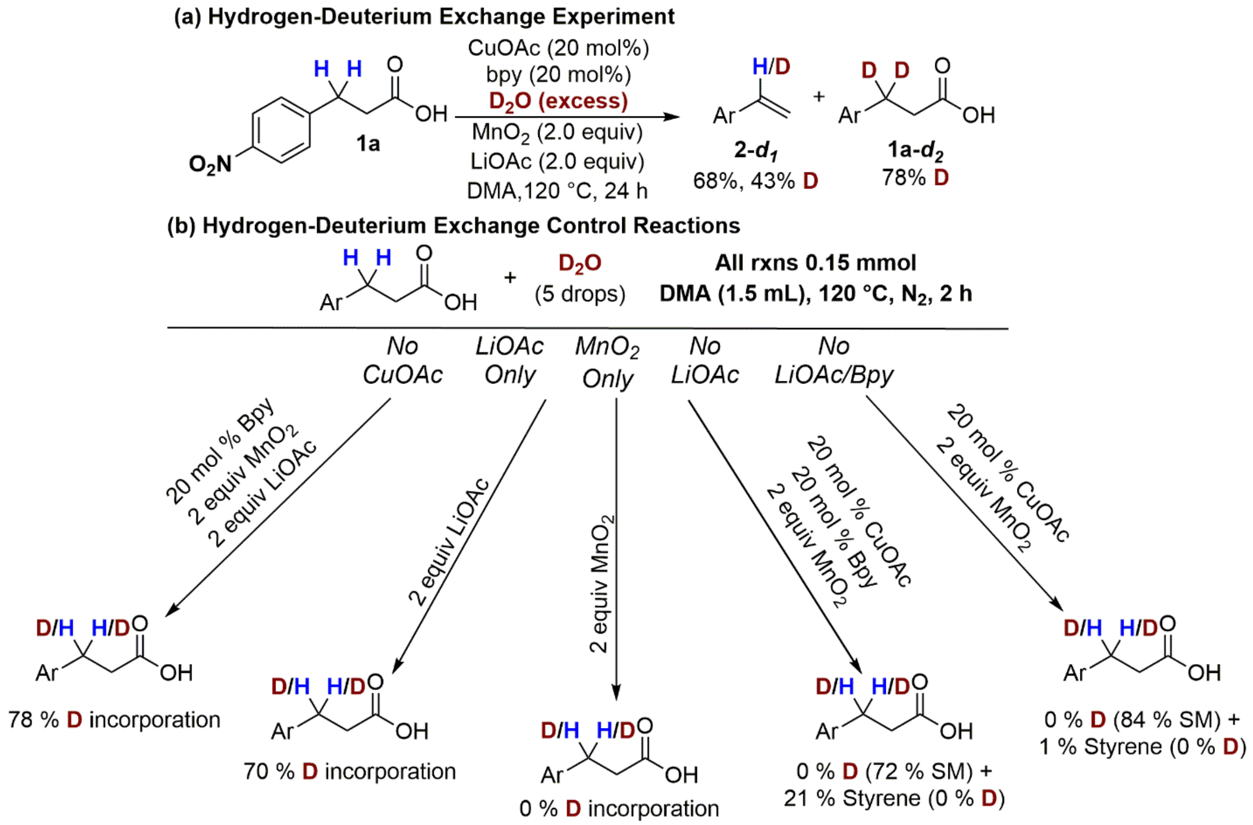

(a) Hydrogen-Deuterium Exchange Experiment from 1a-*d₁*

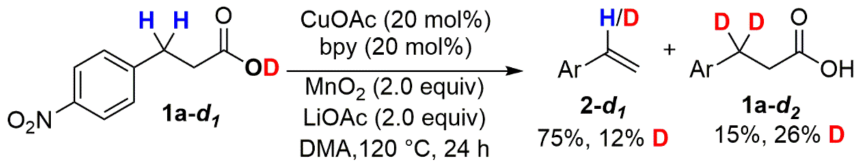

(b) H/D Exchange Experiement from 1a-*d₁* (LiOAc only)

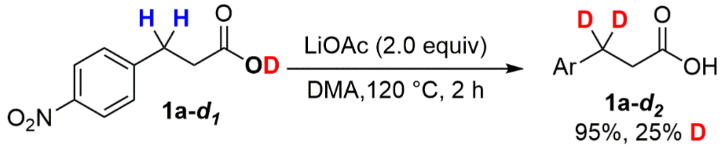

General Procedure for Hydrogen-Deuterium Exchange Reactions with 1a-*d_1*. An oven-dried 25-mL Schlenk flask (pear shape) fitted with a stir bar was charged with 4-nitrohydrocinnamic acid **1a-*d_1*** (29.4 mg, 0.150 mmol) in a nitrogen-filled glovebox, and the appropriate masses of other reactants based on the control reaction being tested. The flask was then sealed with a septum and electrical tape, evacuated, and backfilled with nitrogen, after which dry DMA (1.5 mL) was added via syringe. The flask was then added to a pre-heated oil bath and stirred for 2 h or 24 h at 120 °C. Upon completion, the reaction vessel was cooled to room temperature. The solution was diluted with ethyl acetate (30 mL) and transferred to a separatory funnel along with 1 M HCl (400 mL). The layers were separated, and the aqueous layer was extracted with ethyl acetate (3 x 30 mL). The combined organic layers were washed with brine (~250 mL), dried over Na_2SO_4 and gravity filtered into a round bottom flask. The solvent was removed under reduced pressure and a 1,3,5-trimethoxybenzene (5.00 – 9.00 mg) NMR standard was added to the sample for ^{1}H NMR analysis. A ^{1}H NMR spectrum of the crude residue was obtained in 0.7 mL $CDCl_3$ with 3 drops DMSO-*d_6* and the yield was obtained by integration.

B. Kinetic Isotope Effect Experiments

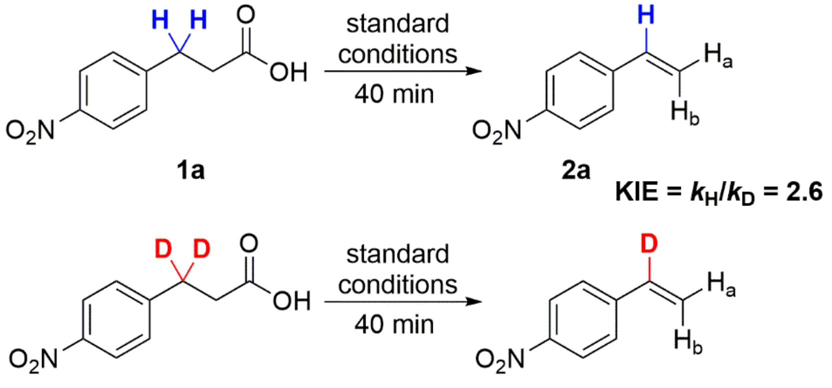

Procedure for Kinetic Isotope Effect from Parallel Reactions. To a 25-mL Schlenk tube fitted with a stir bar, 4-nitrohydrocinnamic acid **1a** (29.3 mg, 0.150 mmol), CuOAc (3.8 mg, 0.030 mmol), 2,2'-bipyridine (4.8 mg, 0.030 mmol), MnO_2 (26.2 mg, 0.300 mmol), LiOAc (19.8 mg, 0.300 mmol), and methyl 3,5-dinitrobenzoate (22.9 mg, 0.101 mmol) were added. The tube was evacuated and backfilled 3 times with nitrogen and dry DMA (1.5 mL) was added via syringe. The tube was placed in a preheated oil bath (The reaction mixture reaches 120 °C ~2 minutes after the tube is placed in the oil bath) and stirred at 500 RPM. Aliquots (100 µL) were taken from the reaction at designated timepoints (6, 9, 12, 15, 18, 21, 24, 27, 30, and 40 minutes) and transferred to a 5 ¾" filter pipette containing silica gel (~ 2 cm) and Celite (~2 cm). $CDCl_3$ (0.8 mL) was used to elute the sample directly into NMR tubes. NMR analysis was used to determine the 4-nitrostyrene concentration and yields at the various timepoints, using methyl 3,5-dinitrobenzoate as the internal standard. The process was repeated for the deuterated substrate **1a-*d₂***. Note: Better shimming of the samples was achieved when the NMR tubes containing the samples were allowed to sit in the freezer overnight and spectra were acquired the next day. This is attributed to any MnO_2 that was not removed by the filter pipette settling to the bottom of the tube.

Data Analysis. After Fourier transform of the FID, an auto-baseline correction protocol was applied to the spectra and the spectra were manually integrated. The integration values of the aromatic peak of the methyl 3,5-dinitrobenzoate standard (9.23 ppm, triplet) and the H_b proton of the styrene product (5.94 ppm) were used to calculate the concentration of product (**2a** or **2a-d_1**) in the reaction at each of the timepoints. The initial rates were determined from a linear fit of the early timepoints of the reactions (approximately the first 20 % product formation). For substrate **2a** this corresponds to timepoints from 0-15 minutes and for substrate **1a-d_2** this corresponds to timepoints from 0-27 minutes. A KIE was determined by dividing the average initial rate of three separate runs for the *proteo*-substrate **1a** by that of the average initial rate of two separate runs for the *deutero*-substrate **1a-d_2** giving a KIE of 2.6.

Table S13. Concentration values for 4-nitrostyrene from an average of 3 trials for **1a** and from an average of 2 trials for **1a-*d*$_2$** determined from ^{1}H NMR spectra integration. This data gives rise to the reaction time course (Figure S4.) and was used to determine the initial rate of the reaction.

Starting Carboxylic Acid	Time (min)	[2a] or [1a-*d*$_2$] (mM) A	[2a] or [1a-*d*$_2$] (mM) B	[2a] or [1a-*d*$_2$] (mM) C	Avg. [2a] or [1a-*d*$_2$] (mM)	Std. Dev. Of Mean	Initial Rate (mM/h)
(**1a**)	0	0	0	0	0	0	122.7
	6	8.0	10.1	10.2	9.4	1.20	
	9	17.1	16.1	16.2	16.5	0.53	
	12	23.1	24.2	23.7	23.4	0.54	
	15	30.2	30.9	29.8	30.3	0.58	
(**1a-*d*$_2$**)	0	0	0	-	0	0	47.4
	6	0.7	1.4	-	1.0	0.47	
	9	2.8	3.4	-	2.0	0.44	
	12	5.5	5.4	-	5.5	0.08	
	15	7.6	8.1	-	7.8	0.37	
	18	10.3	10.8	-	10.6	0.34	
	21	13.8	13.5	-	13.7	0.18	
	24	15.9	16.2	-	16.1	0.27	
	27	19.3	19.6	-	19.5	0.23	

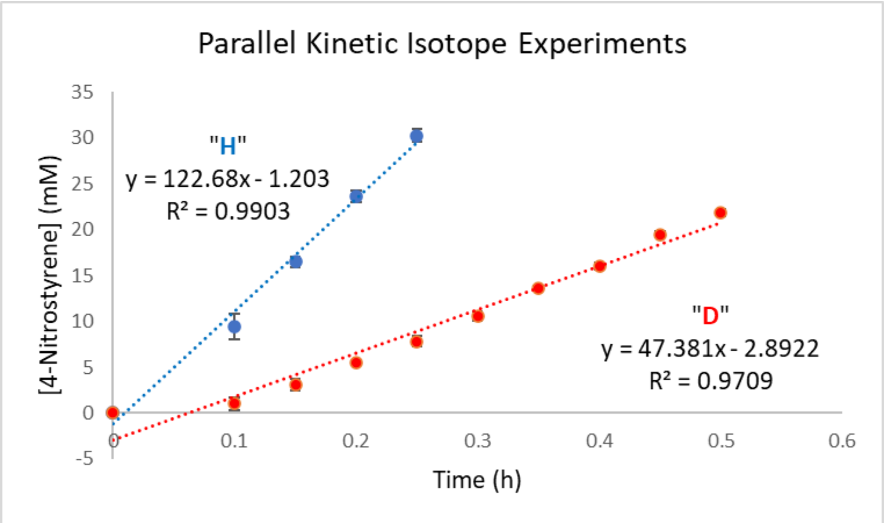

Figure S5. The blue trace (average from 3 trials) is a plot of initial rate data for the dehydrogenative decarboxylation of 4-nitrohydrocinnamic acid (**1a**) while the red trace (average 2 trials) is a plot of the initial rate data for the dehydrogenative decarboxylation of 4-nitrohydrocinnamic acid (**1a-*d*$_2$**). Associated error is given by 2 times the standard deviation divided by the square root of the number of trials ((2 x stdev)/ SQRT(# trials)).

Parallel Experiments (No LiOAc)

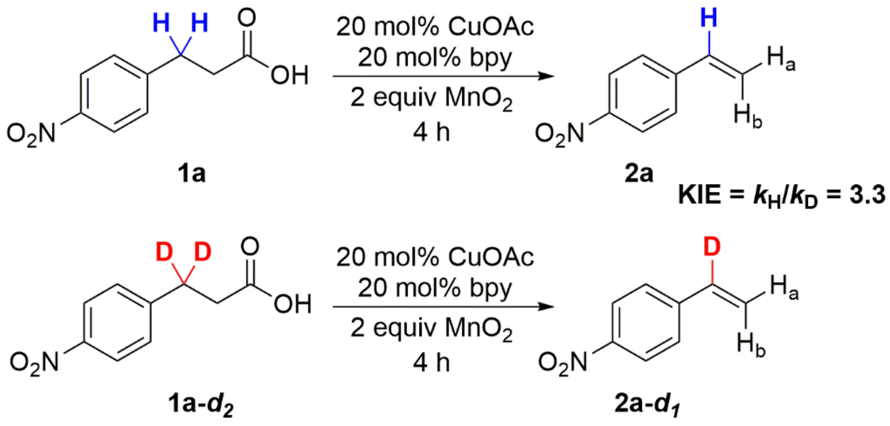

Procedure for Kinetic Isotope Effect from Parallel Reactions without LiOAc. To a 25-mL Schlenk flask fitted with a stir bar, 4-nitrohydrocinnamic acid **1a** (29.3 mg, 0.150 mmol), CuOAc (3.8 mg, 0.030 mmol), 2,2'-bipyridine (4.8 mg, 0.030 mmol), and MnO_2 (26.2 mg, 0.300 mmol) were added. The tube was evacuated and backfilled 3 times with nitrogen and dry DMA (1.5 mL) was added via syringe. The tube was placed in a preheated oil bath and stirred at 800 RPM. Aliquots (100 µL) were taken from the reaction at designated timepoints (20, 40, 60, 90, 120, 150, 180, and 240 minutes) and transferred to a 5 ¾" filter pipette containing silica gel (~ 2 cm) and Celite (~2 cm). A 100µL aliquot was taken from a freshly prepared internal standard stock solution of methyl 3,5-dinitrobenzoate (22.5 mg, 0.0995 mmol) in $CDCl_3$ (1.5 mL) and was then added to the same filter pipette as the reaction aliquot. $CDCl_3$ (0.7 mL) was then used to elute the sample directly into NMR tubes. NMR analysis was used to determine the 4-nitrostyrene concentration and yields at the various timepoints, using methyl 3,5-dinitrobenzoate as the internal standard. The process was repeated for the deuterated substrate **1a-*d*$_2$**. Note: Better shimming of the samples was achieved when the NMR tubes containing the samples were allowed to sit in the freezer overnight and spectra were acquired the next day. This is attributed to any MnO_2 that was not removed by the filter pipette settling to the bottom of the tube.

Data Analysis. After Fourier transform of the FID, an auto-baseline correction protocol was applied to the spectra and the spectra were manually integrated. The integration values of the aromatic peak of the methyl 3,5-dinitrobenzoate standard (9.23 ppm, triplet) and the H_b proton of the styrene product (5.94 ppm) were used to calculate the concentration of product (**2a** or **2a-*d*$_1$**) in the reaction at each of the timepoints. The initial rates were determined from a linear fit of the early timepoints of the reactions. A KIE was determined by dividing the average initial rate of two separate runs for the *proteo*-substrate **1a** by that of the average initial rate of two separate runs for the *deutero*-substrate **1a-*d*$_2$** giving a KIE of 3.3.

Table S14. Concentration values for 4-nitrostyrene from an average of 2 trials for **1a** and **1a-*d*₂** determined from ^{1}H NMR spectra integration. This data gives rise to the reaction time course (Figure S5.) and was used to determine the initial rate of the reaction in the absence of LiOAc.

Starting Carboxylic Acid	Time (h)	[2a] or [1a-d_2] (mM) A	[2a] or [1a-d_2] (mM) B	Avg. [2a] or [1a-d_2] (mM)	Std. Dev. Of Mean	Initial Rate (mM/h)
(**1a**)	0	0	0	0	0	4.9
	0.33	1.34	0.663	1.00	0.34	
	0.67	3.34	2.98	3.16	0.18	
	1	5.34	4.64	4.99	0.35	
	1.5	8.01	7.3	7.66	0.36	
	2	10.7	8.62	9.66	1.04	
	2.5	12.7	10.6	11.7	1.05	
	3	15.7	13.9	14.8	0.90	
	4	20.7	17.9	19.3	1.40	
(**1a-d_2**)	0	0	0	0	0	1.5
	1	1.00	1.32	1.16	0.16	
	1.5	2.32	2.31	2.32	0.005	
	2	2.65	2.97	2.81	0.16	
	2.5	3.98	3.63	3.81	0.18	
	3	4.64	4.29	4.47	0.18	
	4	5.97	5.61	5.79	0.18	

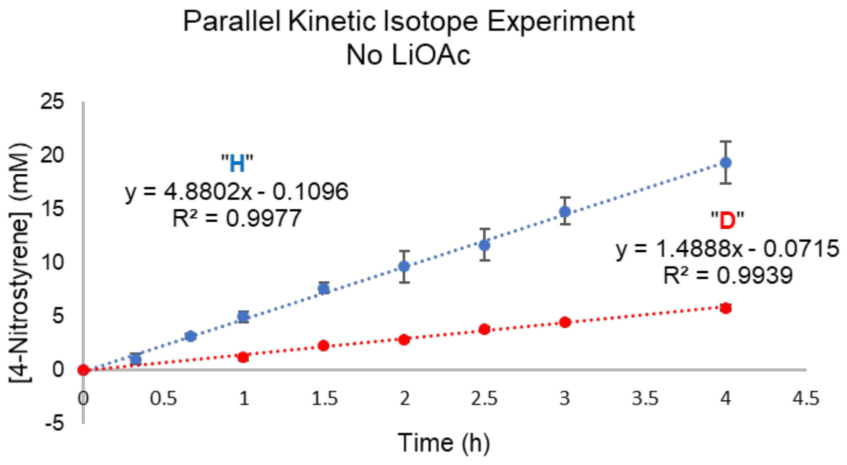

Figure S6. The blue trace (average from 2 trials) is a plot of initial rate data for the dehydrogenative decarboxylation of 4-nitrohydrocinnamic acid (**1a**) in the absence of LiOAc while the red trace (average 2 trials) is a plot of the initial rate data for the dehydrogenative decarboxylation of 4-nitrohydrocinnamic acid (**1a-*d*₂**) in the absence of LiOAc. Associated error is given by 2 times the standard deviation divided by the square root of the number of trials ((2 x stdev)/ SQRT(# trials)).

Intermolecular Competition Experiment

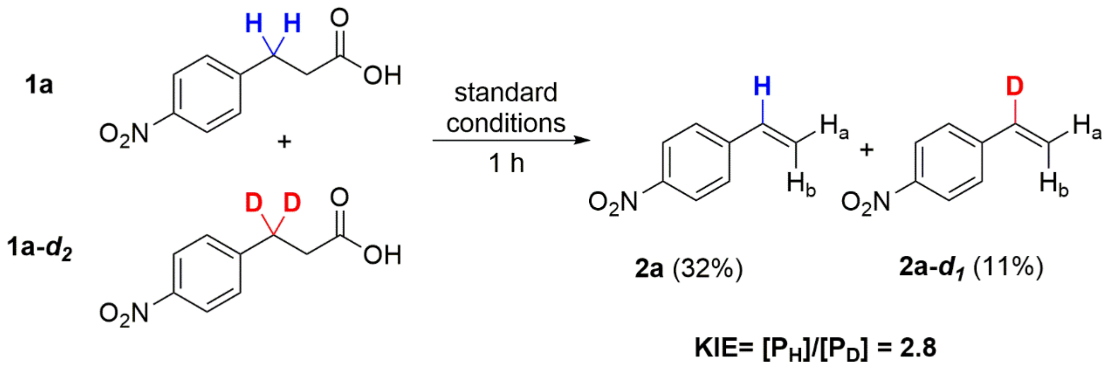

Procedure for Kinetic Isotope Effect by Intermolecular Competition Experiment. To a 4-mL dram vial fitted with a stir bar, *proteo*-4-nitrohydrocinnamic acid **1a** (9.8 mg, 0.050 mmol), *deuterio*-4-nitrohydrocinnamic acid **1a-*d₂*** (9.8 mg, 0.050 mmol), CuOAc (1.2 mg, 0.010 mmol), 2,2'-bipyridine (1.6 mg, 0.010 mmol), MnO_2 (8.7 mg, 0.10 mmol), and LiOAc (6.6 mg, 0.10 mmol) were added. The vial was transferred to a nitrogen filled glovebox where dry DMA (0.5 mL) was added, and the tube was sealed under nitrogen with a PTFE-lined cap and electrical tape. The vial was then removed from the glovebox and transferred to a pre-heated aluminum block and heated at 120 °C for 1 hour. Upon completion the reaction was cooled to room temperature. The solution was diluted with ethyl acetate (10 mL) and added to a separatory funnel along with 1 M HCl (100 mL). The layers were separated, and the aqueous layer was extracted with ethyl acetate (3 x 30 mL). The organic layers were combined, dried over Na_2SO_4 and gravity filtered into a round bottom flask. The solvent was removed under reduced pressure and a 1,3,5-trimethoxybenzene (3.0 - 3.5 mg) NMR standard was added to the sample for ^{1}H NMR analysis. A ^{1}H NMR spectrum of the crude residue was obtained in 0.7 mL $CDCl_3$ and 3 drops DMSO-d_6 and the yield was obtained by integration. After an average of 3 trials, the reaction was found to give the corresponding product mixture of **2a** and **2a-d_1** in 32% and 11% yields respectively (as determined by ^{1}H NMR spectroscopy), giving a KIE of 2.8.

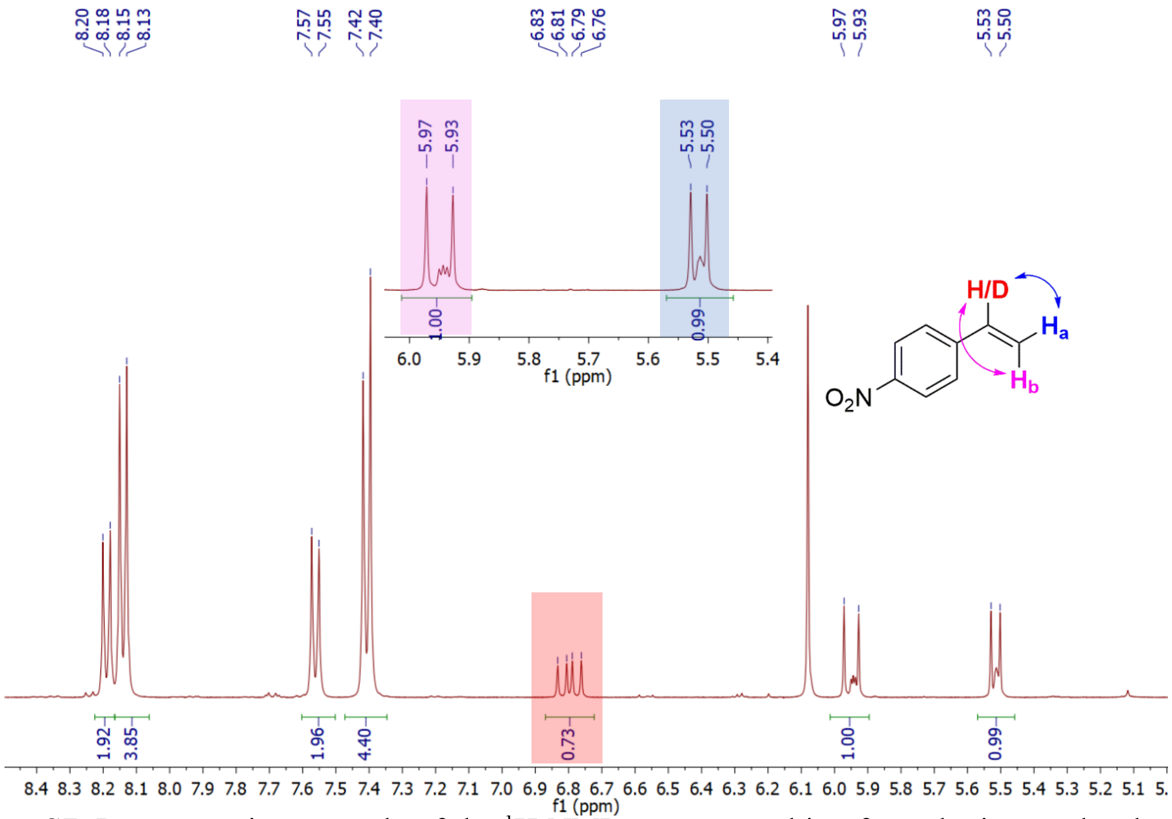

Figure S7. Representative example of the ^{1}H NMR spectra resulting from the intermolecular competition reactions of **1a** and **1a-*d*$_2$**

C. Hammett Competition Experiments

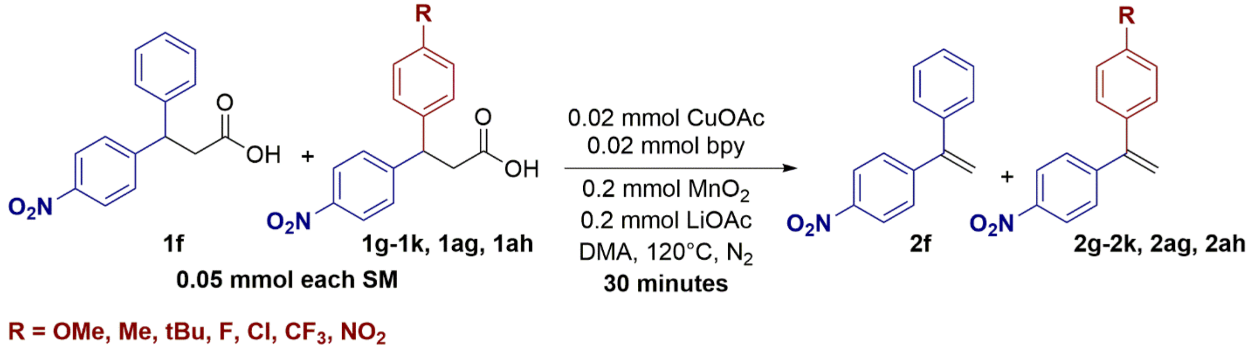

Procedure for Hammett Competition Experiments. To a 4-mL dram vial fitted with a stir bar, 3-(4-nitrophenyl)-3-phenylpropionic acid **1f** (13.6 mg, 0.0500 mmol), 3-(4-nitrophenyl)-3-(4-“R”-phenyl)propionic acid **1g-1k, 1ag, 1ah** (14.3 – 17.0 mg, 0.0500 mmol), CuOAc (2.5 mg, 0.020 mmol), 2,2'-bipyridine (3.1 mg, 0.020 mmol), MnO_2 (17.6 mg, 0.200 mmol), and LiOAc (13.2 mg, 0.200 mmol) were added. The vial was transferred to a nitrogen filled glovebox where dry DMA (1 mL) was added, and the tube was sealed under nitrogen with a PTFE-lined cap and electrical tape. The vial was then removed from the glovebox and transferred to a pre-heated aluminum block and heated at 120 °C for 30 minutes. Upon completion the reaction was cooled to room

temperature. The solution was diluted with ethyl acetate (10 mL) and added to a separatory funnel along with 1 M HCl (100 mL). The layers were separated, and the aqueous layer was extracted with ethyl acetate (3 x 20 mL). The organic layers were combined, dried over Na_2SO_4 and gravity filtered into a round bottom flask. The solvent was removed under reduced pressure and a 1,3,5-trimethoxybenzene (5.0 mg, 0.030 mmol) NMR standard was added to the sample for ^{1}H NMR analysis. A crude NMR spectrum of the residue was obtained in 0.7 mL $CDCl_3$ and 3 drops DMSO-d_6.

Data Analysis. ^{1}H NMR yield of each product (**2f-2k, 2ag, 2ah**) was obtained by integration of the aromatic peak of the 1,3,5-trimethoxybenzene internal standard (6.08 ppm) and the product resonances after the crude reaction workup. After the yields of each product were obtained by NMR spectroscopy, the ratios of substituted product (P_R, **2g-2k, 2ag, 2ah**) to unsubstituted product (P_H, **2f**) were determined (P_R/P_H) and the logarithm of these value was calculated for use in the Hammett plot ($\log(P_R/P_H)$). The data was fit using the substituent constants, σ_p^-.[4]

Table S15. Data for Hammett competition experiments

Entry	Substitution (R)	Average P_R/P_H	Average $\log(P_R/P_H)$	Std. Dev. Of Mean ($\log(P_R/P_H)$)	σ_p^-	σ_p
1	OMe	0.84	-0.076	0.0070	-0.26	-0.27
2	Me	0.93	-0.031	0.0098	-0.17	-0.17
3	*t*Bu	0.93	-0.031	0.0022	-0.13	-0.20
4	F	1.17	0.067	0.0042	-0.03	0.06
5	Cl	1.22	0.086	0.0078	0.19	0.23
6	CF_3	1.59	0.20	0.0051	0.65	0.54
7	NO_2	5.31	0.72	0.024	1.27	0.78

Entries 1-6 are comprised of an average of 2 trials each, while entry 7 is taken from the average of 4 trials. When the data was fit to the modified parameter, $\boldsymbol{\sigma_p^-}$, to account for resonance effects, a good linear fit was found. When utilizing the standard Hammett parameter, $\boldsymbol{\sigma_p}$, there was a poor linear fit.

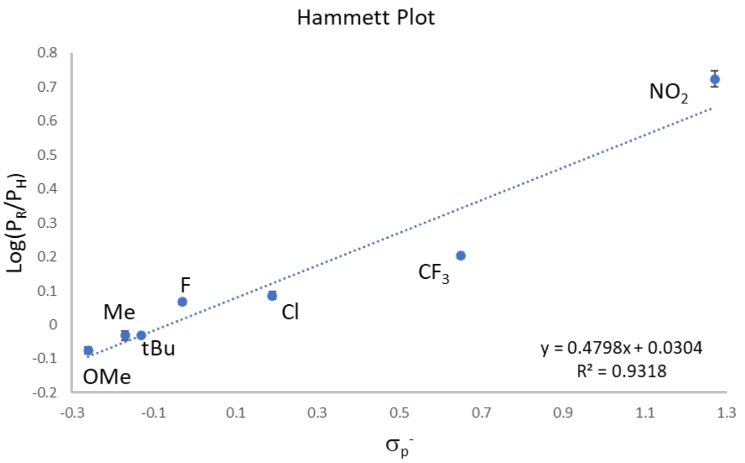

Figure S8. Hammett plot fit to $\boldsymbol{\sigma_p^-}$. Associated error is given by 2 times the standard deviation divided by the square root of the number of trials ((2 x stdev)/ SQRT(# trials)).

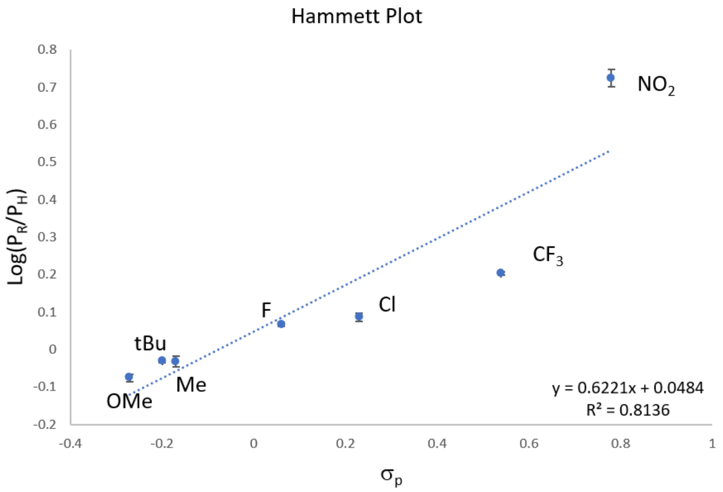

Figure S9. Hammett plot fit to $\boldsymbol{\sigma_p}$. Associated error is given by 2 times the standard deviation divided by the square root of the number of trials ((2 x stdev)/ SQRT(# trials)).

D. Radical Trapping Experiments

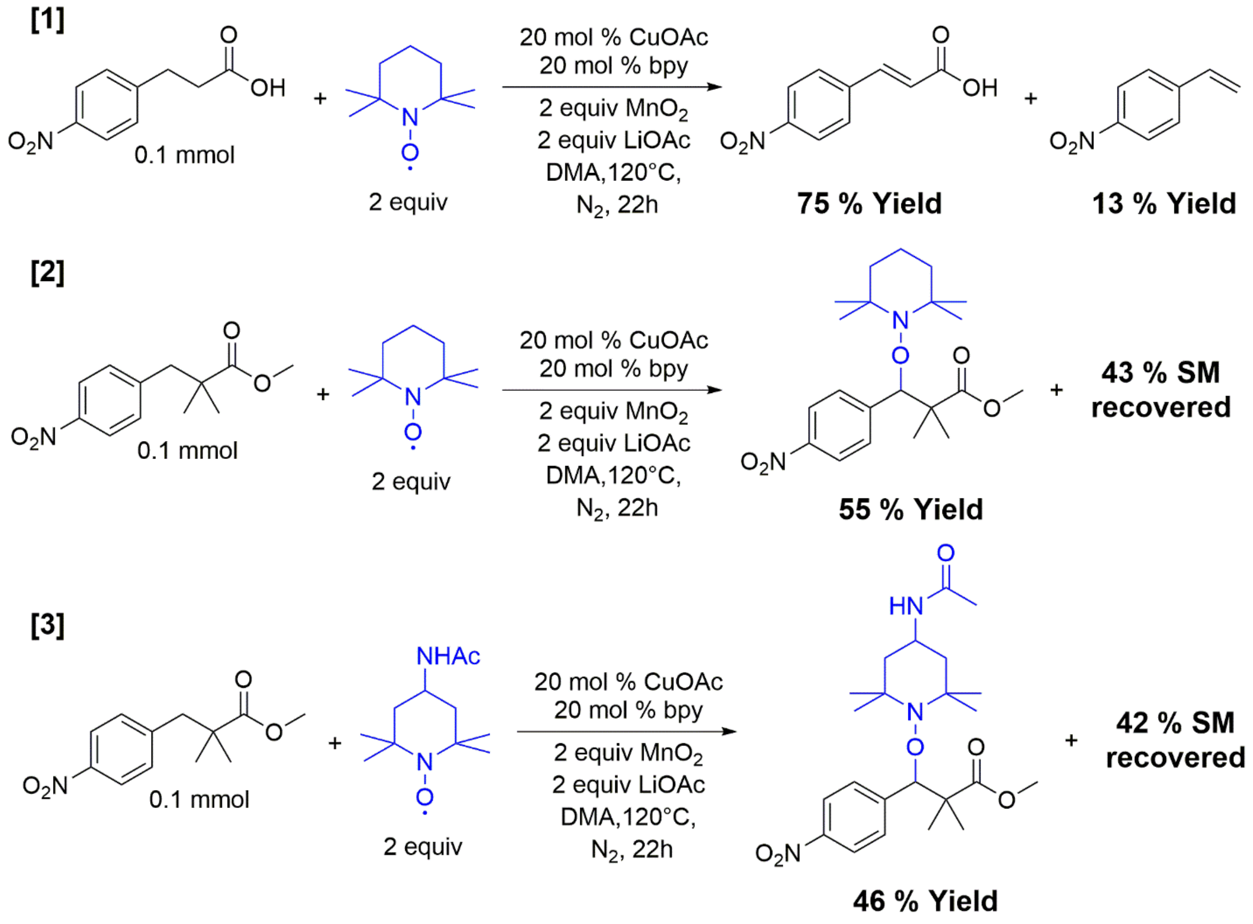

Procedure for Radical Trapping Experiments. To an oven-dried 25-mL Schlenk flask fit with a stir bar, 4-nitrohydrocinnamic acid **1a** (19.5 mg, 0.100 mmol) or 2,2-dimethyl-3-(4-nitrophenyl)propanoic acid methyl ester **5** (23.9 mg, 0.100 mmol), TEMPO (31.3 mg, 0.200 mmol) or 4-acetamido-TEMPO (42.7 mg, 0.200 mmol), CuOAc (2.5 mg, 0.020 mmol), 2,2'-bipyridine (3.1 mg, 0.020 mmol), MnO_2 (17.6 mg, 0.200 mmol), and LiOAc (13.2 mg, 0.200 mmol) were added and the tube was sealed with a septum and electrical tape. The flask was evacuated and backfilled 3 times with N_2 and DMA (1 mL) was added via syringe. The flask was then added to a pre-heated oil bath and stirred for 22 h at 120 °C. Upon completion, the reaction vessel was cooled to room temperature. The solution was diluted with ethyl acetate (15 mL) and added to a separatory funnel along with DI water (300 mL) and extracted with ethyl acetate (3 x 25 mL). The combined organic layers were washed with brine (~200 mL), dried over Na_2SO_4 and gravity filtered into a round bottom flask. The solvent was removed under reduced pressure, a methyl 3,5-dinitrobenzoate (5.0 mg, 0.022 mmol) internal standard was added, and a ^1H NMR spectrum of the crude residue was obtained in $CDCl_3$. The product yield was determined by ^1H NMR spectroscopy. For reaction **[2]** and **[3]** the crude reaction mixture was purified via silica gel column chromatography (gradient elution, hexanes: ethyl acetate (5:1, v/v) to 100 % ethyl acetate). (Note: The starting material and internal standard (methyl 3,5-dinitrobenzoate) are removed from the

column using (5:1) hexanes: ethyl acetate. The desired product does not move in this solvent system. The TEMPO trapped products can then be flushed off the column as an orange band using 100 % ethyl acetate.) For full characterization, the product from reaction **[3]** was carried forward, given the broadness observed in the ^{1}H NMR spectrum for the product from **[2]**.

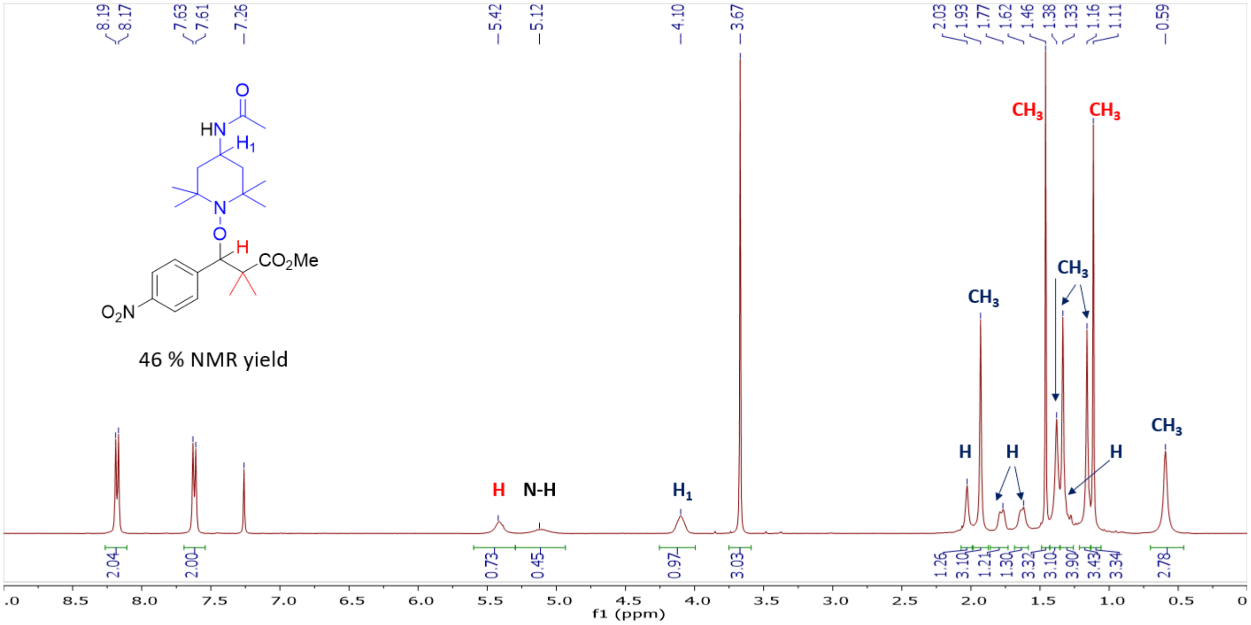

Figure S10. ^{1}H NMR spectrum of the trapped 4-acetamido-TEMPO adduct isolated by column chromatography from reaction **[3]**.

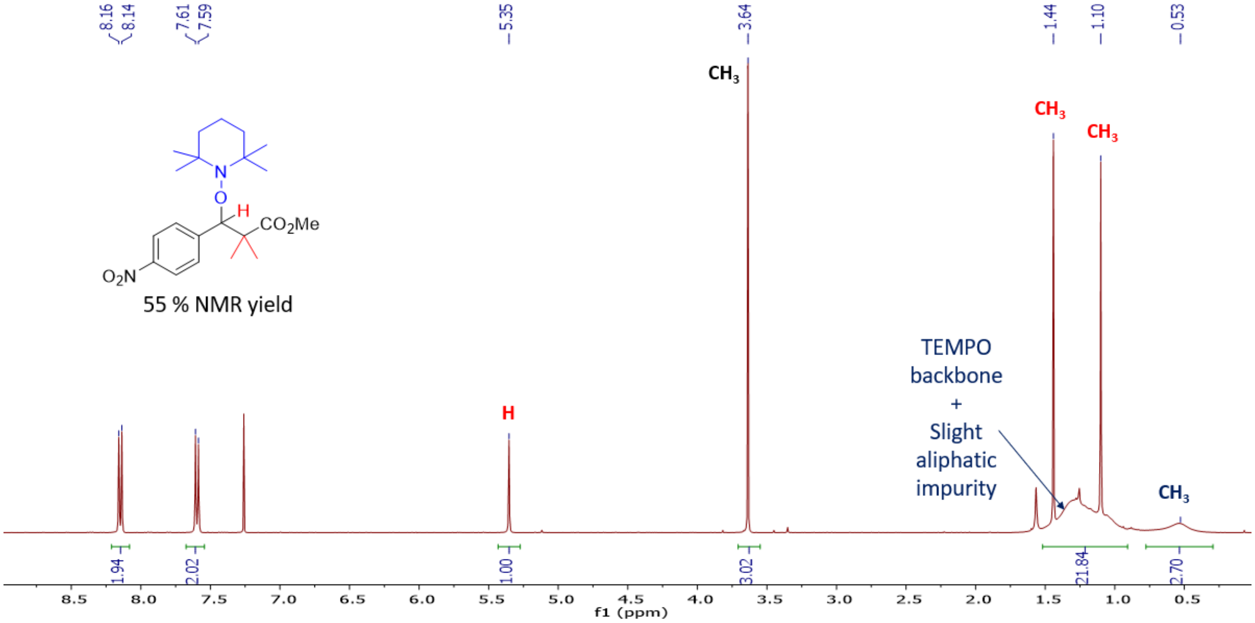

Figure S11. ^{1}H NMR spectrum of the trapped TEMPO adduct isolated by column chromatography from reaction **[2]**.

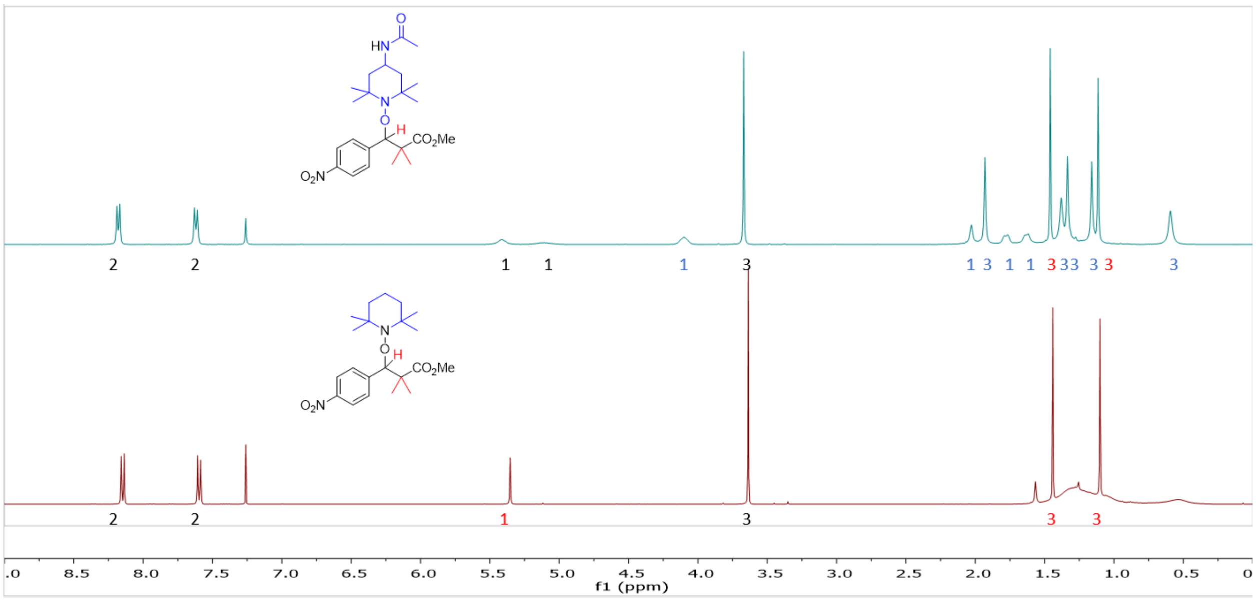

Figure S12. Overlay of ^{1}H NMR spectra of trapped TEMPO adducts from reaction **[2]** and **[3]**.

E. Initial Rate Experiments

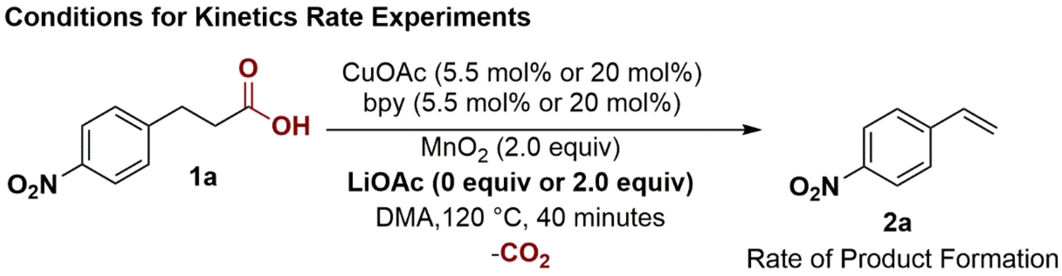

Procedure for Initial Rate Experiment. To a 25-mL Schlenk flask fitted with a stir bar, 4-nitrohydrocinnamic acid **1a** (29.3 mg, 0.150 mmol), CuOAc (1.1 mg (0083 mmol) or 3.8 mg (0.030 mmol)), 2,2'-bipyridine (1.3 mg (0.0083 mmol) or 4.8 mg (0.030 mmol)), LiOAc (0 mg or 19.8 mg, 0.300 mmol), and MnO_2 (26.2 mg, 0.300 mmol) were added. The tube was evacuated and backfilled 3 times with nitrogen and dry DMA (1.5 mL) was added via syringe. The tube was placed in a preheated oil bath and stirred at 800 RPM. Aliquots (100 µL) were taken from the reaction at designated timepoints (6, 9, 12, 15, 18, 21, 27, 33 and 40 minutes) and transferred to a 5 ¾" filter pipette containing silica gel (~ 2 cm) and Celite (~2 cm). A 100 µL aliquot was taken from a freshly prepared internal standard stock solution of methyl 3,5-dinitrobenzoate (22.5 mg, 0.0995 mmol) in $CDCl_3$ (1.5 mL) and was then added to the same filter pipette as the reaction aliquot. $CDCl_3$ (0.7 mL) was then used to elute the sample directly into NMR tubes. NMR analysis was used to determine the 4-nitrostyrene concentration and yields at the various timepoints, using methyl 3,5-dinitrobenzoate as the internal standard. Note: Better shimming of the samples was achieved when the NMR tubes containing the samples were allowed to sit in the freezer overnight and spectra were acquired the next day. This is attributed to any MnO_2 that was not removed by the filter pipette settling to the bottom of the tube.

Table S16. Concentration values for 4-nitrostyrene from multiple trials determined from ^{1}H NMR spectra integration. This data gives rise to the reaction time course (Figure S13) and was used to determine the initial rate of the reaction in presence and absence of LiOAc.

Starting Conditions	Time (h)	[2a] (mM) A	[2a] (mM) B	[2a] (mM) C	Avg. [2a] (mM)	Std. Dev. Of Mean	Initial Rate (mM/h)
20 mol% Cu/bpy 2 equiv LiOAc	0	0	0	0	0	0	123
	0.10	9.4	10.1	10.2	9.4	1.2	
	0.15	16.5	16.1	16.2	16.5	0.53	
	0.20	23.7	24.2	23.7	23.7	0.54	
	0.25	30.3	30.9	29.8	30.3	0.58	
5.5 mol% Cu/bpy 2 equiv LiOAc	0	0	0	-	0	0	72
	0.10	6.0	4.8	-	5.4	1.2	
	0.15	10.0	8.4	-	9.2	1.6	
	0.2	14.6	13.1	-	13.8	1.5	
	0.25	17.3	17.2	-	17.2	0.06	
	0.30	22.6	19.9	-	21.3	2.6	
20 mol % Cu/bpy 0 equiv LiOAc	0	0	0	0	0	0	5
	0.25	0.7	0	0.67	0.45	0.4	
	0.3	1.3	1.4	1.4	1.34	0.006	
	0.35	1.3	1.4	1.4	1.34	0.006	
	0.45	2.0	2.0	2.0	2.0	0.01	
	0.55	2.7	2.7	2.7	2.7	0.01	
	0.67	3.4	2.7	3.0	3.0	0.3	
5.5 mol% Cu/bpy 0 equiv LiOAc	0	0	-	-	0	0	0
	0.67	0	-	-	0	0	

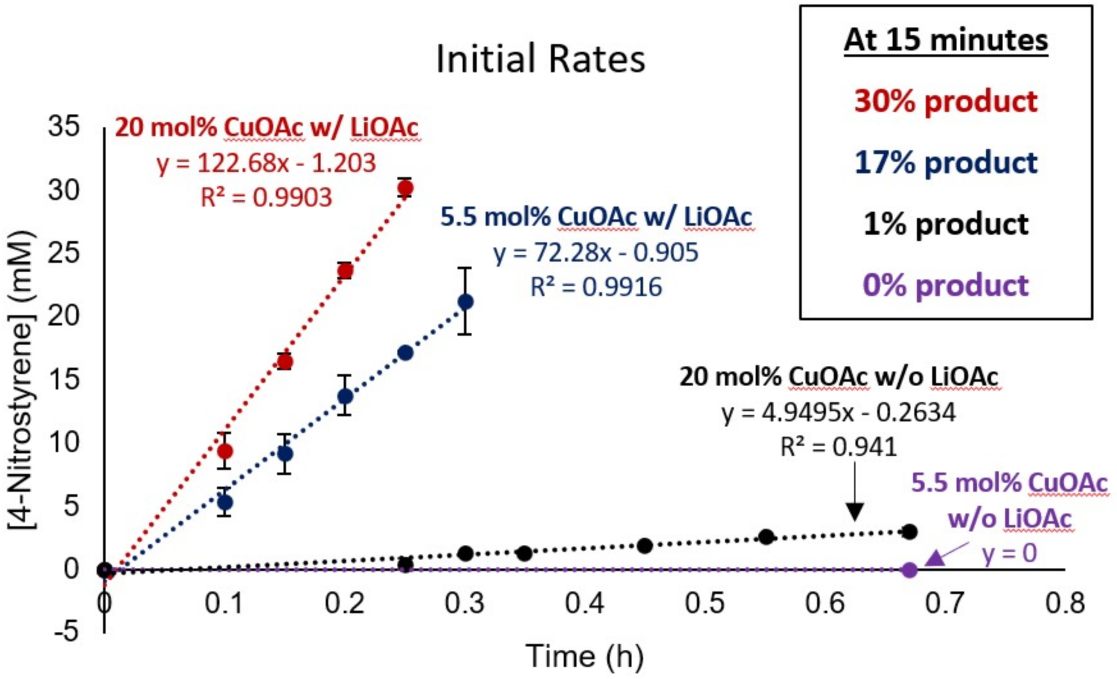

Figure S13. The red trace (average from 3 trials) is a plot of initial rate data for the dehydrogenative decarboxylation of 4-nitrohydrocinnamic acid (**1a**) using 20 mol% CuOAc and bpy in the presence of 2 equivalents of LiOAc (123 mM h^{-1}). The dark blue trace (average from 2 trials) is a plot of initial rate data for the dehydrogenative decarboxylation of 4-nitrohydrocinnamic acid (**1a**) using 5.5 mol% CuOAc and bpy in the presence of 2 equivalents of LiOAc (72 mM h^{-1}). The black trace (average from 3 trials) is a plot of initial rate data for the dehydrogenative decarboxylation of 4-nitrohydrocinnamic acid (**1a**) using 20 mol% CuOAc and bpy in the absence of LiOAc (5 mM h^{-1}). The purple trace (1 trial) is a plot of initial rate data for the dehydrogenative decarboxylation of 4-nitrohydrocinnamic acid (**1a**) using 5.5 mol% CuOAc and bpy in the absence of LiOAc (0 mM h^{-1}). Associated error is given by 2 times the standard deviation divided by the square root of the number of trials ((2 x stdev)/ SQRT(# trials)).

VII. Synthesis of Substrates Arylated at the Benzylic Position

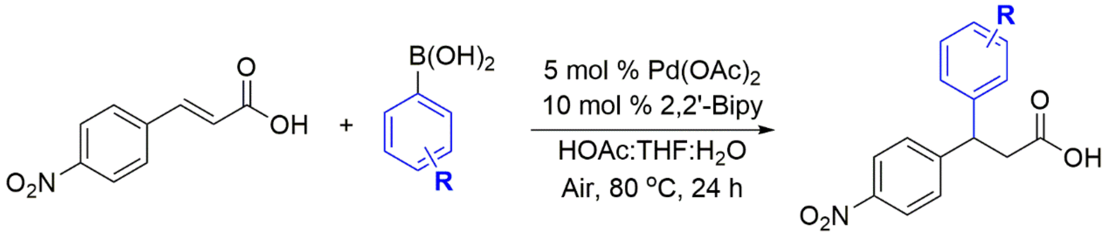

Conjugate Addition of Aryl Boronic Acids to 4-Nitrocinnamic Acid. The title compounds were synthesized according to a modified literature procedure.[5] A 50-mL Schlenk tube fit with a stir bar was charged with 4-nitrocinnamic acid (966 mg, 5.00 mmol), arylboronic acid (1.22 g, 10.0 mmol, 2 equiv), $Pd(OAc)_2$ (56.1 mg, 0.250 mmol), 2,2'-bipyiridine (78.1 mg, 0.500 mmol), HOAc (5

mL), THF (10 mL), and H_2O (3 mL) and fit with a water condenser. The tube was transferred to a pre-heated oil bath and stirred at 80 °C for 24-72 h while monitoring by TLC. Upon completion, the reaction was cooled to room temperature. The solution was diluted with ethyl acetate (20 mL) and added to a separatory funnel along with brine (400 mL). The layers were separated, and the aqueous layer was extracted with ethyl acetate (3 x 30 mL). The combined organic layers were dried over Na_2SO_4, filtered, and the solvent was removed under reduced pressure to yield the crude product. Two different purification methods were used to isolate the acid products cleanly. In most cases isolation procedure A leads to more efficient isolations compared to procedure B.

Procedure A: Esterification of Conjugate Addition Products. To ease in the separation of the crude reaction mixture, the products were converted to methyl esters prior to isolation. A 250-mL round bottom flask fit with a stir bar was charged with the crude product mixture (1.50 g), K_2CO_3 (3 equiv), MeI (3 equiv), and anhydrous DMF (10 mL). The reaction was stirred at room temperature overnight. After completion, brine (400 mL) was added and the layers were separated. The resulting aqueous layer was extracted with ethyl acetate (3 x 50 mL). The organic layers were combined and dried over Na_2SO_4, filtered, and the solvent was removed under reduced pressure. The residue was purified via silica gel column chromatography (mobile phase = hexanes : ethyl acetate (15:1 v/v to 8:1, v/v)) to afford the corresponding methyl ester of 3-(4-nitrophenyl)-3-phenylpropanoic acid. This product was carried to the next step.

Hydrolysis of Methyl Esters of 3-(4-Nitrophenyl)-3-Phenylpropanoic Acids. A 100 mL round bottom flask was charged with the methyl 3-(4-nitrophenyl)-3-phenylpropionate (1.9 mmol), THF/EtOH/H_2O (12 mL, 1:1:1 by volume), and KOH (4 equiv). The reaction mixture was allowed to stir overnight (~12 h). The reaction was acidified with 2 M HCl until a pH of 2 was achieved as determined by pH paper. The acidification was accompanied by formation of a precipitate. The reaction mixture was transferred to a separatory funnel and extracted with ethyl acetate (3 x 50 mL), dried over anhydrous Na_2SO_4, and filtered. The solvent was removed under vacuum, furnishing the desired 3-(4-nitrophenyl)-3-phenylpropanoic acid. Most acid products could be used without further purification. If necessary, further purification by silica gel column chromatography was utilized (dichloromethane: methanol (98:2, v/v) to (95:5, v/v) gradient elution).

Procedure B: Basic Extraction of Crude Carboxylic Acid Reaction Mixture. After the initial aqueous extraction, the crude reaction mixture was diluted with dichloromethane (20 mL) and transferred to separatory funnel along with 2 M aqueous NaOH (20 mL). The layers were separated and the organic layer was extracted with 2 M NaOH (3 x 30 mL). The aqueous layers were then combined and washed with dichloromethane (3 x 15 mL). Following the dichloromethane wash, the aqueous layer was acidified with concentrated HCl until a pH ~ 2 was reached, as determined by pH paper. The acidification was accompanied by precipitation of the product as an off-white to yellow solid. The solid was filtered, washed with DI water (200 mL) and dried under vacuum. The solid was further purified via silica gel column chromatography (gradient elution, dichloromethane: methanol (98:2, v/v) to (95:5, v/v)). If necessary, the product was recrystallized from chloroform or a mixed solvent system of chloroform/methanol to afford the desired carboxylic acid product.

VIII. Characterization of Hydrocinnamic Acid Starting Materials (1)

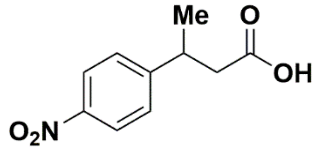

3-(4-Nitrophenyl)butanoic acid (1d). The title compound was obtained in 35% yield (1.69 g, 8.08 mmol) as a light beige solid by nitration of 3-phenylbutanoic acid (3.76 g, 22.9 mmol) following literature procedure.[6] Purification by silica gel column chromatography (dichloromethane: methanol (98:2, v/v)). ^{1}H NMR (400 MHz, DMSO-d_6): δ = 12.14 (s, 1H), 8.15 (d, *J* = 8.8 Hz, 2H), 7.56 (d, *J* = 8.8 Hz, 1H), 3.34 – 3.25 (m, 1H), 2.59 (d, *J* = 7.6 Hz, 2H), 1.24 (d, *J* = 6.8 Hz, 3H). ^{13}C{^{1}H} NMR (100 MHz, DMSO-d_6): δ = 172.8, 154.2, 146.0, 128.2, 123.4, 41.3, 35.8, 21.6. The spectral data are consistent with those reported in the literature.[6]

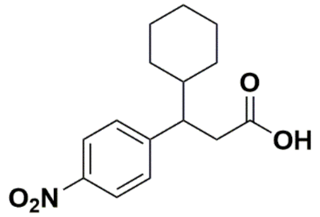

3-Cyclohexyl-3-(4-nitrophenyl)propanoic acid (1e). The title compound was synthesized in 82% crude yield by nitration of β-cyclohexylbenzenepropanoic acid (1.40 g, 6.03 mmol) following literature procedure.[6] Following Procedure A in section VII, **1e** (1.37 g, 4.94 mmol) was then esterified with MeI (922 µL, 13.0 mmol) furnishing the methyl ester product in 76% yield. The ester product was purified via silica gel column chromatography (hexanes to hexanes: ethyl acetate (30:1 v/v) gradient elution). Finally, the methyl ester (1.10 g, 3.78 mmol) was hydrolyzed to the acid, furnishing the title compound **1e** in 32% overall yield (539 mg, 1.94 mmol) as a white solid. mp = 112 –113 °C. ^{1}H NMR (400 MHz, $CDCl_3$): 8.14 (d, *J* = 8.8 Hz, 2H), 7.29 (d, *J* = 8.8 Hz, 2H), 3.02 – 2.96 (m, 1H), 2.88 (dd, *J* = 16.0, 4.0 Hz, 1H), 2.60 (dd, *J* = 16.0, 10.4 Hz, 1H), 1.82 – 1.73 (m, 2H), 1.68 – 1.57 (m, 2H), 1.54 – 1.45 (m, 1H), 1.38– 1.35 (m, 1H), 1.26 – 1.15 (m, 1H), 1.13 – 1.03 (m, 2H), 0.98 – 0.88 (m, 1H), 0.84 – 0.73 (m, 1H). ^{13}C{^{1}H} NMR (150 MHz, $CDCl_3$): δ = 178.4, 150.9, 146.8, 129.2, 123.6, 47.8, 42.8, 37.6, 30.9, 30.8, 26.4, 26.29, 26.28. FTIR (ATR, cm^{-1}): 2926, 2856, 1694, 1515, 1314, 1109, 856. HRMS (ESI-MS) *m/z* calcd for $C_{15}H_{20}NO_4$ $[M + H]^+$ 278.1387 found 278.1382.

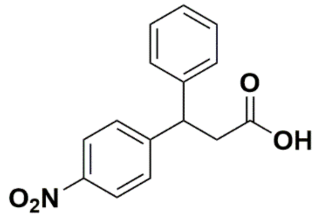

3-(4-Nitrophenyl)-3-phenylpropanoic acid (1f). The title compound was synthesized in 70% crude yield (1.15 g, 4.24 mmol) from 4-nitrocinnamic acid (1.17 g, 6.06 mmol) and phenylboronic acid (1.47 g, 12.1 mmol) following the literature procedure outlined in section VIII. Following

Procedure A, **1f** (1.15 g, 4.24 mmol) was then esterified with MeI (791 µL, 12.7 mmol) furnishing the methyl ester product in 85% yield (1.03 g, 3.61 mmol). The ester product was purified via silica gel column chromatography (hexanes: ethyl acetate (10:1 v/v)). Finally, a portion of the methyl ester product (536.6 mg, 1.881 mmol) was hydrolyzed to the acid, furnishing the title compound **1f** in 56% overall yield (486.3, 1.793 mmol) as an off-white solid.[5] ^{1}H NMR (400 MHz, $CDCl_3$): δ = 8.14 (d, *J* = 8.4 Hz, 2H), 7.40 (d, *J* = 8.4 Hz, 2H), 7.33 – 7.30 (m, 2H), 7.26 – 7.19 (m, 3H), 4.62 (t, *J* = 8.0 Hz, 1H), 3.13 (d, *J* = 8.0 Hz, 2H). ^{13}C{^{1}H} NMR (150 MHz, $CDCl_3$): δ = 176.9, 150.8, 146.9, 141.7, 129.2, 128.7, 127.7, 127.5, 124.1, 46.6, 40.0. The spectral data are consistent with those reported in the literature.[5]

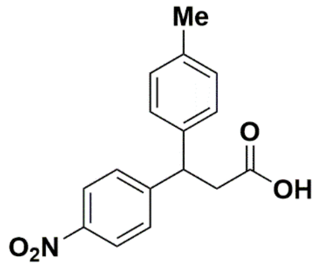

3-(4-Methylphenyl)-3-(4-nitrophenyl)propanoic acid (1g). The title compound was synthesized in 71% crude yield (1.21 g, 4.26 mmol) from 4-nitrocinnamic acid (1.17 g, 6.06 mmol) and 4-methylphenylboronic acid (1.64 g, 12.1 mmol) following the procedure outlined in section VIII.[5] Following Procedure A, **1g** (1.21 g, 4.26 mmol) was then esterified with MeI (800 µL, 12.8 mmol) furnishing the methyl ester product in 78% yield (990.3 mg, 3.309 mmol). The ester product was purified via silica gel column chromatography (hexanes: ethyl acetate (10:1 v/v)). Finally, a portion of the methyl ester product (511.7 mg, 1.709 mmol) was hydrolyzed to the acid, furnishing the title compound **1g** in 54 % overall yield (487.7 mg, 1.653 mmol) as an off-white solid. mp = 158 – 160 °C. ^{1}H NMR (400 MHz, $CDCl_3$): 8.13 (d, *J* = 8.8 Hz, 2H), 7.39 (d, *J* = 8.8 Hz, 2H), 7.13 – 7.07 (m, 4H), 4.58 (t, *J* = 8.0 Hz, 1H), 3.16 – 3.05 (m, 2H), 2.31 (s, 3H). ^{13}C{^{1}H} NMR (150 MHz, $CDCl_3$): δ = 176.9, 151.1, 146.9, 138.7, 137.2, 129.9, 128.7, 127.5, 124.1, 46.3, 40.0, 21.2. FTIR (ATR, cm^{-1}): 1705, 1596, 1595, 1522, 1418, 1342, 1115, 855, 800. HRMS (ESI-MS) *m/z* calcd for $C_{16}H_{14}NO_4$ $[M - H]^-$ 284.0928 found 284.0933.

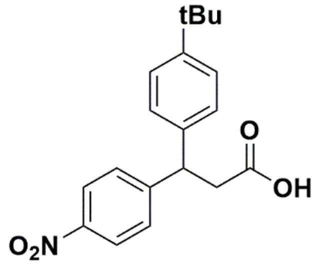

3-(4-Nitrophenyl)-3-(4-*tert*-butylphenyl)propanoic acid (1h). The title compound was synthesized in 42% yield (824.9 mg, 2.520 mmol) as an off-white solid from 4-nitrocinnamic acid (1.16 g, 6.00 mmol) and 4-*tert*-butylphenylboronic acid (2.14 g, 12.0 mmol) following the procedure outlined in section VIII, Procedure B.[5] Purification by silica gel column chromatography (dichloromethane: methanol (98:2, v/v) to (95:5, v/v) gradient elution). mp = 164 – 165 °C. ^{1}H NMR (400 MHz, DMSO-d_6): δ = 12.22 (s, 1H), 8.13 (d, *J* = 8.8 Hz, 2H), 7.63 (d, *J* = 8.8 Hz, 2H), 7.31 – 7.26 (m, 4H), 4.55 (t, *J* = 8.0 Hz, 1H), 3.16 – 3.03 (m, 2H), 1.22 (s, 9H). ^{13}C{^{1}H} NMR

(150 MHz, $CDCl_3$): δ = 177.1, 151.1, 150.4, 146.9, 138.6, 128.8, 127.3, 126.1, 124.1, 46.2, 40.0, 34.6, 31.5. FTIR (ATR, cm^{-1}): 2966, 2908, 2869, 1712, 1607, 1594, 1511, 1342, 1259, 1110, 856. HRMS (ESI-MS) *m/z* calcd for $C_{19}H_{20}NO_4$ $[M - H]^-$ 326.1398 found 326.1402.

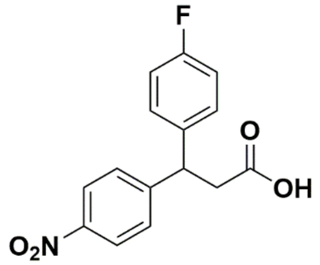

3-(4-Fluorophenyl)-3-(4-nitrophenyl)propanoic acid (1i). The title compound was synthesized in 88% crude yield (1.55 g, 5.36 mmol) from 4-nitrocinnamic acid (1.16 g, 6.00 mmol) and 4-fluorophenylboronic acid (1.67 g, 11.9 mmol) following the literature procedure outlined in section VIII.[5] Following Procedure A, **1i** (1.55 g, 5.34 mmol) was then esterified with MeI (995 μL, 16.0 mmol) furnishing the methyl ester product in 71% yield (1.16 g, 3.83 mmol). The ester product was purified via silica gel column chromatography (hexanes: ethyl acetate (15:1 v/v) to (10:1, v/v) gradient elution). Finally, a portion of the methyl ester product (570.9 mg, 1.882 mmol) was hydrolyzed to the acid, furnishing the title compound **1i** in 57% overall yield (544.6 mg, 1.88 mmol) as a light tan to off-white solid. mp = 153 – 155 °C. ^1H NMR (400 MHz, $CDCl_3$): δ = 8.16 (d, J = 8.8 Hz, 2H), 7.38 (d, J = 8.8 Hz, 2H), 7.19 – 7.14 (m, 2H), 7.01 (t, J = 8.8 Hz, 2H), 4.61 (t, J = 8.0 Hz, 1H), 3.10 (d, J = 8.0 Hz, 2H).$^{13}C\{^1H\}$ NMR (150 MHz, $CDCl_3$): δ = 176.5, 162.1 (d), 150.5, 147.0, 137.4 (d), 129.3 (d), 128.6, 124.2, 116.1 (d), 45.9, 40.1. FTIR (ATR, cm^{-1}): 1706, 1607, 1599, 1508, 1420, 1343, 1228, 1160, 1110, 1098, 817. HRMS (ESI-MS) *m/z* calcd for $C_{15}H_{12}FNO_4Na$ $[M + Na]^+$ 312.0643 found 312.0641.

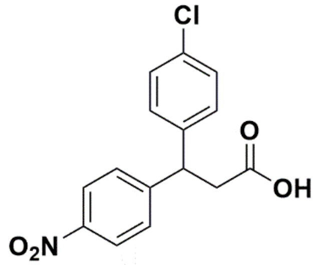

3-(4-Chlorophenyl)-3-(4-nitrophenyl)propanoic acid (1j). The title compound was synthesized in 89% crude yield (1.66 g, 5.41 mmol) from 4-nitrocinnamic acid (1.17 g, 6.06 mmol) and 4-chlorophenylboronic acid (1.80 g, 11.5 mmol) following the literature procedure outlined in section VIII.[5] Following Procedure A, **1j** (1.66 g, 5.41 mmol) was then esterified with MeI (1010 μL, 16.2 mmol) furnishing the methyl ester product in 78% yield (1.35 g, 4.22 mmol). The ester product was purified via silica gel column chromatography (hexanes: ethyl acetate (15:1 v/v) to (10:1, v/v) gradient elution). Finally, a portion of the methyl ester product (587.3 mg, 1.837 mmol) was hydrolyzed to the acid, furnishing the title compound **1j** in 65% overall yield (530.1 mg, 1.734 mmol) as an off-white solid. mp = 142 – 143 °C. ^1H NMR (400 MHz, $CDCl_3$): δ = 8.16 (d, J = 8.8 Hz, 2H), 7.37 (d, J = 8.8 Hz, 2H), 7.29 (d, J = 8.4 Hz, 2H), 7.14 (d, J = 8.4 Hz, 2H), 4.60 (t, J = 7.9 Hz, 1H), 3.10 (d, J = 8.0 Hz, 2H). $^{13}C\{^1H\}$ NMR (150 MHz, $CDCl_3$): δ = 176.4, 150.2, 147.1, 140.2, 133.5, 129.4, 129.1, 128.7, 124.2, 46.0, 39.8. FTIR (ATR, cm^{-1}): 2920, 1706, 1598, 1597,

1517, 1491, 1414, 1342, 1289, 1093. HRMS (ESI-MS) *m/z* calcd for $C_{15}H_{11}ClNO_4$ [M - H]$^-$ 304.0382 found 304.0387.

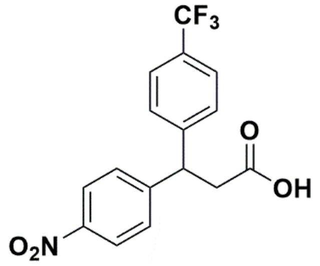

3-(4-Nitrophenyl)-3-(4-trifluoromethylphenyl)propanoic acid (1k). The title compound was synthesized in 43% yield (723.2 mg, 2.131 mmol) as a bright white solid from 4-nitrocinnamic acid (980.0 mg, 5.072 mmol) and 4-trifluoromethylphenylboronic acid (1.89 g, 10.0 mmol) following the literature procedure outlined in section VIII, Procedure B.[5] Purification by silica gel column chromatography (dichloromethane: methanol (98:2, v/v) to (95:5, v/v) gradient elution). mp = 143 – 144 °C. ^{1}H NMR (400 MHz, $CDCl_3$): δ = 8.17 (d, *J* = 8.8 Hz, 2H), 7.58 (d, *J* = 8.4 Hz, 2H), 7.39 (d, *J* = 8.8 Hz, 2H), 7.33 (d, *J* = 8.4 Hz, 2H), 4.69 (t, *J* = 8.0 Hz, 1H), 3.15 (d, *J* = 8.0 Hz, 2H). ^{13}C{^{1}H} NMR (150 MHz, $CDCl_3$): δ = 175.6, 149.6, 147.2, 145.6, 130.0 (q), 128.7, 128.2, 126.2 (q), 124.3, 124.1 (q), 46.4, 39.6. FTIR (ATR, cm^{-1}): 3045, 2855, 1705, 1598, 1515, 1347, 1326, 1270, 1163, 1110, 1067, 1017. HRMS (ESI-MS) *m/z* calcd for $C_{16}H_{11}F_3NO_4$ [M - H]$^-$ 338.0646 found 338.0651.

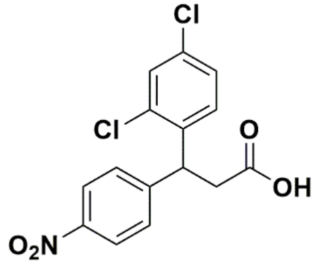

3-(2,4-Dichlorophenyl)-3-(4-nitrophenyl)propanoic acid (1l). The title compound was synthesized in 20% yield (344.2 mg, 1.012 mmol) as a white solid from 4-nitrocinnamic acid (967.1 mg, 12.00 mmol) and 2,4-dichlorophenylboronic acid (1.90 g, 10.0 mmol) following the literature procedure outlined in section VIII, Procedure B.[5] Purification by silica gel column chromatography (dichloromethane: methanol (98:2, v/v) to (95:5, v/v) gradient elution). mp = 197 – 198 °C. ^{1}H NMR (400 MHz, $CDCl_3$): δ = 8.16 (d, *J* = 8.8 Hz, 2H), 7.41 – 7.38 (m, *J* = 8.8 Hz, 3H), 7.28 – 7.25 (m, 1H), 7.18 (d, *J* = 8.4 Hz, 1H), 5.07 (t, *J* = 8.0 Hz, 1H), 3.15 – 3.05 (m, 2H). ^{13}C{^{1}H} NMR (150 MHz, $CDCl_3$ + 1 drop DMSO-d_6): δ = 172.6, 149.5, 146.8, 138.3, 134.9, 133.7, 130.1, 129.2, 129.0, 127.6, 123.9, 42.9, 39.6. FTIR (ATR, cm^{-1}): 3028, 2930, 1711, 1597, 1516, 1412, 1349, 1257, 1218, 1105, 803, 702. HRMS (ESI-MS) *m/z* calcd for $C_{15}H_{10}Cl_2NO_4$ [M - H]$^-$ 337.9992 found 337.9998.

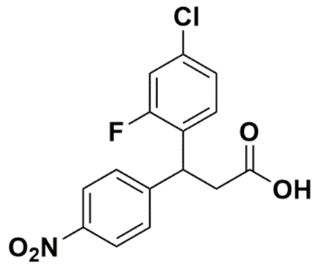

3-(2-Fluoro-4-chlorophenyl)-3-(4-nitrophenyl)propanoic acid (1m). The title compound was synthesized in 20% yield (323.6 mg, 1.00 mmol) as an off-white solid from 4-nitrocinnamic acid (980.2 mg, 5.01 mmol) and 2-fluoro-4-chlorophenylboronic acid (1.74 g, 10.0 mmol) following the literature procedure outlined in section VIII, Procedure B.[5] Purification by silica gel column chromatography (dichloromethane: methanol (98:2, v/v) to (95:5, v/v) gradient elution). mp = 143 – 145 °C. ^{1}H NMR (400 MHz, $CDCl_3$): δ = 8.16 (d, *J* = 8.8 Hz, 2H), 7.40 (d, *J* = 8.8 Hz, 2H), 7.19 – 7.06 (m, 3H), 7.18 (d, *J* = 8.4 Hz, 1H), 4.83 (t, *J* = 8.0 Hz, 1H), 3.14 (d, *J* = 7.6 Hz, 2H). ^{13}C{^{1}H} NMR (150 MHz, $CDCl_3$ + 2 drops DMSO-*d*$_6$): δ =172.7, 160.4 (d), 149.7, 146.9, 133.9 (d), 129.3 (d), 128.7, 128.1 (d), 124.9 (d), 123.9, 116.8 (d), 40.2, 38.8. FTIR (ATR, cm^{-1}): 3082, 2855, 1704, 1607, 1519, 1486, 1347, 1265, 894, 856, 695. HRMS (ESI-MS) *m/z* calcd for $C_{15}H_{11}Cl_1F_1NO_4Na$ [M + Na]$^+$ 346.0253 found 346.0250.

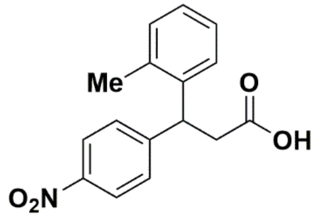

3-(2-Methylphenyl)-3-(4-nitrophenyl)propanoic acid (1n). The title compound was synthesized in 14% crude yield (236.2 mg, 0.8279 mmol) as a white solid from 4-nitrocinnamic acid (1.16 g, 6.00 mmol) and 2-methylphenylboronic acid (1.63 g, 12.0 mmol) following the literature procedure outlined in section VIII.[5] Following Procedure A, **1n** (236.2 g, 0.8279 mmol) was then esterified with MeI (155 μL, 2.48 mmol) furnishing the methyl ester product in 65% yield (160.5 mg, 0.5362 mmol). The ester product was purified via silica gel column chromatography (hexanes: ethyl acetate (10:1 v/v)). Finally, the methyl ester product (160.5 mg, 0.5362 mmol) was hydrolyzed to the acid, furnishing the title compound **1n** in 6% overall yield (107.7 mg, 0.3775 mmol). mp = 121 – 122 °C. ^{1}H NMR (400 MHz, $CDCl_3$): δ = 8.12 (d, *J* = 8.8 Hz, 2H), 7.36 (d, *J* = 8.8 Hz, 2H), 7.24 – 7.14 (m, 4H), 4.83 (t, *J* = 8.0 Hz, 1H), 3.15 – 3.03 (m, 2H), 2.27 (s, 3H). ^{13}C{^{1}H} NMR (150 MHz, $CDCl_3$): δ = 177.0, 150.6, 146.9, 139.6, 136.4, 131.3, 129.0, 127.5, 126.8, 126.3, 124.0, 42.7, 40.3, 19.9. FTIR (ATR, cm^{-1}): 1710, 1607, 1596, 1515, 1345, 1261, 1110, 854, 738. HRMS (ESI-MS) *m/z* calcd for $C_{16}H_{14}NO_4$ [M - H]$^-$ 284.0928 found 284.0932.

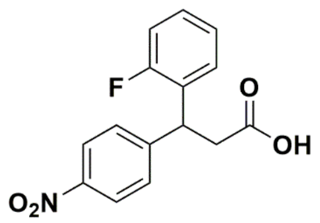

3-(2-Fluorophenyl)-3-(4-nitrophenyl)propanoic acid (1o). The title compound was synthesized in 57% crude yield (985.4 mg, 3.407 mmol) from 4-nitrocinnamic acid (1.16 g, 6.00 mmol) and 2-fluorophenylboronic acid (1.68 g, 12.0 mmol) following the literature procedure outlined in section VIII.[5] Following Procedure A, **1o** (985.4 g, 3.407 mmol) was then esterified with MeI (635 μL, 10.2 mmol) furnishing the methyl ester product in 82% yield (844.8 mg, 2.79 mmol). The ester product was purified via silica gel column chromatography (hexanes: ethyl acetate (10:1 v/v)). Finally, a portion of the methyl ester product (451.5 mg, 1.489 mmol) was hydrolyzed to the acid, furnishing the title compound **1o** in 44% overall yield (407.2 mg, 1.408 mmol) as a light-yellow solid. mp = 125 – 126 °C. ^{1}H NMR (400 MHz, $CDCl_3$): δ = 8.15 (d, *J* = 8.4 Hz, 2H), 7.43 (d, *J* = 8.4 Hz, 2H), 7.28 – 7.21 (m, 3H), 7.15 – 7.11 (m, 1H), 7.05 – 7.01 (m, 1H), 4.88 (t, *J* = 8.0 Hz, 1H), 3.16 (d, *J* = 8.0 Hz, 2H) ^{13}C{^{1}H} NMR (150 MHz, $CDCl_3$ + 1 drops DMSO-d_6): δ = 173.1, 160.5 (d), 150.3, 146.7, 129.3 (d), 129.0 (d), 128.8, 128.5 (d), 124.6 (d), 123.8, 116.0 (d), 40.5, 38.9. FTIR (ATR, cm^{-1}) 1699, 1609, 1597, 1521, 1487, 1343, 1232, 1103, 856, 745. HRMS (ESI-MS) *m/z* calcd for $C_{15}H_{11}FNO_4$ [M - H]$^-$ 288.0678 found 288.0682.

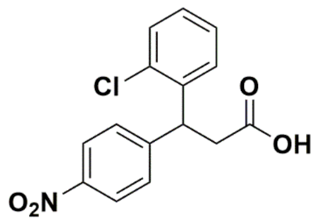

3-(2-Chlorophenyl)-3-(4-nitrophenyl)propanoic acid (1p). The title compound was synthesized in 29% yield (354.5 mg, 1.160 mmol) as a white solid from 4-nitrocinnamic acid (780.1 mg, 4.038 mmol) and 2-chlorophenylboronic acid (1.25 g, 10.0 mmol) following the literature procedure outlined in section VIII, Procedure B.[5] Purification by silica gel column chromatography (dichloromethane: methanol (98:2, v/v) to (95:5, v/v) gradient elution). mp = 172 – 173 °C. ^{1}H NMR (600 MHz, $CDCl_3$ + 1 drop DMSO-d_6): δ = 8.10 (d, *J* = 8.4 Hz, 2H), 7.41 (d, *J* = 8.4, 2H), 7.34 (d, *J* = 7.8 Hz, 1H), 7.25 – 7.23 (m, 2H), 7.20 – 7.15 (m, 1H), 5.11 (t, *J* = 9.2 Hz, 1H), 3.03 (m, 2H). ^{13}C{^{1}H} NMR (150 MHz, $CDCl_3$ + 1 drop DMSO-d_6): δ = 173.2, 150.1, 146.8, 139.6, 134.2, 130.1, 129.1, 128.5, 128.3, 127.4, 123.8, 43.3, 39.7. FTIR (ATR, cm^{-1}): 3027, 2921, 1710, 1600, 1598, 1516, 1412, 1348, 1258, 1215, 745, 702. HRMS (ESI-MS) *m/z* calcd for $C_{15}H_{11}ClNO_4$ [M - H]$^-$ 304.0382 found 304.0386.

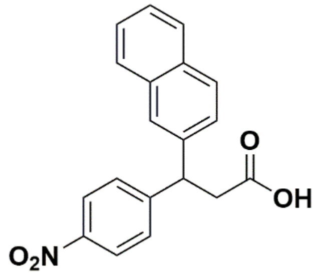

3-(4-Naphthyl)-3-(4-nitrophenyl)propanoic acid (1q). The title compound was synthesized in 39% yield (629.8 mg, 1.96 mmol) as a tan solid from 4-nitrocinnamic acid (970.1 mg, 5.00 mmol) and 2-naphthylboronic acid (1.72 g, 10.0 mmol) following the literature procedure outlined in section VIII, Procedure B.[5] Purification by silica gel column chromatography (dichloromethane: methanol (98:2, v/v) to (95:5, v/v) gradient elution). mp = 140 – 141 °C. ^{1}H NMR (600 MHz, $CDCl_3$): δ = 8.14 (d, J = 8.8 Hz, 2H), 7.81 – 7.76 (m, 3H), 7.69 (s, 1H), 7.52 – 7.43 (m, 4H), 7.27 – 7.24 (m, 2H), 4.79 (t, J = 8.0 Hz, 1H), 3.29 – 3.17 (m, 2H). ^{13}C{^{1}H} NMR (150 MHz, $CDCl_3$ + 2 drops DMSO-d_6): δ = 173.3, 151.3, 146.7, 139.7, 133.5, 132.4, 128.9, 128.7, 127.9, 127.7, 126.4, 126.2, 126.1, 126.0, 123.9, 46.9, 40.2. FTIR (ATR, cm^{-1}): 3050, 1722, 1662, 1597, 1515, 1346, 1232, 1115, 849, 814, 748. HRMS (ESI-MS) m/z calcd for $C_{19}H_{14}NO_4$ $[M - H]^-$ 320.0928 found 320.0931.

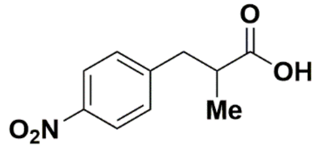

2-Methyl-3-(4-nitrophenyl)propanoic acid (1r). The title compound was obtained in 30% yield (1.47 g, 7.03 mmol) as a light yellow to off-white solid by nitration of 2-methyl-3-phenylpropanoic acid (4.24 g, 25.8 mmol) following literature procedure.[6] Purification by silica gel column chromatography (dichloromethane: methanol (98:2, v/v)). ^{1}H NMR (400 MHz, $CDCl_3$): δ = 8.16 (d, J = 8.4 Hz, 2H), 7.35 (d, J = 8.4 Hz, 2H), 3.17 – 3.13 (m, 1H), 2.84 – 2.79 (m, 2H), 1.23 (d, J = 7.2 Hz, 3H). ^{13}C{^{1}H} NMR (150 MHz $CDCl_3$): δ = 177.5, 147.7, 146.5, 129.9, 123.5, 40.6, 39.3, 17.0. The compound is known in the literature.[7]

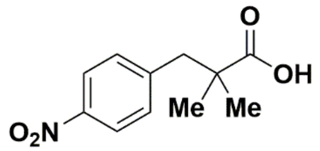

2,2-Dimethyl-3-(4-nitrophenyl)propanoic acid (1ae). The title compound was obtained in 32% crude yield (730.0 mg, 1.30 mmol) from pivalic acid (1.03 g, 10.0 mmol) and 4-nitroiodobenzene (2.01 g, 8.07 mmol), following literature procedure.[8] Following Procedure A, **1ae** (544.1 mg, 2.400 mmol) was then esterified with MeI (450 µL, 7.20 mmol) furnishing the methyl ester product in 65% yield (370 mg, 1.56 mmol). The ester product was purified via silica gel column chromatography (hexanes: ethyl acetate (12:1 v/v)). Finally, the methyl ester product (370 mg, 1.560 mmol) was hydrolyzed to the acid, furnishing the title compound **1ae** in 17% overall yield (290.0 mg, 1.299 mmol) as a white solid. ^{1}H NMR (600 MHz, $CDCl_3$): δ = 8.15 (d, J = 8.0 Hz, 2H), 7.34 (d, J = 8.0 Hz, 2H), 3.00 (s, 2H), 1.25 (s, 6H). ^{13}C{^{1}H} NMR (100 MHz, DMSO-d_6):

δ = 177.8, 146.5, 146.2, 131.2, 122.9, 44.7, 42.7, 24.7. The spectral data are consistent with those reported in the literature.[9]

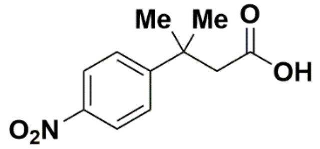

3-Methyl-3-(4-nitrophenyl)butanoic acid (1af). The title compound was obtained as a mixture of isomers in 86% crude yield (1.88 g, 8.42 mmol) by nitration of 3-methyl-3-(phenyl)butanoic acid (1.75 g, 10.0 mmol), synthesized according to literature procedure.[6] Following Procedure A, **1af** (1.88 g, 8.42 mmol) was then esterified with MeI (1570 µL, 25.3 mmol) furnishing the methyl ester product in 80% yield (1.59 g, 6.72 mmol). The ester product was purified via silica gel column chromatography (hexanes: ethyl acetate (12:1 v/v)). Finally, a portion of the methyl ester product (408.3 mg, 1.720 mmol) was hydrolyzed to the acid, furnishing the title compound **1af** in 66% overall yield (370.1 mg, 1.658 mmol) as an off-white solid. ^{1}H NMR (600 MHz, $CDCl_3$): δ = 8.09 (d, J = 9.0 Hz, 2H), 7.49 (d, J = 9.0 Hz, 2H), 2.62 (s, 2H), 1.44 (s, 6H). $^{13}C\{^{1}H\}$ NMR (150 MHz, $CDCl_3$ + 1 drop DMSO-d_6): δ = 173.2, 156.4, 146.1, 126.7, 123.4, 47.7, 37.5, 29.1. The spectral data are consistent with those reported in the literature.[6]

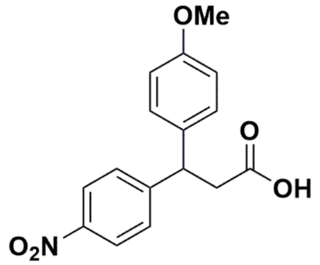

3-(4-Methoxyphenyl)-3-(4-nitrophenyl)propanoic acid (1ag). The title compound was synthesized in 9% yield (106.4 mg, 0.353 mmol) as a white solid from 4-methoxycinnamic acid (712.8 mg, 4.00 mmol) and 4-nitrophenylboronic acid (1.34 g, 8.03 mmol) following the literature procedure outlined in section VIII (Procedure B).[5] Purification by silica gel column chromatography (dichloromethane to dichloromethane: methanol (97:3, v/v) gradient elution). ^{1}H NMR (400 MHz, $CDCl_3$): δ = 8.14 (d, J = 8.8 Hz, 2H), 7.39 (d, J = 8.8 Hz, 2H), 7.12 (d, J = 8.8 Hz, 2H), 6.84 (d, J = 8.8 Hz, 2H), 4.58 (t, J = 7.9 Hz, 1H), 3.78 (s, 3H), 3.10 (d, J = 8.0 Hz, 2H). $^{13}C\{^{1}H\}$ NMR (150 MHz, $CDCl_3$): δ = 174.8, 158.9, 151.2, 146.9, 133.8, 128.7, 128.6, 124.1, 114.6, 55.5, 46.0, 39.9. FTIR (ATR, cm^{-1}): 2935, 2837, 1707, 1606, 1512, 1346, 1250, 1179, 1113. HRMS (ESI-MS) m/z calcd for $C_{16}H_{15}NO_5Na$ $[M + Na]^+$ 324.0842 found 324.0840.

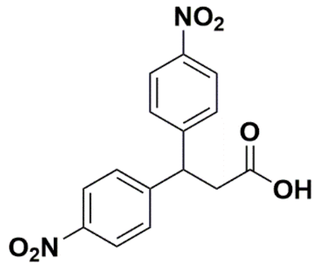

3,3-Bis(4-nitrophenyl)propanoic acid (1ah). The title compound was synthesized in 13% yield (168.6 mg, 0.5331 mmol) as a white solid from 4-nitrocinnamic acid (770 mg, 3.99 mmol) and 4-nitrophenylboronic acid (1.34 g, 8.03 mmol) following the literature procedure outlined in section VIII, Procedure B.[5] Purification by silica gel column chromatography (dichloromethane to dichloromethane: methanol (97:3, v/v) gradient elution). mp = 165 – 166 °C. ^{1}H NMR (400 MHz, $CDCl_3$): δ = 8.19 (d, *J* = 8.8 Hz, 4H), 7.40 (d, *J* = 8.8 Hz, 4H), 4.75 (t, *J* = 8.0 Hz, 1H), 3.18 (d, *J* = 8.0 Hz, 2H). $^{13}C\{^{1}H\}$ NMR (150 MHz, $CDCl_3$ + 2 drops DMSO-*d*$_6$): δ = 172.5, 149.7, 147.1, 128.8, 124.2, 46.7, 39.9. FTIR (ATR, cm^{-1}): 3073, 2856, 1712, 1596, 1594, 1515, 1343, 1109. HRMS (ESI-MS) *m/z* calcd for $C_{15}H_{11}N_2O_6$ [M - H]$^-$ 315.0623 found 315.0629.

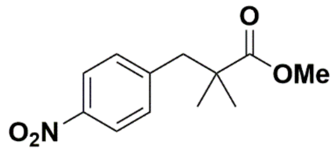

Methyl 2,2-dimethyl-3-(4-nitrophenyl)propionate (1ai). The title compound was obtained by esterification of **1ae** (544.1 mg, 2.400 mmol) with MeI (450 µL, 7.20 mmol) furnishing the methyl ester product in 65% yield (370 mg, 1.56 mmol) as an off-white solid. The ester product was purified via silica gel column chromatography (hexanes: ethyl acetate (12:1 v/v)). ^{1}H NMR (600 MHz, $CDCl_3$): δ = 8.12 (d, *J* = 9.0 Hz, 2H), 7.27 (d, *J* = 9.0 Hz, 2H), 3.66 (s, 3H), 2.95 (s, 2H), 1.20 (s, 6H). $^{13}C\{^{1}H\}$ NMR (150 MHz, $CDCl_3$): δ = 177.3, 147.0, 146.0, 131.0, 123.4, 52.1, 46.2, 43.9, 25.3. The spectral data are consistent with those reported in the literature.[9]

IX. General Procedure for the Catalytic Decarboxylative Elimination Reaction (2)

For substituted styrene products (**2a-2r**) and (**2ad**): On the benchtop, to an oven-dried 25-mL pear-shaped Schlenk flask was added a stir bar, 4-nitrohydrocinnamic acid **1a** (58.6 mg, 0.300 mmol), CuOAc (7.4 mg, 0.060 mmol), 2,2'-bipyridine (9.4 mg, 0.060 mmol), MnO_2 (52.2 mg, 0.600 mmol), and LiOAc (39.6 mg, 0.600 mmol). The flask was then sealed with a septum and wrapped with electrical tape. The flask was evacuated and backfilled with nitrogen three times after which dry DMA (3 mL) was added via syringe. The flask was then placed in a pre-heated oil bath and stirred under N_2 for 24 h at 120 °C. Upon completion, the reaction vessel was cooled to room temperature. Workup **Procedure A** was used for 4-nitrostyrene and substituted derivatives.

For nitrogen heterocycle products (**2s-2ac**): On the benchtop, to an oven-dried 25-mL pear-shaped Schlenk flask was added a stir bar, 3-(pyridine-4-yl)propanoic acid **1s** (60.5 mg, 0.400 mmol), CuOAc (58.8 mg, 0.480 mmol), and LiOAc (52.8 mg, 0.800 mmol). The flask was then sealed with a septum and wrapped with electrical tape. The flask was evacuated and backfilled with nitrogen three times after which dry DMA (4 mL) was added via syringe. The flask was then placed in a pre-heated oil bath and stirred under N_2 for 24 h at 140 °C. Upon completion, the reaction vessel was cooled to room temperature. Workup **Procedure B** was used for nitrogen heterocycles.

Procedure A, Acidic Aqueous Extraction (Used for Styrene Derivatives, Scheme 3a): The reaction mixture was diluted with ethyl acetate (20 mL) and added to a 500 mL separatory funnel along with 1 M HCl (400 mL). The layers were separated and the aqueous layer was extracted with ethyl acetate (3 x 30 mL). The combined organic layers were washed with brine (~250 mL), dried over Na_2SO_4 and gravity filtered into a round bottom flask. The solvent was removed under reduced pressure and a crude ^1H NMR spectrum of the residue was obtained. (Note: samples were stored in scintillation vials under N_2 in the freezer (-18 °C) when not in use to avoid decomposition of the styrene products.)

Purification Method 1: After the acidic aqueous extraction the residue was purified by silica gel column chromatography. A gradient elution of hexanes (100%) to hexanes: ethyl acetate: methanol (100:10:1, v/v) was used for styrene samples. (Note: the styrene samples decompose over time, and this can be observed by 2D-TLC)

Purification Method 2: After the acidic aqueous extraction a basic aqueous extraction was done to remove any remaining starting carboxylic acid or minor amounts of α,β-unsaturated carboxylic acid from the mixture. **Basic aqueous extraction:** The crude product mixture was diluted with dichloromethane (30 mL) and added to a separatory funnel. The organic layer was washed with 0.5 M aqueous ammonium hydroxide (50 mL), pH = ~10, as determined by pH paper. This process was repeated 2 more times with fresh aqueous layers each time. The aqueous layers were then combined and washed with dichloromethane (2 x 20 mL) to recover any organic product. The organic layers were combined, dried over Na_2SO_4 and gravity filtered into a round bottom flask. The product mixture was concentrated by rotary evaporation. To purify further, a filtration through filter paper was done using a solvent mixture of 60:1 hexanes: ethyl acetate (~150 mL). A brown solid was filtered off and a yellow filtrate containing the product was obtained. The filtrate was concentrated by rotary evaporation to afford a yellow oil. In certain cases, this residue could be subjected to a recrystallization (pentane/chloroform) to yield the pure product. In most cases however, the residue was purified via silica gel column chromatography (gradient elution of hexanes (100%) to hexanes: ethyl acetate: methanol (100:10:1, v/v)) to yield

the desired product. (Note: the styrene samples decompose over time, and this can be observed by 2D-TLC) If necessary, a recrystallization (pentane/chloroform) can be performed after the column to further purify the product.

Procedure B, Basic Aqueous Extraction (Used for Nitrogen Heterocycles, Scheme 3b): The reaction mixture was diluted with 30 mL of ethyl acetate (**2w, 2x**) or diethyl ether (**2s-2v**, **2y-2ac**) and added to a 500 mL separatory funnel and washed with 0.5 M aqueous ammonium hydroxide (100 mL). This process was repeated 2 more times with fresh aqueous layers each time. The aqueous layers were then combined and washed with (2 x 30 mL) ethyl acetate (**2w, 2x**) or diethyl ether (**2s-2v**, **2y-2ac**) to recover any organic product. The organic layers were combined washed with brine (~250 mL), dried over Na_2SO_4 and gravity filtered into a round bottom flask. The product mixture was concentrated by rotary evaporation (A cold-water bath, 1 to 4 °C, was used for samples **2s-2v, 2y, 2z** to avoid volatility concerns while concentrating). A crude ^{1}H NMR spectrum of the residue was obtained. The residue was purified by silica gel column chromatography. A 5 ¾ inch pipette was filled approximately halfway with silica and used as a short path column for the samples. A gradient elution of pentane (100 %) to pentane: diethyl ether (2:1, v/v) or mobile phase of DCM (100%) was used to yield the corresponding product. (Note: samples were stored in scintillation vials under N_2 in the freezer (-18 °C) when not in use to avoid decomposition of the vinyl products.)

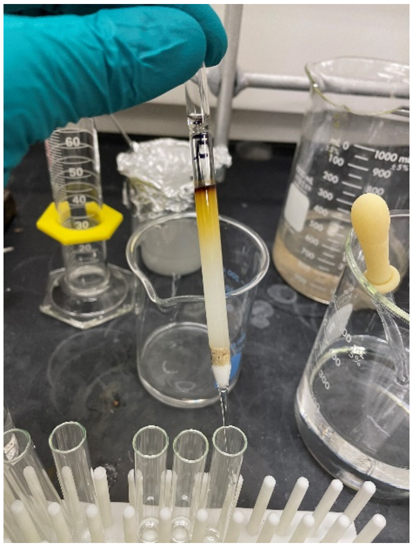

Representative Example of Short Path Column Setup

For indole products (**2w, 2x**) a 30 mm diameter column was used and a gradient elution of hexanes (100 %) to hexanes: ethyl acetate: methanol (100:4:1, v/v) yielded the corresponding products. (A recrystallization of 5-chloroindole from pentane/chloroform was necessary).

X. Characterization of Decarboxylative Elimination Products (2)

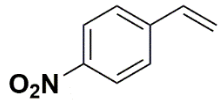

4-Nitrostyrene (2a). The title compound was synthesized from 4-nitrohydrocinnamic acid **(1a)** (58.6 mg, 0.300 mmol & 195.3 mg, 1.00 mmol) as a yellow oil in 62 % yield (27.8 mg, 0.186 mmol) and 55% yield (82.3 mg, 0.552 mmol) following the General Procedure in section IX (Reaction conducted at 120 °C, Procedure A, Purification Method 1). The title compound was synthesized from 2-(4-nitrophenyl)succinic acid (71.8 mg, 0.300 mmol) as a yellow oil in 66% yield (29.5 mg, 0.198 mmol) following the General Procedure in section IX (Reaction conducted at 120 °C , Procedure A, Purification Method 1). The product was purified by silica gel column chromatography (hexanes: ethyl acetate (15:1, v/v) gradient elution). ^{1}H NMR (400 MHz, $CDCl_3$): δ = 8.19 (d, *J* = 8.8 Hz, 2H), 7.54 (d, *J* = 8.8 Hz, 2H), 6.78 (dd, *J* = 17.6, 10.9 Hz, 1H), 5.93 (d, *J* = 17.6 Hz, 1H), 5.50 (d, *J* = 10.9 Hz, 1H). ^{13}C{^{1}H} NMR (150 MHz, $CDCl_3$): δ = 147.3, 144.0, 135.1, 126.9, 124.1, 118.7. The spectral data are consistent with those reported in the literature.[10]

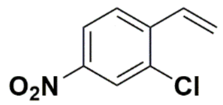

2-Chloro-4-nitrostyrene (2b). The title compound was synthesized from 2-chloro-4-nitrohydrocinnamic acid **(1b)** (68.8 mg, 0.300 mmol) as a light-yellow oil in 22% yield (12.1 mg, 0.0652 mmol) following the General Procedure in section IX (Reaction conducted at 90 °C, Procedure A, Purification Method 2). The product was purified by silica gel column chromatography (hexanes to hexanes: ethyl acetate: methanol (100:10:1, v/v) gradient elution). ^{1}H NMR (600 MHz, $CDCl_3$): δ = 8.26 (d, *J* = 2.4 Hz, 1H), 8.10 (dd, *J* = 9.0, 2.4 Hz, 1H), 7.72 (d, *J* = 9.0 Hz, 1H), 7.13 (dd, *J* = 17.4, 10.8 Hz, 1H), 5.92 (d, *J* = 17.4 Hz, 1H), 5.63 (d, *J* = 10.8 Hz, 1H). ^{13}C{^{1}H} NMR (150 MHz, $CDCl_3$): δ = 147.5, 142.2, 133.9, 131.9, 127.2, 125.3, 122.1, 121.0. The spectral data are consistent with those reported in the literature.[11]

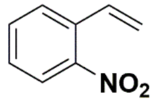

2-Nitrostyrene (2c). The title compound was synthesized from 2-nitrohydrocinnamic acid **(1c)** (58.6 mg, 0.300 mmol) using 1.2 equiv of CuOAc (44.2 mg, 0.361 mmol) and bpy (56.2 mg, 0.360 mmol) in the absence of MnO_2, furnishing the product as a faint yellow oil in 43% yield (19.2 mg, 0.129 mmol). The General Procedure in section IX was followed (Reaction conducted at 110 °C, Procedure A, Purification Method 2). The product was purified by silica gel column chromatography (hexanes to hexanes: ethyl acetate (20:1, v/v) gradient elution). ^{1}H NMR (600 MHz, $CDCl_3$): δ = 7.93 (dd, *J* = 7.8, 1.2 Hz, 1H), 7.63 (dd, *J* = 7.2, 1.2 Hz, 1H), 7.58 (td, *J* = 7.2, 0.6 Hz, 1H), 7.42 (td, *J* = 8.4, 1.2 Hz, 1H), 7.18 (dd, *J* = 17.4, 10.8 Hz, 1H), 5.74 (dd, *J* = 17.4, 1.2 Hz, 1H), 5.48 (dd, *J* = 10.8, 0.6 Hz, 1H). ^{13}C{^{1}H} NMR (150 MHz, $CDCl_3$): δ = 148.1, 133.5, 133.3, 132.7, 128.7, 128.5, 124.5, 119.1. The spectral data are consistent with those reported in the literature.[12]

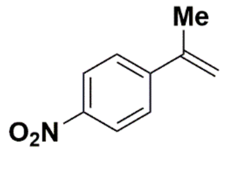

4-Nitro-*α*-methylstyrene (2d). The title compound was synthesized from 3-(4-nitrophenyl)butanoic acid **(1d)** (62.8 mg, 0.300 mmol) as an off-white/light-yellow solid in 80% yield (39.2 mg, 0.240 mmol) following the General Procedure in section IX (Reaction conducted at 120 °C, Procedure A, Purification Method 1). The product was purified by silica gel column chromatography (hexanes to hexanes: ethyl acetate (40:1, v/v) gradient elution). ^{1}H NMR (400 MHz, $CDCl_3$): δ = 8.19 (d, *J* = 8.8 Hz, 2H), 7.59 (d, *J* = 8.8 Hz, 2H), 5.52 (m, 1H), 5.29 (m, 1H), 2.19 (m, 3H). ^{13}C{^{1}H} NMR (150 MHz, $CDCl_3$): δ = 147.8, 147.2, 141.8, 126.4, 123.8, 116.6, 21.8. The spectral data are consistent with those reported in the literature.[13]

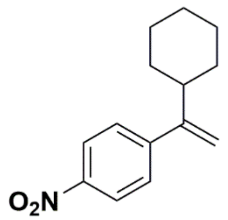

1-Cyclohexyl-1-(4-nitrophenyl)ethylene (2e). The title compound was synthesized from 3-cyclohexyl-3-(4-nitrophenyl)propanoic acid **(1e)** (83.2 mg, 0.300 mmol) as a light-yellow oil in 65% yield (44.9 mg, 0.194 mmol) following the General Procedure in section IX (Reaction conducted at 120 °C, Procedure A, Purification Method 1). The product was purified by silica gel column chromatography (hexanes to hexanes: ethyl acetate (20:1, v/v) gradient elution). ^{1}H NMR (400 MHz, $CDCl_3$): δ = 8.18 (d, *J* = 8.8 Hz, 2H), 7.47 (d, *J* = 8.8 Hz, 2H), 5.25 (m, 1H), 5.17 (m, 1H), 2.47 – 2.38 (m, 1H), 1.84 – 1.76 (m, 4H), 1.76 – 1.69 (m, 1H), 1.39 – 1.27 (m, 2H), 1.24 – 1.12 (m, 3H). ^{13}C{^{1}H} NMR (100 MHz, $CDCl_3$): δ = 153.4, 149.9, 147.0, 127.6, 123.7, 113.6, 42.5, 32.6, 26.8, 26.4. FTIR (cm^{-1}): 2926, 2853, 1596, 1515, 1344, 1110, 857. HRMS (ESI-MS) *m/z* calcd for $C_{14}H_{18}NO_2$ $[M + H]^+$ 232.1332 found 232.1330.

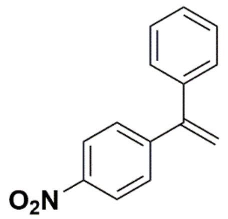

1-(4-Nitrophenyl)-1-phenylethylene (2f). The title compound was synthesized from 3-(4-nitrophenyl)-3-phenylpropanoic acid **(1f)** (81.4 mg, 0.300 mmol) as a pale-yellow solid in 90% yield (60.9 mg, 0.270 mmol) following the General Procedure in section IX (Reaction conducted at 120 °C, Procedure A, Purification Method 1). The product was purified by silica gel column chromatography (hexanes to hexanes: ethyl acetate (40:1, v/v) gradient elution). ^{1}H NMR (400 MHz, $CDCl_3$): δ = 8.20 (d, *J* = 9.2 Hz, 2H), 7.50 (d, *J* = 9.2 Hz, 2H), 7.39-7.34 (m, 3H), 7.32-7.27 (m, 2H), 5.63 (d, *J* = 0.8 Hz, 1H), 5.59 (s, *J* = 0.8 Hz, 1H). ^{13}C{^{1}H} NMR (150 MHz, $CDCl_3$): δ = 148.6, 148.2, 147.5, 140.3, 129.2, 128.7, 128.5, 128.3, 123.7, 117.4. The spectral data are consistent with those reported in the literature.[14]

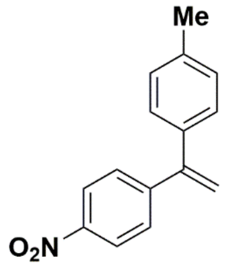

1-(4-Methylphenyl)-1-(4-nitrophenyl)ethylene (2g). The title compound was synthesized from 3-(4-methylphenyl)-3-(4-nitrophenyl)propanoic acid **(1g)** (85.6 mg, 0.300 mmol) as a light-yellow solid in 84% yield (60.3 mg, 0.252 mmol) following the General Procedure in section IX (Reaction conducted at 120 °C, Procedure A, Purification Method 1). The product was purified by silica gel column chromatography (hexanes to hexanes: ethyl acetate (40:1, v/v) gradient elution). ^{1}H NMR (400 MHz, $CDCl_3$): δ = 8.19 (d, *J* = 8.9 Hz, 2H), 7.49 (d, *J* = 8.9 Hz, 2H), 7.18 (s, 4H), 5.60 (d, *J* = 0.8 Hz, 1H), 5.53 (d, *J* = 0.8 Hz, 1H), 2.39 (s, 3H). $^{13}C\{^1H\}$ NMR (150 MHz, $CDCl_3$): δ = 148.50, 148.46, 147.5, 138.5, 137.4, 129.4, 129.2, 128.2, 123.7, 116.7, 21.4. The spectral data are consistent with those reported in the literature.[15]

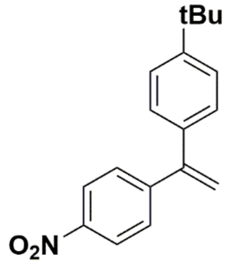

1-(4-Nitrophenyl)-1-(4-*tert*-butylphenyl)ethylene (2h). The title compound was synthesized from 3-(4-nitrophenyl)-3-(4-*tert*-butylphenyl)propanoic acid **(1h)** (98.3 mg, 0.300 mmol) as a yellow oil in 91% yield (76.6 mg, 0.272 mmol) following the General Procedure in section IX (Reaction conducted at 120 °C, Procedure A, Purification Method 1). The product was purified by silica gel column chromatography (hexanes to hexanes: ethyl acetate (20:1, v/v) gradient elution). ^{1}H NMR (400 MHz, $CDCl_3$): δ = 8.19 (d, *J* = 8.6 Hz, 2H), 7.51 (d, *J* = 8.6 Hz, 2H), 7.38 (d, *J* = 8.4 Hz, 2H), 7.23 (d, *J* = 8.4 Hz, 2H), 5.62 (s, 1H), 5.53 (s, 1H), 1.35 (s, 9H). $^{13}C\{^1H\}$ NMR (150 MHz, $CDCl_3$): δ = 151.7, 148.5, 148.4, 147.5, 137.3, 129.2, 128.0, 125.6, 123.7, 116.8, 34.8, 31.5. FTIR (cm^{-1}): 3080, 2962, 2868, 1595, 1514, 1343, 1106, 859, 842. HRMS (ESI-MS) *m/z* calcd for $C_{18}H_{18}NO_2$ $[M - H]^-$ 280.1343 found 280.1350.

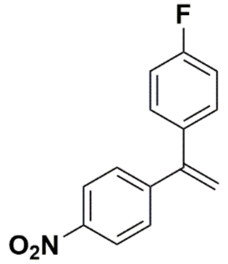

1-(4-Fluorophenyl)-1-(4-nitrophenyl)ethylene (2i). The title compound was synthesized from 3-(4-fluorophenyl)-3-(4-nitrophenyl)propanoic acid **(1i)** (86.8 mg, 0.300 mmol) as light-yellow

crystals in 68% yield (49.3 mg, 0.203 mmol) following the General Procedure in section IX (Reaction conducted at 120 °C, Procedure A, Purification Method 2). The product was obtained by recrystallization (pentane). mp = 64 – 66 °C. ^{1}H NMR (400 MHz, $CDCl_3$): δ = 8.20 (d, *J* = 8.8 Hz, 2H), 7.48 (d, *J* = 8.8 Hz, 2H), 7.29 – 7.24 (m, 2H), 7.09 – 7.03 (m, 2H), 5.59 (s, 1H), 5.57 (s, 1H). ^{13}C{^{1}H} NMR (150 MHz, $CDCl_3$): δ = 163.0 (d), 148.0, 147.6, 136.4 (d), 130.0 (d), 129.1, 123.8, 117.4 (d), 115.8, 115.6. FTIR (ATR, cm^{-1}): 3073, 2927, 2848, 1596, 1517, 1506, 1346, 1317, 1217, 842, 835, 706. HRMS (ESI-MS) *m/z* calcd for $C_{14}H_9FNO_2$ $[M - H]^-$ 242.0623 found 242.0626.

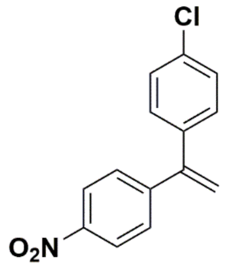

1-(4-Chlororphenyl)-1-(4-nitrophenyl)ethylene (2j). The title compound was synthesized from 3-(4-chlorophenyl)-3-(4-nitrophenyl)propanoic acid **(1j)** (91.7 mg, 0.300 mmol) as yellow crystals in 71% yield (55.2 mg, 0.213 mmol) following the General Procedure in section IX (Reaction conducted at 120 °C, Procedure A, Purification Method 2). The product was obtained by recrystallization (pentane). mp = 90 – 92 °C. ^{1}H NMR (400 MHz, $CDCl_3$): δ = 8.20 (d, *J* = 8.8 Hz, 2H), 7.47 (d, *J* = 8.8 Hz, 2H), 7.34 (d, *J* = 8.4 Hz, 2H), 7.23 (d, *J* = 8.4 Hz, 2H), 5.62 (s, 1H), 5.60 (s, 1H). ^{13}C{^{1}H} NMR (150 MHz, $CDCl_3$): δ = 147.7, 147.6, 147.5, 138.8, 134.5, 129.6, 129.1, 128.9, 123.8, 117.8. FTIR (ATR, cm^{-1}): 3070, 2930, 2840, 1594, 1509, 1488, 1344, 1318, 1088, 1013, 861, 831, 707. HRMS (ESI-MS) *m/z* calcd for $C_{14}H_9ClNO_2$ $[M - H]^-$ 258.0327 found 258.0333.

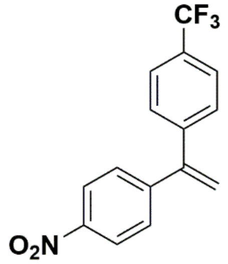

1-(4-Nitrophenyl)-1-(4-trifluoromethylphenyl)ethylene (2k). The title compound was synthesized from 3-(4-nitrophenyl)-3-(4-trifluoromethylphenyl)propanoic acid **(1k)** (101.8 mg, 0.300 mmol) as yellow crystals in 65% yield (56.8 mg, 0.193 mmol) following the General Procedure in section IX (Reaction conducted at 120 °C, Procedure A, Purification Method 2). The product was obtained by recrystallization (pentane). mp = 70 – 72 °C. ^{1}H NMR (400 MHz, $CDCl_3$): δ = 8.22 (d, *J* = 8.8 Hz, 2H), 7.63 (d, *J* = 8.0 Hz, 2H), 7.48 (d, *J* = 8.8 Hz, 2H), 7.41 (d, *J* = 8.0 Hz, 2H), 5.70 (s, 1H), 5.70 (s, 1H). ^{13}C{^{1}H} (150 MHz, $CDCl_3$): δ = 147.8, 147.5, 147.2, 143.9, 130.6 (q), 129.1, 128.7, 125.7 (q), 124.2 (q), 123.9, 119.0. FTIR (ATR, cm^{-1}): 3103, 3081, 2931, 2854, 1600, 1596, 1515, 1346, 1323, 1157, 1112, 1075, 1062, 1016, 927, 851, 709. HRMS (ESI-MS) *m/z* calcd for $C_{15}H_{10}F_3NO_2$ $[M]^-$ 293.0669 found 293.0666.

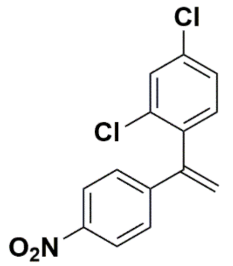

1-(2,4-Dichlorophenyl)-1-(4-nitrophenyl)ethylene (2l). The title compound was synthesized from 3-(2,4-dichlorophenyl)-3-(4-nitrophenyl)propanoic acid **(1l)** (102.0 mg, 0.300 mmol) as yellow crystals in 51% yield (45.0 mg, 0.153 mmol) following the General Procedure in section IX (Reaction conducted at 120 °C, Procedure A, Purification Method 2). The product was purified by silica gel column chromatography (hexanes: ethyl acetate: methanol (100:10:1.5, v/v)). mp = 79 – 80 °C. ^{1}H NMR (600 MHz, $CDCl_3$): δ = 8.17 (d, *J* = 9.0 Hz, 2H), 7.44 (d, *J* = 1.8 Hz, 1H), 7.39 (d, *J* = 9.0 Hz, 2H), 7.34 (dd, *J* = 8.4, 1.8 Hz, 1H), 7.28 (d, *J* = 8.4 Hz, 1H), 5.96 (s, 1H), 5.50 (s, 1H). $^{13}C\{^1H\}$ NMR (150 MHz, $CDCl_3$): δ =147.5, 145.9, 145.3, 138.0, 135.1, 134.2, 132.4, 130.0, 127.6, 127.3, 124.0, 120.7. FTIR (ATR, cm^{-1}): 3083, 2935, 2847,1596, 1514, 1471, 1344, 1104, 859, 843. HRMS (ESI-MS) *m/z* calcd for $C_{14}H_9Cl_2NO_2$ $[M]^-$ 293.0016 found 293.0014.

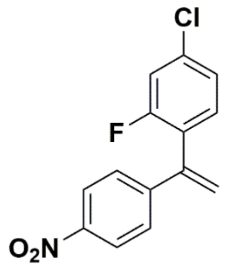

1-(2-Fluoro-4-chlorophenyl)-1-(4-nitrophenyl)ethylene (2m). The title compound was synthesized from 3-(2-fluoro-4-chlorophenyl)-3-(4-nitrophenyl)propanoic acid **(1m)** (97.1 mg, 0.300 mmol) as orange crystals in 45% yield (37.5 mg, 0.135 mmol) following the General Procedure in section IX (Reaction conducted at 120 °C, Procedure A, Purification Method 2). The product was purified by silica gel column chromatography (hexanes: ethyl acetate: methanol (100:10:1.5, v/v)). mp = 65 – 66 °C. ^{1}H NMR (600 MHz, $CDCl_3$): δ = 8.18 (d, *J* = 9.0 Hz, 2H), 7.44 (d, *J* = 8.8 Hz, 2H), 7.25 – 7.22 (m, 1H), 7.19 (dd, *J* = 5.6, 1.6 Hz, 1H), 7.14 (dd, *J* = 9.6, 1.8 Hz, 1H), 5.87 (s, 1H), 5.62 (s, 1H). $^{13}C\{^1H\}$ NMR (150 MHz, $CDCl_3$): δ = 159.9 (d), 147.6, 146.7, 142.1, 135.4 (d), 132.1 (d), 127.7, 126.8 (d), 125.0 (d), 123.9, 121.0, 117.1 (d). FTIR (ATR, cm^{-1}): 3074, 2933, 2845 1598, 1596, 1515, 1508, 1409, 1344, 1218, 1083, 902, 858. HRMS (ESI-MS) *m/z* calcd for $C_{14}H_9ClFNO_2$ $[M]^-$ 277.0311 found 277.0309.

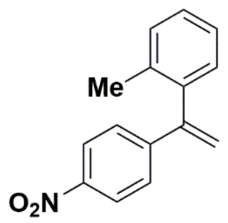

1-(2-Methylphenyl)-1-(4-nitrophenyl)ethylene (2n). The title compound was synthesized from 3-(2-methylphenyl)-3-(4-nitrophenyl)propanoic acid **(1n)** (85.6 mg, 0.300 mmol) as an off-white solid in 71% yield (50.8 mg, 0.212 mmol) following the General Procedure in section IX (Reaction conducted at 120 °C, Procedure A, Purification Method 2). The product was purified by silica gel column chromatography (hexanes to hexanes: ethyl acetate: methanol (100:10:1.5, v/v) gradient elution). mp = 55 – 57 °C. ^{1}H NMR (600 MHz, $CDCl_3$): δ = 8.15 (d, J = 9.0 Hz, 2H), 7.41 (d, J = 9.0 Hz, 2H), 7.30 (dt, J = 7.8, 1.2 Hz, 1H), 7.26 – 7.24 (m, 1H), 7.22 – 7.21 (m, 2H), 5.92 (s, 1H), 5.42 (s, 1H), 2.02 (s, 3H). ^{13}C{^{1}H} NMR (150 MHz, $CDCl_3$): δ = 148.1, 147.3, 147.2, 140.3, 136.1, 130.6, 130.2, 128.4, 127.4, 126.3, 124.0, 118.9, 20.3. FTIR (ATR, cm^{-1}): 3072, 2923, 1595 ,1517, 1342, 860. HRMS (ESI-MS) *m/z* calcd for $C_{15}H_{14}NO_2$ $[M + H]^+$ 240.1019 found 240.1017.

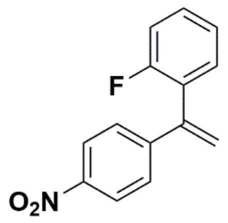

1-(2-Fluorophenyl)-1-(4-nitrophenyl)ethylene (2o). The title compound was synthesized from 3-(2-fluorophenyl)-3-(4-nitrophenyl)propanoic acid **(1o)** (86.8 mg, 0.300 mmol) as a light-yellow solid in 62% yield (45.3 mg, 0.186 mmol) following the General Procedure in section IX (Reaction conducted at 120 °C, Procedure A, Purification Method 2). The product was obtained by recrystallization (pentane). mp = 49 – 50 °C. ^{1}H NMR (400 MHz, $CDCl_3$): δ = 8.18 (d, J = 8.8 Hz, 2H), 7.46 (d, J = 8.8, 2H), 7.40 – 7.35 (m, 1H), 7.30 (td, J = 7.6, 2.0 Hz, 1H), 7.19 (td, J = 7.6, 1.2 Hz, 1H), 7.11 – 7.07 (m, J = 7.8 Hz, 1H), 5.87 (s, 1H), 5.62 (s, 1H). ^{13}C NMR (150 MHz, $CDCl_3$): δ = 160.1 (d), 147.5, 146.2, 143.0, 131.5 (d), 130.4 (d), 127.7, 124.6 (d), 123.9, 120.5 (d), 116.3, 116.1. FTIR (ATR, cm^{-1}): 3081, 2931, 2835, 1595, 1510, 1486, 1448, 1340, 1212, 1097, 860, 754, 745, 715. HRMS (ESI-MS) *m/z* calcd for $C_{14}H_{11}FNO_2$ $[M - H]^-$ 244.0768 found 244.0763.

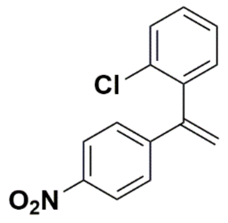

1-(2-Chlorophenyl)-1-(4-nitrophenyl)ethylene (2p). The title compound was synthesized from 3-(2-chlorophenyl)-3-(4-nitrophenyl)propanoic acid **(1p)** (91.7 mg, 0.300 mmol) as yellow crystals in 52% yield (40.2 mg, 0.158 mmol) following the General Procedure in section IX (Reaction conducted at 120 °C, Procedure A, Purification Method 2). The product was purified by silica gel column chromatography (hexanes: ethyl acetate: methanol (100:10:1.5, v/v))

mp = 106 – 108 °C. ^{1}H NMR (600 MHz, $CDCl_3$): δ = 8.16 (d, *J* = 8.4 Hz, 2H), 7.43 – 7.40 (m, 3H), 7.37 – 7.33 (m, 3H), 5.96 (s, 1H), 5.51 (s, 1H). ^{13}C{^{1}H} NMR (150 MHz, $CDCl_3$): δ = 147.4, 146.4, 146.3, 139.4, 133.3, 131.7, 130.1, 129.8, 127.4, 127.3, 123.9, 120.2. FTIR (ATR, cm^{-1}): 3073, 2935, 2847, 1595, 1513, 1340, 1110, 1046, 920, 858, 747. HRMS (ESI-MS) *m/z* calcd for $C_{14}H_{10}ClNO_2$ $[M]^-$ 259.0406 found 259.0402.

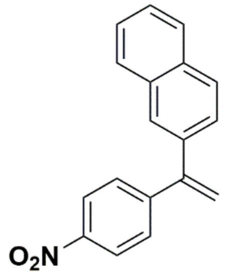

1-(Naphthyl)-1-(4-nitrophenyl)ethylene (2q). The title compound was synthesized from 3-(4-naphthyl)-3-(4-nitrophenyl)propanoic acid **(1q)** (96.4 mg, 0.300 mmol) as a light-yellow solid in 61% yield (50.5 mg, 0.183 mmol) following the General Procedure in section IX (Reaction conducted at 120 °C, Procedure A, Purification Method 2). The product was obtained by recrystallization (pentane). mp = 67 – 68 °C. ^{1}H NMR (400 MHz, $CDCl_3$): δ = 8.22 (d, *J* = 8.8 Hz, 2H), 7.88 – 7.78 (m, 3H), 7.73 (d, *J* = 1.6 Hz, 1H), 7.56 – 7.48 (m, 4H), 7.43 (dd, *J* = 8.4, 2.0 Hz, 1H), 5.77 (d, *J* = 0.4 Hz, 1H), 5.68 (d, *J* = 0.4 Hz, 1H). ^{13}C{^{1}H} NMR (100 MHz, $CDCl_3$): δ = 148.6, 148.3, 147.6, 137.7, 133.4, 133.3, 129.3, 128.37, 128.36, 127.9, 127.6, 126.71, 126.67, 126.0, 123.8, 117.8. FTIR (ATR, cm^{-1}): 3057, 2929, 2854,1592, 1513, 1339, 1324, 1108, 915, 856, 821, 752. HRMS (ESI-MS) *m/z* calcd for $C_{18}H_{12}NO_2$ $[M - H]^-$ 274.0874 found 274.0881.

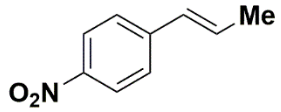

1-Nitro-4-(1-propen-1-yl)benzene (2r). The title compound was synthesized from 2-methyl-3-(4-nitrophenyl)propanoic acid **(1r)** (68.2 mg, 0.300 mmol) using 1.2 equiv of CuOAc (44.2 mg, 0.361 mmol) and bpy (56.2 mg, 0.360 mmol) in the absence of MnO_2, furnishing the product as an off-white solid in 61% yield (29.9 mg, 0.183 mmol). The General Procedure in section IX was followed (Reaction conducted at 110 °C, Procedure A, Purification Method 2). The product was purified by silica gel column chromatography (hexanes to hexanes: ethyl acetate (20:1, v/v) gradient elution). ^{1}H NMR (600 MHz, $CDCl_3$): δ = 8.14 (d, *J* = 9.0 Hz, 2H), 7.44 (d, *J* = 9.0 Hz, 2H), 6.49 – 6.42 (m, 2H), 1.95 (d, *J* = 4.8 Hz, 3H).^{13}C{^{1}H} NMR (150 MHz, $CDCl_3$): δ = 146.6, 144.6, 131.4, 129.6, 126.4, 124.1, 18.9. The spectral data are consistent with those reported in the literature.[16]

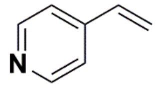

4-Vinylpyridine (2s). The title compound was synthesized from 3-(pyridine-4-yl)propanoic acid **(1s)** (60.5 mg, 0.400 mmol) as a yellow oil in 42% yield (17.7 mg, 0.168 mmol) following the General Procedure in section IX (Reaction conducted at 140 °C, Procedure B). The product was purified by silica gel column chromatography (pentane to pentane: diethyl ether (2:1, v/v) gradient

elution). ^{1}H NMR (400 MHz, $CDCl_3$): δ = 8.58 (bs, 2H), 7.28 (d, *J* = 4.8 Hz, 2H), 6.65 (dd, *J* = 17.6, 10.8 Hz, 1H), 5.97 (d, *J* = 17.6 Hz, 1H), 5.48 (d, *J* = 10.8 Hz, 1H). $^{13}C\{^{1}H\}$ NMR (150 MHz, $CDCl_3$): δ = 150.2, 145.0, 134.9, 121.0, 118.9. The spectral data are consistent with those reported in the literature.[17]

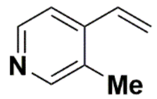

3-Methyl-4-vinylpyridine (2t). The title compound was synthesized from 3-(2-methyl-pyridin-4-yl)propanoic acid **(1t)** (66.1 mg, 0.400 mmol) as a yellow oil in 40% yield (18.9 mg, 0.159 mmol) following the General Procedure in section IX (Reaction conducted at 140 °C, Procedure B). The product was purified by silica gel column chromatography (dichloromethane to dichloromethane: methanol (100:0.25, v/v) gradient elution). ^{1}H NMR (400 MHz, $CDCl_3$): δ = 8.39 (m, 2H), 7.31 (d, *J* = 5.2 Hz, 1H), 6.86 (dd, *J* = 17.2, 10.8 Hz, 1H), 5.84 (dd, *J* = 17.6, 0.8 Hz, 1H), 5.50 (dd, *J* = 10.8, 0.8 Hz, 1H), 2.32 (s, 3H). $^{13}C\{^{1}H\}$ NMR (150 MHz, $CDCl_3$): δ = 151.4, 147.8, 144.0, 132.8, 130.4, 119.4, 119.3, 16.6. The spectral data are consistent with those reported in the literature.[18]

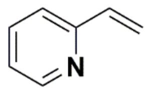

2-Vinylpyridine (2u). The title compound was synthesized from 3-(pyridin-2-yl)propanoic acid **(1u)** (60.5 mg, 0.400 mmol) as a colorless oil in 27% yield (11.2 mg, 0.107 mmol) following the General Procedure in section IX (Reaction conducted at 140 °C, Procedure B). The product was purified by silica gel column chromatography (pentane to pentane: diethyl ether (2:1, v/v) gradient elution). ^{1}H NMR (600 MHz, $CDCl_3$): δ = 8.58 (d, *J* = 4.8 Hz, 1H), 7.67 (dt, *J* = 7.8, 1.8 Hz, 1H), 7.37 (d, *J* = 7.8 Hz, 1H), 7.19 – 7.17 (m, 1H), 6.23 (d, *J* = 17.4 Hz, 1H), 5.51 (d, *J* = 10.8 Hz, 1H). $^{13}C\{^{1}H\}$ NMR (150 MHz, $CDCl_3$): δ = 155.7, 149.2, 137.1, 136.7, 122.7, 121.5, 118.9. The spectral data are consistent with those reported in the literature.[19]

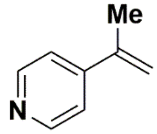

4-(1-Methylvinyl)pyridine (2v). The title compound was synthesized from 3-(pyridine-4-yl)butanoic acid **(1v)** (66.1 mg, 0.400 mmol) as a faint yellow oil in 35% yield (16.7 mg, 0.140 mmol) following the General Procedure in section IX (Reaction conducted at 140 °C, Procedure B). The product was purified by silica gel column chromatography (dichloromethane to dichloromethane: methanol (100:0.25, v/v) gradient elution). ^{1}H NMR (400 MHz, $CDCl_3$): δ = 8.58 (bs, 2H), 7.33 (d, *J* = 3.6 Hz, 2H), 5.57 (m, 1H), 5.26 (m, 1H), 2.14 (q, 3H). $^{13}C\{^{1}H\}$ NMR (100 MHz, $CDCl_3$): δ = 150.0, 148.4, 141.1, 120.3, 116.0, 21.1. The spectral data are consistent with those reported in the literature.[20]

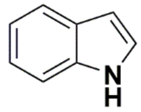

Indole (2w). The title compound was synthesized from indoline-2-carboxylic acid **(1w)** (49.0 mg, 0.300 mmol) as an off-white solid in 45% yield (15.7 mg, 0.134 mmol) following the General Procedure in section IX (Reaction conducted at 140 °C, Procedure B). The product was purified by silica gel column chromatography (hexanes to 1% methanol in hexanes: ethyl acetate (25:1, v/v) gradient elution). ^{1}H NMR (400 MHz, $CDCl_3$): δ = 8.13 (s, br, 1H), 7.66 (d, *J* = 8.0 Hz, 1H), 7.41 (d, *J* = 8.0 Hz, 1H), 7.23 – 7.11 (m, 3H), 6.58 – 6.56 (m, 1H). ^{13}C{^{1}H} NMR (150 MHz, $CDCl_3$): δ = 136.0, 128.0, 124.3, 122.2, 120.9, 120.0, 111.2, 102.8. The spectral data are consistent with those reported in the literature.[21]

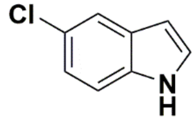

5-Chloroindole (2x). The title compound was synthesized from 5-chloroindoline-2-carboxylic acid hydrochloride hydrate **(1x)** (75.6 mg, 0.300 mmol) as an off-white solid in 32% yield (14.4 mg, 0.0950 mmol) following the General Procedure in section IX (Reaction conducted at 140 °C, 3 equiv LiOAc used, Procedure B). The product was purified by silica gel column chromatography (hexanes to 1% methanol in hexanes: ethyl acetate (35:1, v/v) gradient elution). Recrystallization from pentane/chloroform was necessary. ^{1}H NMR (600 MHz, $CDCl_3$): δ = 8.18 (s, br, 1H), 7.61 (d, *J* = 1.8 Hz, 1H), 7.31 (d, *J* = 8.4 Hz, 1H), 7.23 (t, *J* = 2.4 Hz, 1H), 7.15 (dd, *J* = 8.4, 2.4 Hz, 1H), 6.51 – 6.50 (m, 1H). ^{13}C{^{1}H} NMR (150 MHz, $CDCl_3$): δ = 134.3, 129.1, 125.68, 125.66, 122.5, 120.3, 112.1, 102.6. The spectral data are consistent with those reported in the literature.[22]

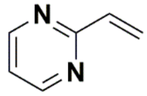

2-Vinylpyrimidine (2y). The title compound was synthesized from 3-(pyrimidin-2-yl)propanoic acid **(1y)** (60.9 mg, 0.400 mmol) as a colorless oil in 36% yield (15.4 mg, 0.144 mmol). following the General Procedure in section IX (Reaction conducted at 140 °C, Procedure B). The product was purified by silica gel column chromatography (pentane to pentane: diethyl ether (2:1, v/v) gradient elution). ^{1}H NMR (400 MHz, $CDCl_3$): δ = 8.71 (d, *J* = 5.2 Hz, 2H), 7.15 (t, *J* = 4.8 Hz, 1H), 6.89 (dd, *J* = 17.6, 10.8 Hz, 1H), 6.63 (dd, *J* = 17.6, 1.6 Hz, 1H), 6.20 (dd, *J* = 10.8, 1.6 Hz, 1H). ^{13}C{^{1}H} NMR (150 MHz, $CDCl_3$): δ = 164.5, 157.2, 136.5, 124.3, 119.3. The spectral data are consistent with those reported in the literature.[23]

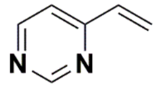

4-Vinylpyrimidine (2z). The title compound was synthesized from 3-(pyrimidin-4-yl)propanoic acid **(1z)** (60.9 mg, 0.400 mmol) as a colorless oil in 47% yield (20.0 mg, 0.187 mmol) following the General Procedure in section IX (Reaction conducted at 140 °C, Procedure B). The product was purified by silica gel column chromatography (pentane to pentane: diethyl ether (2:1, v/v) gradient elution). ^{1}H NMR (400 MHz, $CDCl_3$): δ = 9.16 (s, 1H), 8.69 (d, *J* = 5.6 Hz, 1H), 7.30 (dd,

J = 7.2, 1.6 Hz, 1H), 6.78 – 6.71 (m, 1H), 6.49 (dd, J = 17.6, 1.2 Hz, 1H), 5.73 (dd, J = 10.8, 1.2 Hz, 1H). $^{13}C\{^1H\}$ NMR (150 MHz, $CDCl_3$): δ = 162.5, 159.0, 157.5, 135.2, 123.6, 118.3. The compound is known in the literature.[24]

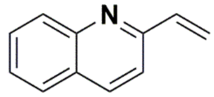

2-Vinylquinoline (2aa). The title compound was synthesized from 3-(quinolin-2-yl)propanoic acid **(1aa)** (80.5 mg, 0.400 mmol) as a yellow oil in 37% yield (22.8 mg, 0.147 mmol) following the General Procedure in section IX (Reaction conducted at 140 °C, Procedure B). The product was purified by silica gel column chromatography (dichloromethane (100%)). ^1H NMR (400 MHz, $CDCl_3$): δ = 8.12 (d, J = 8.8 Hz, 1H), 8.07 (d, J = 8.4 Hz, 1H), 7.79 (dd, J = 8.4, 1.2 Hz, 1H), 7.70 (td, J = 7.2, 1.6 Hz, 1H), 7.62 (d, J = 8.4 Hz, 1H), 7.51 (td, J = 7.2, 1.2 Hz, 1H), 7.04 (dd, J = 18.0, 10.8 Hz, 1H), 6.28 (dd, J = 18.0, 0.8 Hz, 1H), 5.67 (dd, J= 10.8, 0.8 Hz, 1H). $^{13}C\{^1H\}$ NMR (150 MHz, $CDCl_3$): δ = 156.2, 148.1, 138.1, 136.6, 129.9, 129.5, 127.6, 126.5, 120.1, 118.5. The spectral data are consistent with those reported in the literature.[25]

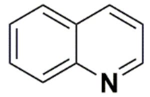

Quinoline (2ab). The title compound was synthesized from 1,2,3,4-tetrahydroquinoline-3-carboxylic acid **(1ab)** (70.9 mg, 0.400 mmol) using 20 mol% CuOAc (9.8 mg, 0.080 mmol) and 4 equiv MnO_2 (139.1 mg, 1.600 mmol) furnishing the product as a colorless oil in 42% yield (21.6 mg, 0.167 mmol). The General Procedure in section IX was followed (Reaction conducted at 140 °C, Procedure B). The product was purified by silica gel column chromatography (pentane to pentane: diethyl ether (2:1, v/v) gradient elution). ^1H NMR (400 MHz, $CDCl_3$): δ = 8.93 (dd, J = 4.0, 1.6 Hz, 1H), 8.19 (d, J = 8.4 Hz, 1H), 8.15 (d, J = 8.4 Hz, 1H), 7.84 (d, J = 8.0 Hz, 1H), 7.74 (td, J = 6.8, 1.6 Hz, 1H), 7.57 (td, J = 7.2, 1.2 Hz, 1H), 7.44 – 7.41 (m, 1H). $^{13}C\{^1H\}$ NMR (150 MHz, $CDCl_3$): δ = 150.3, 148.1, 136.5, 129.8, 129.4, 128.5, 128.0, 126.8, 121.2. The spectral data are consistent with those reported in the literature.[26]

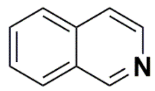

Isoquinoline (2ac). The title compound was synthesized from 1,2,3,4-tetrahydroisoquinoline-3-carboxylic acid **(1ac)** (70.9 mg, 0.400 mmol) using 20 mol% CuOAc (9.8 mg, 0.080 mmol) and 4 equiv MnO_2 (139.1 mg, 1.600 mmol) furnishing the product as light-yellow platelets in 32% yield (16.8 mg, 0.130 mmol). The General Procedure in section IX was followed (Reaction conducted at 140 °C, Procedure B). The product was purified by silica gel column chromatography (pentane to pentane: diethyl ether (2:1, v/v) gradient elution). ^1H NMR (400 MHz, $CDCl_3$): δ = 9.35 (s, 1H), 8.53 (d, J = 5.6 Hz, 1H), 8.09 (d, J = 8.4 Hz, 1H), 7.93 (d, J = 8.0 Hz, 1H), 7.86 – 7.42 (m, 2H), 7.73 (td, J = 6.8, 1.2 Hz, 1H). $^{13}C\{^1H\}$ NMR (150 MHz, $CDCl_3$): δ = 152.3, 142.5, 136.2, 130.9, 128.8, 128.0, 127.7, 126.7, 120.9. The spectral data are consistent with those reported in the literature.[27]

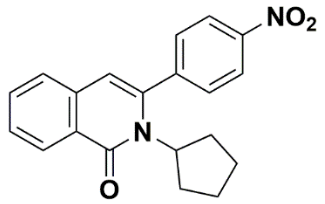

2-Cyclopentyl-3-(4-nitrophenyl)-1(2*H*)-isoquinolinone (2ad) The title compound was synthesized from 2-cyclopentyl-3-(4-nitrophenyl)-1-oxo-1,2,3,4-tetrahydro-4-isoquinolinecarboxylic acid (**1ad**) (114.1 mg, 0.300 mmol) as a yellow solid in 39% yield (38.9 mg, 0.116 mmol) following the General Procedure in section IX (Procedure A, Purification Method 2). The product was purified by silica gel column chromatography (hexanes to hexanes: ethyl acetate (15:1, v/v) gradient elution). mp = 194 – 195 °C. ^{1}H NMR (600 MHz, $CDCl_3$): δ = 8.42 (d, *J* = 7.8 Hz, 1H), 8.35 (d, *J* = 9.0 Hz, 2H), 7.66 – 7.62 (m, 3H), 7.51 (td, *J* = 4.8, 0.4 Hz, 1H), 7.48 (d , *J* = 7.8 Hz, 1H), 6.38 (s, 1H), 4.14 – 4.09 (m, 1H), 2.52 – 2.46 (m, 2H), 2.12 – 2.06 (m, 2H), 1.72 – 1.67 (m, 2H), 1.52 – 1.45 (m, 2H). ^{13}C{^{1}H} NMR (150 MHz, $CDCl_3$): δ = 162.7, 148.1, 143.8, 142.7, 135.7, 132.6, 129.6, 127.8, 127.5, 126.9, 126.0, 124.1, 108.9, 62.3, 29.4, 26.4. FTIR (cm^{-1}): 3065, 2952, 2866, 1649, 1622, 1593, 1519, 1484, 1345, 1106, 855, 758. HRMS (ESI-MS) *m/z* calcd for $C_{20}H_{18}N_2O_3Na$ $[M + Na]^+$ 357.1210 found 357.1207.

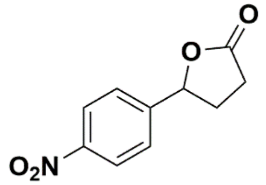

4-(4-Nitrophenyl)-γ-butyrolactone (4). The title compound was synthesized from 3-(4-nitrophenyl)butanoic acid following the General Procedure in section II as a white solid (14% NMR yield). The product was purified by silica gel column chromatography (hexanes: ethyl acetate (12:1, v/v) gradient elution). ^{1}H NMR (400 MHz, $CDCl_3$): δ = 8.27 (d, *J* = 8.4 Hz, 2H), 7.53 (d, *J* = 8.4 Hz, 2H), 5.60 (t, *J* = 8.0 Hz, 1H), 2.81 – 2.63 (m, 3H), 2.16 – 2.11 (m, 1H). ^{13}C{^{1}H} NMR (100 MHz, $CDCl_3$): δ = 176.1, 146.8, 126.1, 124.3, 79.8, 31.1, 28.8. The spectral data are consistent with those reported in literature.[28]

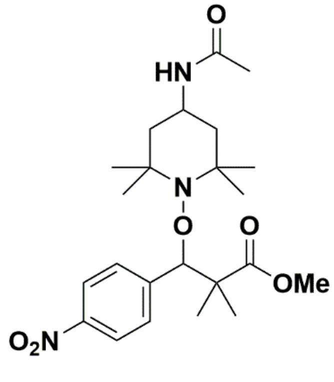

2,2-Dimethyl-[3-[4-(acetamido)-2,2,6,6-tetramethyl-1-piperidinyl]oxy]-3-(4-nitrophenyl)methyl propionate (6) The title compound was synthesized following the General Procedure in section VI - D, as a light-orange oil in 46% ^{1}H NMR yield. The product was purified

by silica gel column chromatography (hexanes: ethyl acetate (5:1, v/v) to ethyl acetate (100%) gradient elution). mp = 194 – 195 °C. ^{1}H NMR (600 MHz, $CDCl_3$): δ = 8.18 (d, *J* = 8.4 Hz, 2H), 7.62 (d, *J* = 8.4 Hz, 2H), 5.42 (s, br, 1H), 5.14 (s, br, 1H), 4.10 (s, br, 1H), 3.67 (s, 3H), 2.06 – 2.00 (m, 1H), 1.93 (s, 3H), 1.82 – 1.74 (m, 1H), 1.67 – 1.58 (m, 1H), 1.46 (s, 3H), 1.38 (s, 3H), 1.36 – 1.28 (m, 4H), 1.16 (s, 3H), 1.11 (s, 3H), 0.59 (s, 3H). ^{13}C{^{1}H} NMR (150 MHz, $CDCl_3$): δ = 176.3, 168.9, 147.2, 131.2, 122.1, 87.3, 51.8, 47.4, 46.6, 45.7, 40.7, 33.8, 23.8, 23.3, 20.9, 20.8, 20.6. FTIR (cm^{-1}): 3065, 2952, 2866, 1649, 1622, 1593, 1519, 1484, 1345, 1106, 855, 758. HRMS (ESI-MS) *m/z* calcd for $C_{23}H_{35}N_3O_6$ $[M - H]^-$ 448.2453 found 448.2459.

XI. References

1. Gottlieb, H. E.; Kotlyar, V.; Nudelman, A. NMR Chemical Shifts of Common Laboratory Solvents as Trace Impurities. *J. Org. Chem.* **1997**, *62*, 7512-7515.

2. Fulmer, G. R; Miller, A. J; Sherden, N. H.; Gottlieb, H. E.; Nudelman, A.; Stoltz, B. M.; Bercaw, J. E.; Goldberg, K. I. NMR Chemical Shifts of Trace Impurities: Common Laboratory Solvents, Organics, and Gases in Deuterated Solvents Relevant to the Organometallic Chemist. *Organometallics*. **2010**, *29*, 2176-2179.

3. Kurita, T.; Hattori, K.; Seki, S.; Mizumoto, T.; Aoki, F.; Yamada, Y.; Ikawa, K.; Maegawa, T.; Monguchi, Y.; Sajiki, H. Efficient and Convenient Heterogeneous Palladium-Catalyzed Regioselective Deuteration at the Benzylic Position. *Chem. Eur. J.* **2008**, *14*, 664-673.

4. Hansch, C.; Leo, A.; Taft, W. A Survey of Hammett Substituent Constants and Resonance and Field Parameters. *Chem. Rev.* **1991**, *91*, 165-195.

5. Liu, R.; Yang, Z.; Ni, Y.; Song, K.; Shen, K.; Lin, S.; Pan, Q. Pd(II)/Bipyridine-Catalyzed Conjugate Addition of Arylboronic Acids to a,ß-Unsaturated Carboxylic Acids. Synthesis of ß-Quaternary Carbons Substituted Carboxylic Acids. *J. Org. Chem.* **2017**, *82*, 8023-8030.

6. Carceller, E.; Salas, J.; Merlos, M.; Giral, M.; Ferrando, R.; Escamilla, I.; Ramis, J.; Garcia-Rafanell, J.; Forn, J. Novel Azo Derivatives as Prodrugs of 5-Aminosalicylic Acid and Amino Derivatives with Potent Platelet Activating Factor Antagonist Activity. *J. Med. Chem.* **2001**, *44*, 3001-3013.

7. Tan, X.; Liu, Z.; Shen, H.; Zhang, P.; Zhang, Z.; Li, C. Silver-Catalyzed Decarboxylative Trifluoromethylation of Aliphatic Carboxylic Acids. *J. Am. Chem. Soc.* **2017**, *139*, 12430-12433.

8. Giri, R.; Maugel, N.; Li, J.-J.; Wang, D.-H.; Breazzano, S.; Saunders, L.; Yu, J.-Q. Palladium-Catalyzed Methylation and Arylation of sp^2 and sp^3 C−H Bonds in Simple Carboxylic Acids. *J. Am. Chem. Soc.* **2007**, *129*, 3510-3511.

9. Rit, R.; Yadav, R.; Sahoo, A. Pd(II)-Catalyzed Primary-C(sp^3)-H Acyloxylation at Room Temperature. *Org. Lett.* **2012**, *14*, 3724-3727.

10. Zhang, C.-P.; Wang, Z.-L.; Chen, Q.-Y.; Zhang, C.-T.; Gu, Y.-C.; Xiao, J.-C. Generation of the CF_3 Radical from Trifluoromethylsulfonium triflate and Its Trifluoromethylation of Styrenes. *Chem. Comm.* **2011**, *47*, 6632-6634.

11. Trusova, M.; Rodriguez-Zubiri, M.; Kutonova, K.; Jung, N.; Bräse, S.; Felpin, F.-X.; Postnikov, P. Ultra-fast Suzuki and Heck Reactions for the Synthesis of Styrenes and Stilbenes using Arenediazonium Salts as Super-electrophiles. *Org. Chem. Front.* **2018**, *5*, 41-45.

12. Denmark, S.; Butler, C. Vinylation of Aryl Bromides Using an Inexpensive Vinylpolysiloxane. *Org. Lett.* **2006**, *8*, 63-66.

13. Cabre, A.; Sciortino, G.; Ujaque, G.; Verdaguer, X.; Lledos, A.; Riera, A. Iridium-Catalyzed Isomerization of N-Sulfonyl Aziridines to Allyl Amines. *Org. Lett.* **2018**, *20*, 5747-5751.

14. Sore, H.; Blackwell, D.; MacDonald, S.; Spring, D. Diversity-Oriented Synthesis of Disubstituted Alkenes using Masked Silanols. *Org. Lett.* **2010**, *12*, 2806-2809.

15. Lamaa, D.; Messe, E.; Gandon, V.; Alami, M.; Hamze, A. Towards a Greener Barluenga-Valdés Cross-Coupling: Microwave Promoted C-C Bond Formation with a Pd/PEG/H_2O Recyclable Catalytic System. *Org. Lett.* **2019**, *21*, 8708-8712.

16. Rong, G.; Liu, D.; Lu, L.; Yan, H.; Zheng, Y.; Chen, J.; Mao, J. Iron-Catalyzed Decarboxylative Methylation of α,β-Unsaturated Acids under Ligand-Free Conditions. *Tetrahedron*, **2014**, *70*, 5033-5037.

17. Panda, S.; Coffin, A.; Nguyen, Q. N.; Tantillo, D. J.; Ready, J. M. Synthesis and Utility of Dihydropyridine Boronic Esters. *Angew. Chem. Int. Ed.* **2016**, *55*, 2205-2209.

18. Ortiz, L.; Khan, I. Synthesis of Optically Active Helical Poly(3-methyl-4-vinylpyridine). *Macromolecules*, **1998**, *31*, 5927-5929.

19. Manojveer, S.; Forrest, S.; Johnson, M. Ru-Catalyzed Completely Deoxygenative Coupling of 2-Arylethanols through Base-Induced Net Decarbonylation. *Chem. Eur. J.* **2018**, *24*, 803−807.

20. Gonzalez-de-Castro, A.; Xiao, J. Green and Efficient: Iron-Catalyzed Selective Oxidation of Olefins to Carbonyls with O_2. *J. Am. Chem. Soc.* **2015**, *137*, 8206-8218.

21. Wang, Y.-H.; Tian, J.-S.; Tan, P.-W.; Zhang, X.-X.; Cao, Z.-Y.; Zhou, F.; Wang, X.; Zhou, J. Regiodivergent Intramolecular Nucleophilic Addition of Ketimines for the Diverse Synthesis of Azacycles. *Angew. Chem. Int. Ed.* **2020**, *59*, 1634-1643.

22. Yang, K.; Zhou, F.; Kuang, Z.; Gao, G.; Driver, T.; Song, Q. Diborane-Mediated Deoxygenation of *o*-Nitrostyrenes To Form Indoles. *Org. Lett.* **2016**, *18*, 4088-4091.

23. Sandosham, J.; Undheim, K. Synthesis of Pyrimidinyl Triflates and Palladium-Catalyzed Coupling With Organotin and Organozinc Reagents. *Heterocycles*, **1994**, *37*, 501-514.

24. DiBerardino, T. Synthesis and Polymerization of 4-Vinylpyrimidine. *Journal of Polymer Science.* **1989**, *27*, 2483-2486.

25. Xiao, J.; Huang, Y.; Song, Z.; Feng, W. Facile Catalyst-free Synthesis of 2-Vinylquinolines via a Direct Deamination Reaction Occurring during Mannich Synthesis. *RSC Adv*. **2015**, *5*, 99095-99098.

26. Zhang, X.-W.; Jiang, G.-Q.; Lei, S.-H.; Shan, X.-H.; Qu, J.-P.; Kang, Y.-B. Iron-Catalyzed α,β-Dehydrogenation of Carbonyl Compounds. *Org. Lett.* **2021**, *23*, 1611-1615.

27. Lee, J.; Bae, D.; Park, J.; Jo, H.; Lee, E.; Rhee, Y.; Park, J. Concurrent Formation of N–H Imines and Carbonyl Compounds by Ruthenium-Catalyzed C–C Bond Cleavage of β-Hydroxy Azides. *Org. Lett.* **2020**, *22*, 4608-4613.

28. Dohi, T.; Takenaga, N.; Goto, A.; Maruyama, A.; Kita, Y. Direct Lactone Formation Using Hypervalent Iodine(III) Reagents with KBr via Selective C-H Abstraction Protocol. *Org. Lett.* **2007**, *9*, 3129-3132.

XII. ^{1}H and ^{13}C NMR Spectra of Staring Materials (1)

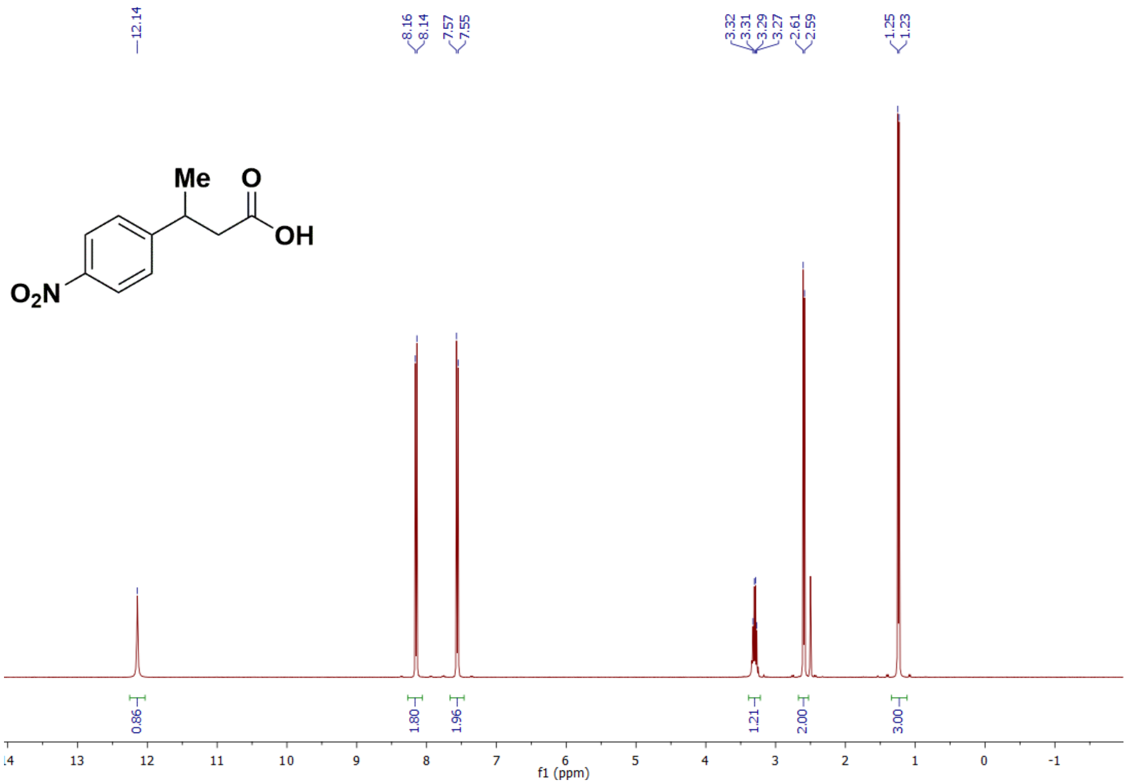

^{1}H NMR spectrum of **3-(4-nitrophenyl)butanoic acid (1d)** in DMSO-d_6 at 400 MHz

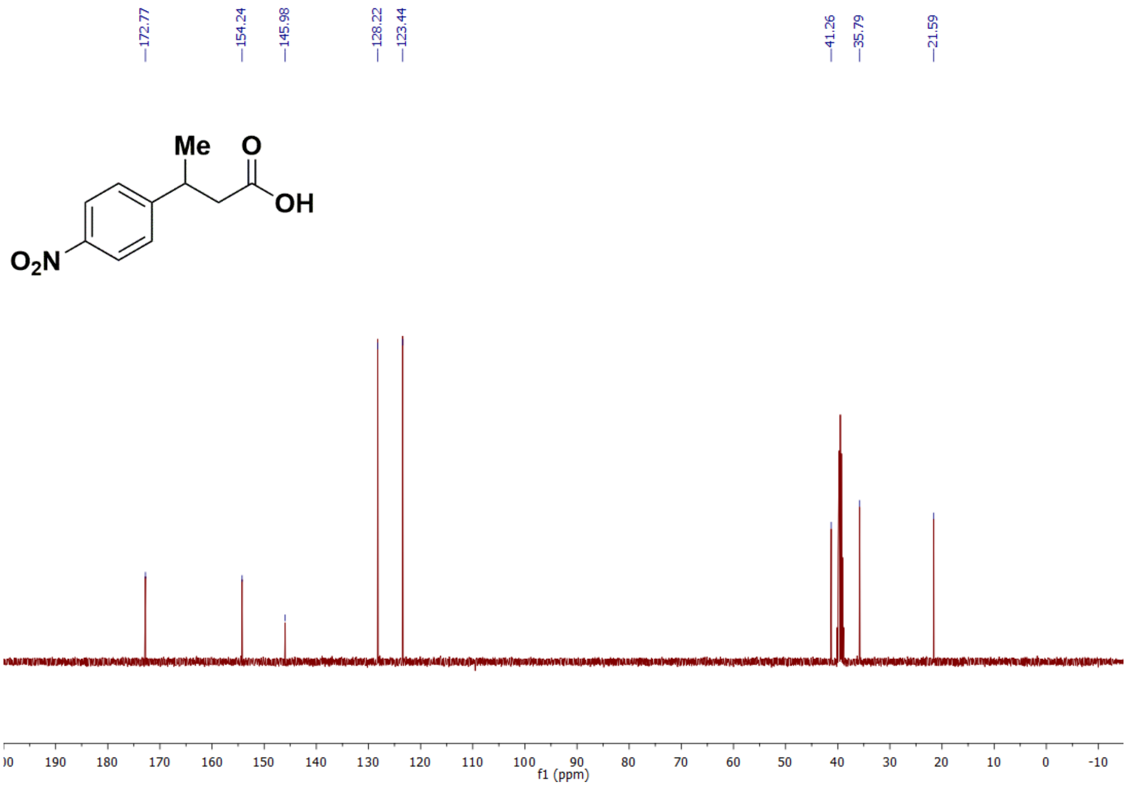

$^{13}C\{^{1}H\}$ NMR spectrum of **3-(4-nitrophenyl)butanoic acid (1d)** in DMSO-d_6 at 150 MHz

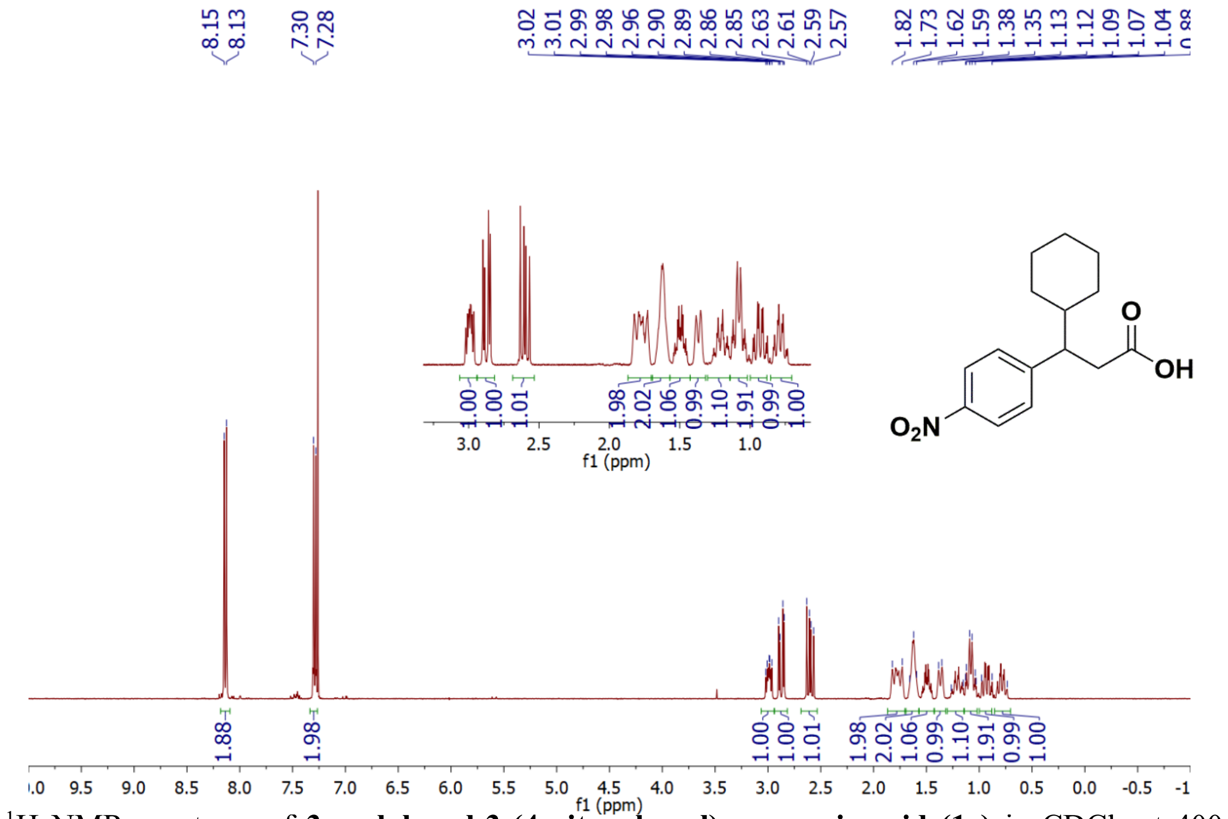

^{1}H NMR spectrum of **3-cyclohexyl-3-(4-nitrophenyl)propanoic acid (1e)** in $CDCl_3$ at 400 MHz

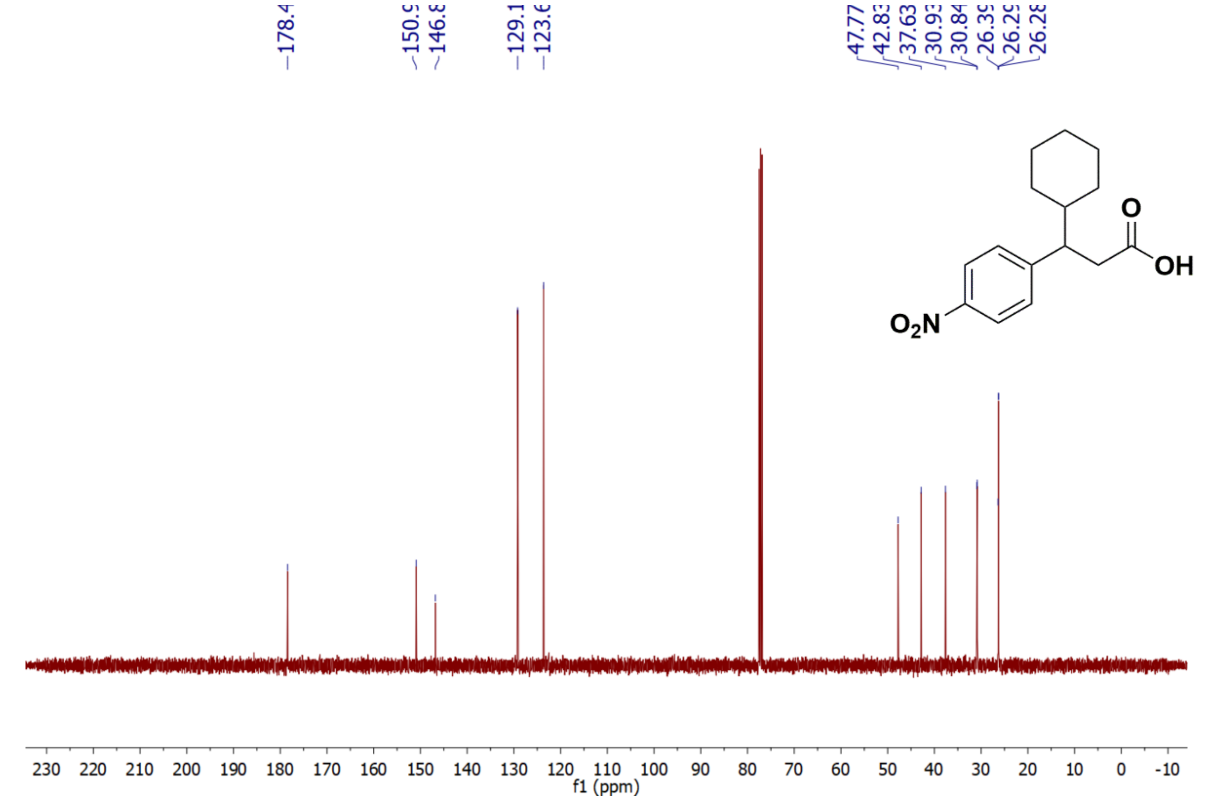

^{13}C{^{1}H}NMR spectrum of **3-cyclohexyl-3-(4-nitrophenyl)propanoic acid (1e)** in $CDCl_3$ at 150 MHz

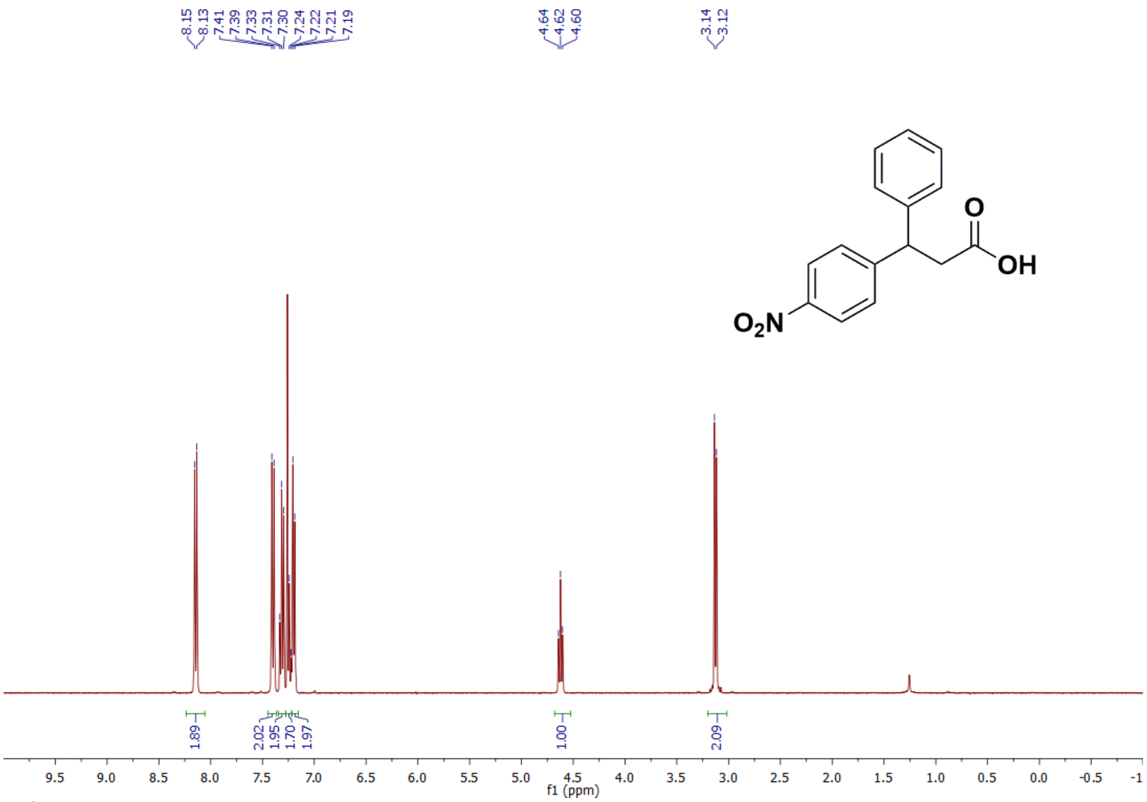

^{1}H NMR spectrum of **3-(4-nitrophenyl)-3-phenylpropanoic acid (1f)** in $CDCl_3$ at 400 MHz

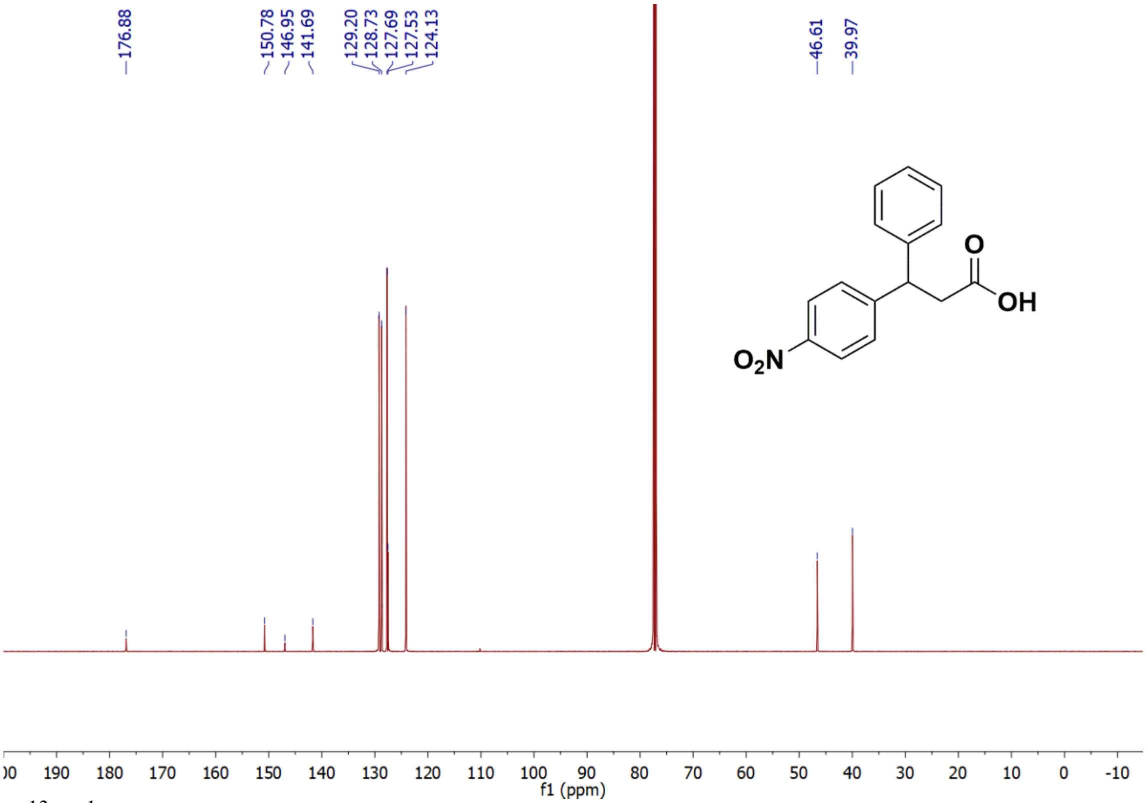

^{13}C{^{1}H} NMR spectrum of **3-(4-nitrophenyl)-3-phenylpropanoic acid (1f)** in $CDCl_3$ at 150 MHz

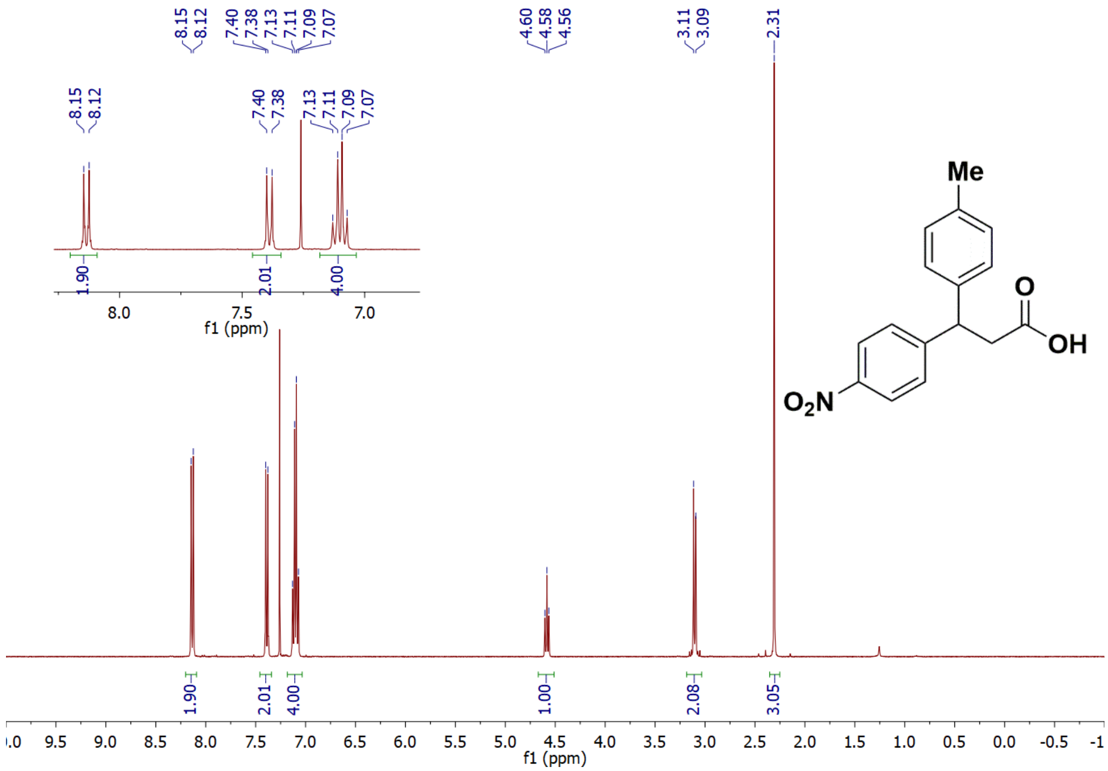

^{1}H NMR spectrum of **3-(4-methylphenyl)-3-(4-nitrophenyl)propanoic acid (1g)** in $CDCl_3$ at 400 MHz

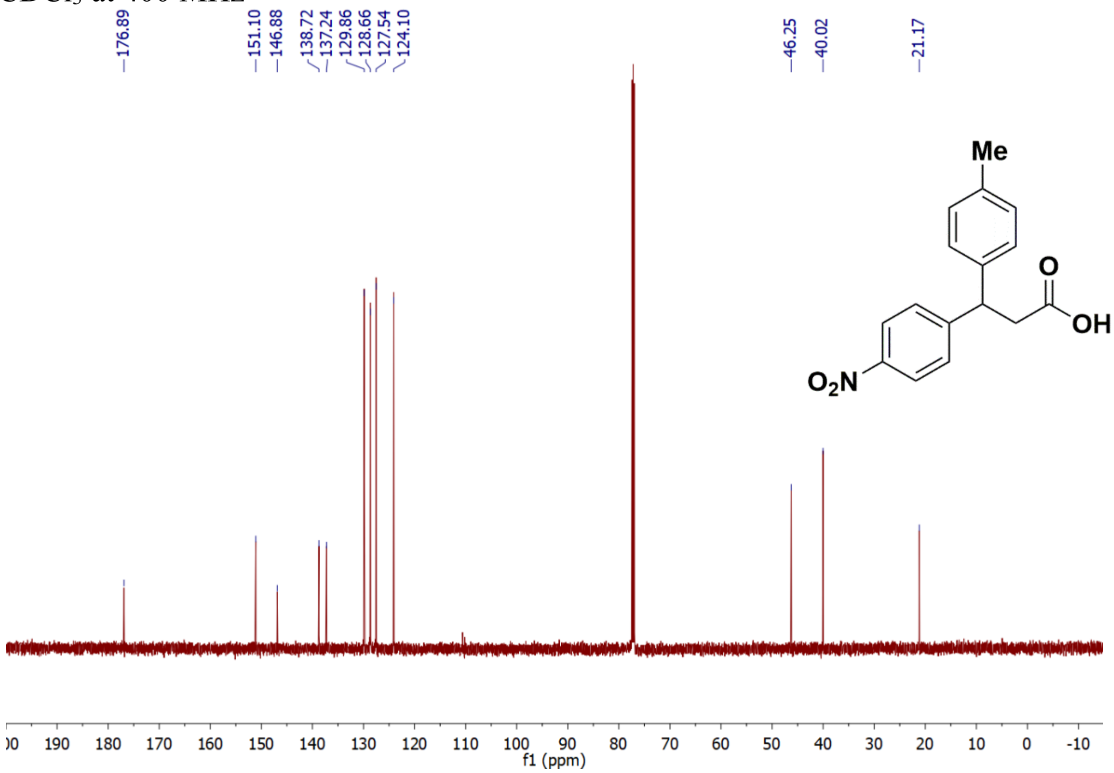

^{13}C{^{1}H} NMR spectrum of **3-(4-methylphenyl)-3-(4-nitrophenyl)propanoic acid (1g)** in $CDCl_3$ at 150 MHz

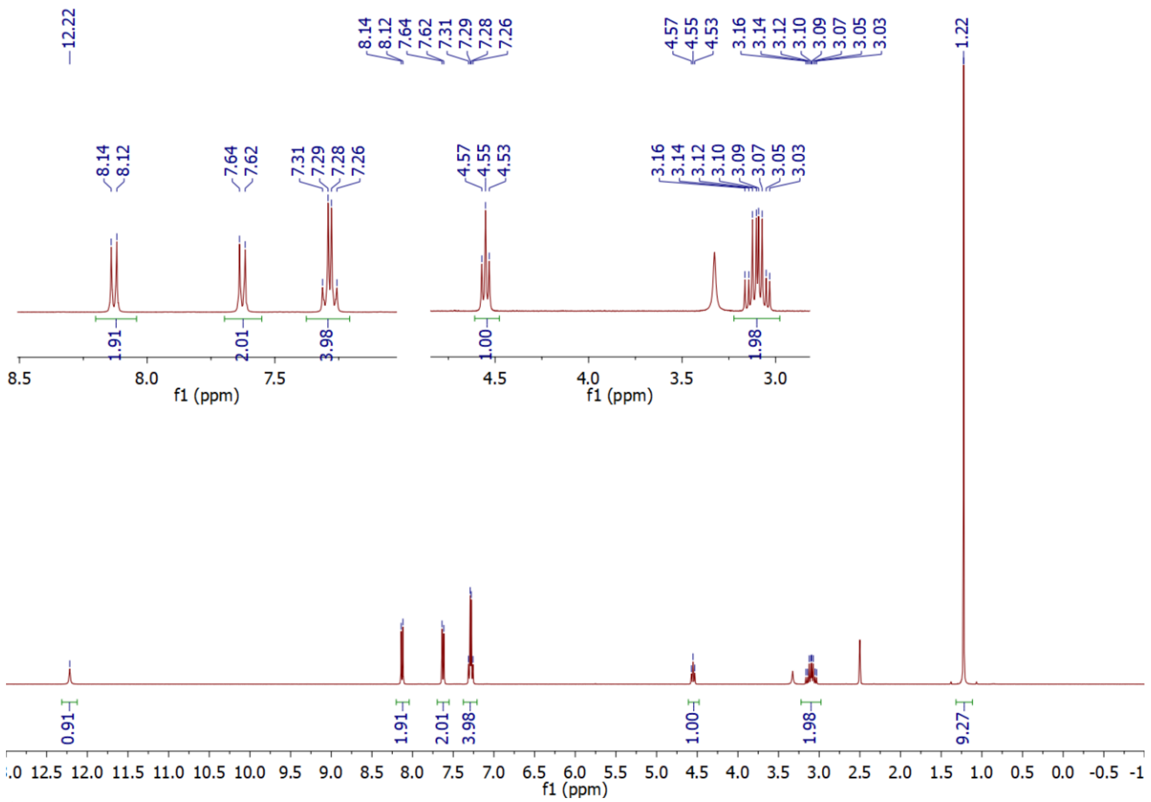

^{1}H NMR spectrum of **3-(4-nitrophenyl)-3-(4-*tert*-butylphenyl)propanoic acid (1h)** in DMSO-d_6 at 400 MHz

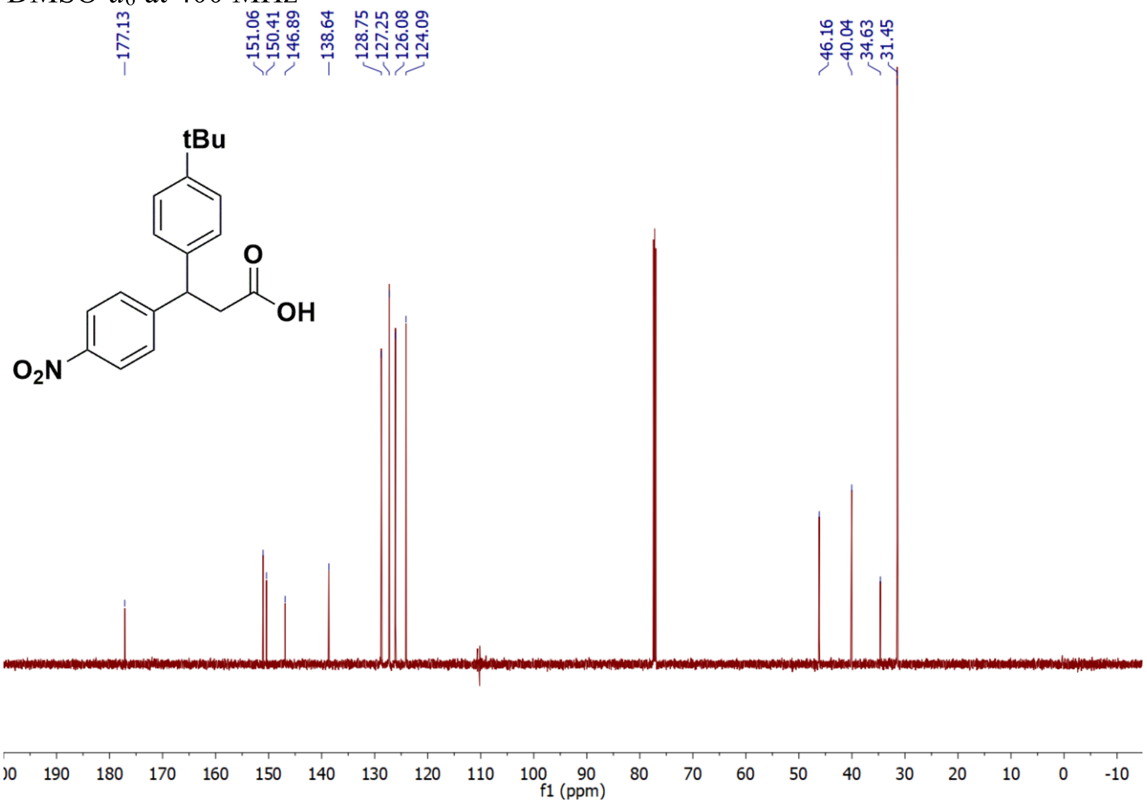

^{13}C{^{1}H} NMR spectrum of **3-(4-nitrophenyl)-3-(4-*tert*-butylphenyl)propanoic acid (1h)** in $CDCl_3$ at 150 MHz

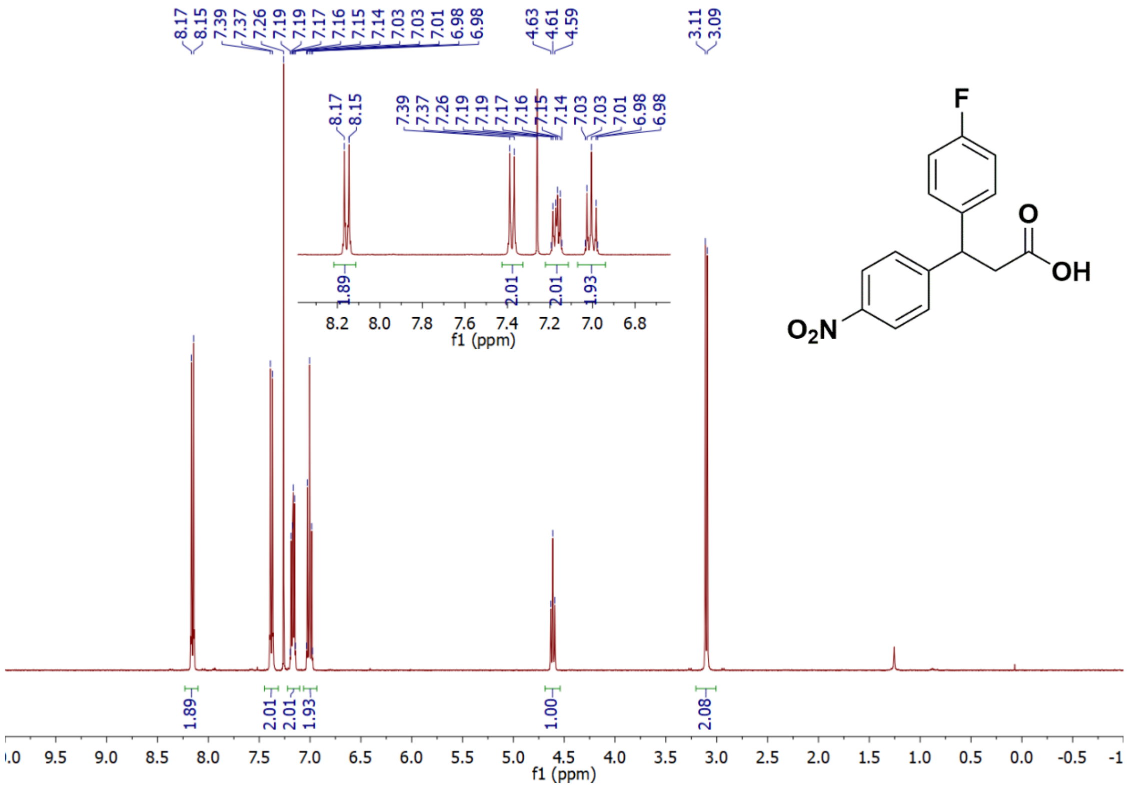

^{1}H NMR spectrum of **3-(4-fluorophenyl)-3-(4-nitrophenyl)propanoic acid (1i)** in $CDCl_3$ at 400 MHz

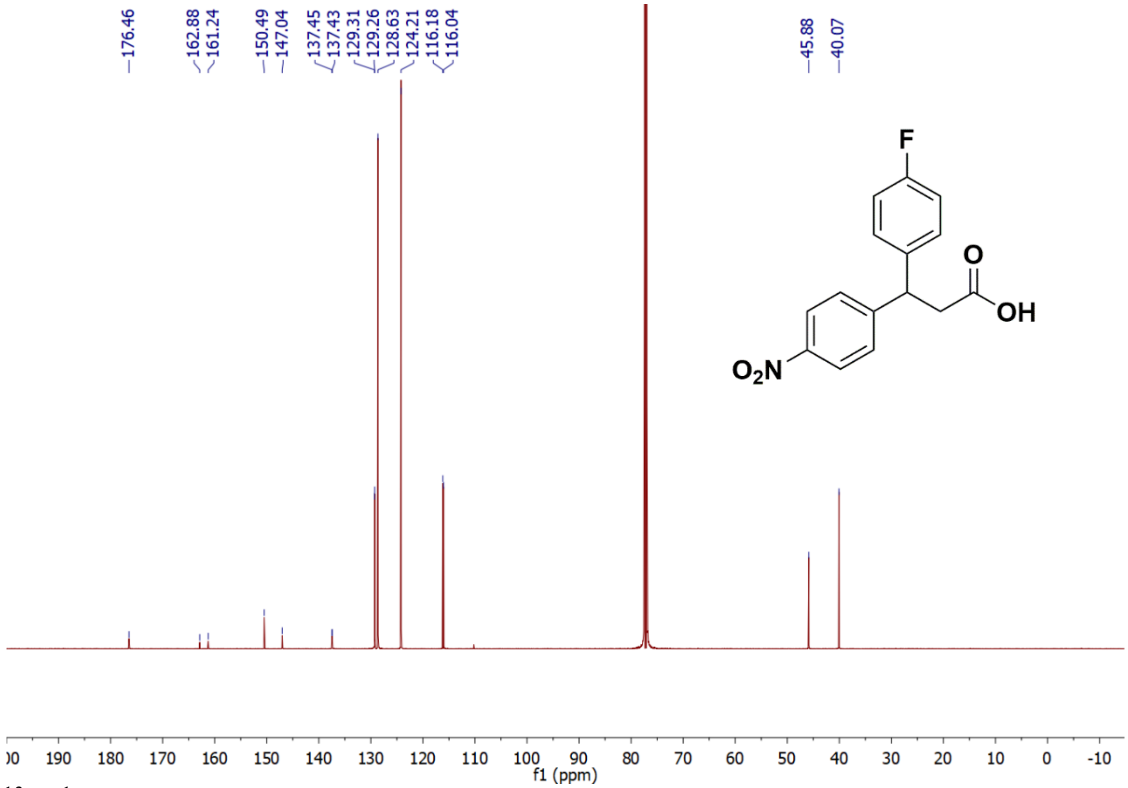

^{13}C{^{1}H} NMR spectrum of **3-(4-fluorophenyl)-3-(4-nitrophenyl)propanoic acid (1i)** in $CDCl_3$ at 150 MHz

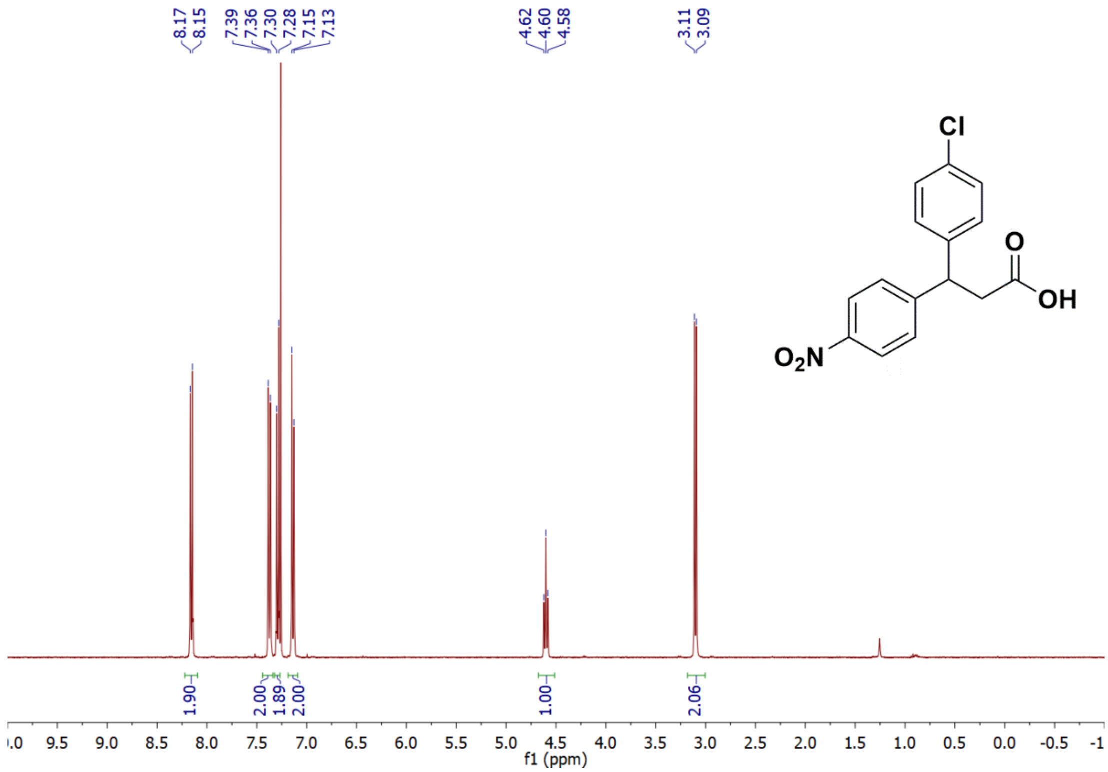

^{1}H NMR spectrum of **3-(4-chlorophenyl)-3-(4-nitrophenyl)propanoic acid (1j)** in $CDCl_3$ at 400 MHz

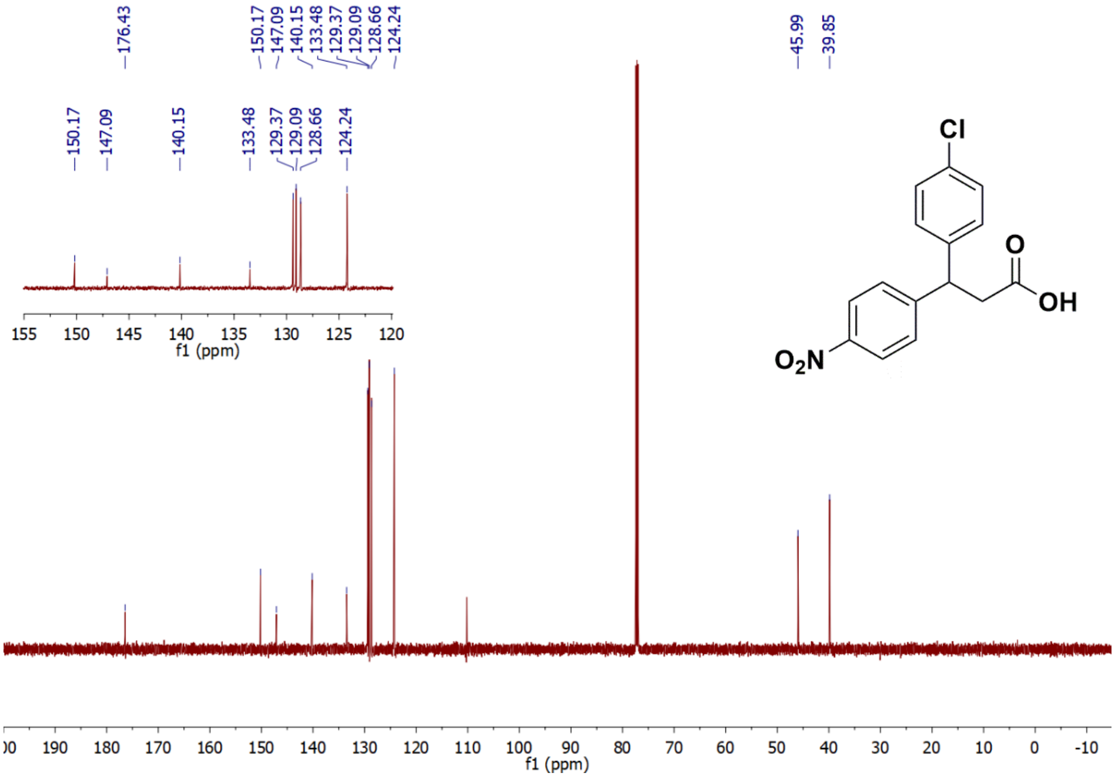

^{13}C{^{1}H} NMR spectrum of **3-(4-chlorophenyl)-3-(4-nitrophenyl)propanoic acid (1j)** in $CDCl_3$ at 150 MHz

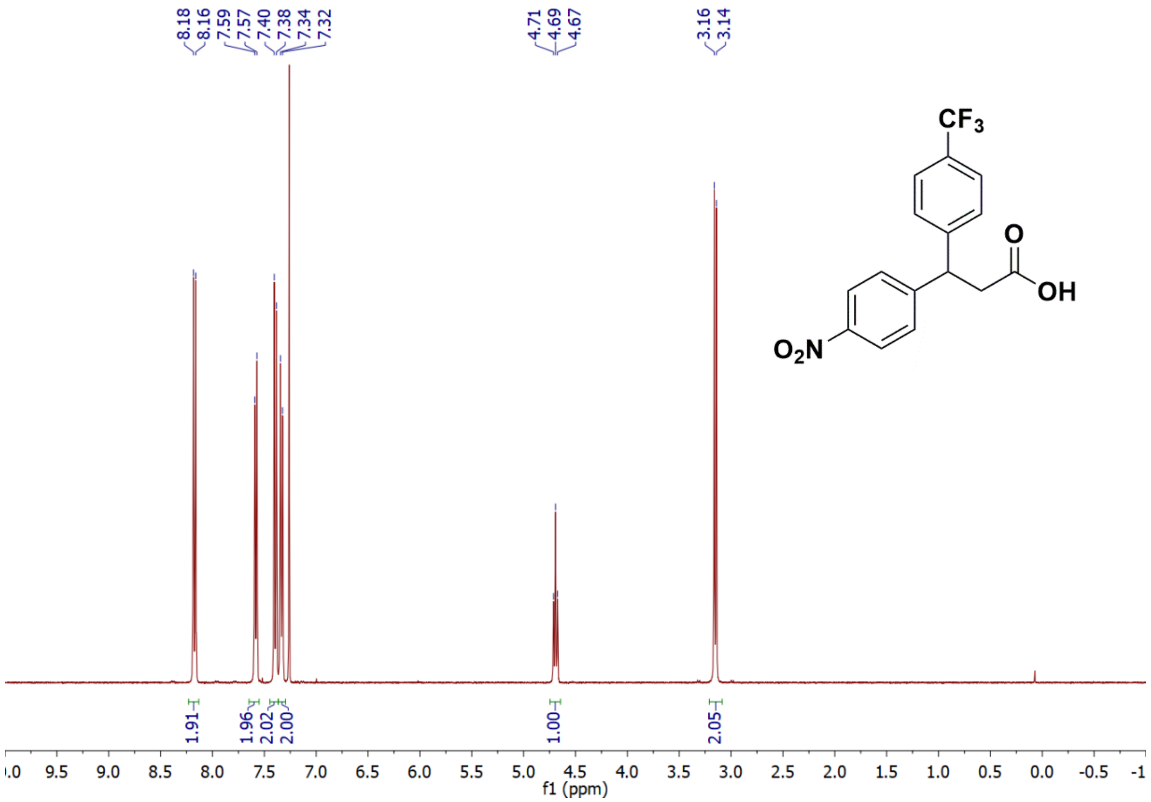

^{1}H NMR spectrum of **3-(4-nitrophenyl)-3-(4-trifluoromethylphenyl)propanoic acid (1k)** in $CDCl_3$ at 400 MHz

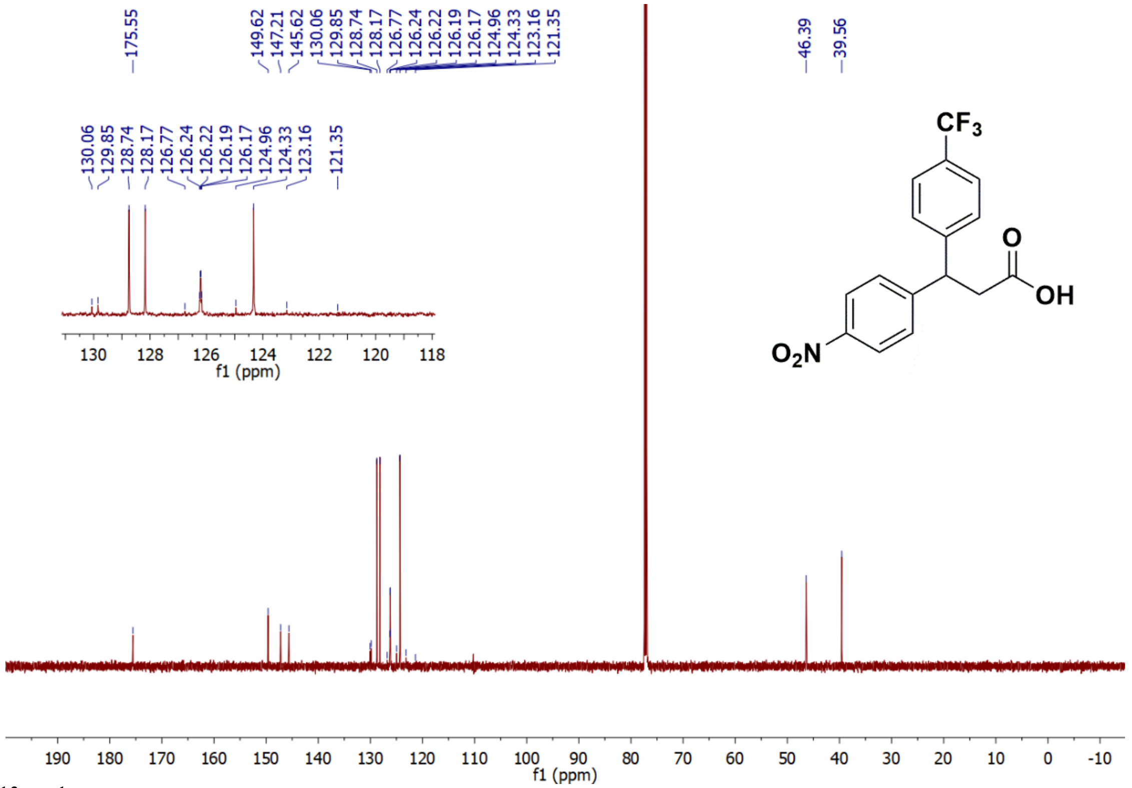

^{13}C{^{1}H} NMR spectrum of **3-(4-nitrophenyl)-3-(4-trifluoromethylphenyl)propanoic acid (1k)** in $CDCl_3$ at 150 MHz

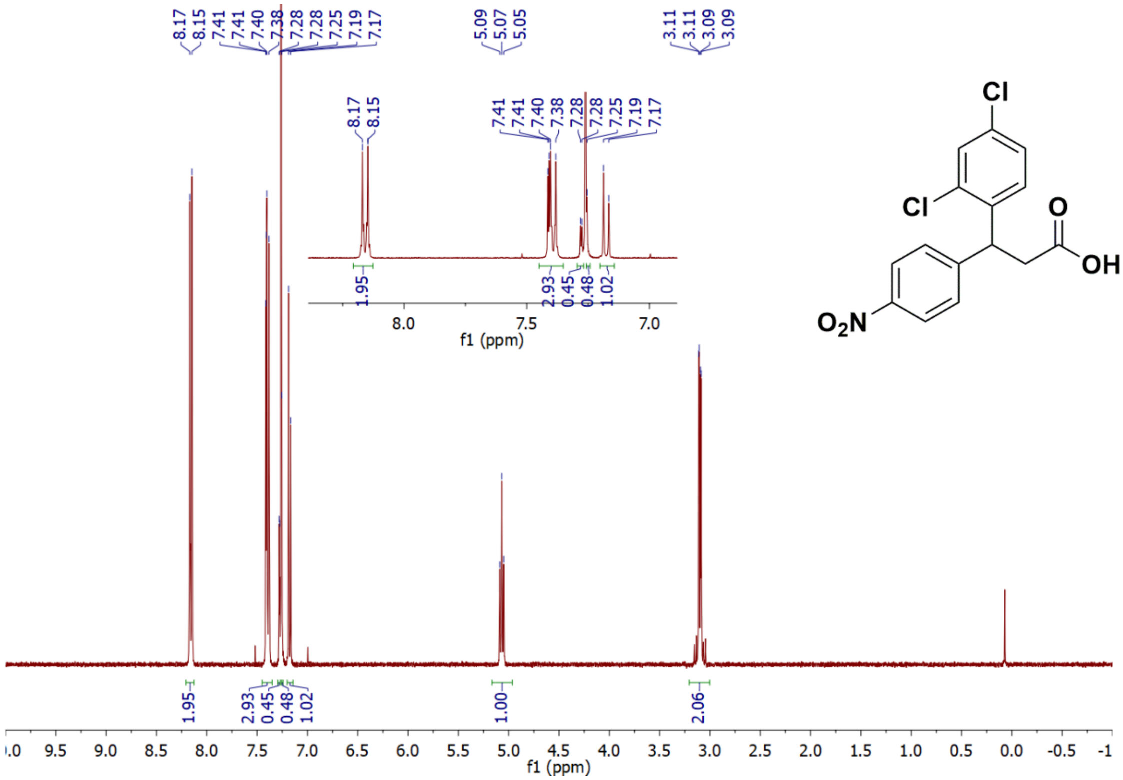

^{1}H NMR spectrum of **3-(2,4-dichlorophenyl)-3-(4-nitrophenyl)propanoic acid (1l)** in $CDCl_3$ at 400 MHz

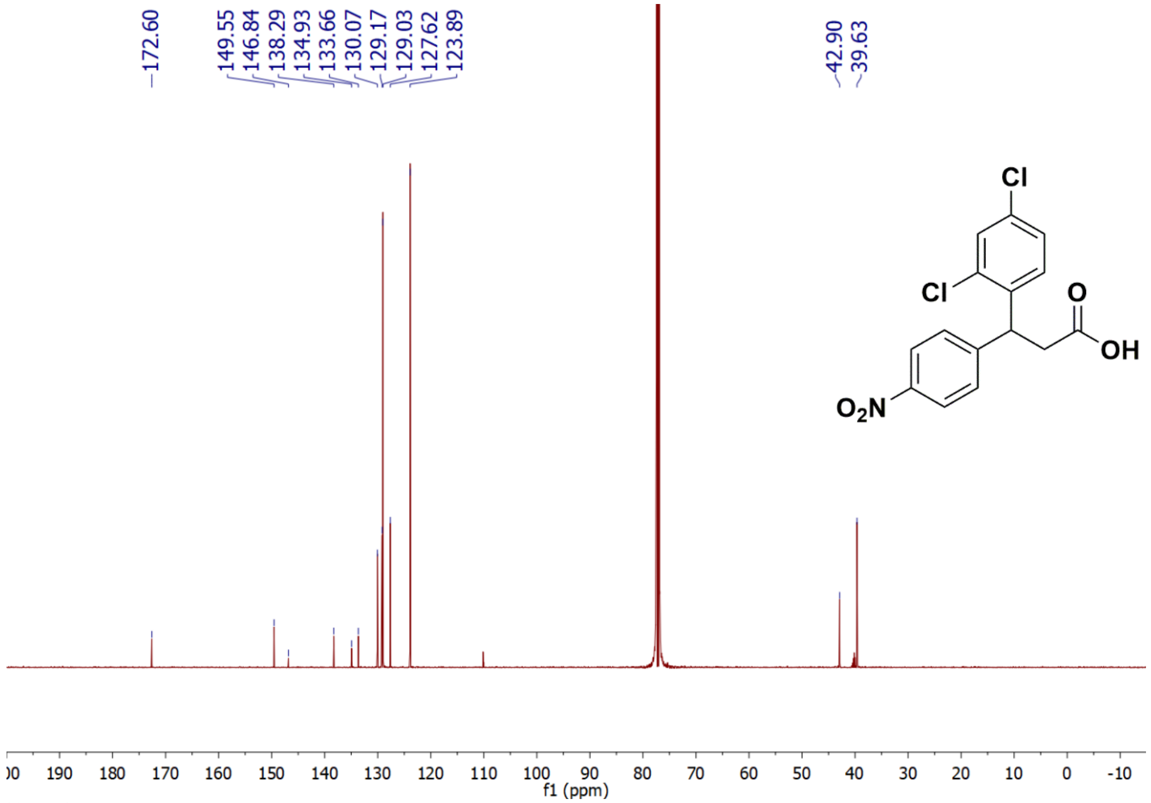

$^{13}C\{^{1}H\}$ NMR spectrum of **3-(2,4-dichlorophenyl)-3-(4-nitrophenyl)propanoic acid (1l)** in $CDCl_3$ + 2 drops DMSO-d_6 at 150 MHz

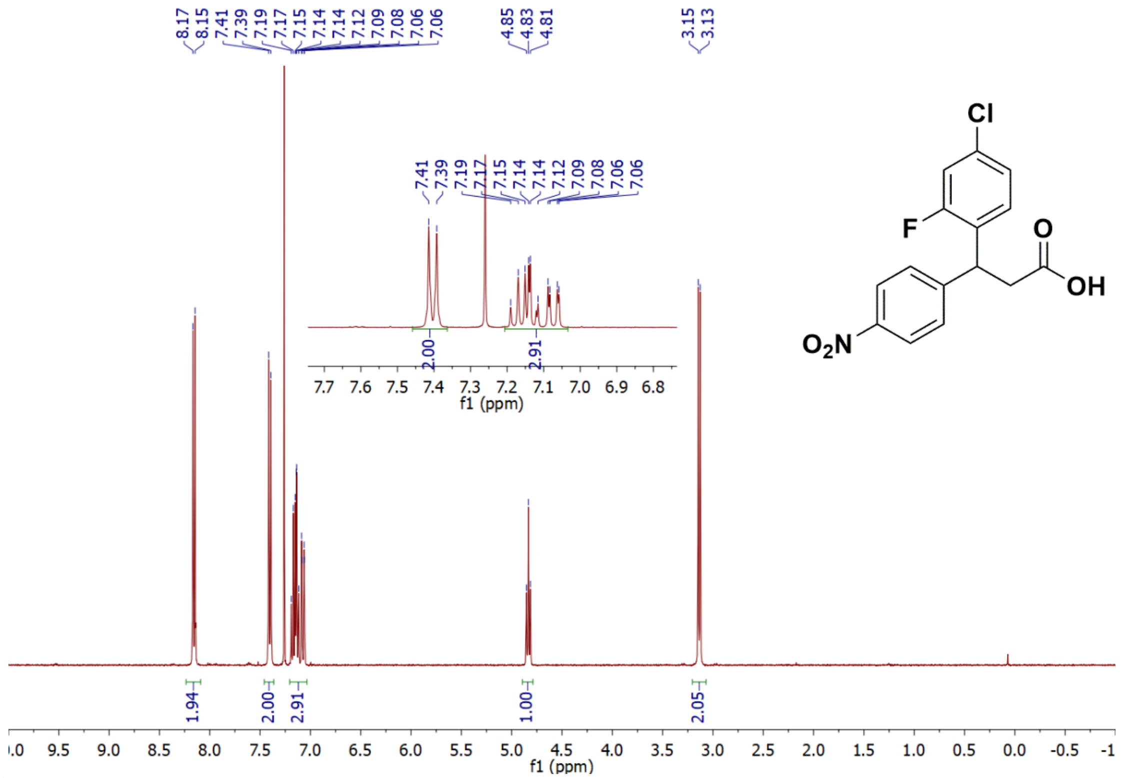

^{1}H NMR spectrum of **3-(2-fluoro-4-chlorophenyl)-3-(4-nitrophenyl)propanoic acid (1m)** in $CDCl_3$ at 400 MHz

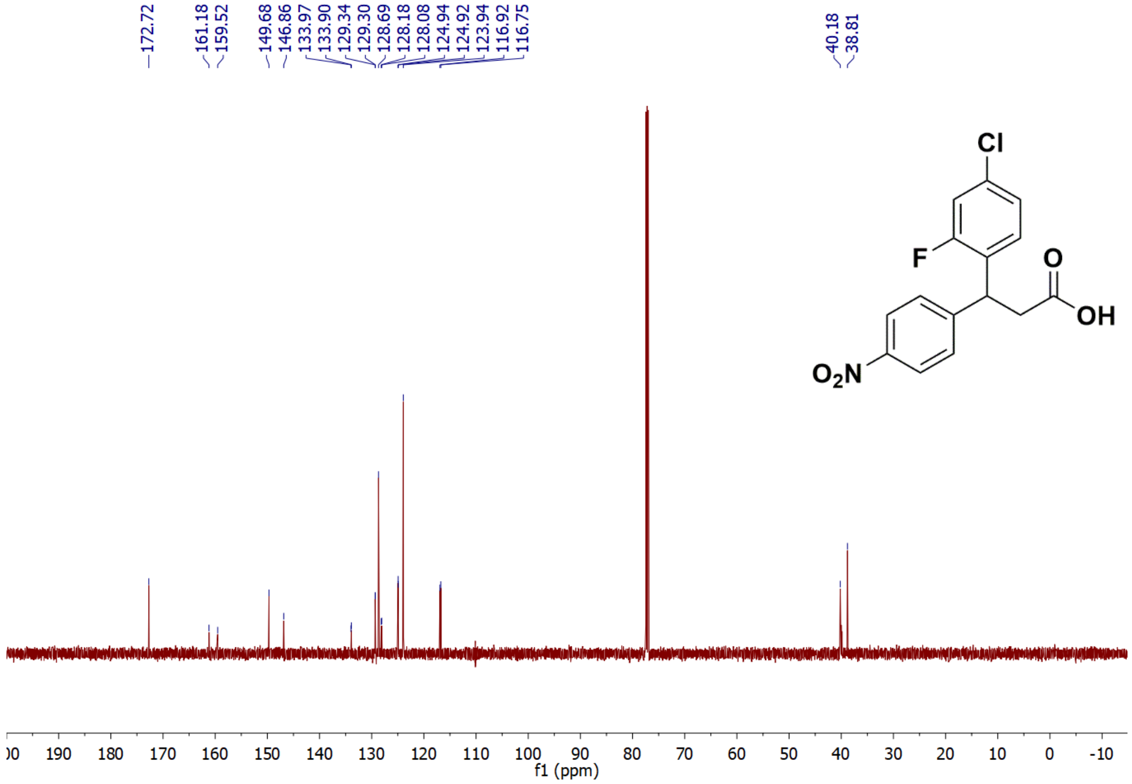

^{13}C{^{1}H} NMR spectrum of **3-(2-fluoro-4-chlorophenyl)-3-(4-nitrophenyl)propanoic acid (1m)** in $CDCl_3$ + 2 drops DMSO-d_6 at 150 MHz

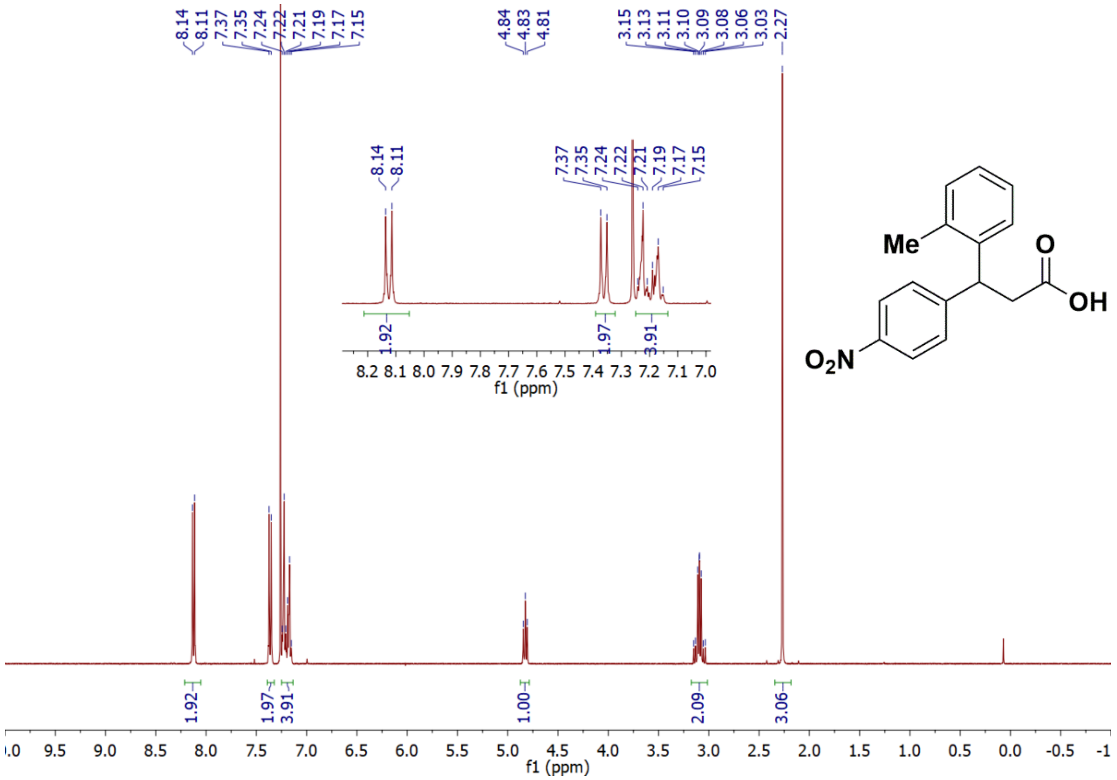

^{1}H NMR spectrum of **3-(2-methylphenyl)-3-(4-nitrophenyl)propanoic acid (1n)** in $CDCl_3$ at 400 MHz

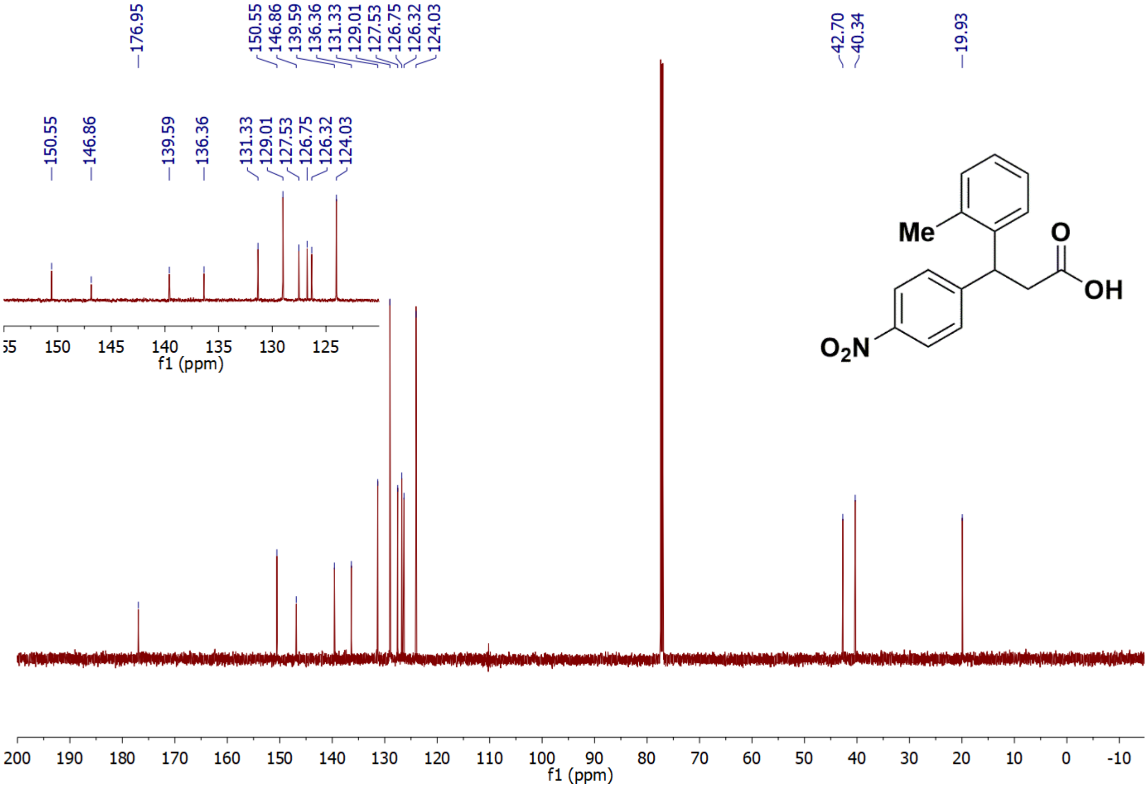

$^{13}C\{^{1}H\}$ NMR spectrum of **3-(2-methylphenyl)-3-(4-nitrophenyl)propanoic acid (1n)** in $CDCl_3$ at 150 MHz

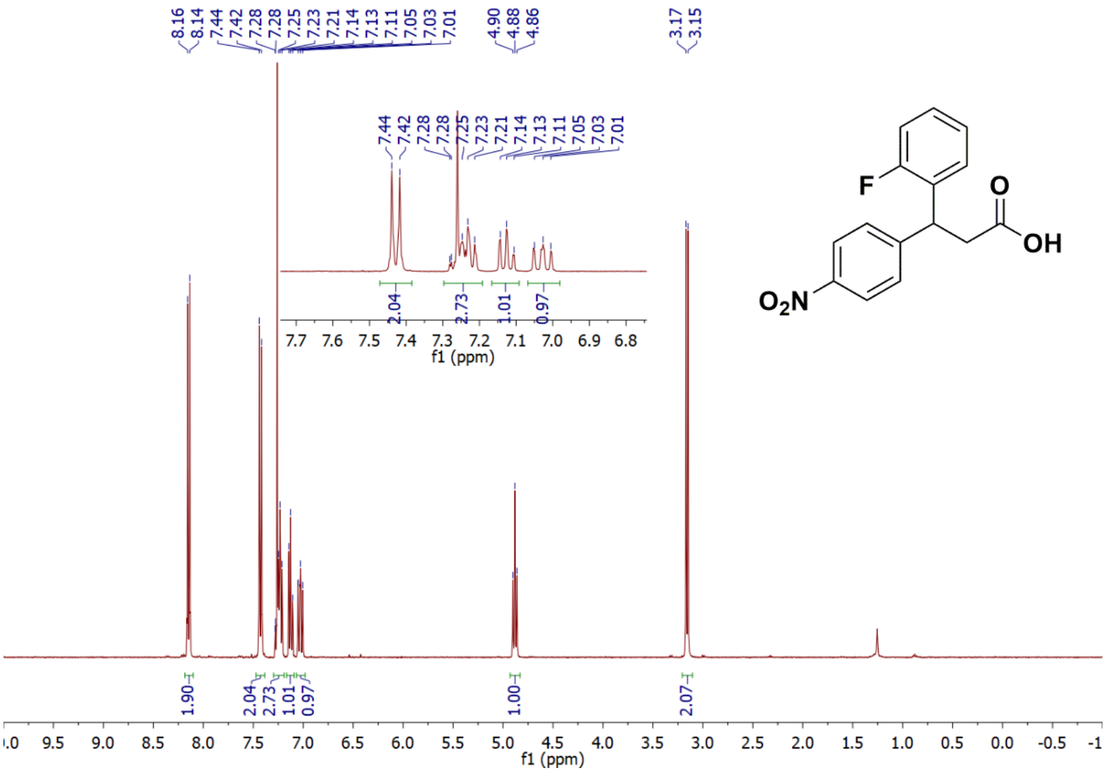

^{1}H NMR spectrum of **3-(2-fluorophenyl)-3-(4-nitrophenyl)propanoic acid (1o)** in $CDCl_3$ at 400 MHz

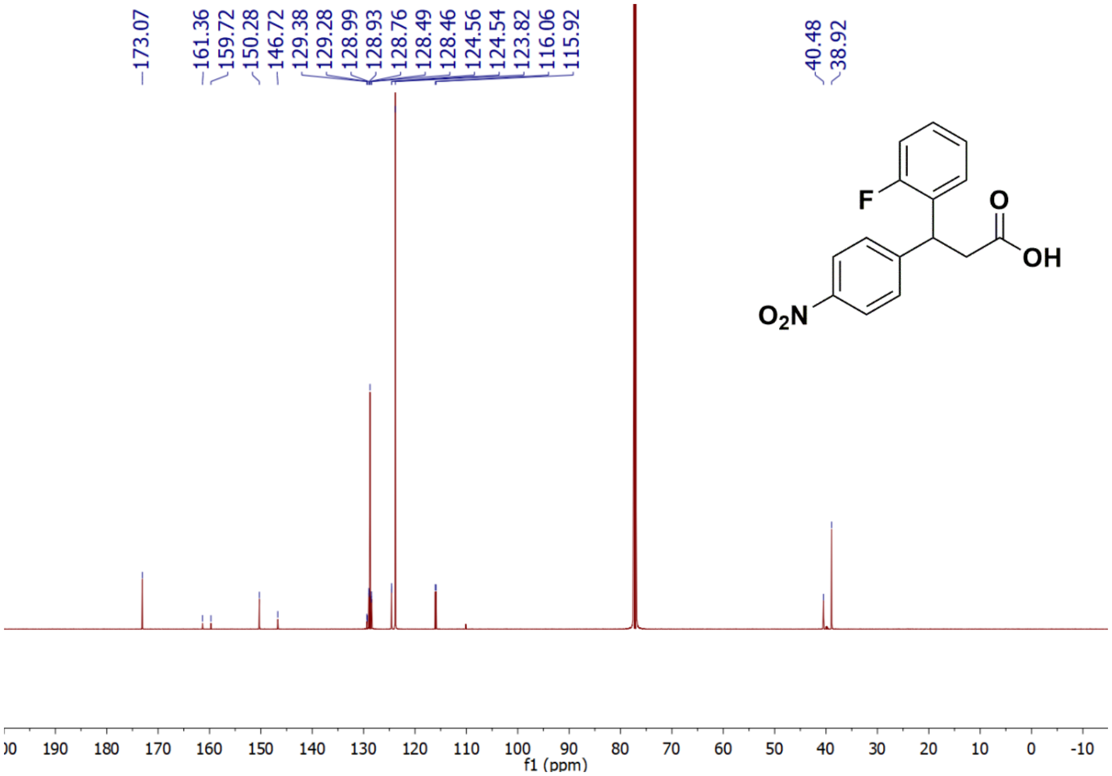

^{13}C{^{1}H} NMR spectrum of **3-(2-fluorophenyl)-3-(4-nitrophenyl)propanoic acid (1o)** in $CDCl_3$ + 2 drops DMSO-d_6 at 150 MHz

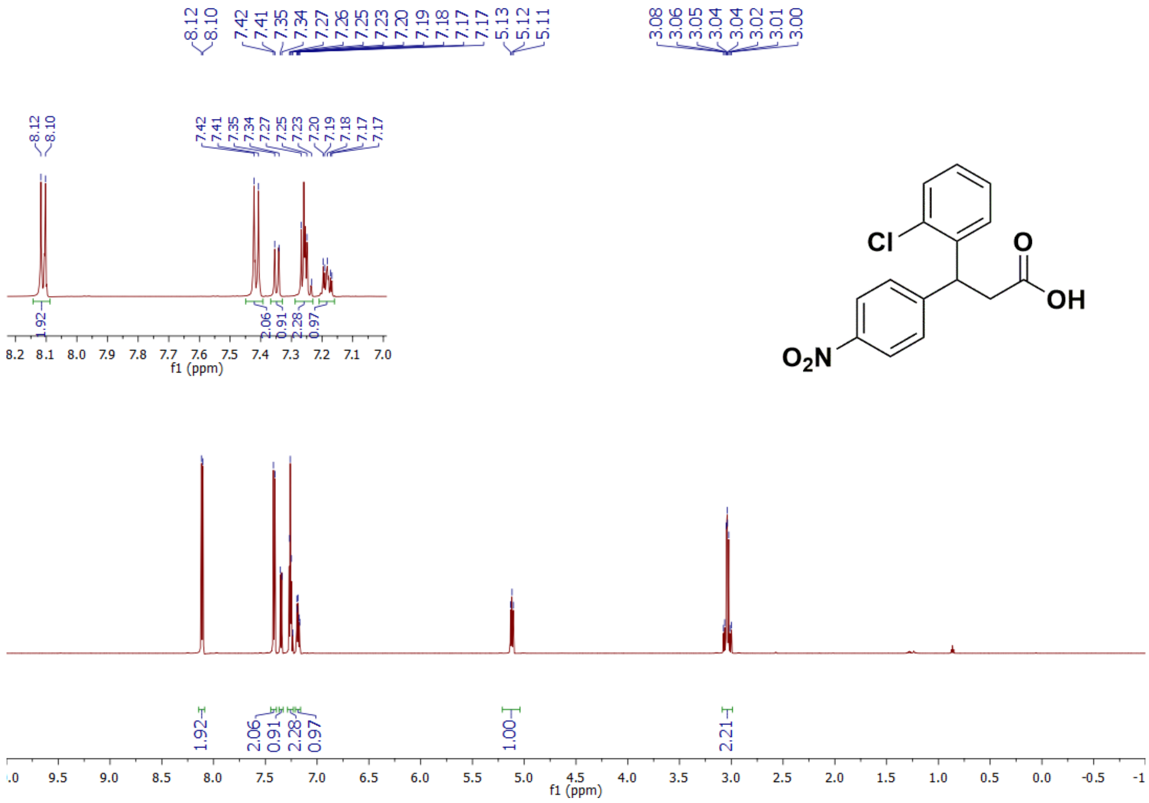

^{1}H NMR spectrum of **3-(2-chlorophenyl)-3-(4-nitrophenyl)propanoic acid (1p)** in $CDCl_3$ at 600 MHz

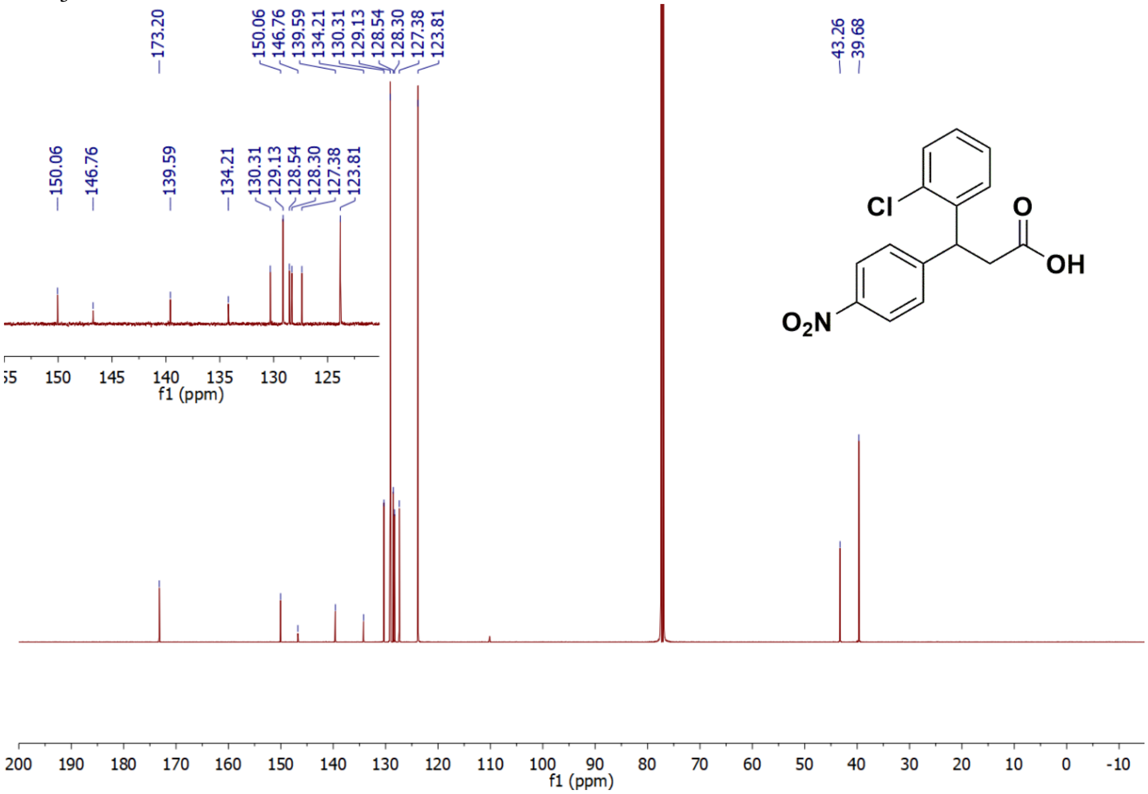

^{13}C{^{1}H} NMR spectrum of **3-(2-chlorophenyl)-3-(4-nitrophenyl)propanoic acid (1p)** in $CDCl_3$ at 150 MHz

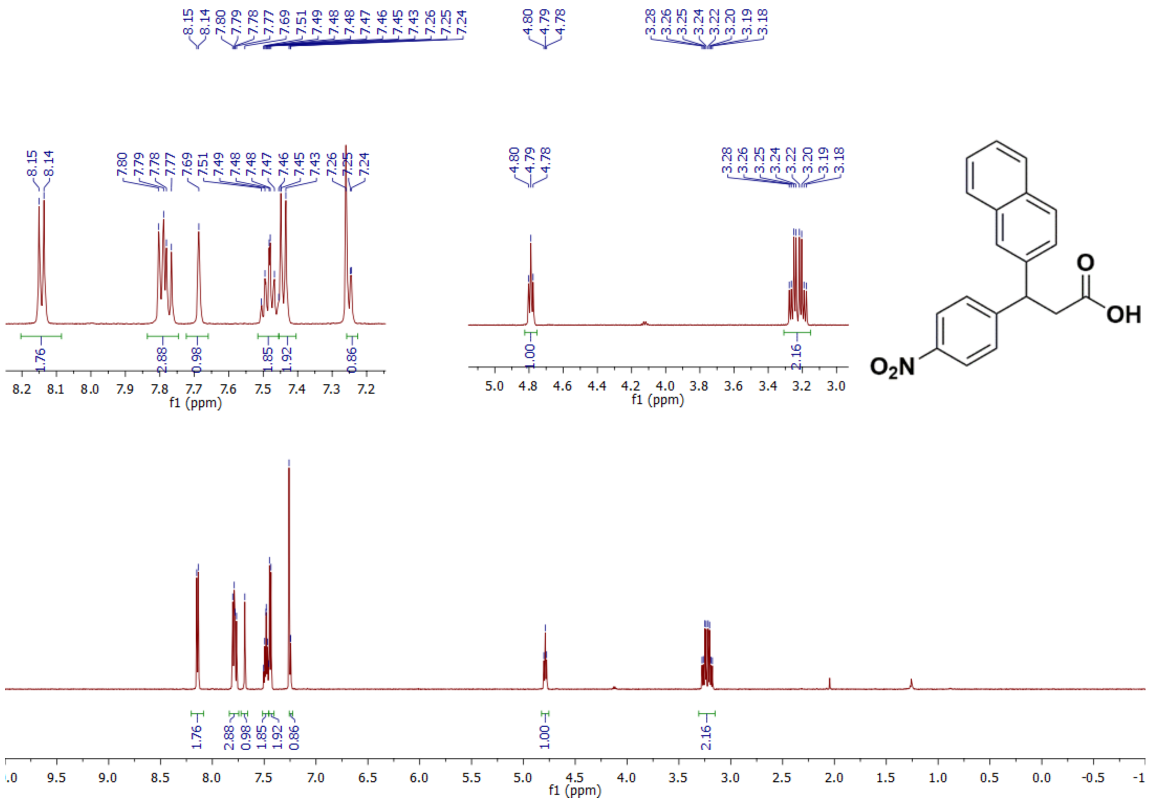

^{1}H NMR spectrum of **3-(4-naphthyl)-3-(4-nitrophenyl)propanoic acid (1q)** in $CDCl_3$ at 600 MHz

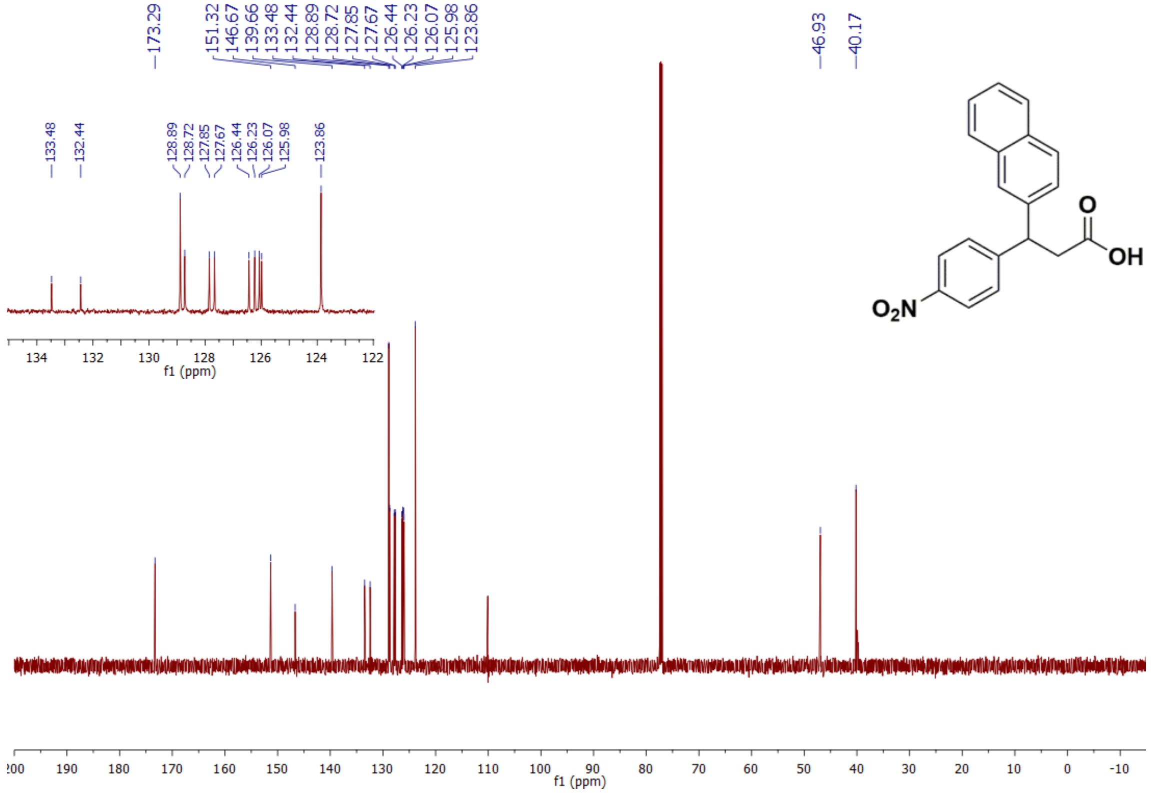

^{13}C{^{1}H} NMR spectrum of **3-(4-naphthyl)-3-(4-nitrophenyl)propanoic acid (1q)** in $CDCl_3$ + 2 drops DMSO-d_6 at 150 MHz

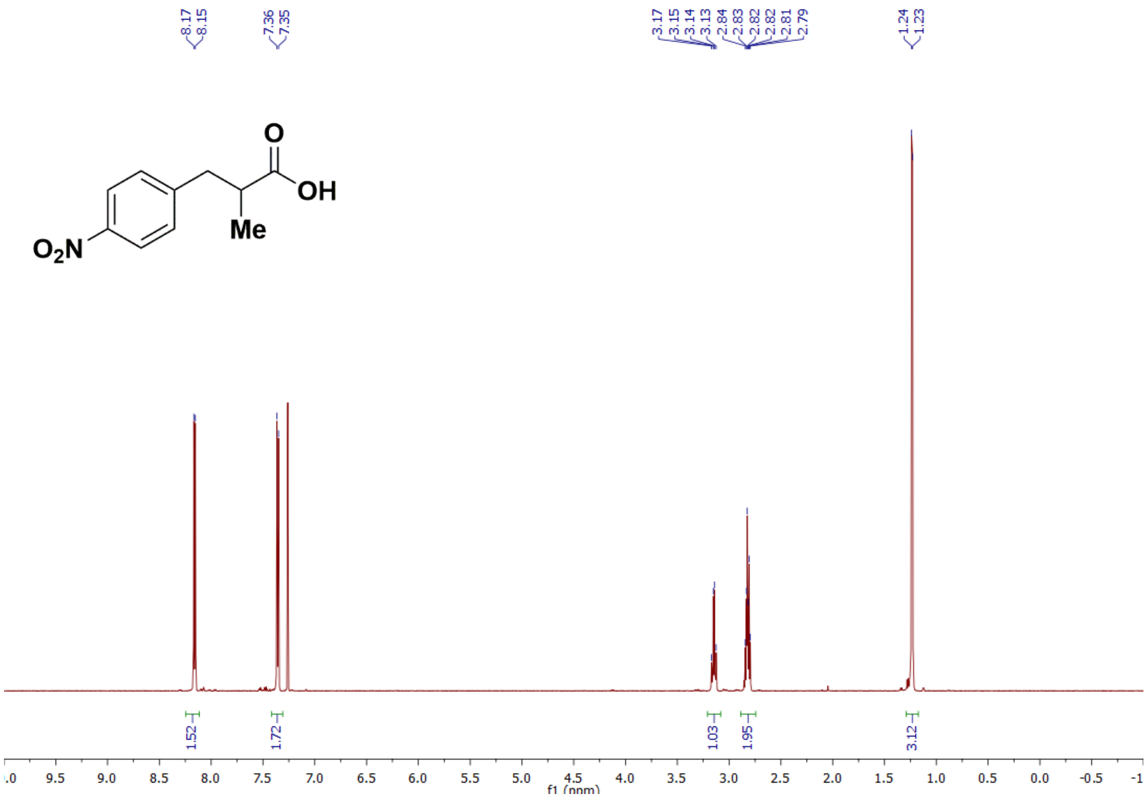

^{1}H NMR spectrum of **2-methyl-3-(4-nitrophenyl)propanoic acid (1r)** in $CDCl_3$ at 400 MHz

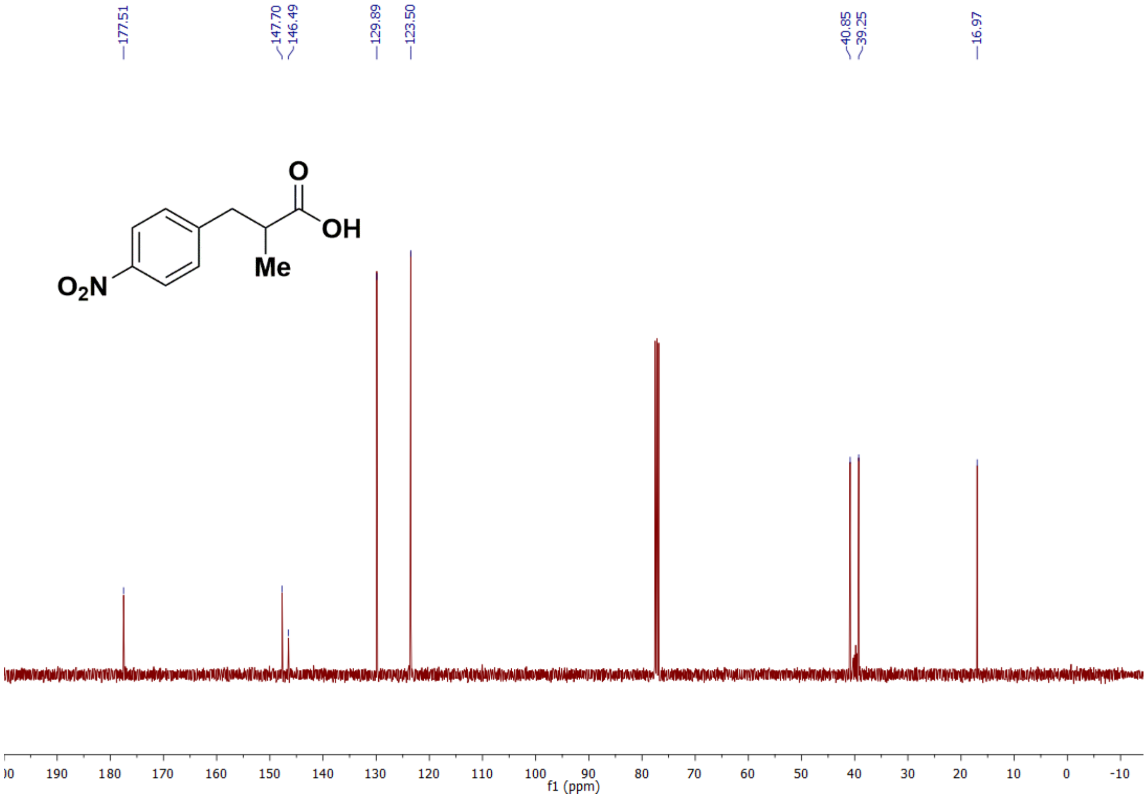

^{13}C{^{1}H} NMR spectrum of **2-methyl-3-(4-nitrophenyl)propanoic acid (1r)** in $CDCl_3$ + 1 drop DMSO-d_6 at 150 MHz

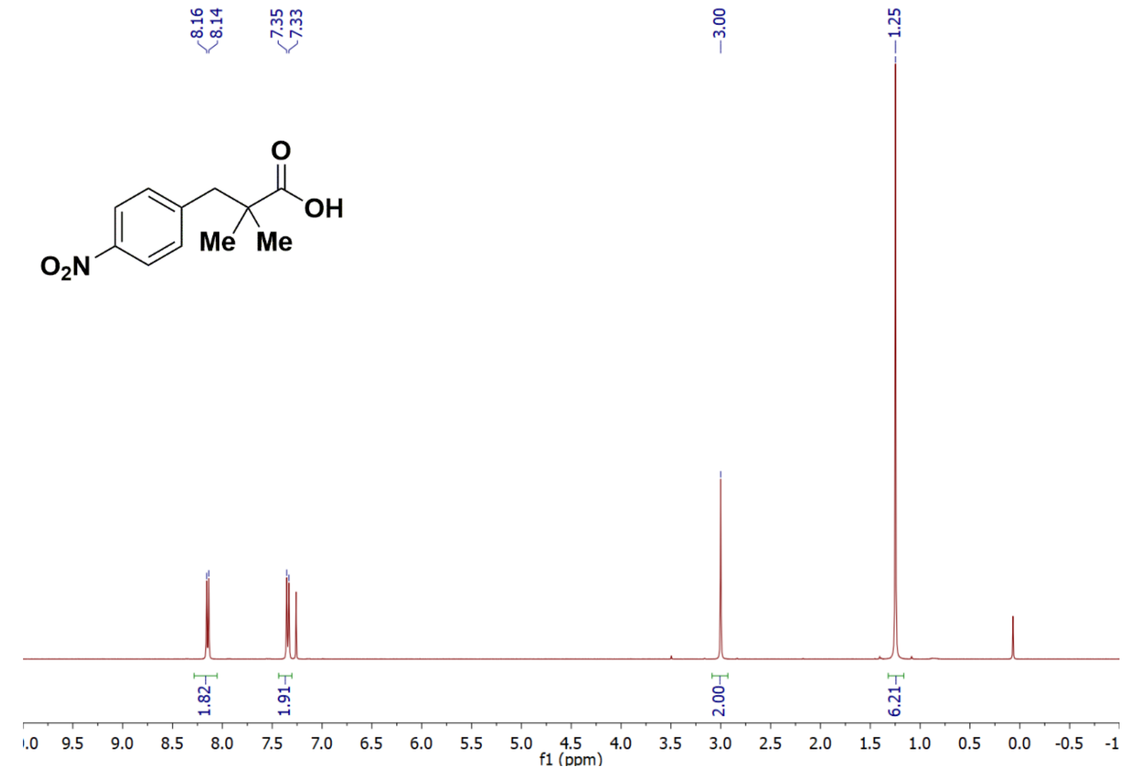

^{1}H NMR spectrum of **2,2-dimethyl-3-(4-nitrophenyl)propanoic acid (1ae)** in $CDCl_3$ at 600 MHz

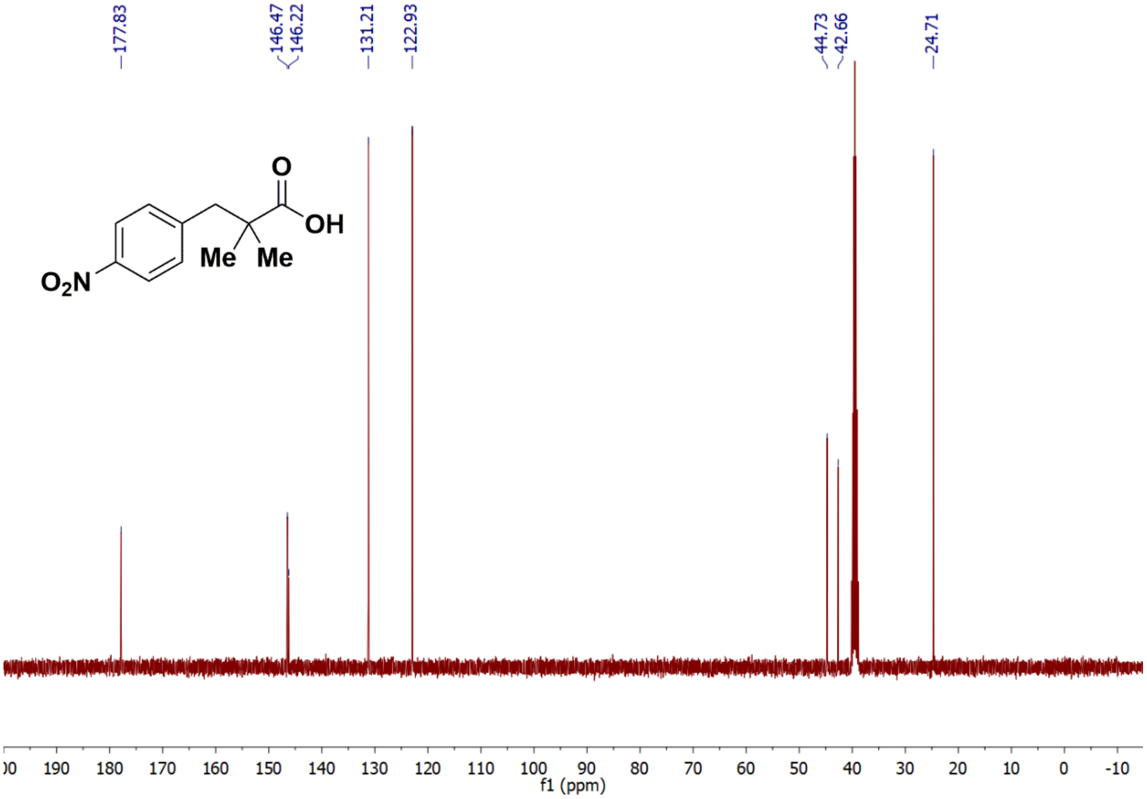

^{13}C{^{1}H} NMR spectrum of **2,2-dimethyl-3-(4-nitrophenyl)propanoic acid (1ae)** in DMSO-d_6 at 150 MHz

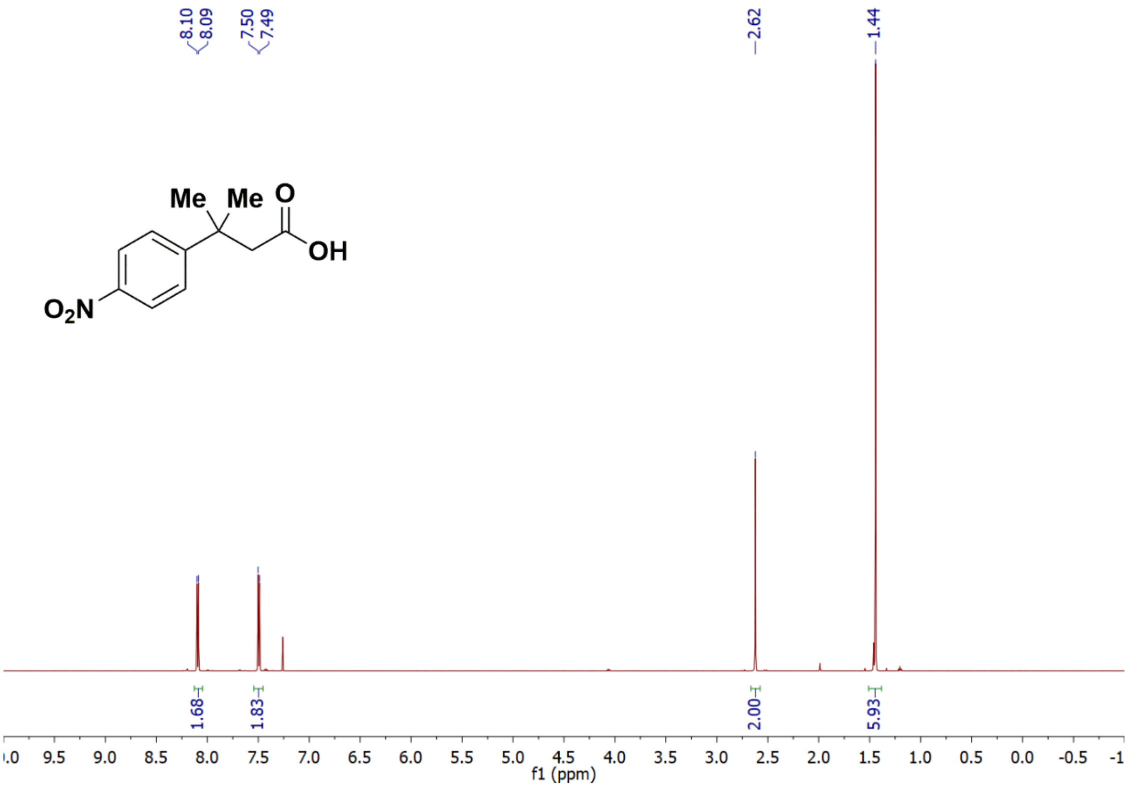

^{1}H NMR spectrum of **3-methyl-3-(4-nitrophenyl)butanoic acid (1af)** in $CDCl_3$ at 600 MHz

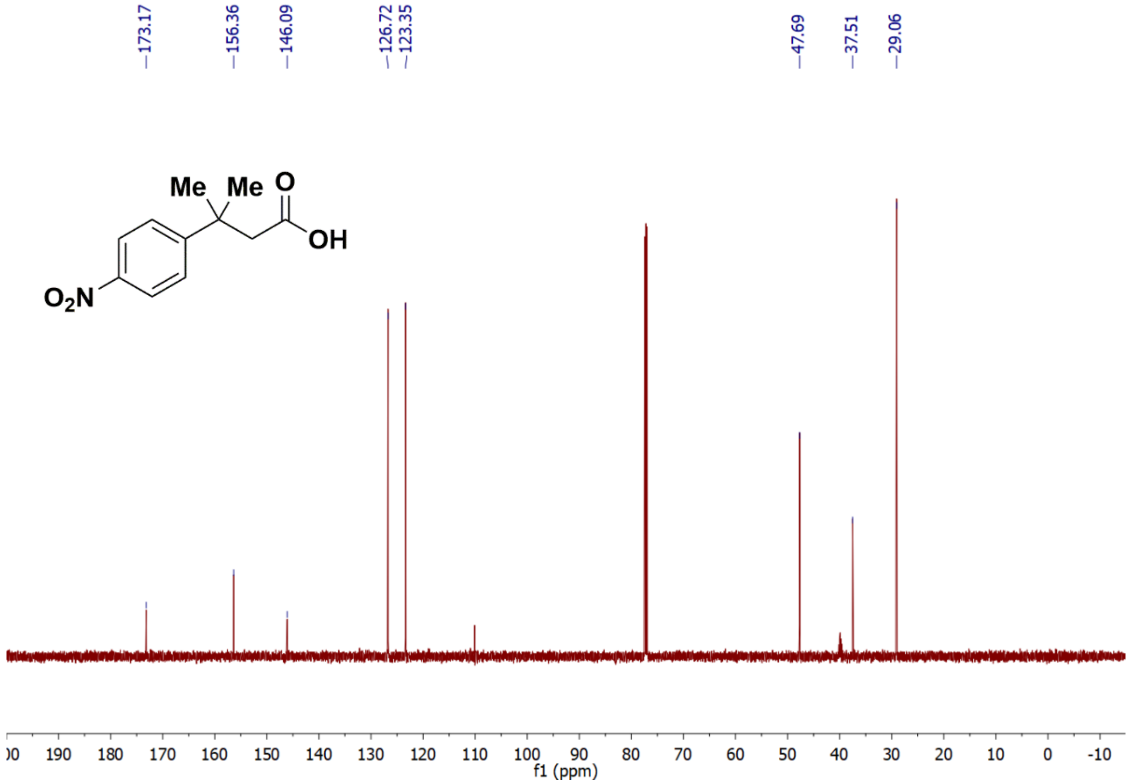

^{13}C {^{1}H} NMR spectrum of **3-methyl-3-(4-nitrophenyl)butanoic acid (1af)** in $CDCl_3$ + 2 drops DMSO-d_6 at 150 MHz

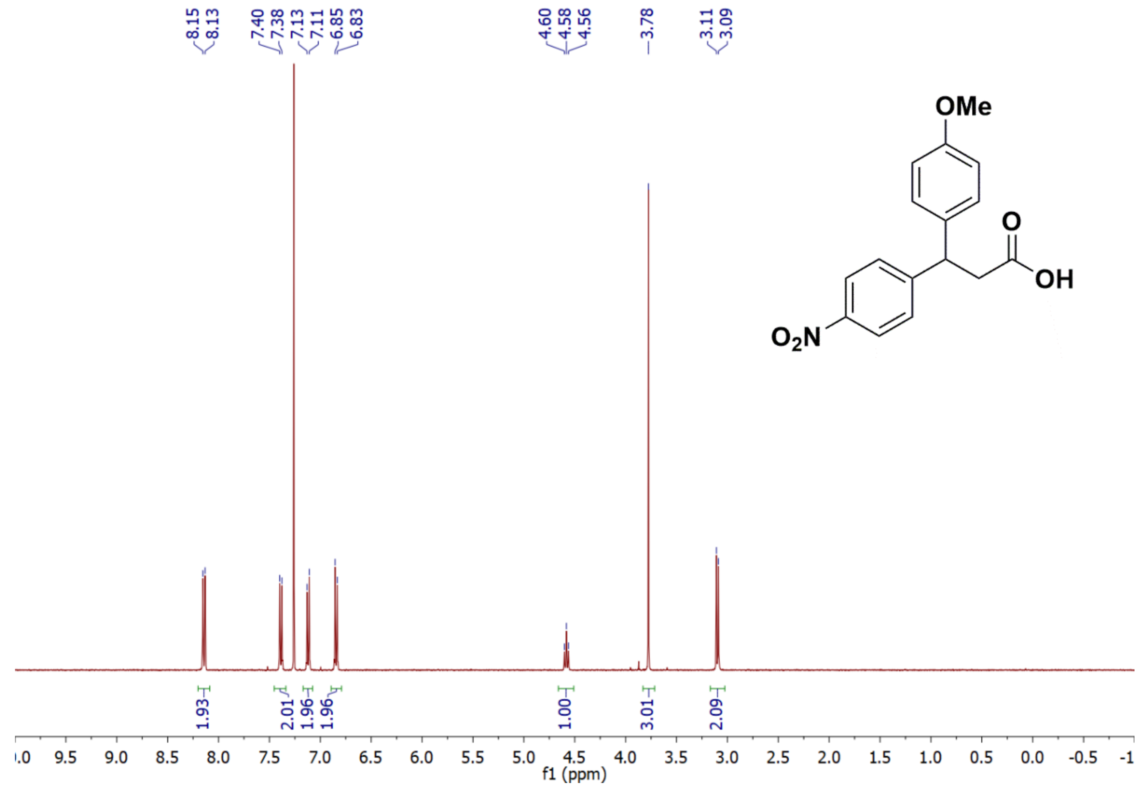

^{1}H NMR spectrum of **3-(4-methoxyphenyl)-3-(4-nitrophenyl)propanoic acid (1ag)** in $CDCl_3$ at 400 MHz

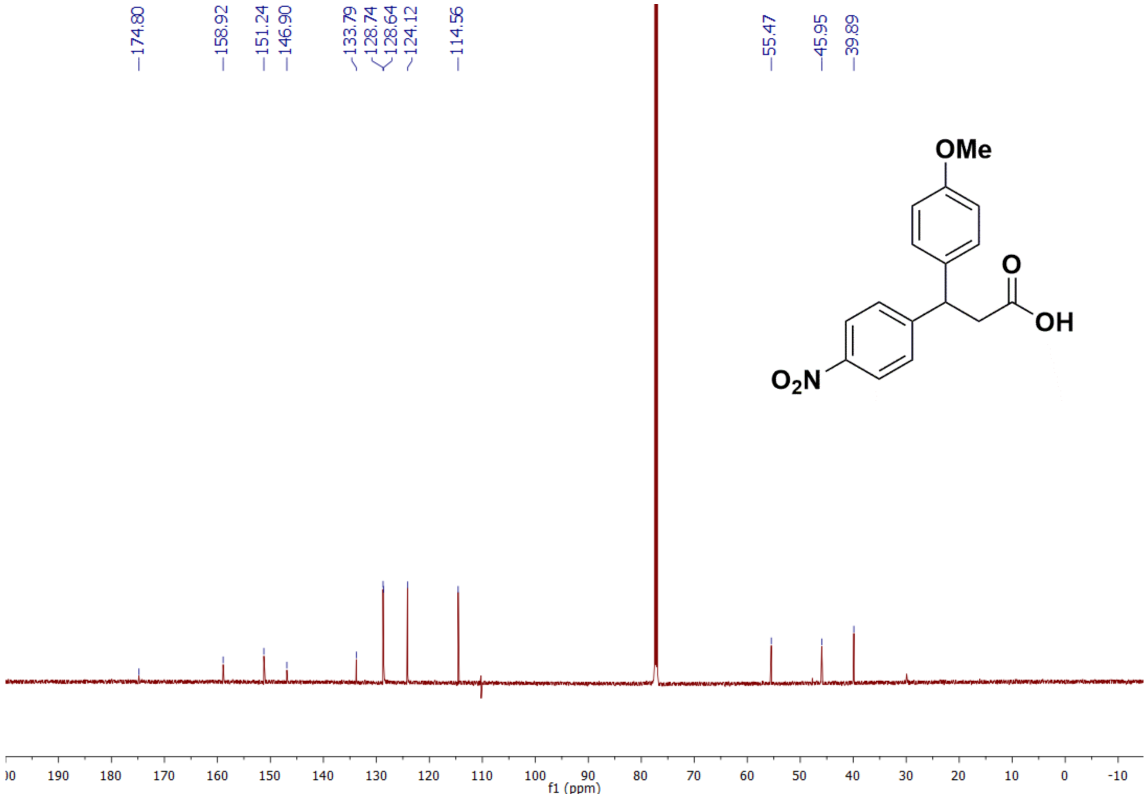

$^{13}C\{^{1}H\}$ NMR spectrum of **3-(4-methoxyphenyl)-3-(4-nitrophenyl)propanoic acid (1ag)** in $CDCl_3$ at 150 MHz

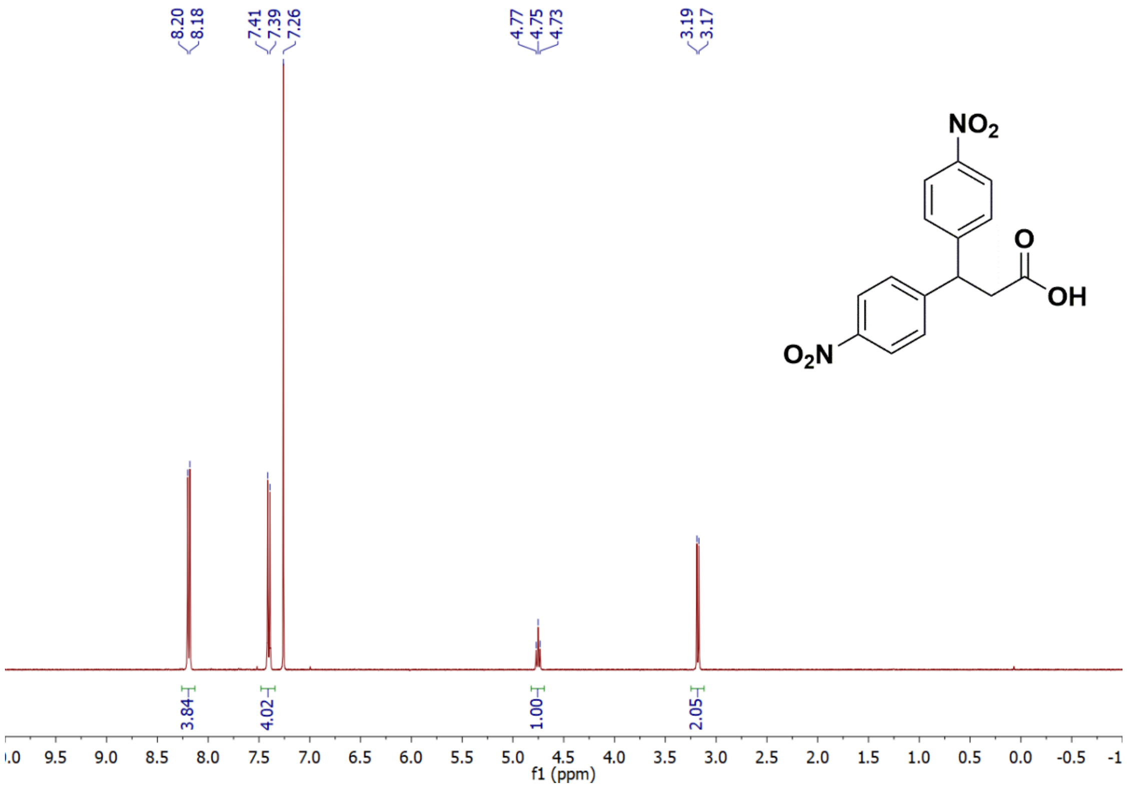

^{1}H NMR spectrum of **3,3-bis(4-nitrophenyl)propanoic acid (1ah)** in $CDCl_3$ at 400 MHz

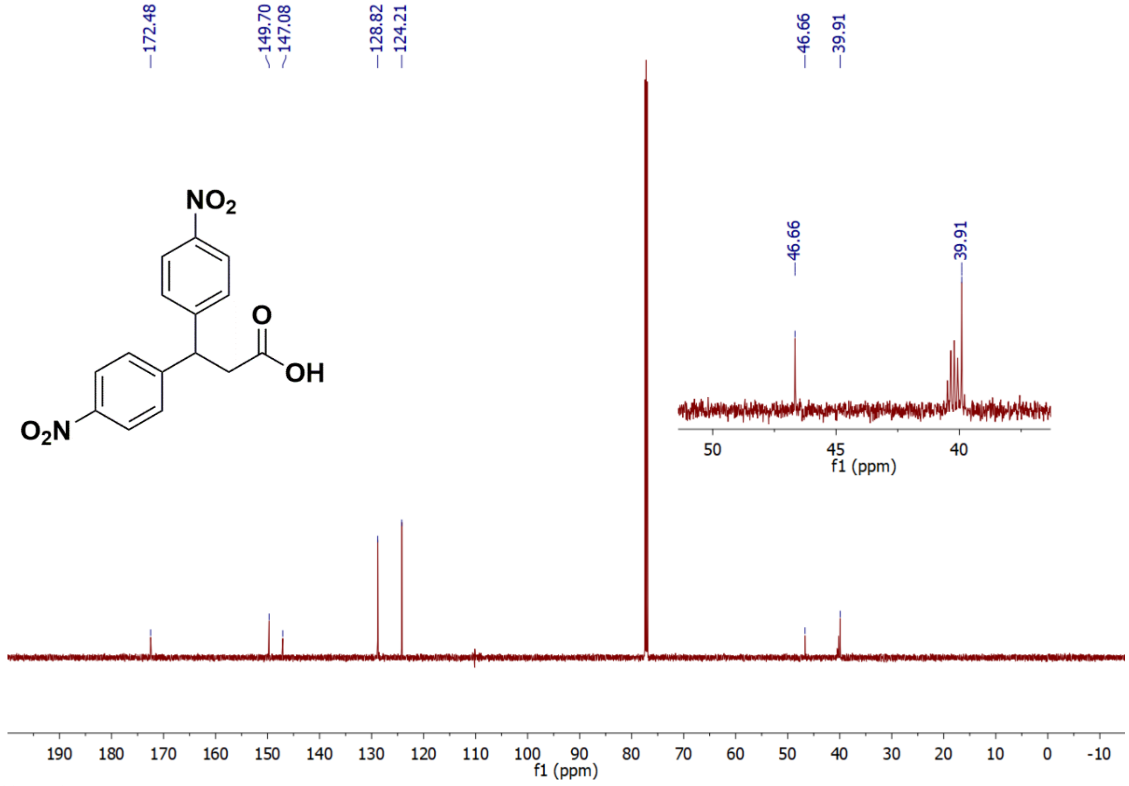

^{13}C{^{1}H} NMR spectrum of **3,3-bis(4-nitrophenyl)propanoic acid (1ah)** in $CDCl_3$ + 2 drops DMSO-d_6 at 150 MHz

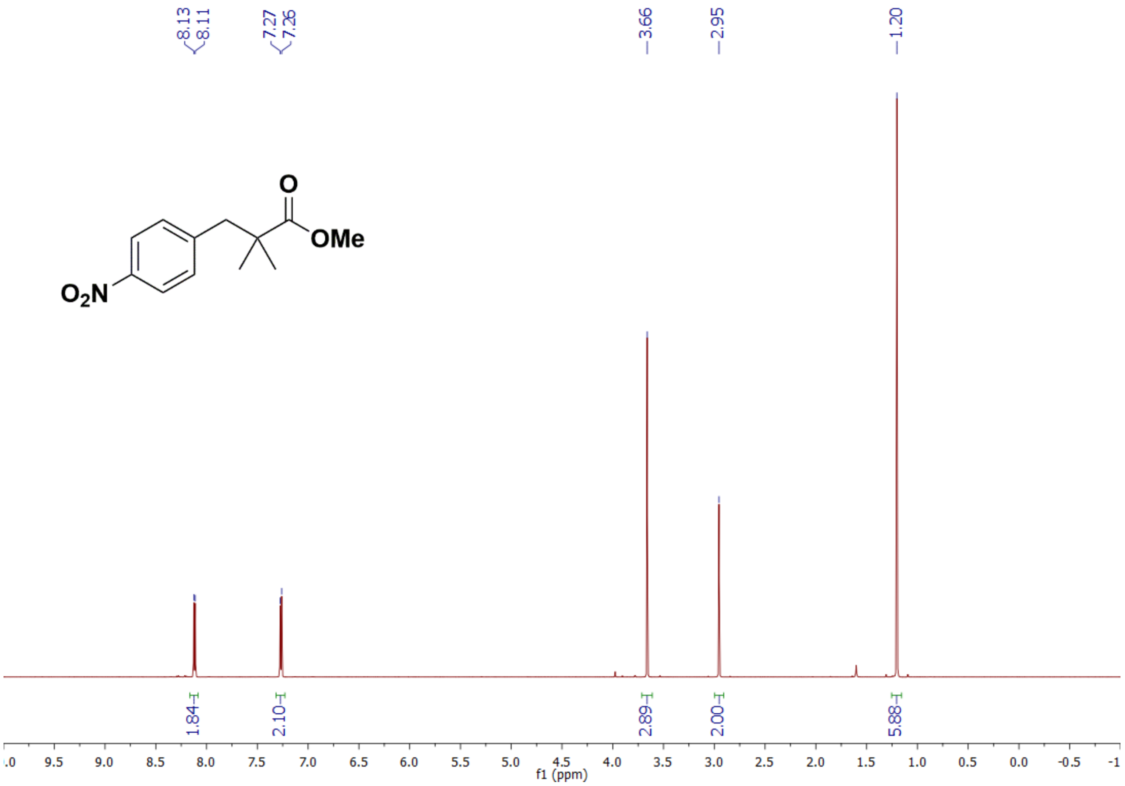

^{1}H NMR spectrum of **methyl 2,2-dimethyl-3-(4-nitrophenyl)propionate (1ai)** in $CDCl_3$ at 600 MHz

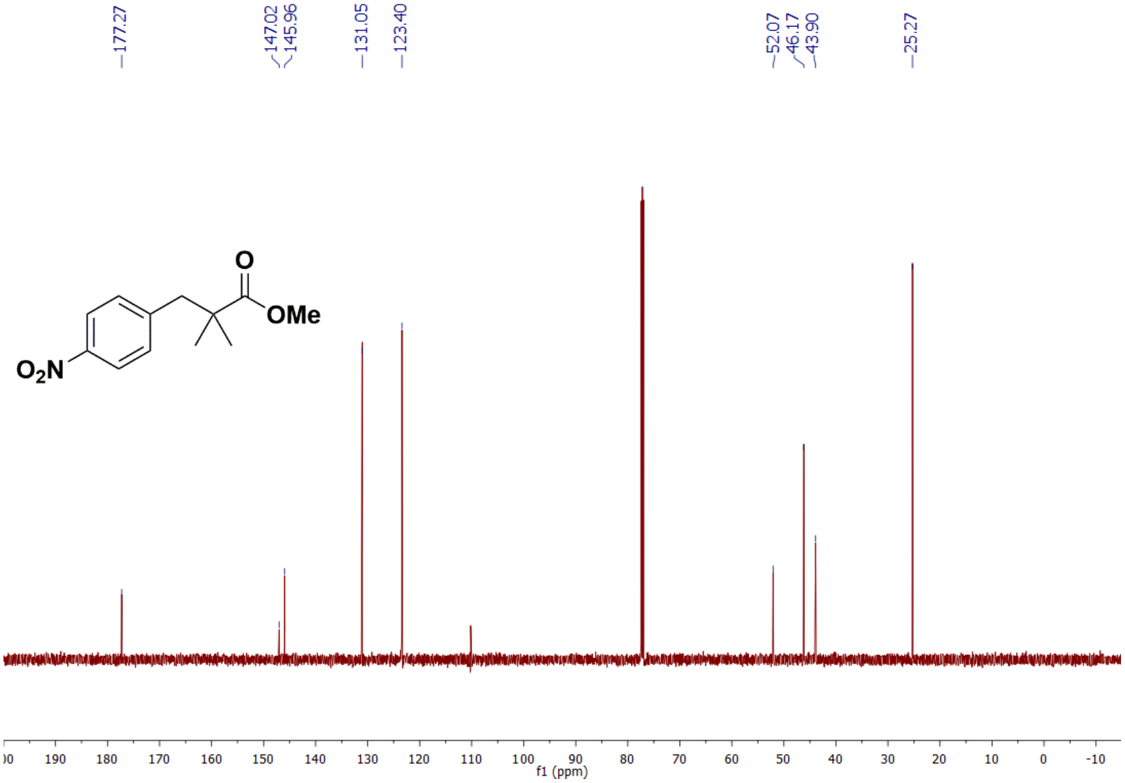

^{13}C{^{1}H} NMR spectrum of **methyl 2,2-dimethyl-3-(4-nitrophenyl)propionate (1ai)** in $CDCl_3$ at 150 MHz

XIII. ^{1}H and ^{13}C NMR Spectra of Decarboxylative Elimination Products (2)

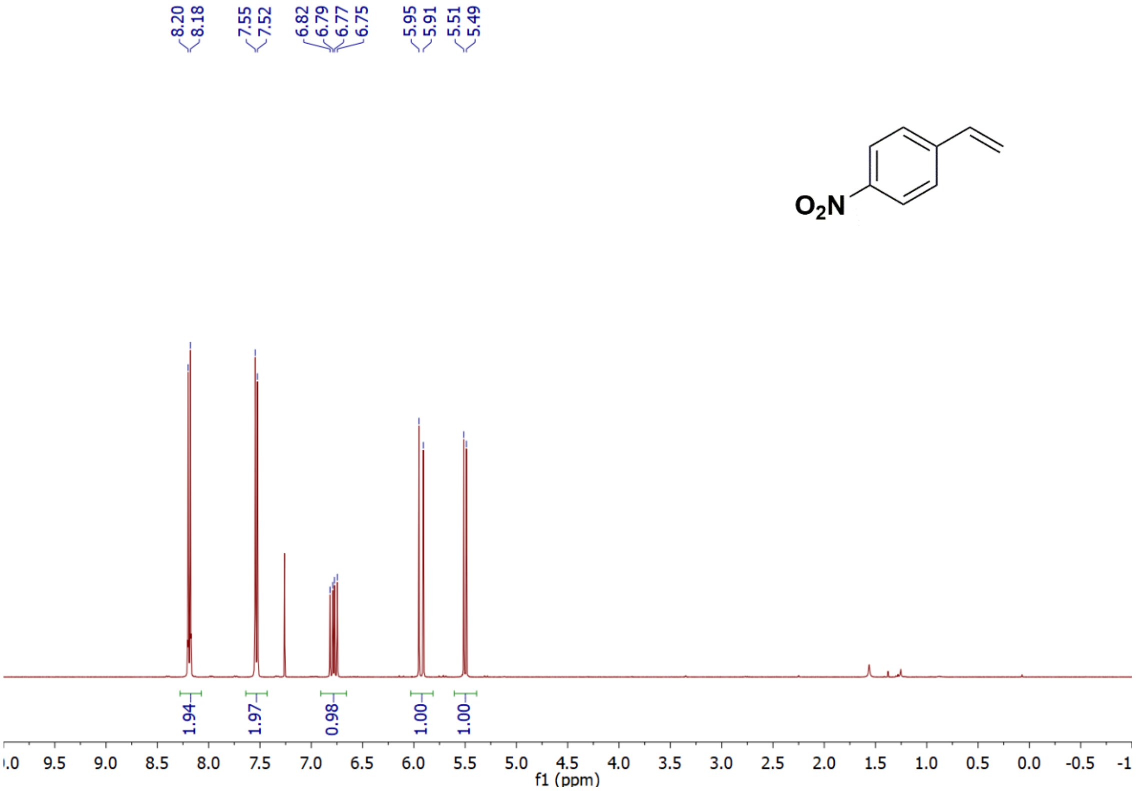

^{1}H NMR spectrum of **4-nitrostyrene (2a)** in $CDCl_3$ at 400 MHz

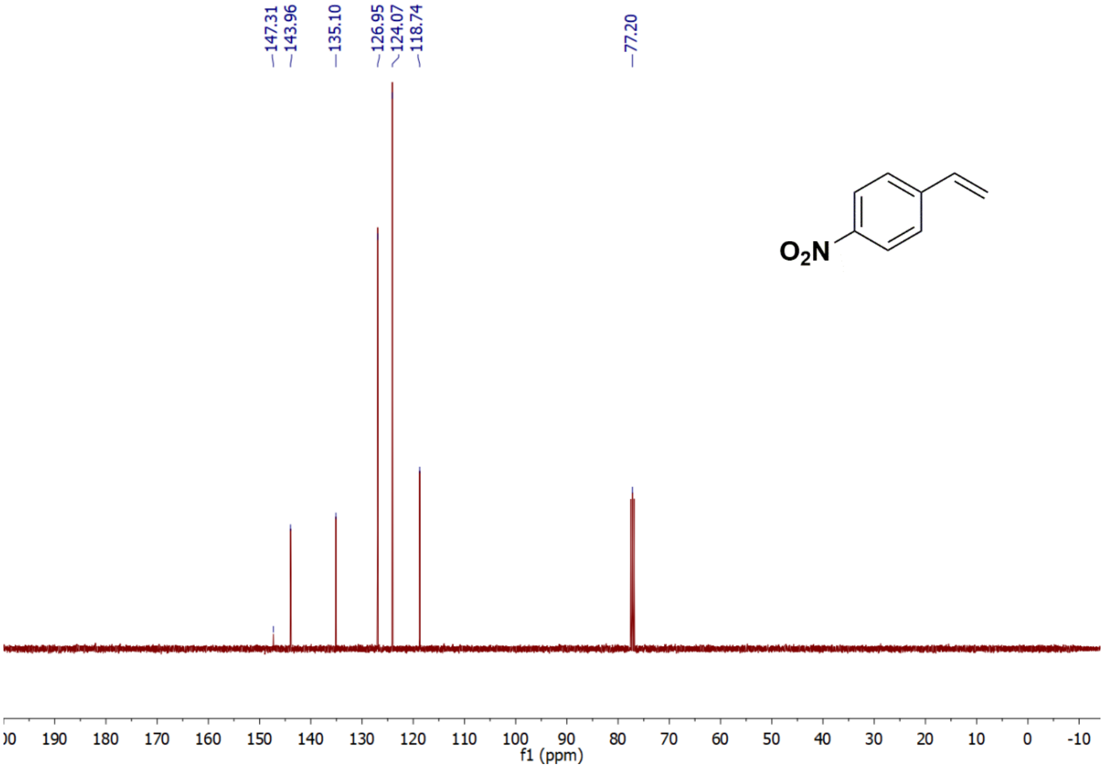

^{13}C{^{1}H} NMR spectrum of **4-nitrostyrene (2a)** in $CDCl_3$ at 150 MHz

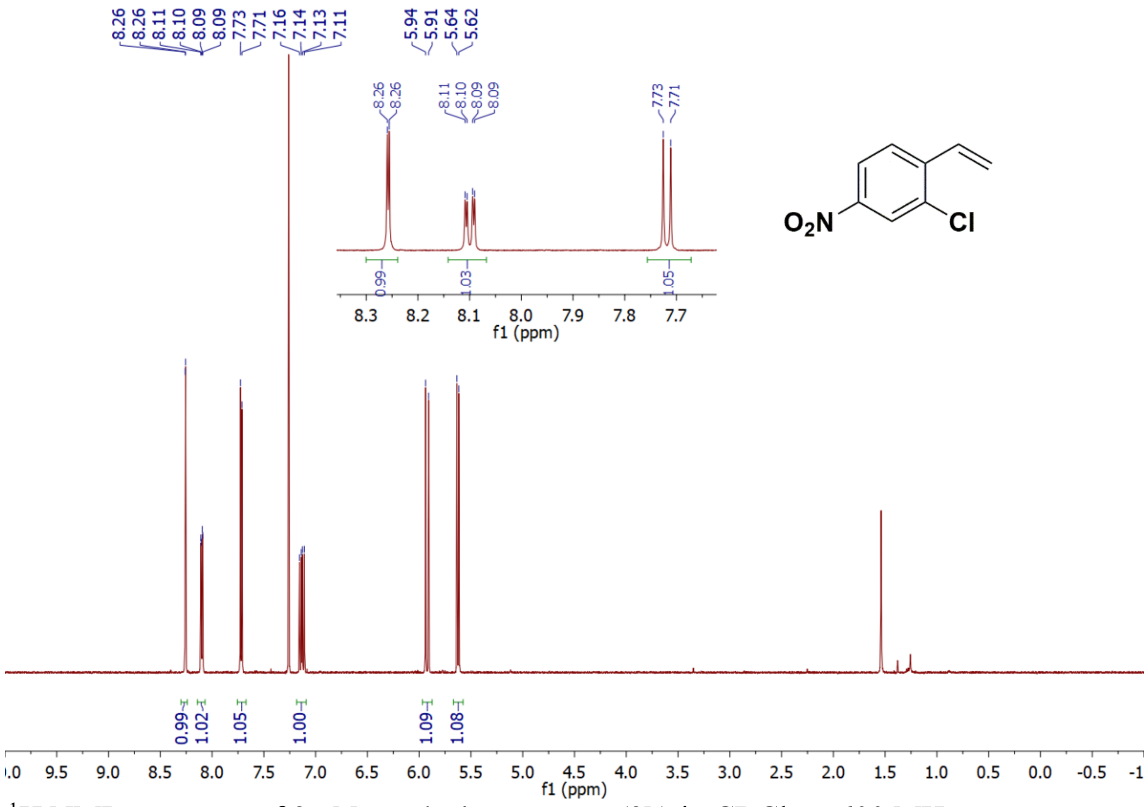

^{1}H NMR spectrum of **2-chloro-4-nitrostyrene (2b)** in $CDCl_3$ at 600 MHz

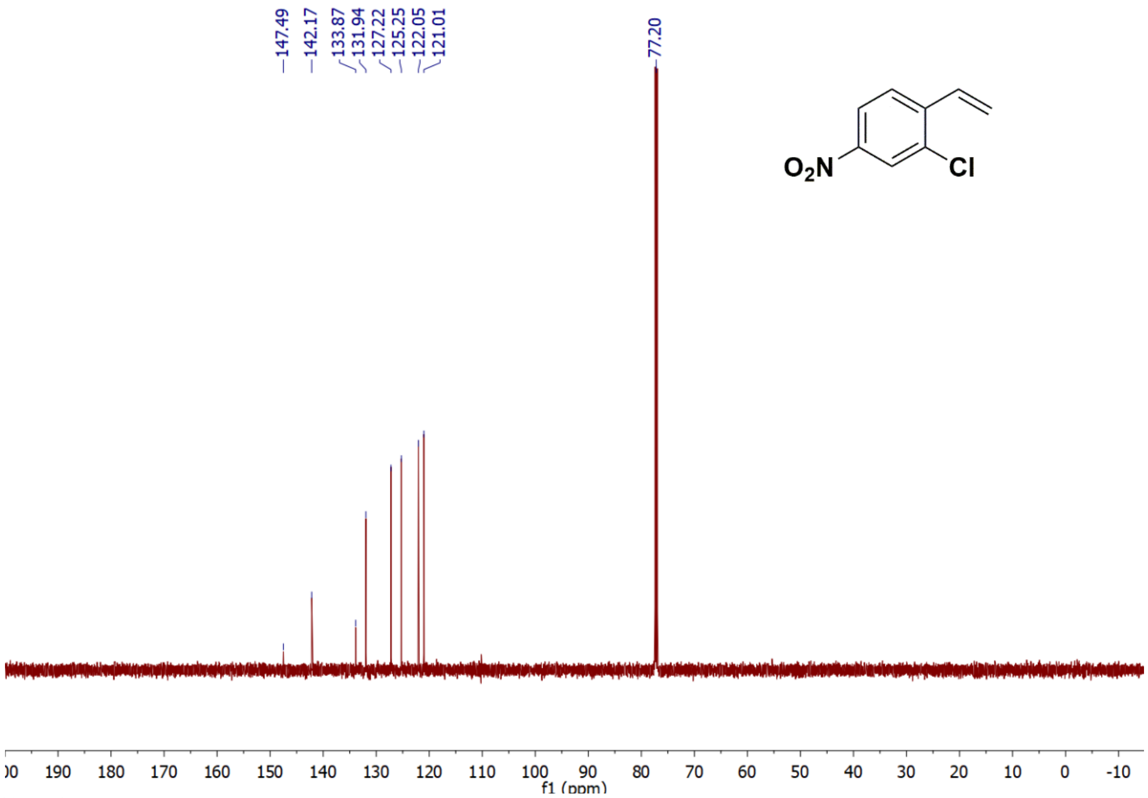

$^{13}C\{^{1}H\}$ NMR spectrum of **2-chloro-4-nitrostyrene (2b)** in $CDCl_3$ at 150 MHz

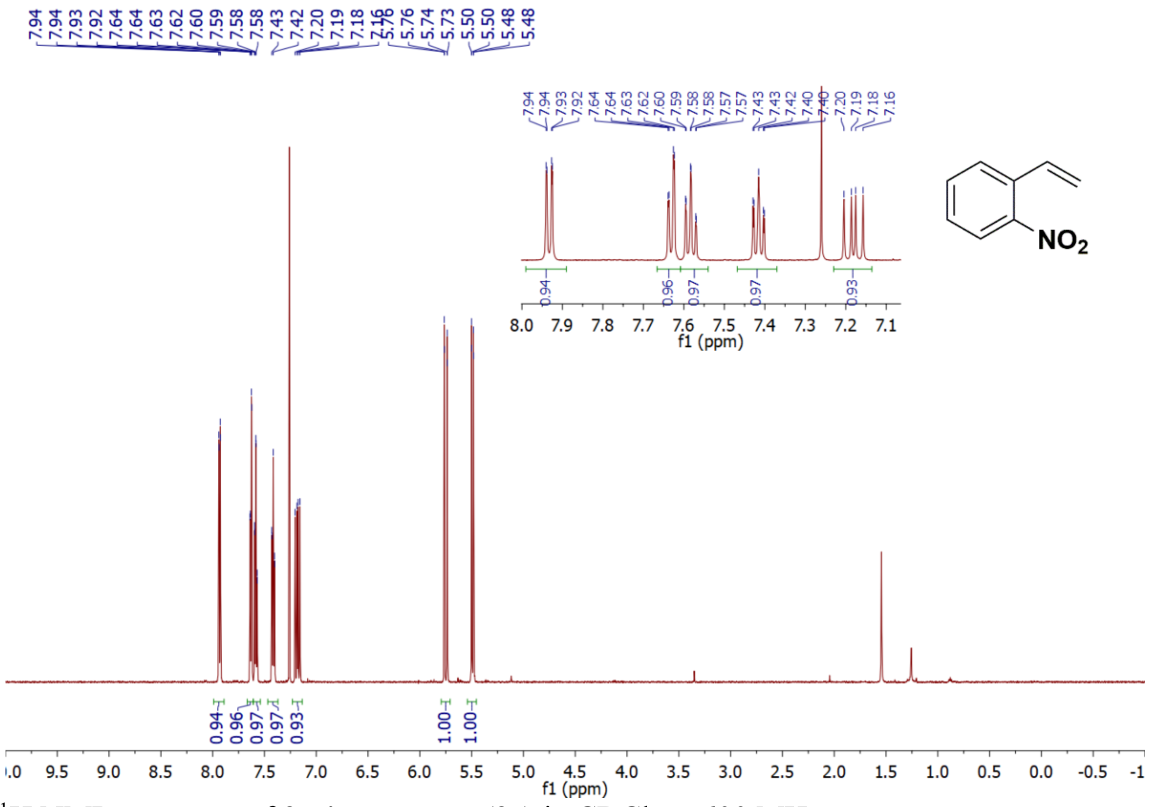

^{1}H NMR spectrum of **2-nitrostyrene (2c)** in $CDCl_3$ at 600 MHz

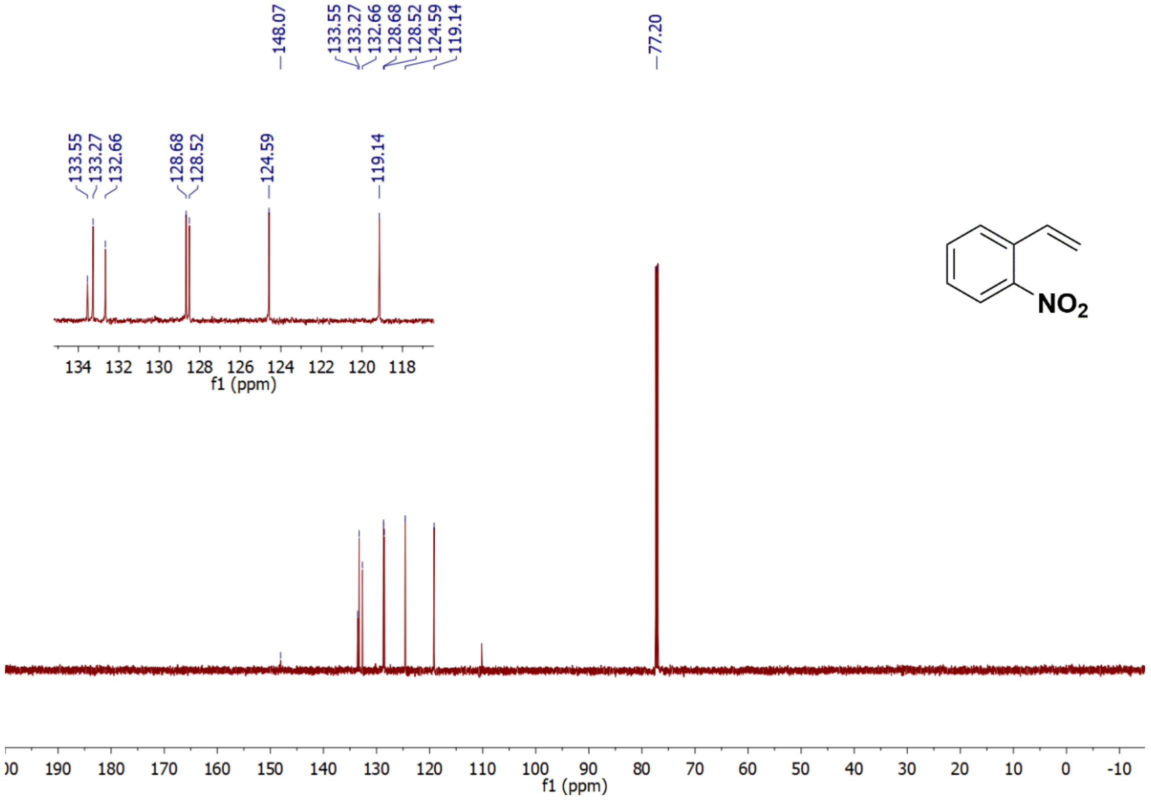

^{13}C{^{1}H} NMR spectrum of **2-nitrostyrene (2c)** in $CDCl_3$ at 150 MHz

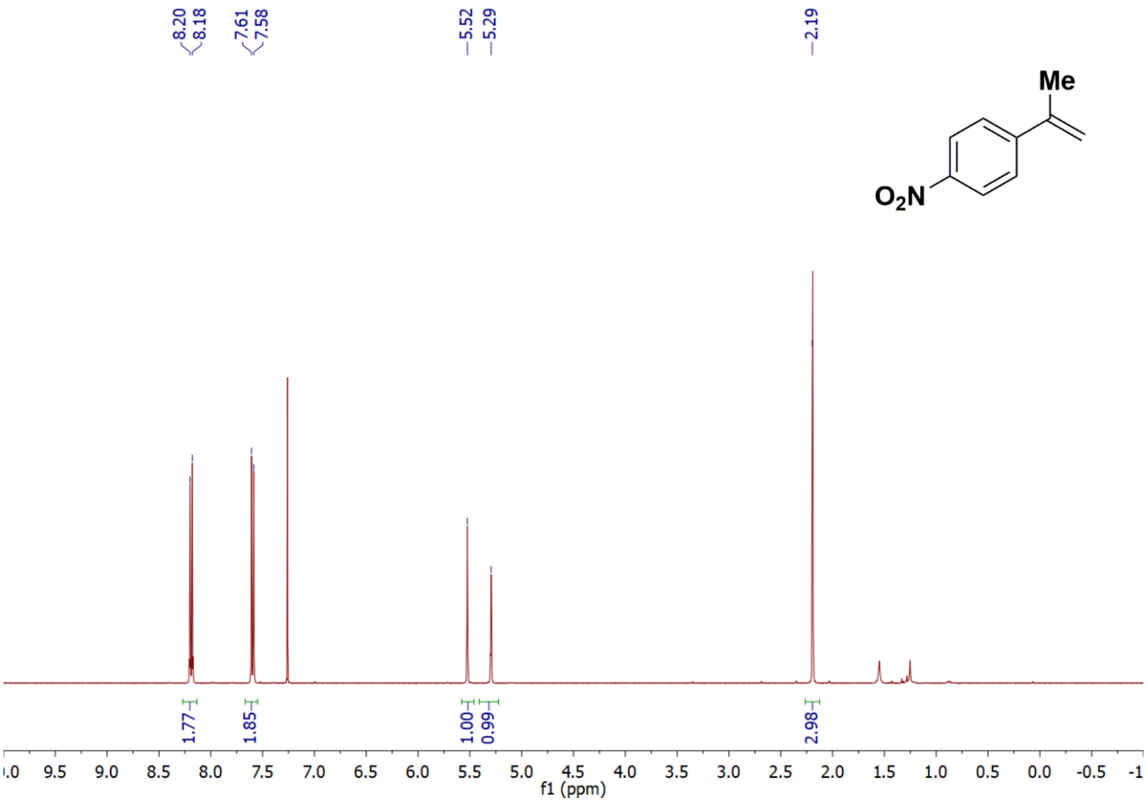

^{1}H NMR spectrum of **4-nitro-α-methylstyrene (2d)** in $CDCl_3$ at 400 MHz

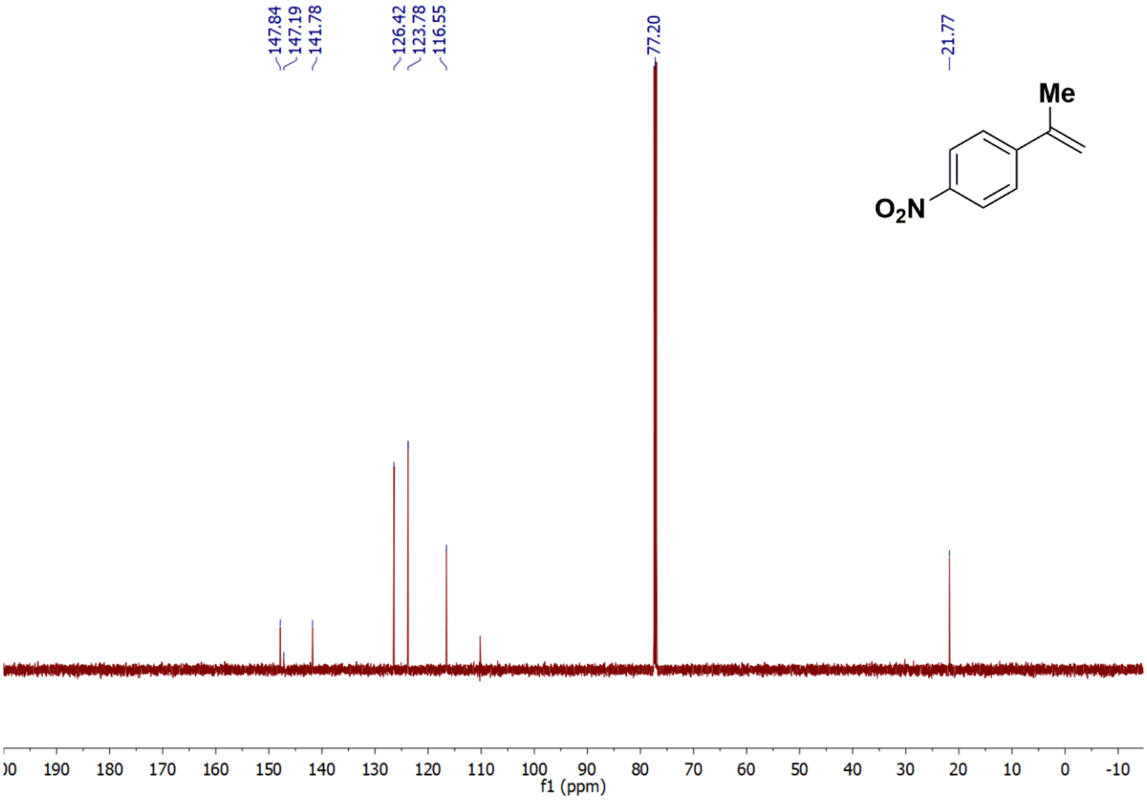

$^{13}C\{^{1}H\}$ NMR spectrum of **4-nitro-α-methylstyrene (2d)** in $CDCl_3$ at 150 MHz

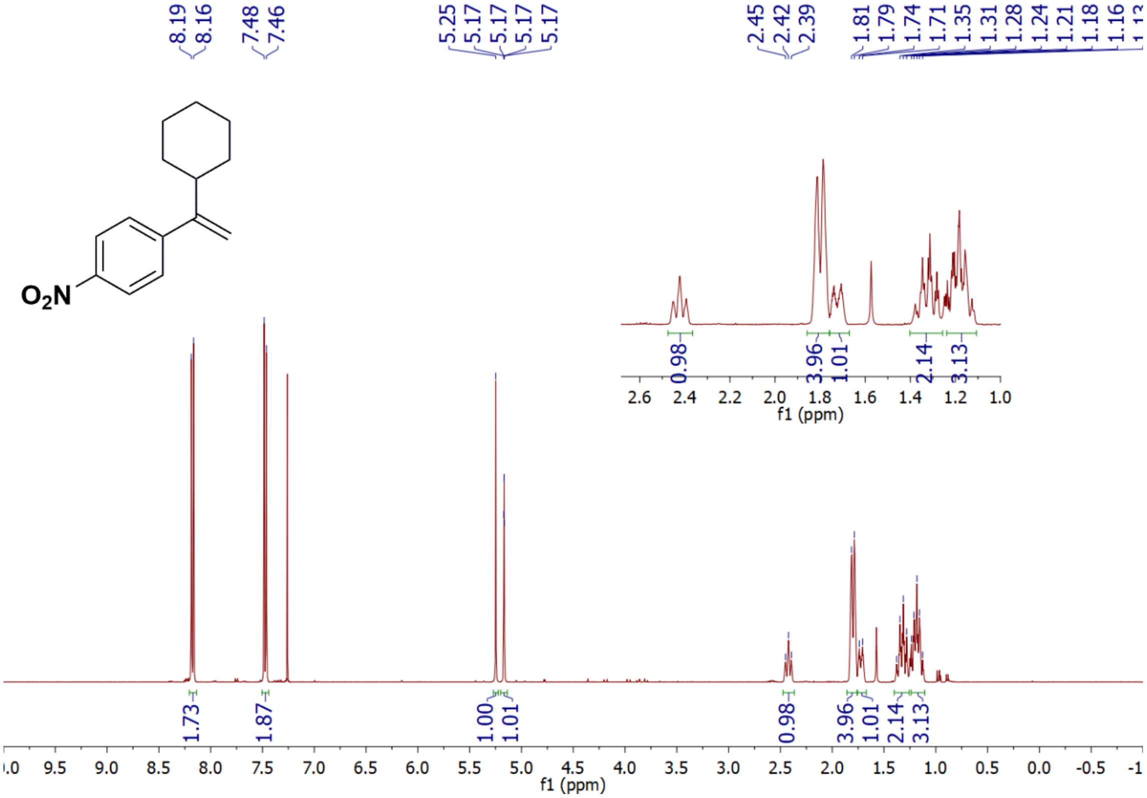

^{1}H NMR spectrum of **1-cyclohexyl-1-(4-nitrophenyl)ethylene (2e)** in $CDCl_3$ at 400 MHz

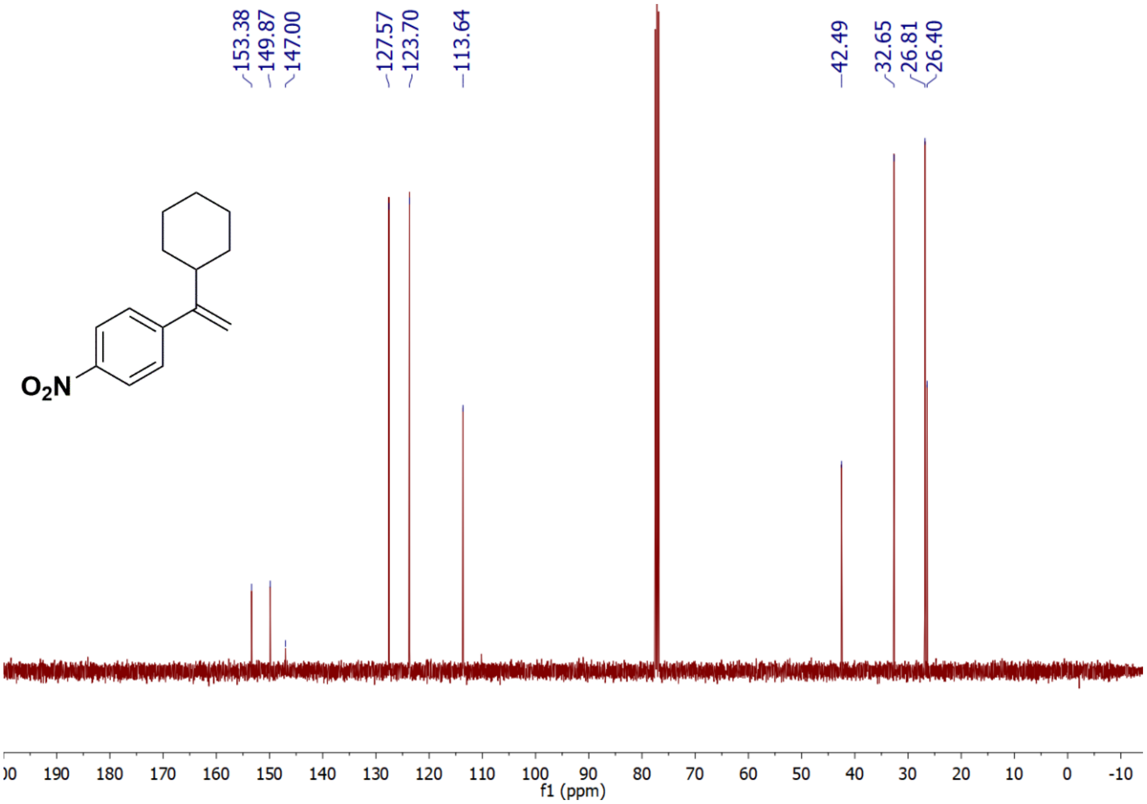

$^{13}C\{^{1}H\}$ NMR spectrum of **1-cyclohexyl-1-(4-nitrophenyl)ethylene (2e)** in $CDCl_3$ at 100 MHz

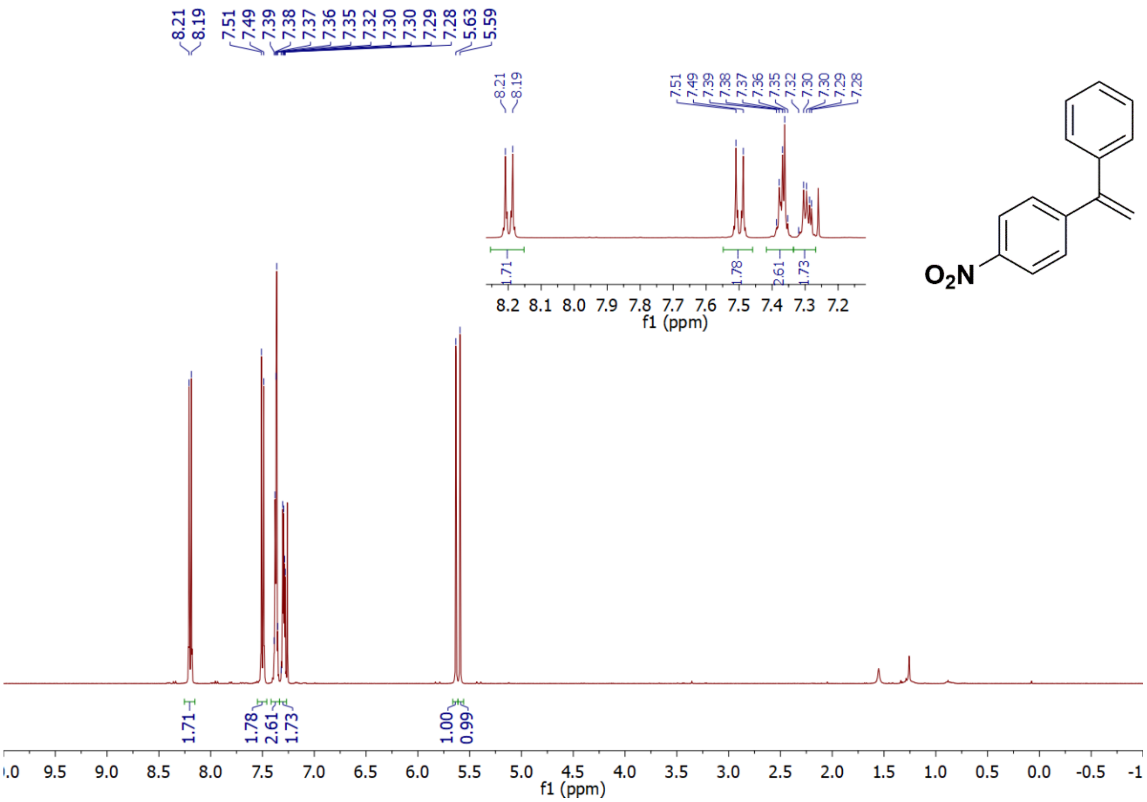

^{1}H NMR spectrum of **1-(4-nitrophenyl)-1-phenylethylene (2f)** in $CDCl_3$ at 400 MHz

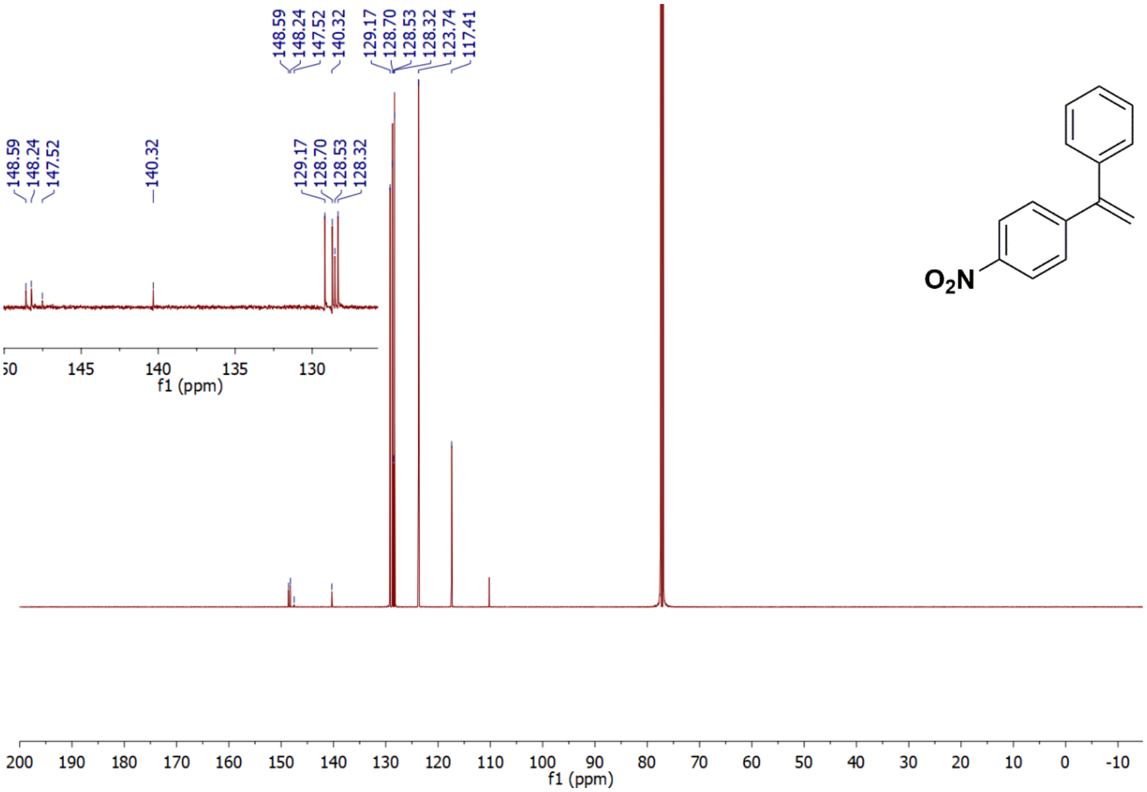

$^{13}C\{^{1}H\}$ NMR spectrum of **1-(4-nitrophenyl)-1-phenylethylene (2f)** in $CDCl_3$ at 150 MHz

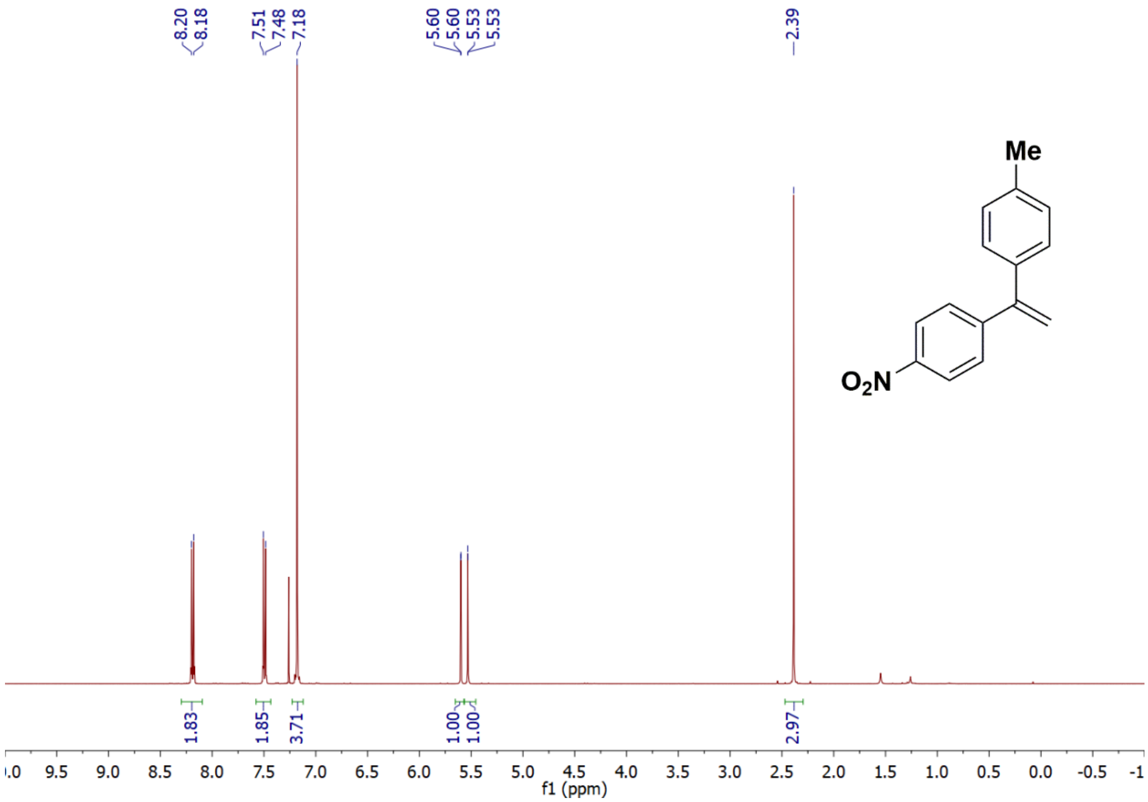

^{1}H NMR spectrum of **1-(4-methylphenyl)-1-(4-nitrophenyl)ethylene (2g)** in $CDCl_3$ at 400 MHz

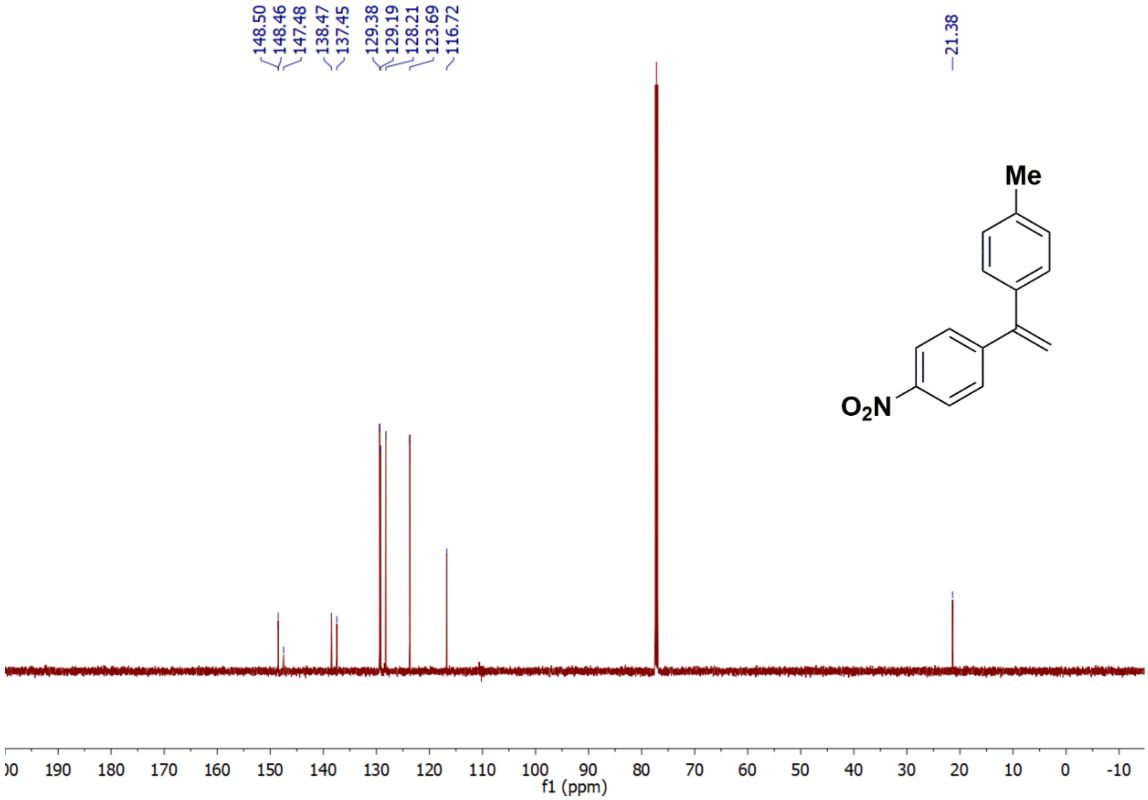

$^{13}C\{^{1}H\}$ spectrum of **1-(4-methylphenyl)-1-(4-nitrophenyl)ethylene (2g)** in $CDCl_3$ at 150 MHz

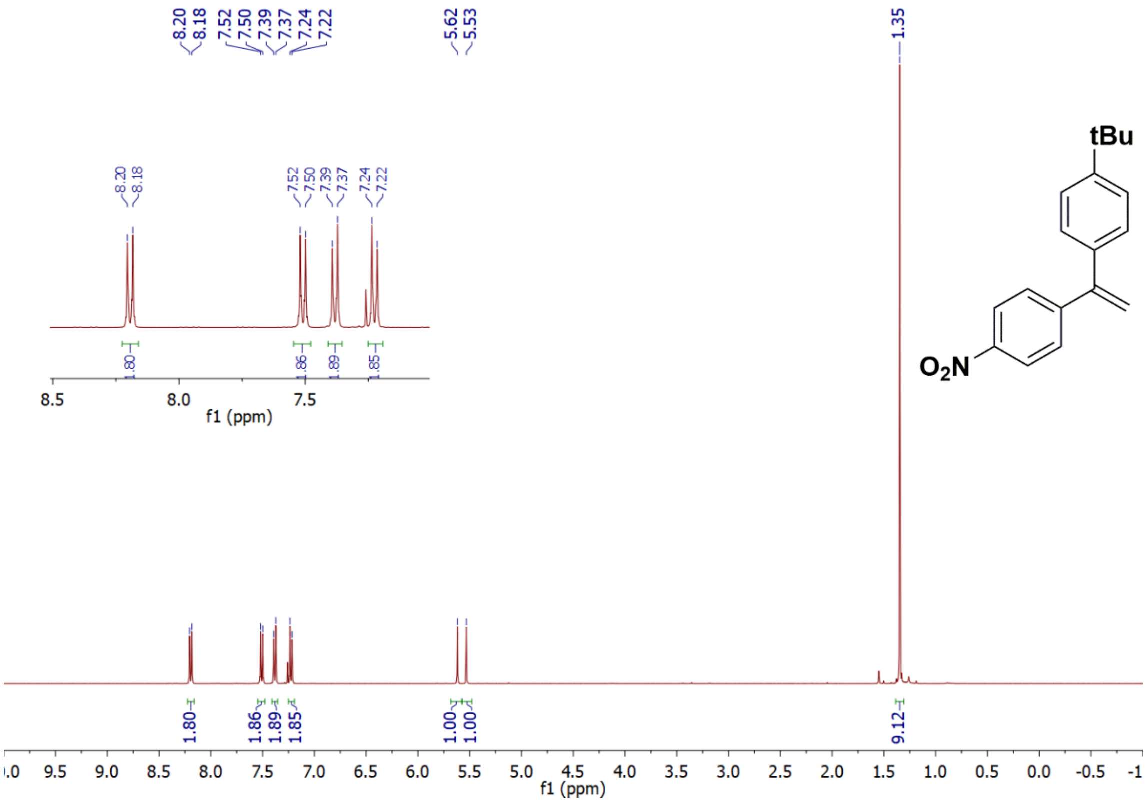

^{1}H NMR spectrum of **1-(4-nitrophenyl)-1-(4-*tert*-butylphenyl)ethylene (2h)** in $CDCl_3$ at 400 MHz

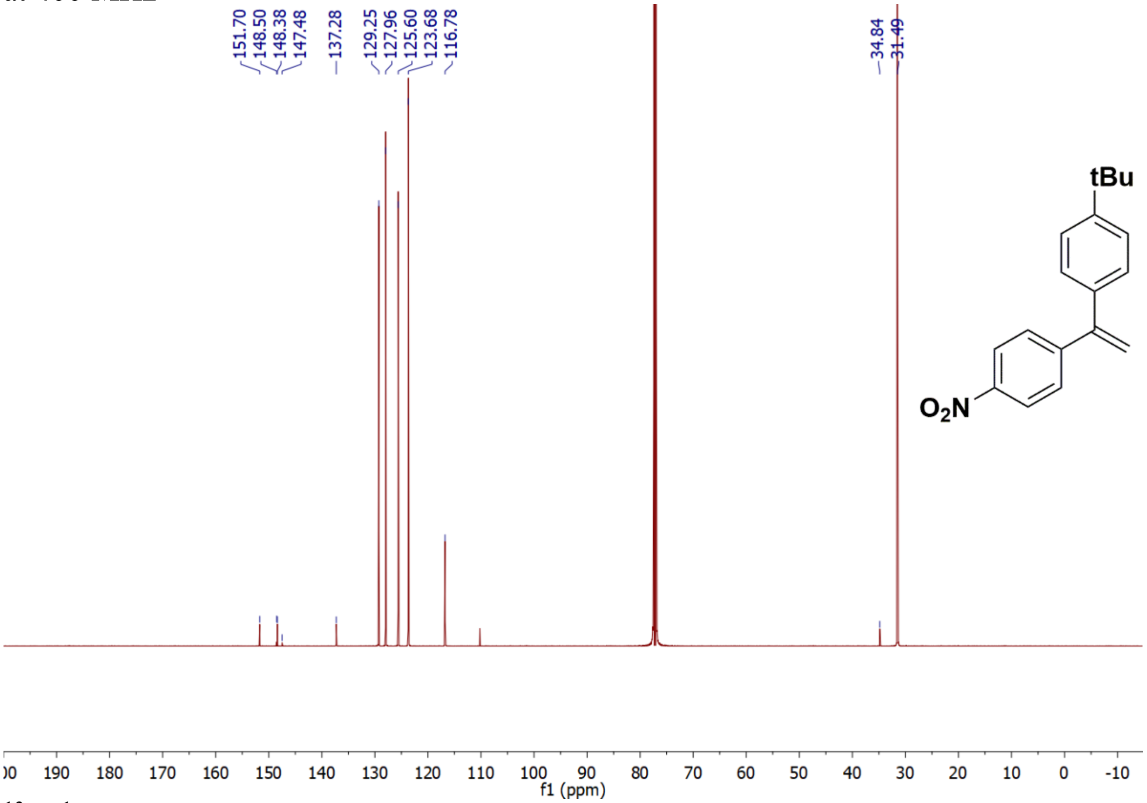

^{13}C{^{1}H} NMR spectrum of **1-(nitrophenyl)-1-(4-*tert*-butylphenyl)ethylene (2h)** in $CDCl_3$ at 150 MHz

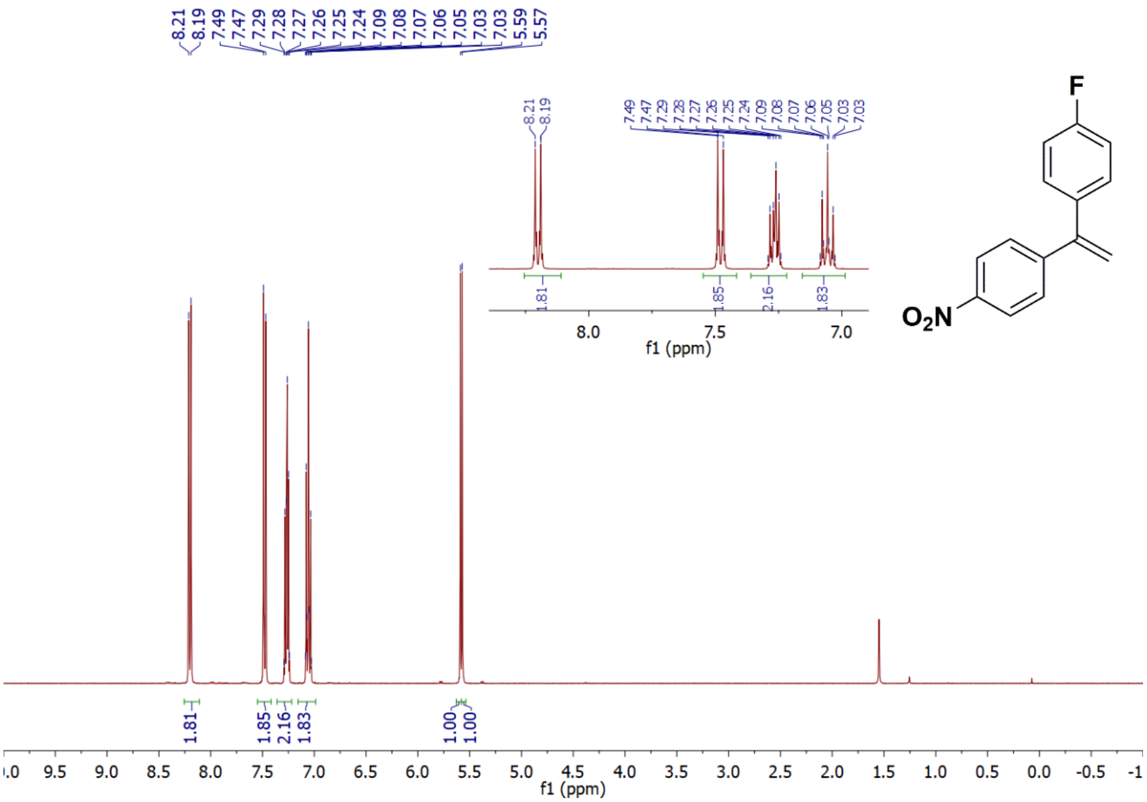

^{1}H NMR spectrum of **1-(4-fluorophenyl)-1-(4-nitrophenyl)ethylene (2i)** in $CDCl_3$ at 400 MHz

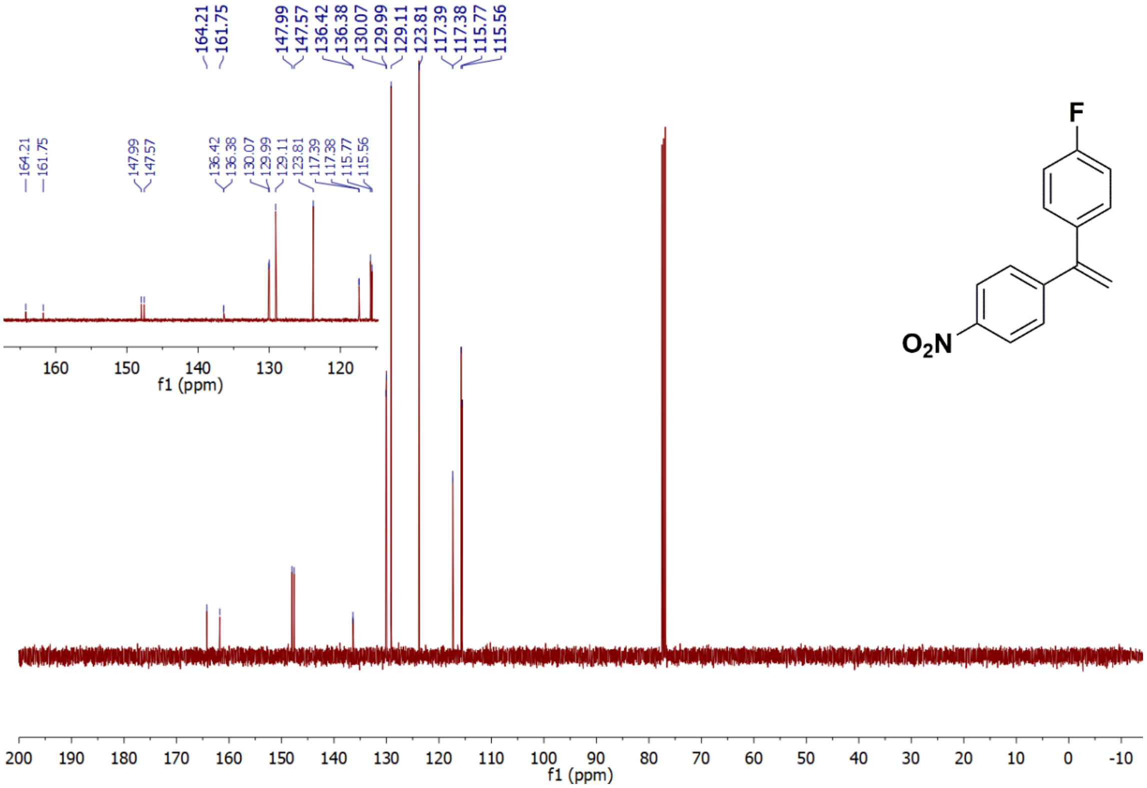

$^{13}C\{^1H\}$ NMR spectrum of **1-(4-fluorophenyl)-1-(4-nitrophenyl)ethylene (2i)** in $CDCl_3$ at 150 MHz

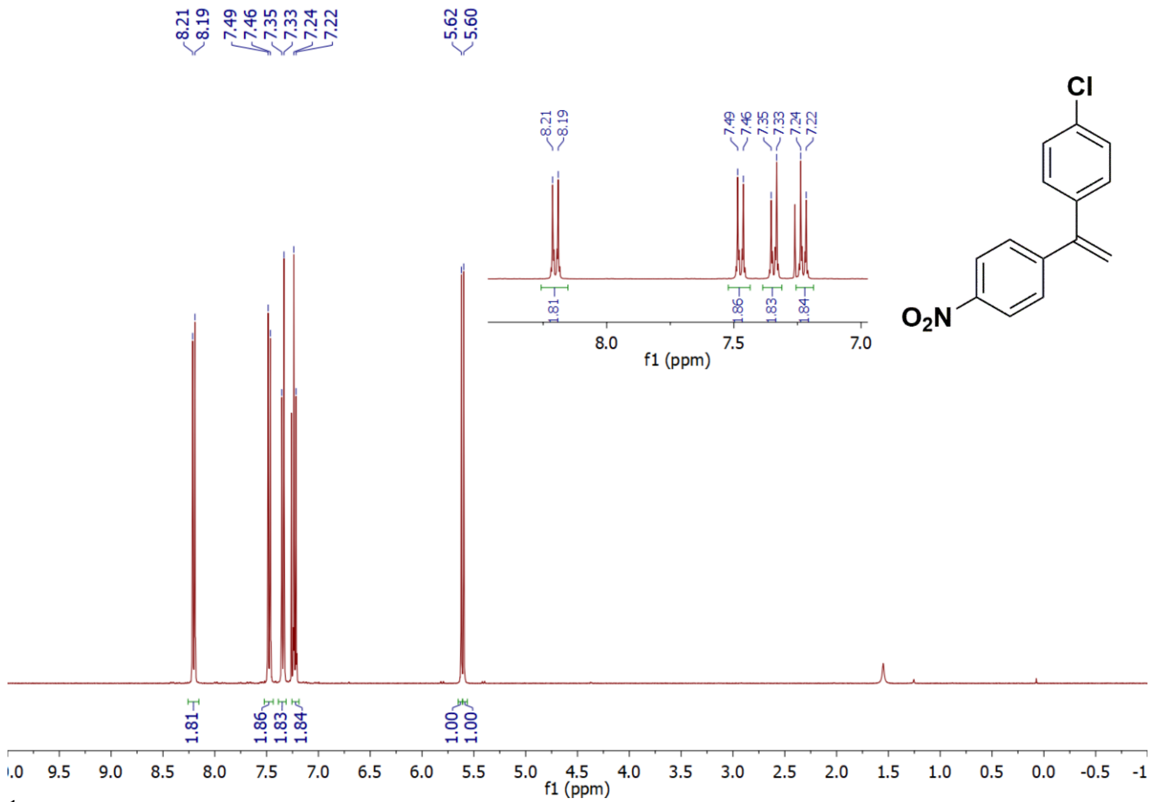

^{1}H NMR spectrum of **1-(4-chlorophenyl)-1-(4-nitrophenyl)ethylene (2j)** in $CDCl_3$ at 400 MHz

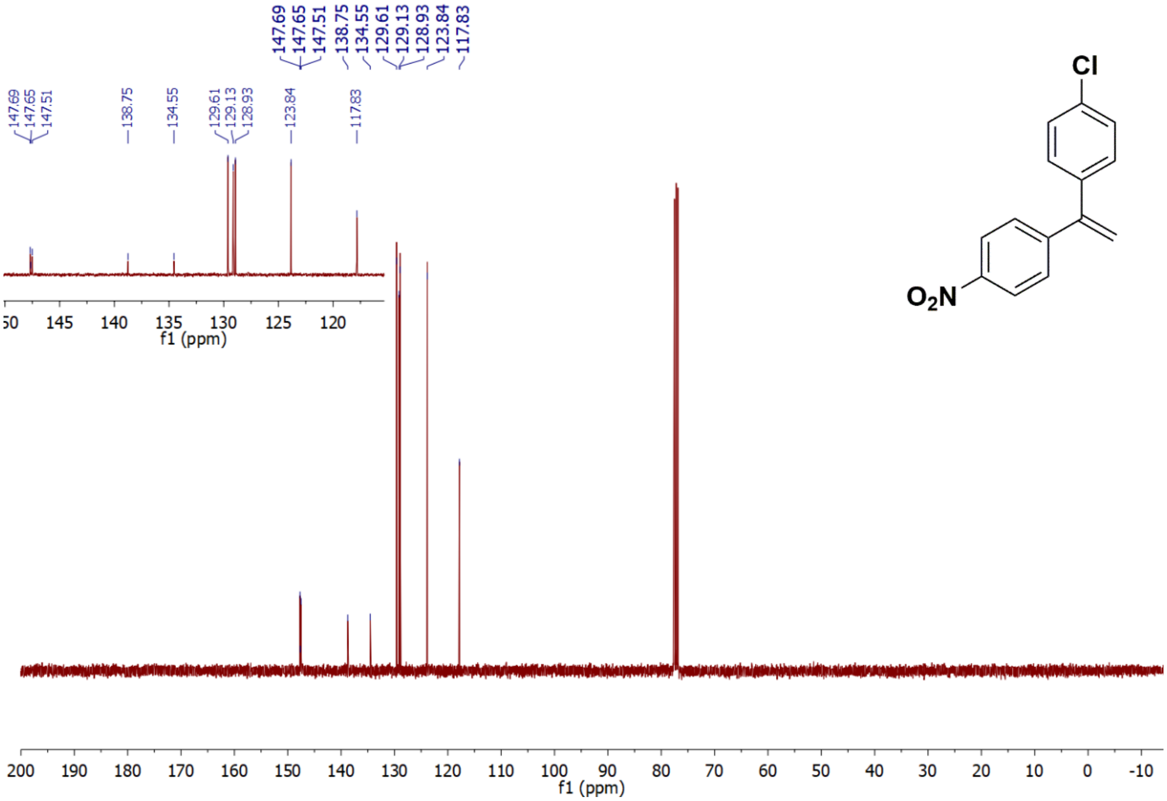

$^{13}C\{^{1}H\}$ NMR spectrum of **1-(4-chlorophenyl)-1-(4-nitrophenyl)ethylene (2j)** in $CDCl_3$ at 150 MHz

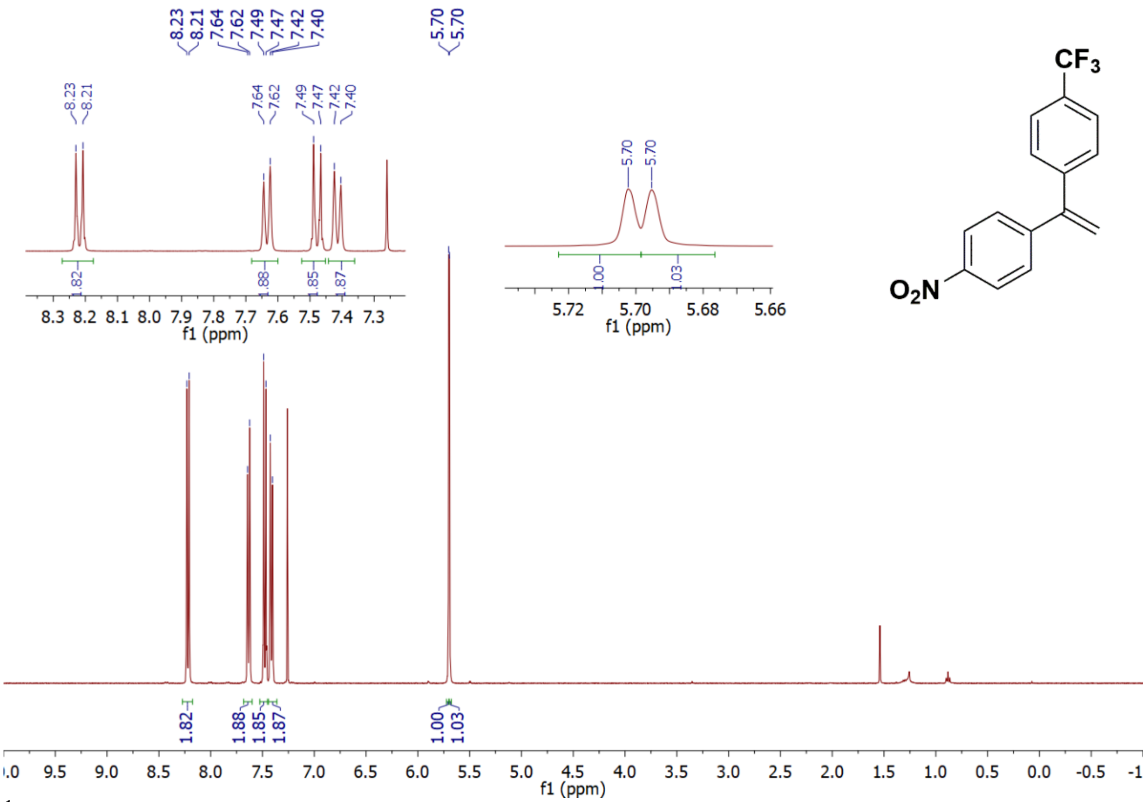

^{1}H NMR spectrum of **1-(4-nitrophenyl)-1-(4-trifluoromethylphenyl)ethylene (2k)** in $CDCl_3$ at 400 MHz

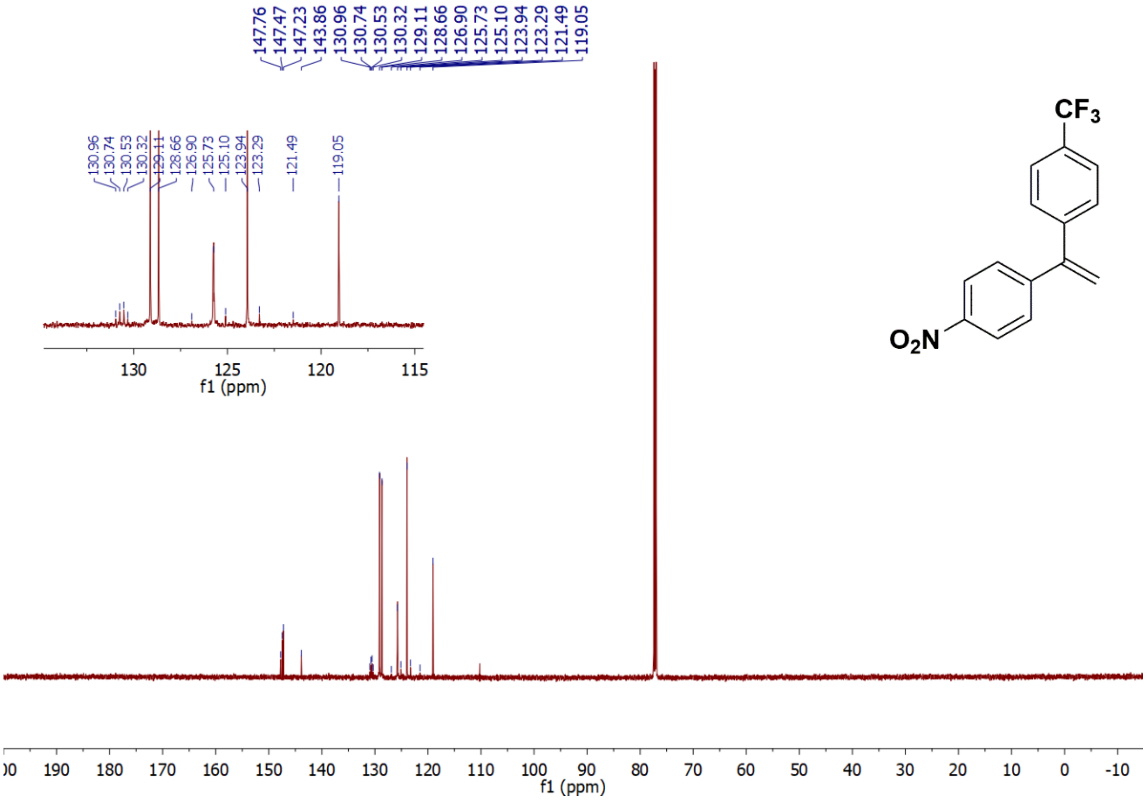

^{13}C{^{1}H} NMR spectrum of **1-(4-nitrophenyl)-1-(4-trifluoromethylphenyl)ethylene (2k)** in $CDCl_3$ at 150 MHz

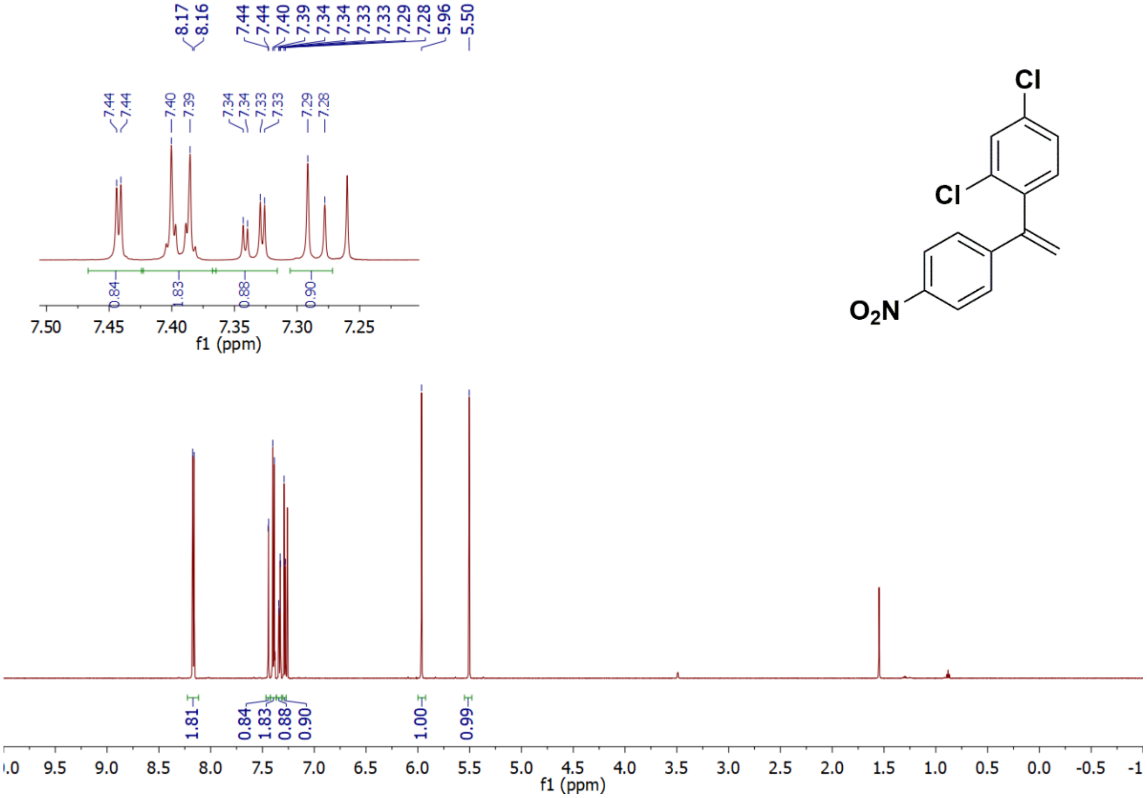

^{1}H NMR spectrum of **1-(2,4-dichlorophenyl)-1-(4-nitrophenyl)ethylene (2l)** in $CDCl_3$ at 600 MHz

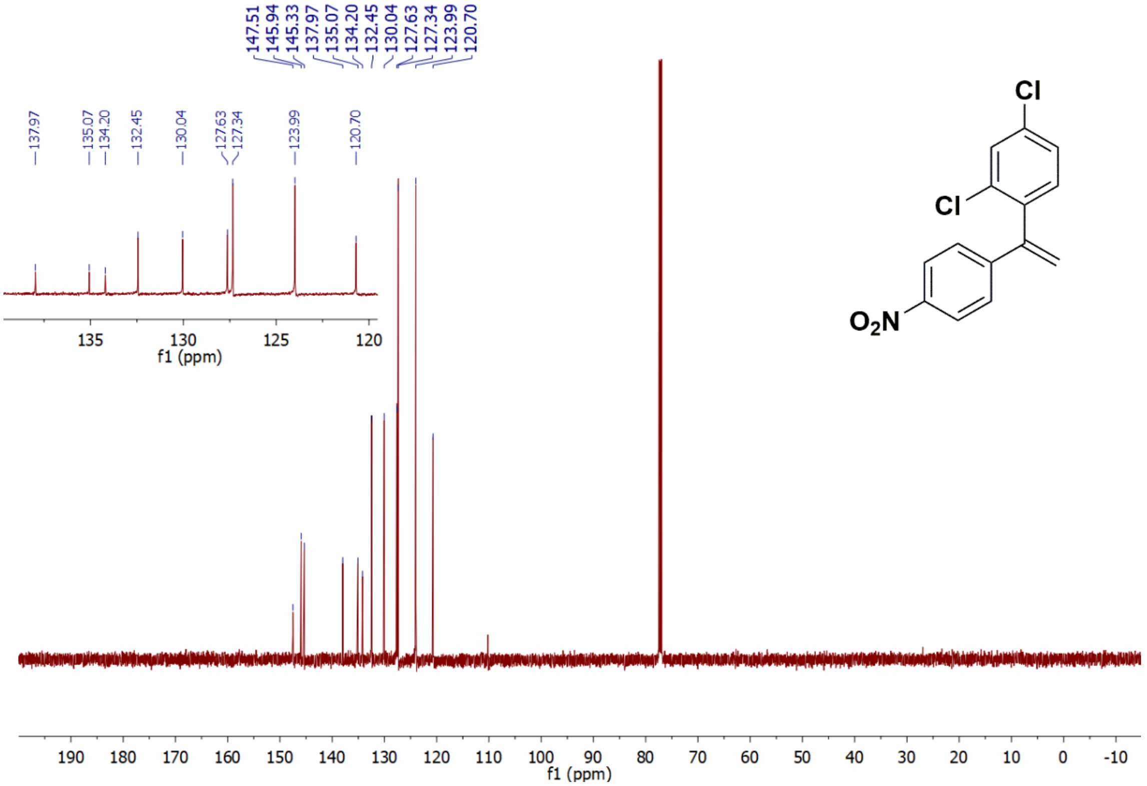

^{13}C{^{1}H} NMR spectrum of **1-(2,4-dichlorophenyl)-1-(4-nitrophenyl)ethylene (2l)** in $CDCl_3$ at 150 MHz

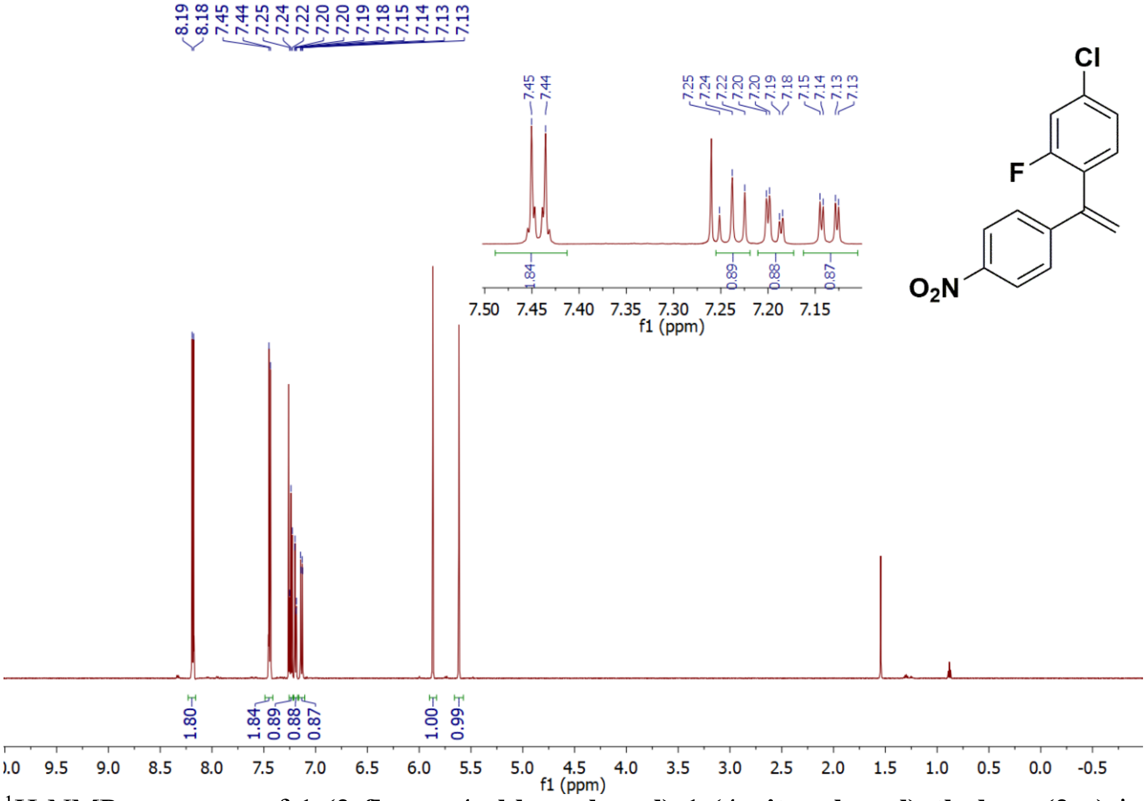

^{1}H NMR spectrum of **1-(2-fluoro-4-chlorophenyl)-1-(4-nitrophenyl)ethylene (2m)** in $CDCl_3$ at 600 MHz

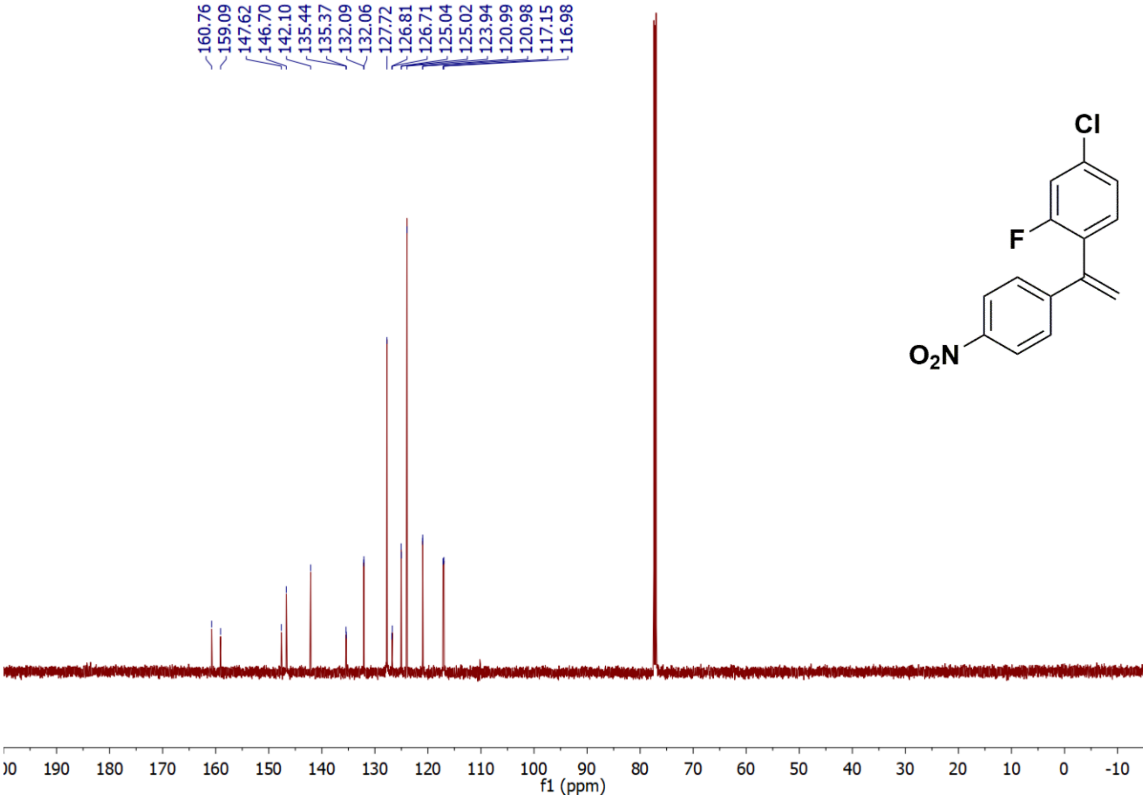

^{13}C{^{1}H} NMR spectrum of **1-(2-fluoro-4-chlorophenyl)-1-(4-nitrophenyl)ethylene (2m)** in $CDCl_3$ at 150 MHz

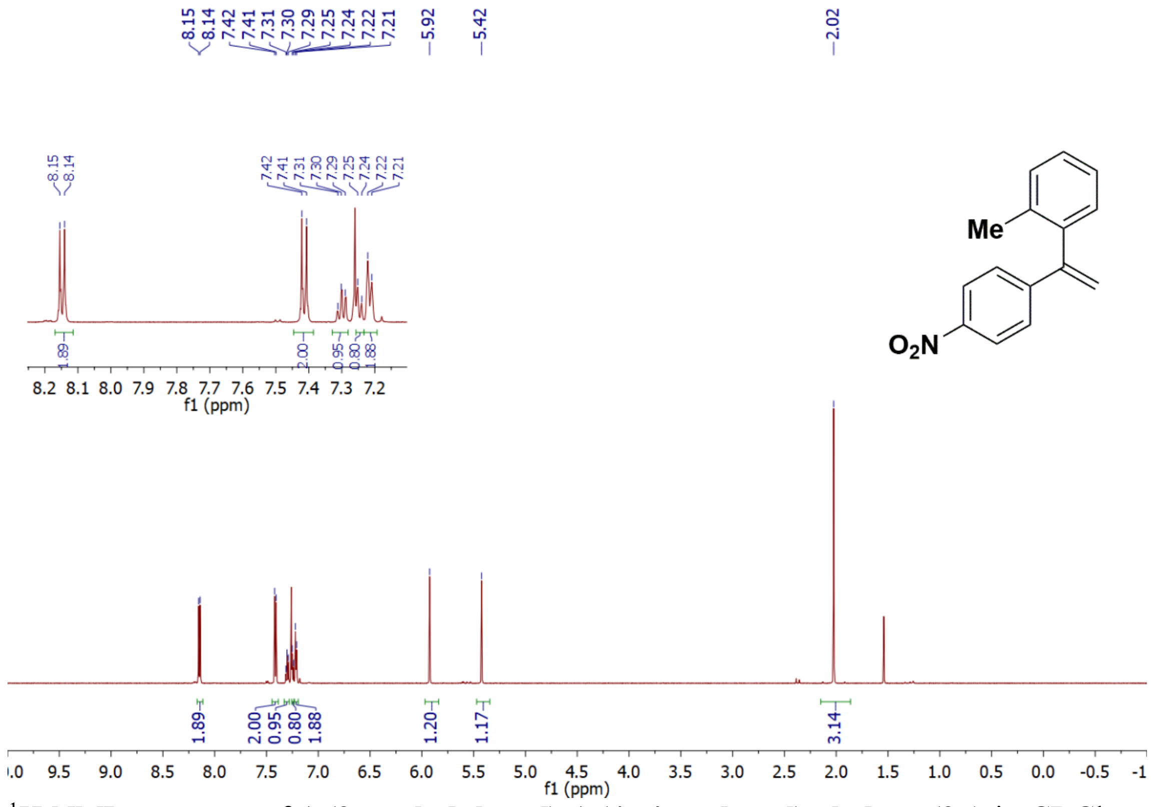

^{1}H NMR spectrum of **1-(2-methylphenyl)-1-(4-nitrophenyl)ethylene (2n)** in $CDCl_3$ at 600 MHz

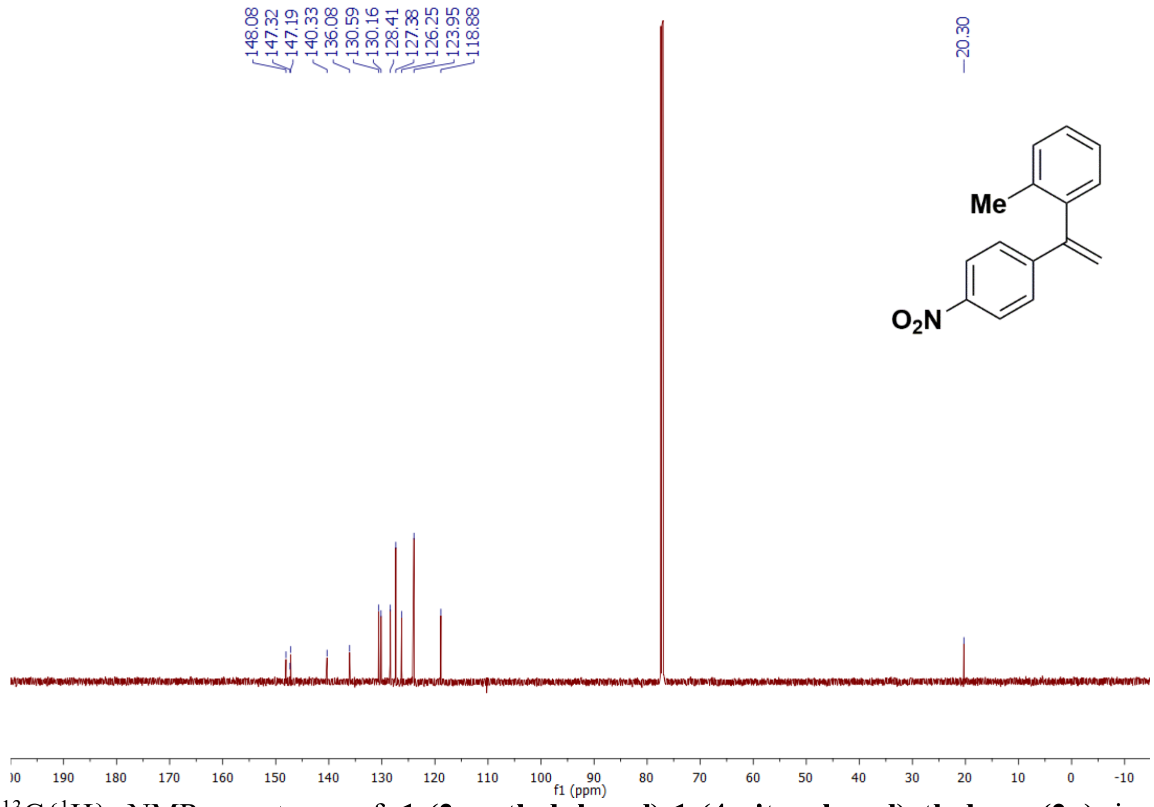

$^{13}C\{^{1}H\}$ NMR spectrum of **1-(2-methylphenyl)-1-(4-nitrophenyl)ethylene (2n)** in $CDCl_3$ at 150 MHz

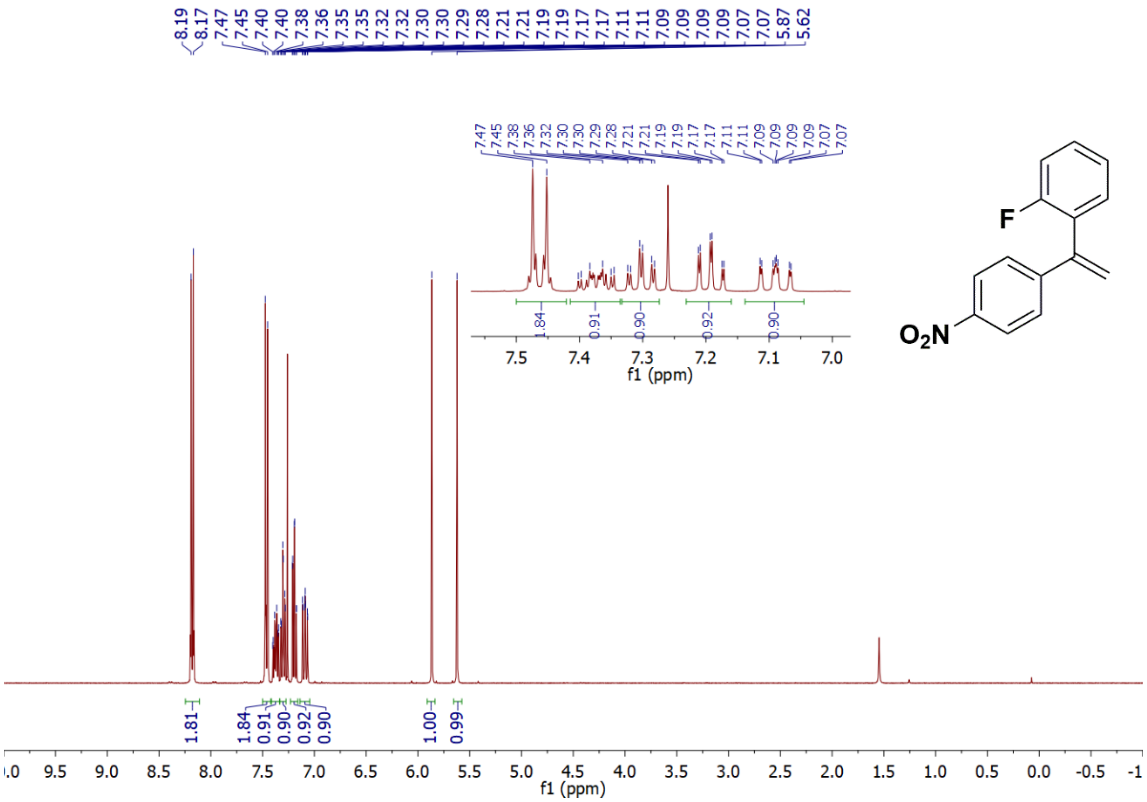

^{1}H NMR spectrum of **1-(2-fluorophenyl)-1-(4-nitrophenyl)ethylene (2o)** in $CDCl_3$ at 600 MHz

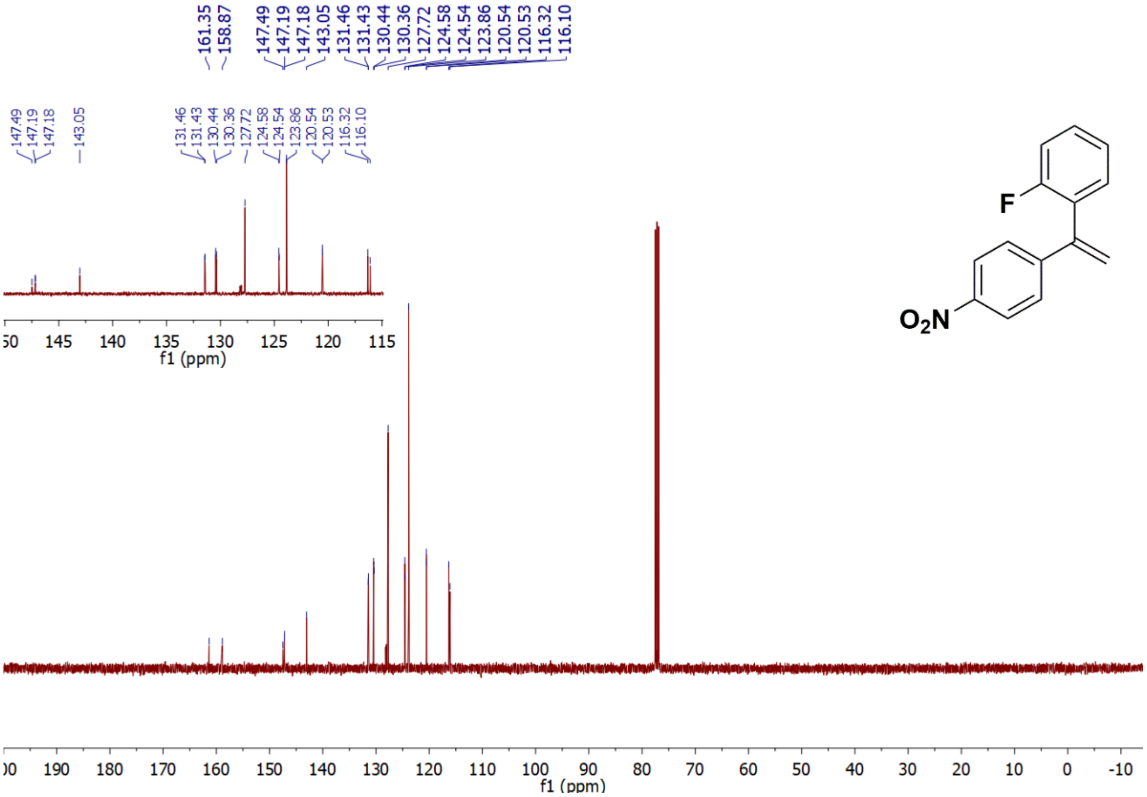

^{13}C{^{1}H} NMR spectrum of **1-(2-fluorophenyl)-1-(4-nitrophenyl)ethylene (2o)** in $CDCl_3$ at 150 MHz

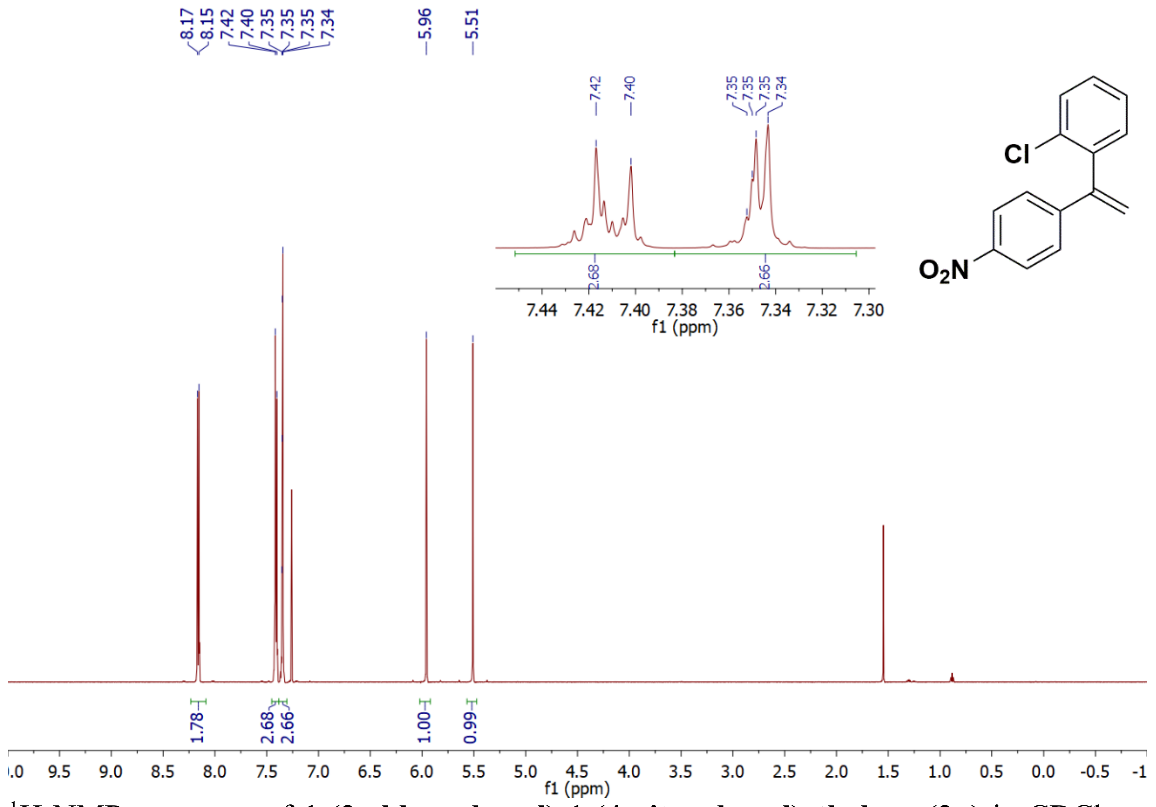

^{1}H NMR spectrum of **1-(2-chlorophenyl)-1-(4-nitrophenyl)ethylene (2p)** in $CDCl_3$ at 600 MHz

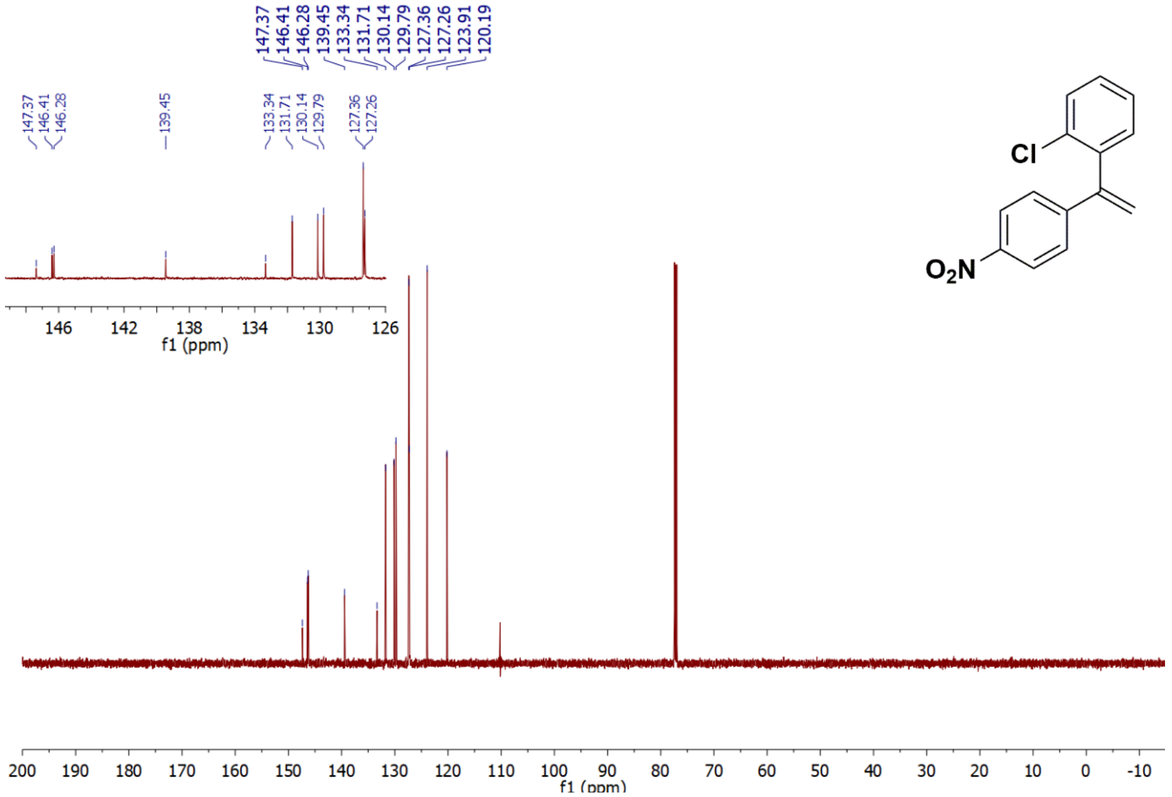

^{13}C{^{1}H} NMR spectrum of **1-(2-chlorophenyl)-1-(4-nitrophenyl)ethylene (2p)** in $CDCl_3$ at 150 MHz

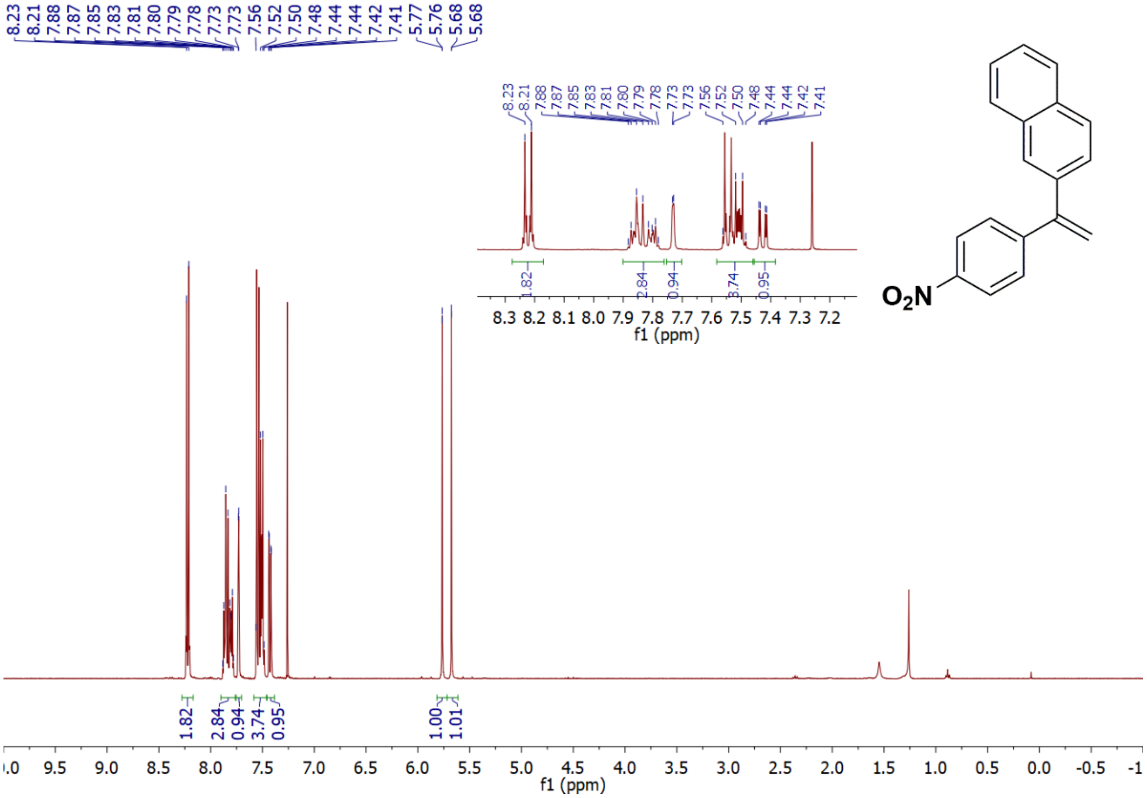

^{1}H NMR spectrum of **1-(naphthyl)-1-(4-nitrophenyl)ethylene (2q)** in $CDCl_3$ at 400 MHz

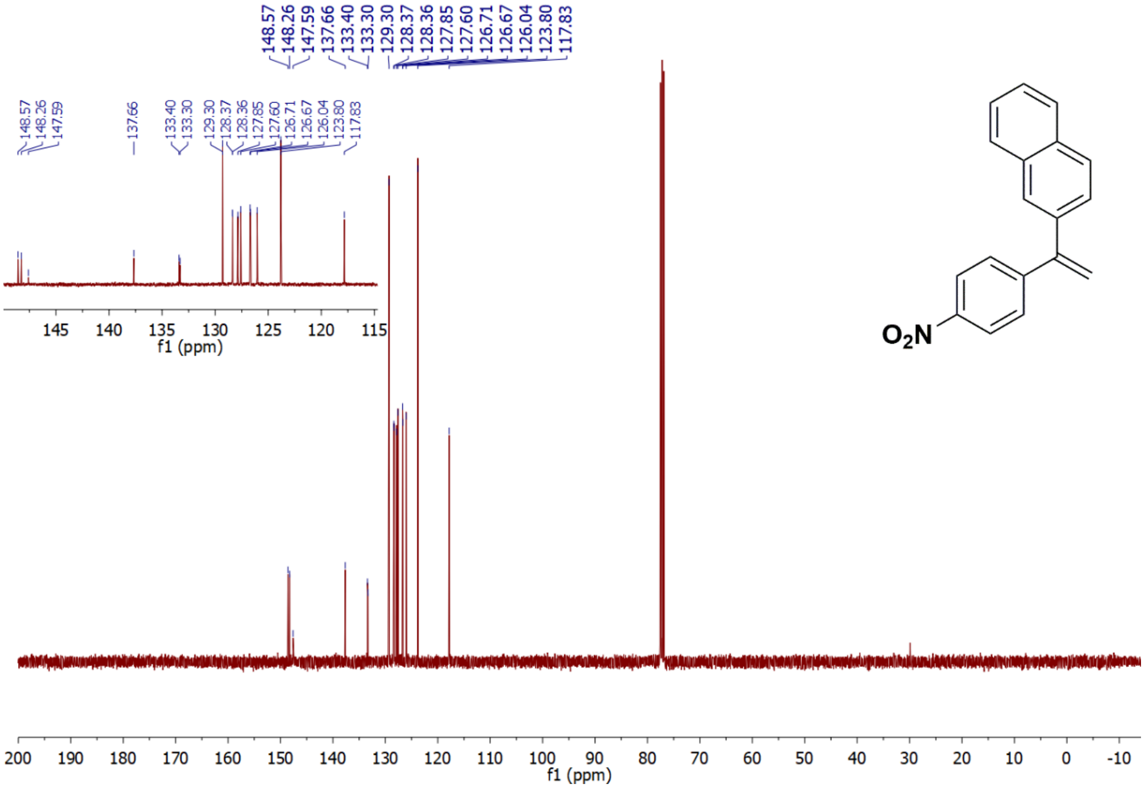

^{13}C{^{1}H} NMR spectrum of **1-(naphthyl)-1-(4-nitrophenyl)ethylene (2q)** in $CDCl_3$ at 100 MHz

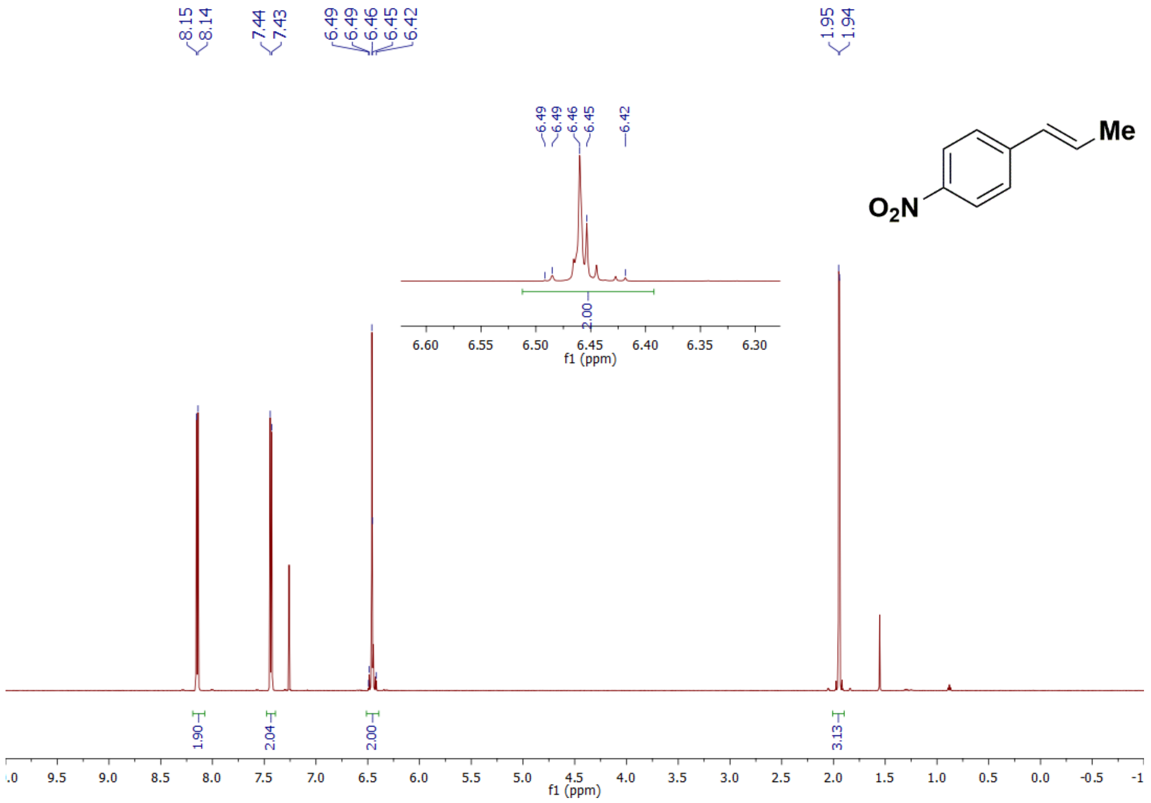

^{1}H NMR spectrum of **1-nitro-4-(1-propen-1-yl)benzene (2r)** in $CDCl_3$ at 400 MHz

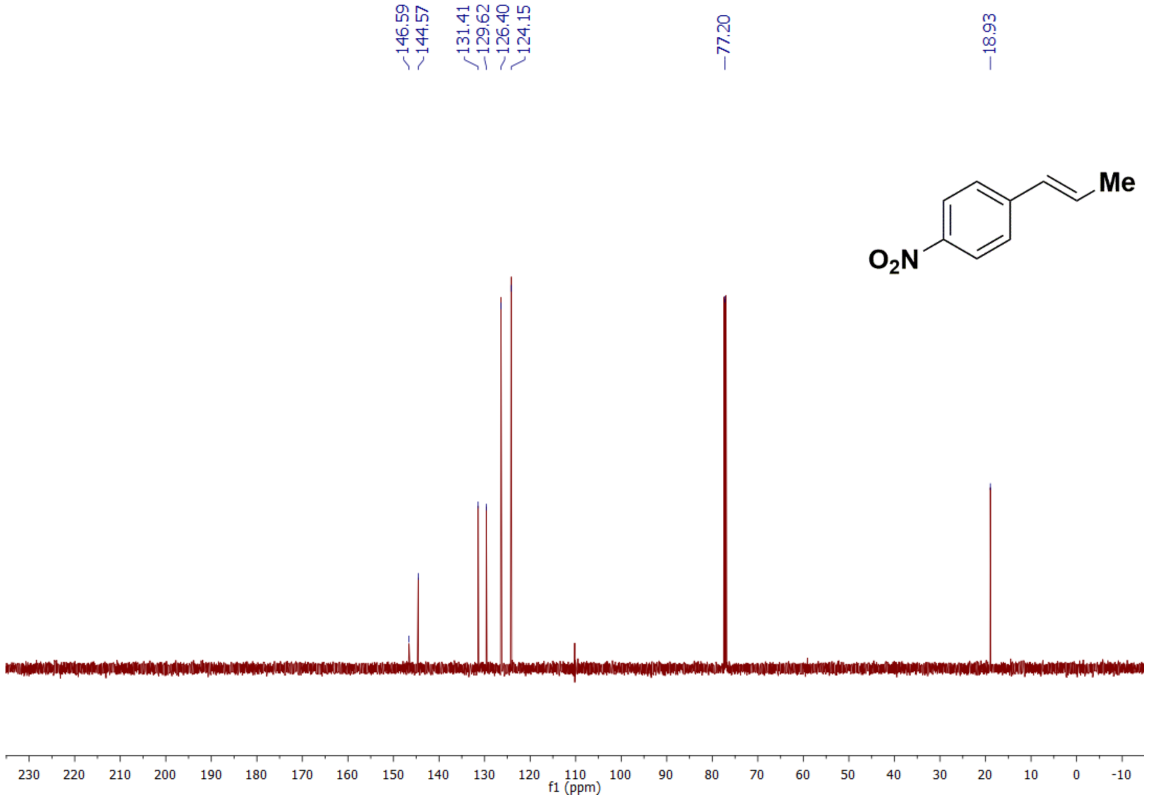

^{13}C{^{1}H} NMR spectrum of **1-nitro-4-(1-propen-1-yl)benzene (2r)** in $CDCl_3$ at 150 MHz

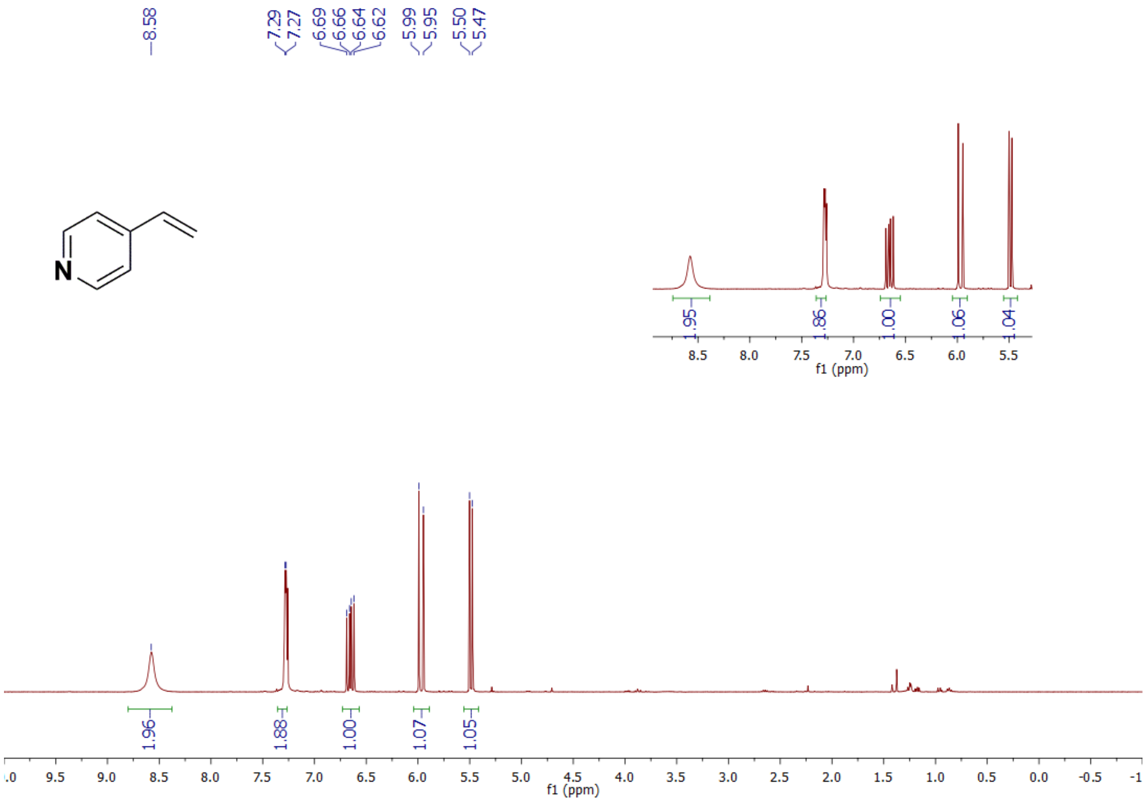

^{1}H NMR spectrum of **4-vinylpyridine (2s)** in $CDCl_3$ at 400 MHz

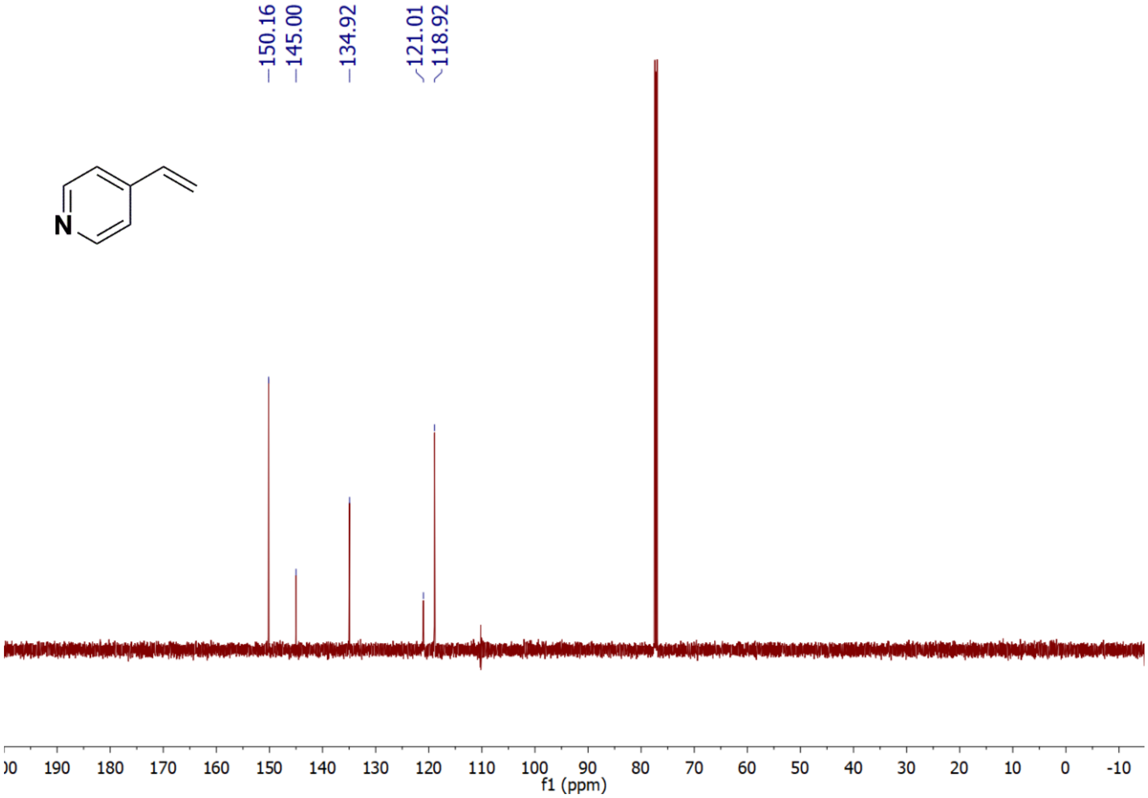

$^{13}C\{^{1}H\}$ NMR spectrum of **4-vinylpyridine (2s)** in $CDCl_3$ at 150 MHz

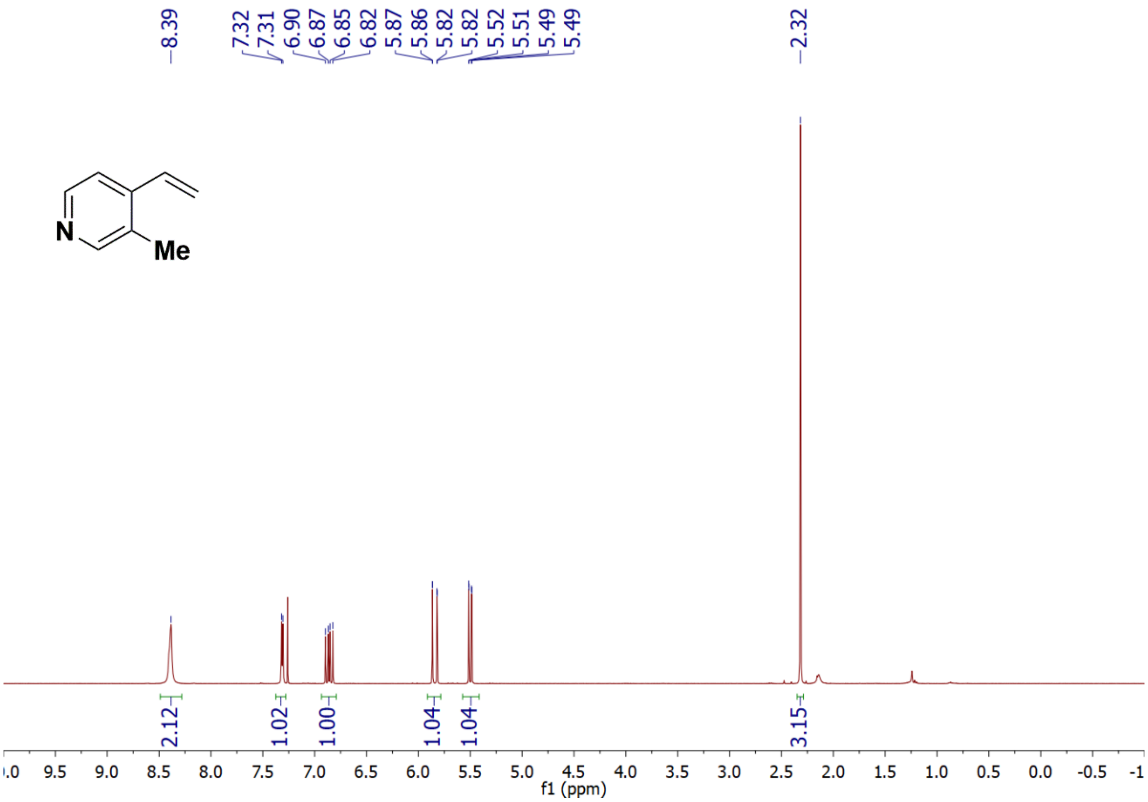

^{1}H NMR spectrum of **3-methyl-4-vinylpyridine (2t)** in $CDCl_3$ at 400 MHz

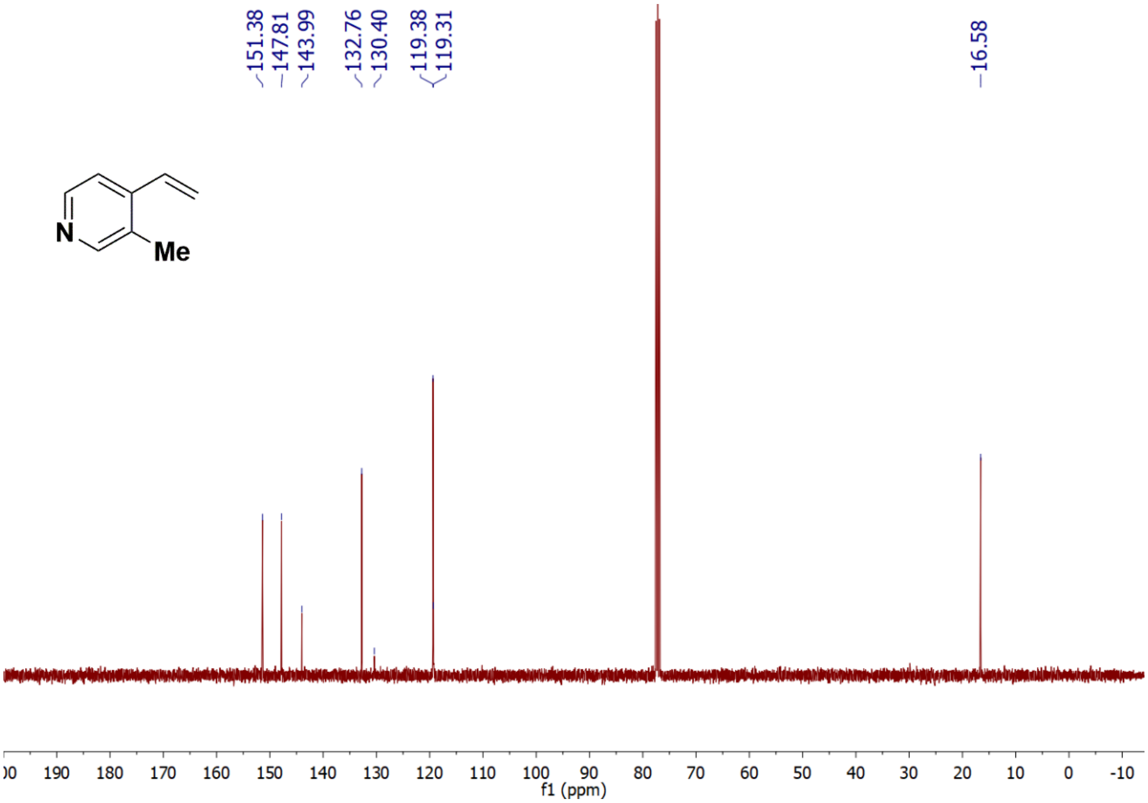

$^{13}C\{^{1}H\}$ NMR spectrum of **3-methyl-4-vinylpyridine (2t)** in $CDCl_3$ at 150 MHz

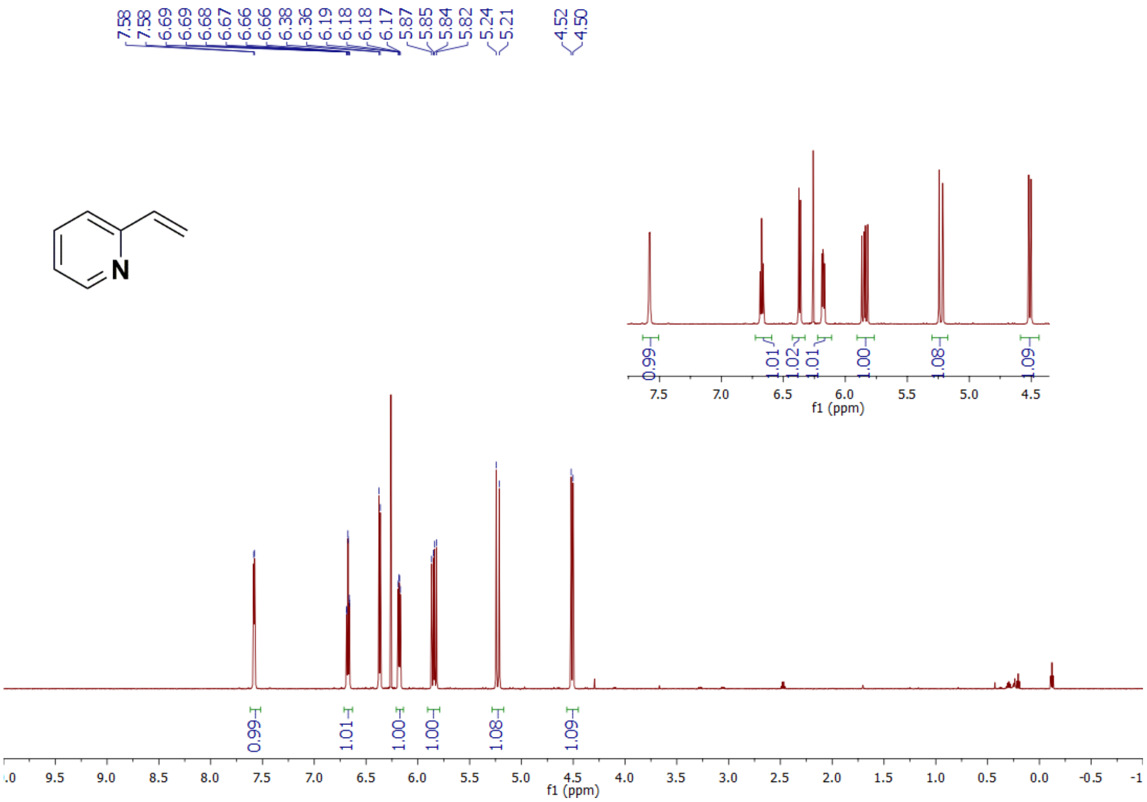

^{1}H NMR spectrum of **2-vinylpyridine (2u)** in $CDCl_3$ at 600 MHz

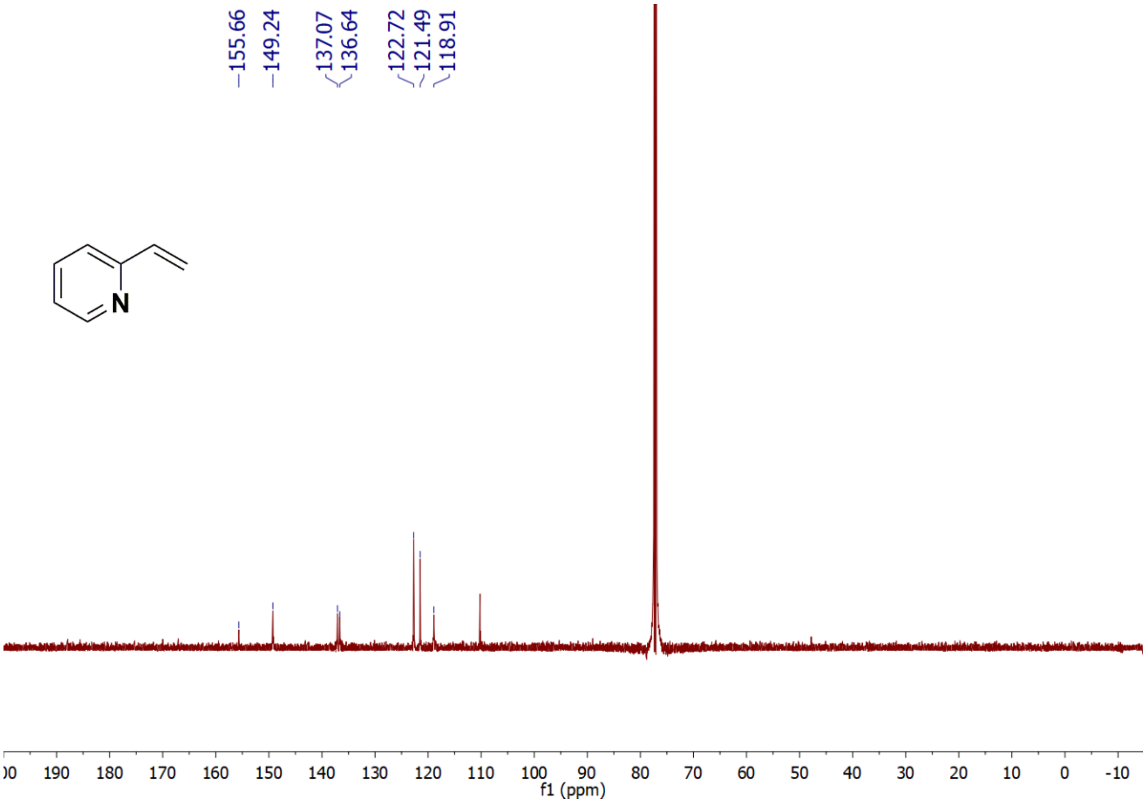

^{13}C{^{1}H} NMR spectrum of **2-vinylpyridine (2u)** in $CDCl_3$ at 150 MHz

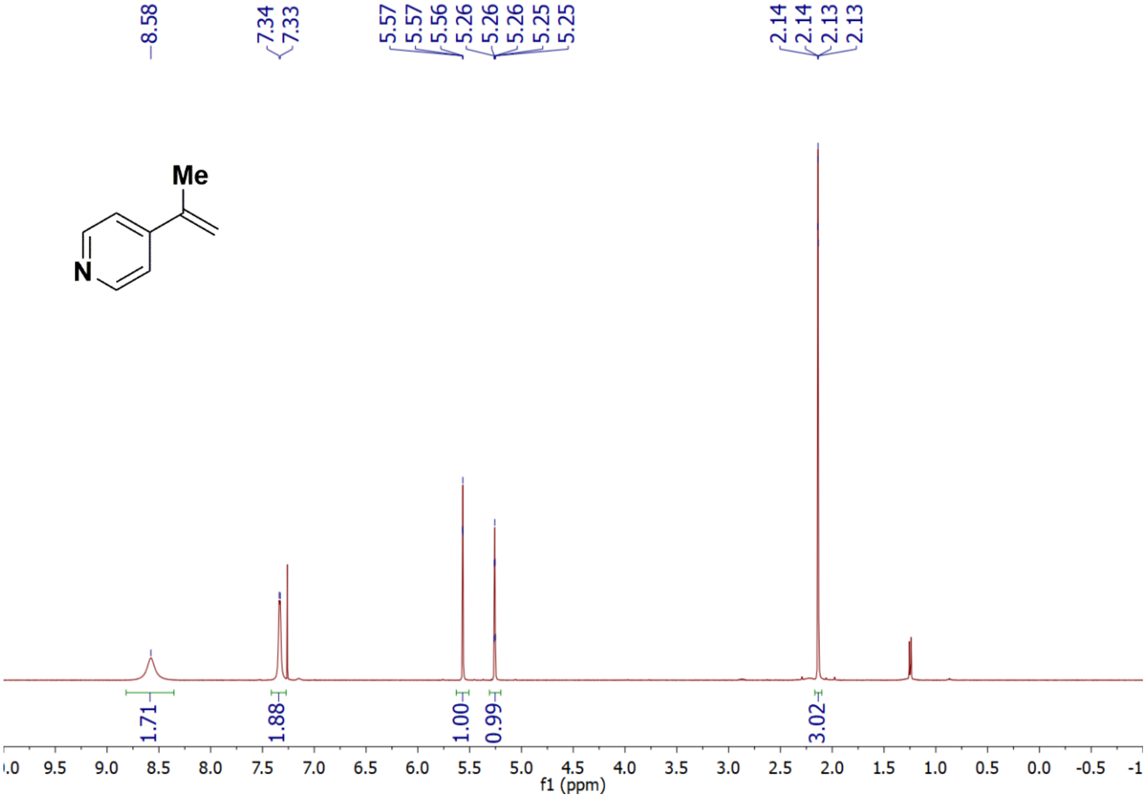

^{1}H NMR spectrum of **4-(1-methylvinyl)pyridine (2v)** in $CDCl_3$ at 400 MHz

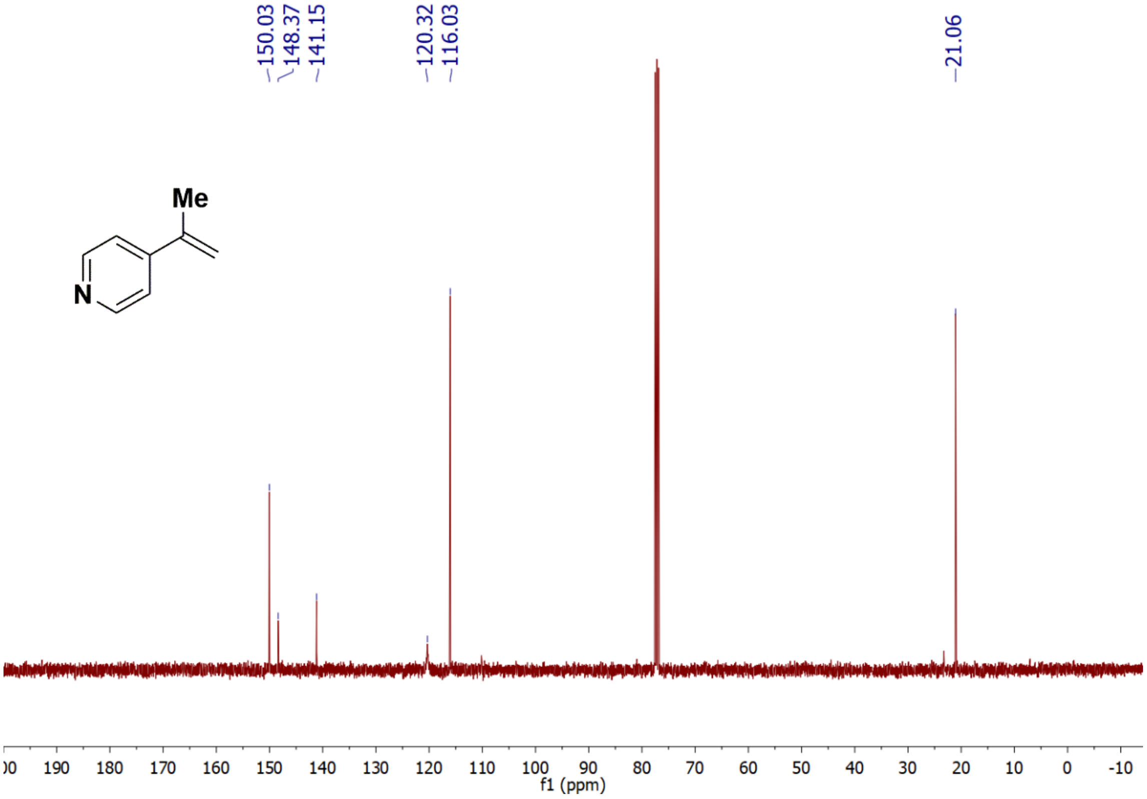

^{13}C{^{1}H} NMR spectrum of **4-(1-methylvinyl)pyridine (2v)** in $CDCl_3$ at 150 MHz

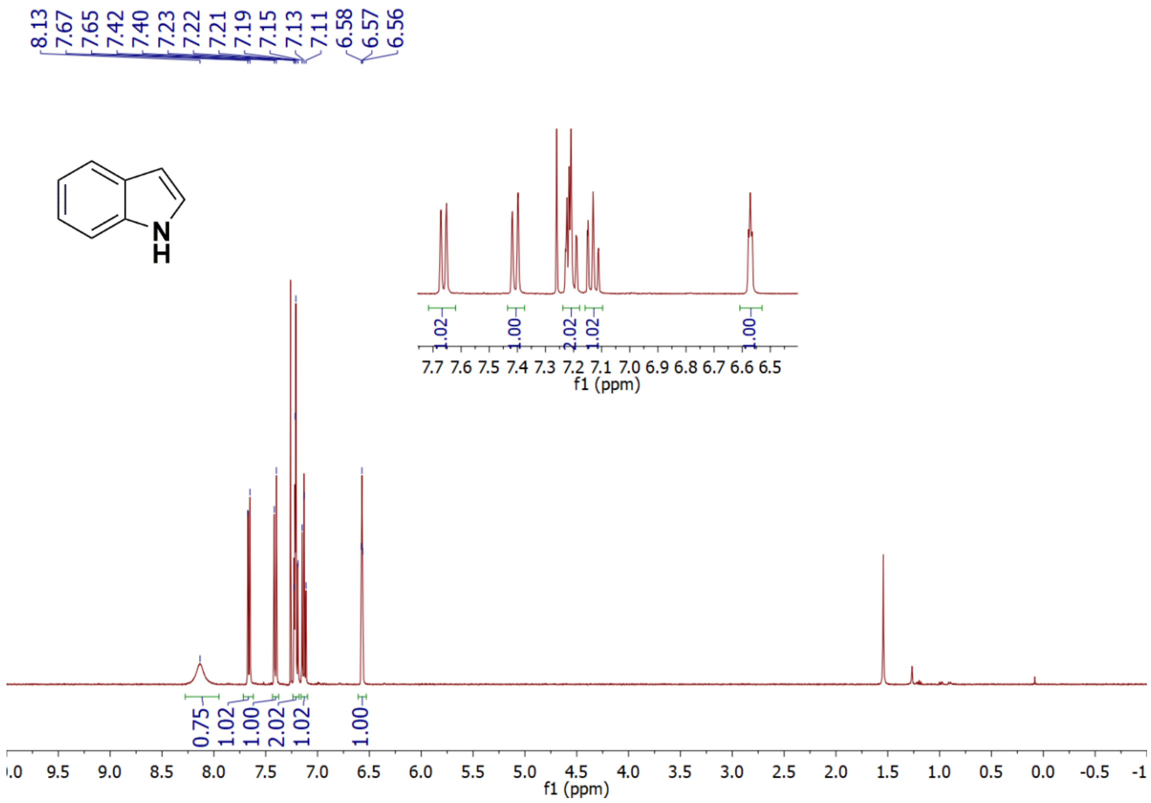

^{1}H NMR spectrum of **indole (2w)** in $CDCl_3$ at 400 MHz

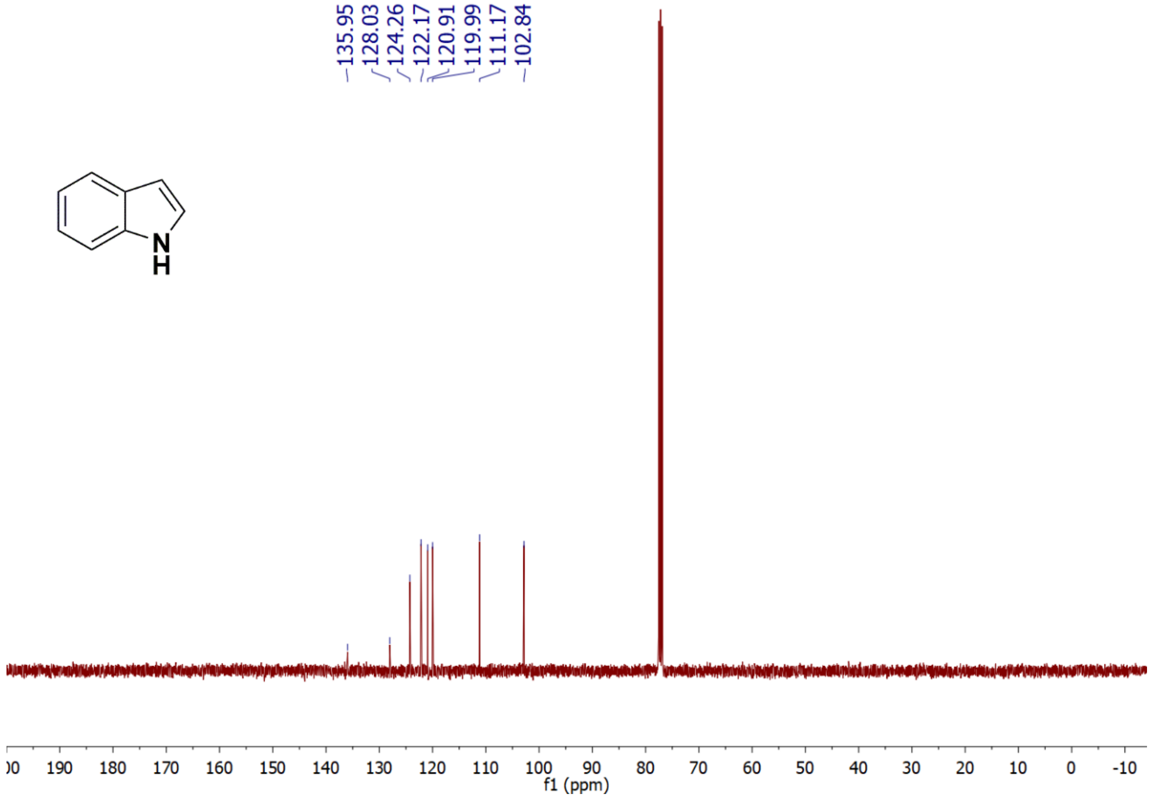

^{13}C{^{1}H} NMR spectrum of **indole (2w)** in $CDCl_3$ at 150 MHz

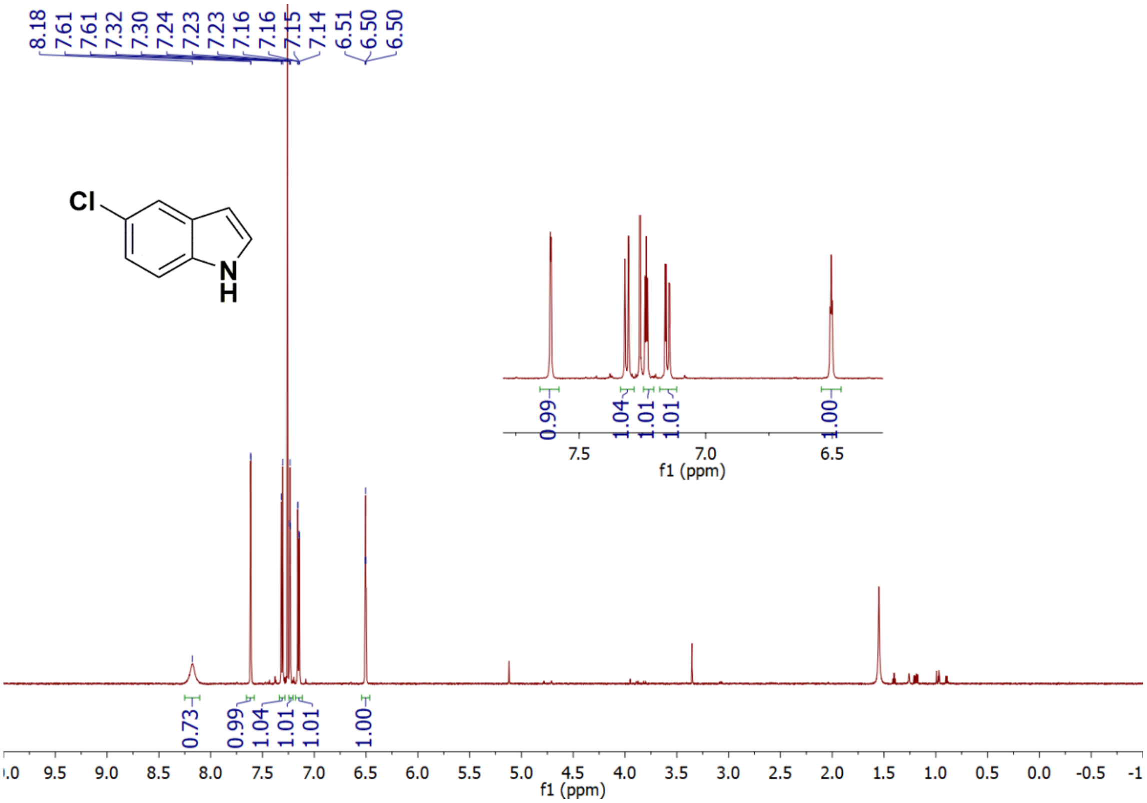

^{1}H NMR spectrum of **5-chloroindole (2x)** in $CDCl_3$ at 600 MHz

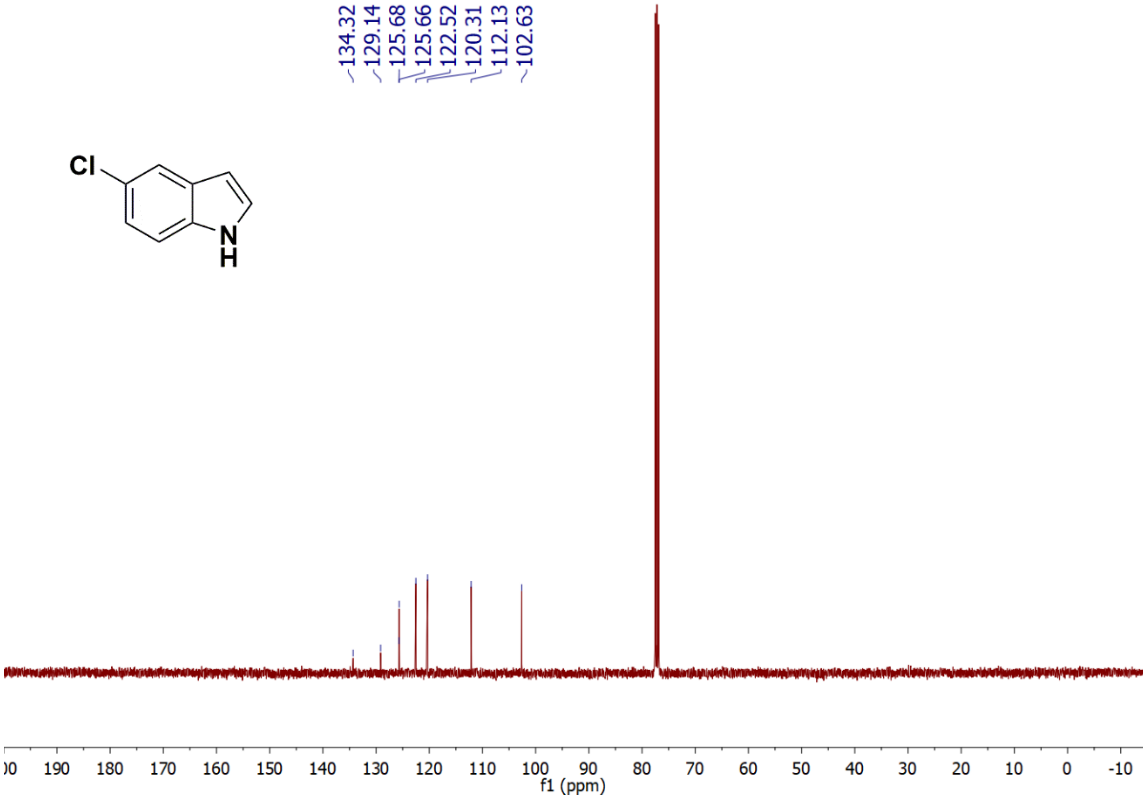

^{13}C{^{1}H} NMR spectrum of **5-chloroindole (2x)** in $CDCl_3$ at 150 MHz

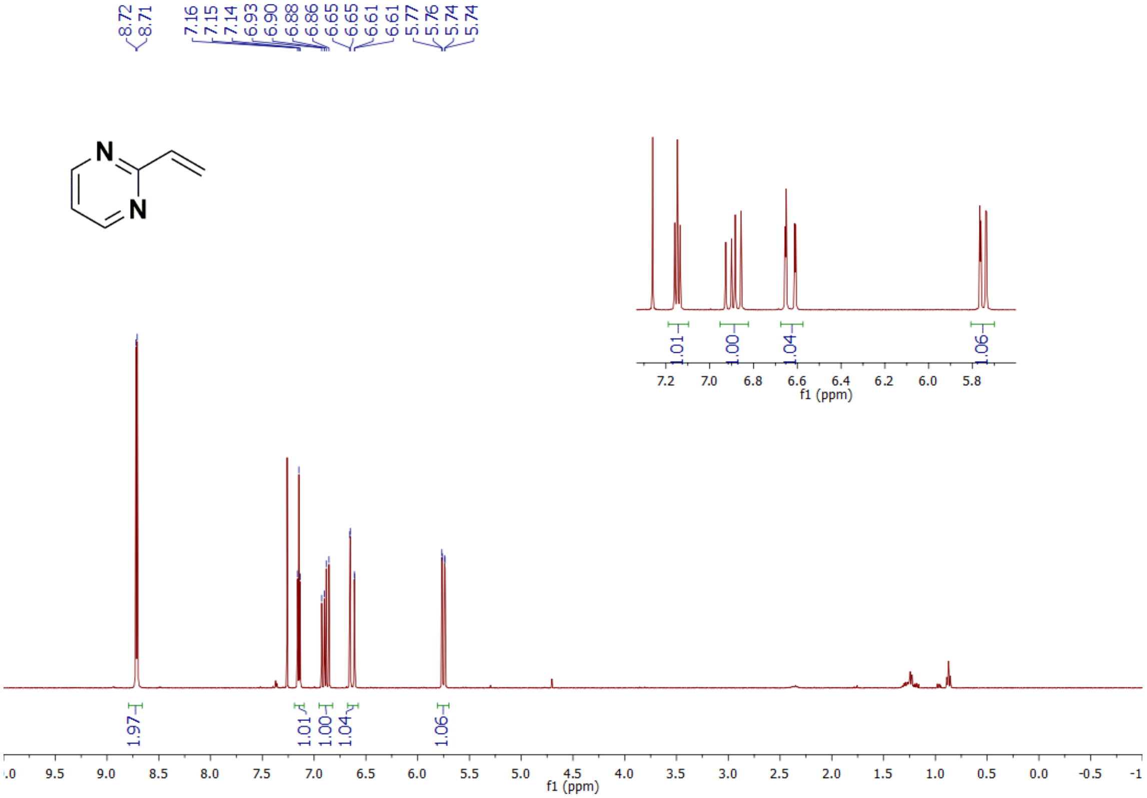

^{1}H NMR spectrum of **2-vinylpyrimidine (2y)** in $CDCl_3$ at 400 MHz

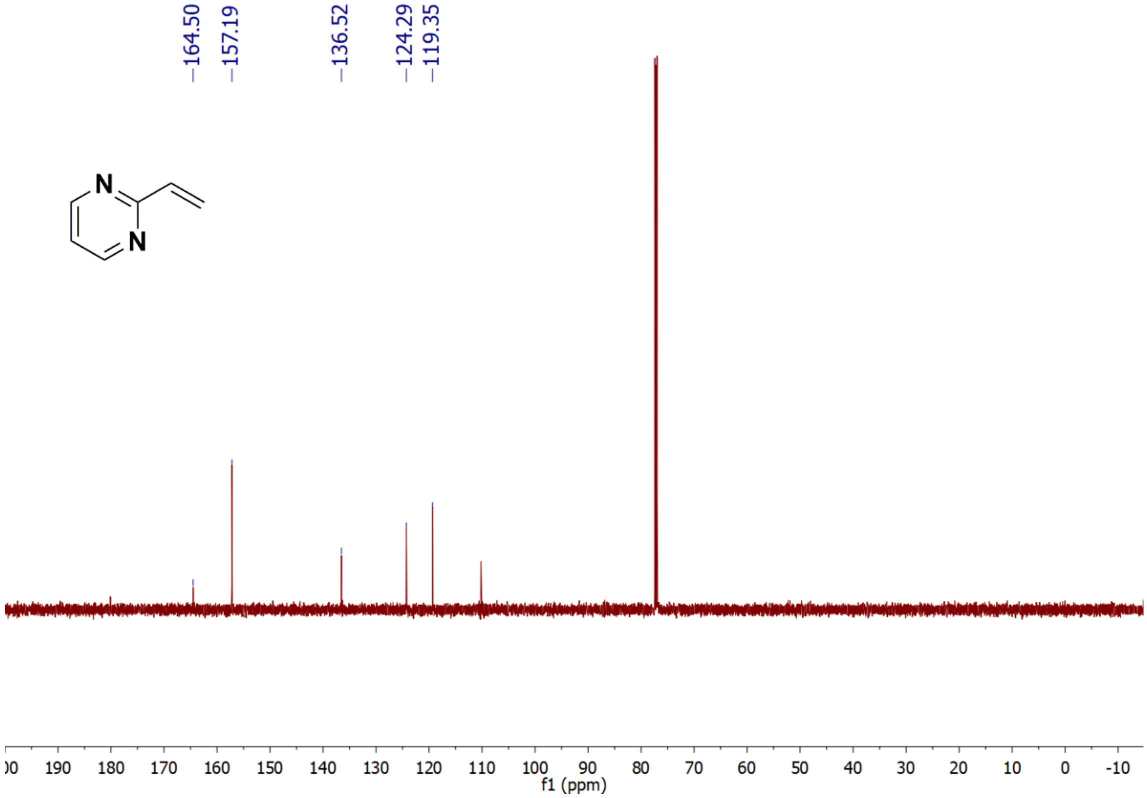

$^{13}C\{^{1}H\}$ NMR spectrum of **2-vinylpyrimidine (2y)** in $CDCl_3$ at 150 MHz

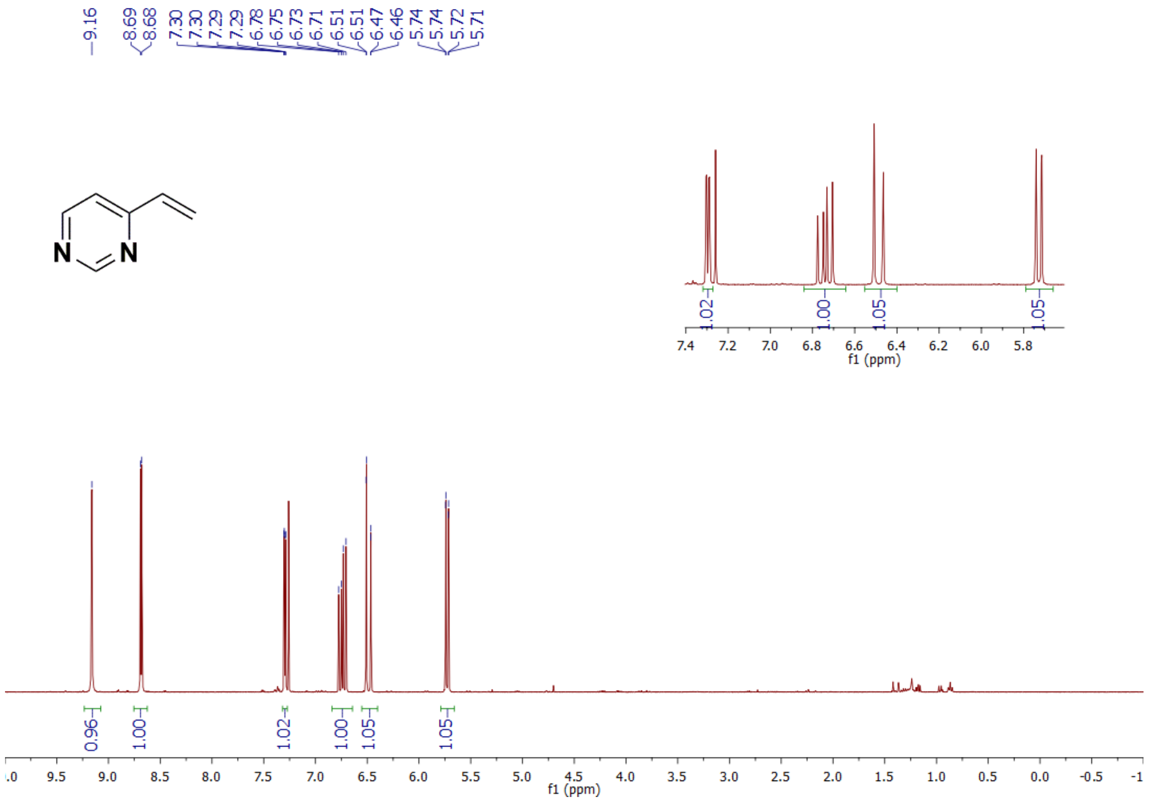

^{1}H NMR spectrum of **4-vinylpyrimidine (2z)** in $CDCl_3$ at 400 MHz

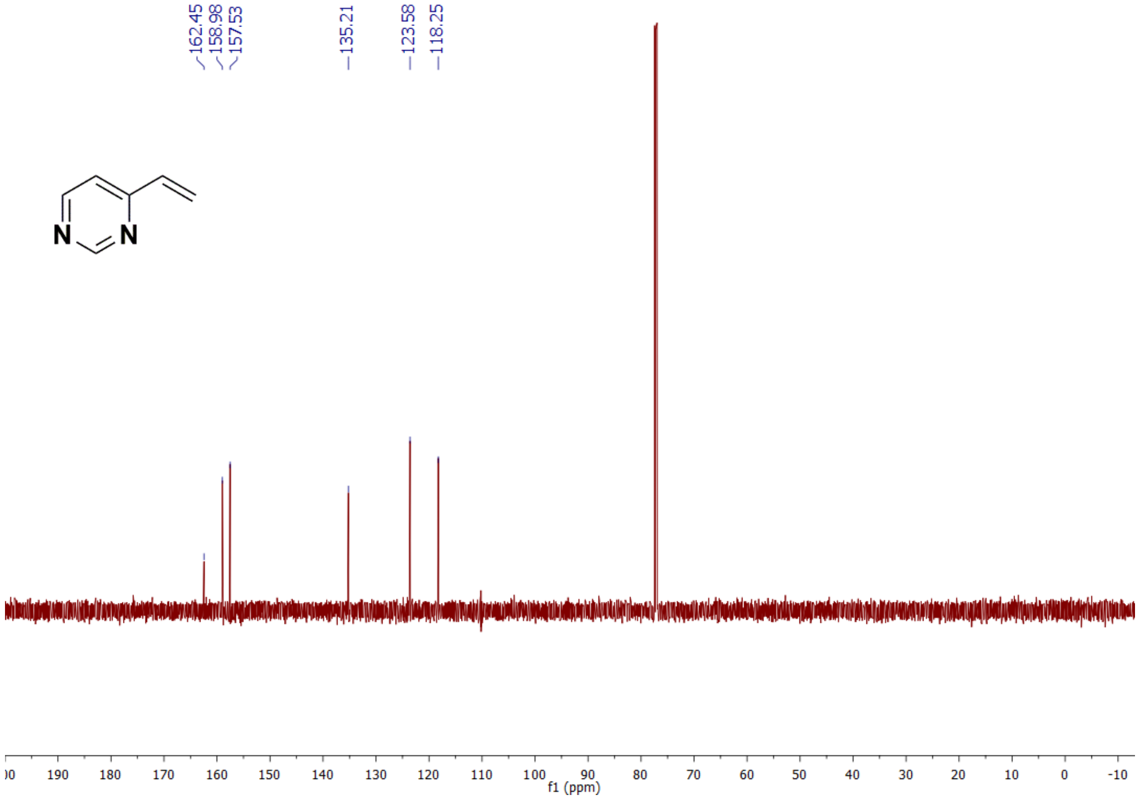

^{13}C{^{1}H} NMR spectrum of **4-vinylpyrimidine (2z)** in $CDCl_3$ at 150 MHz

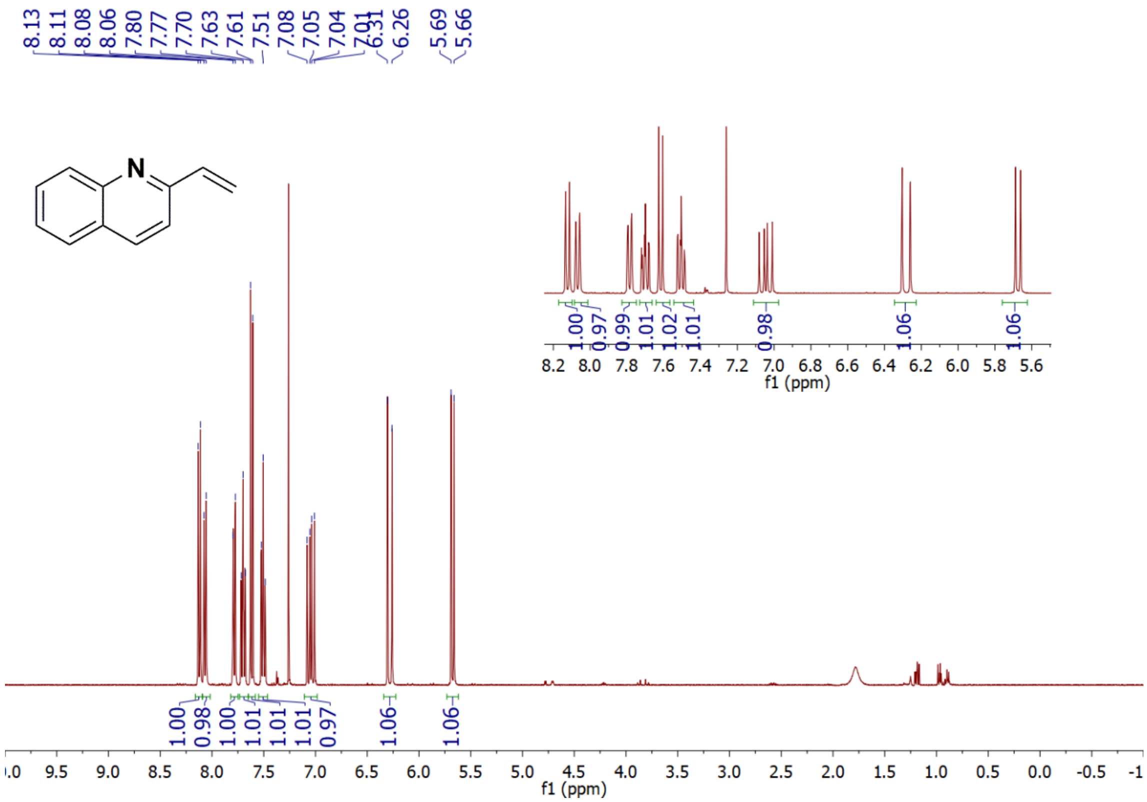

^{1}H NMR spectrum of **2-vinylquinoline (2aa)** in $CDCl_3$ at 400 MHz

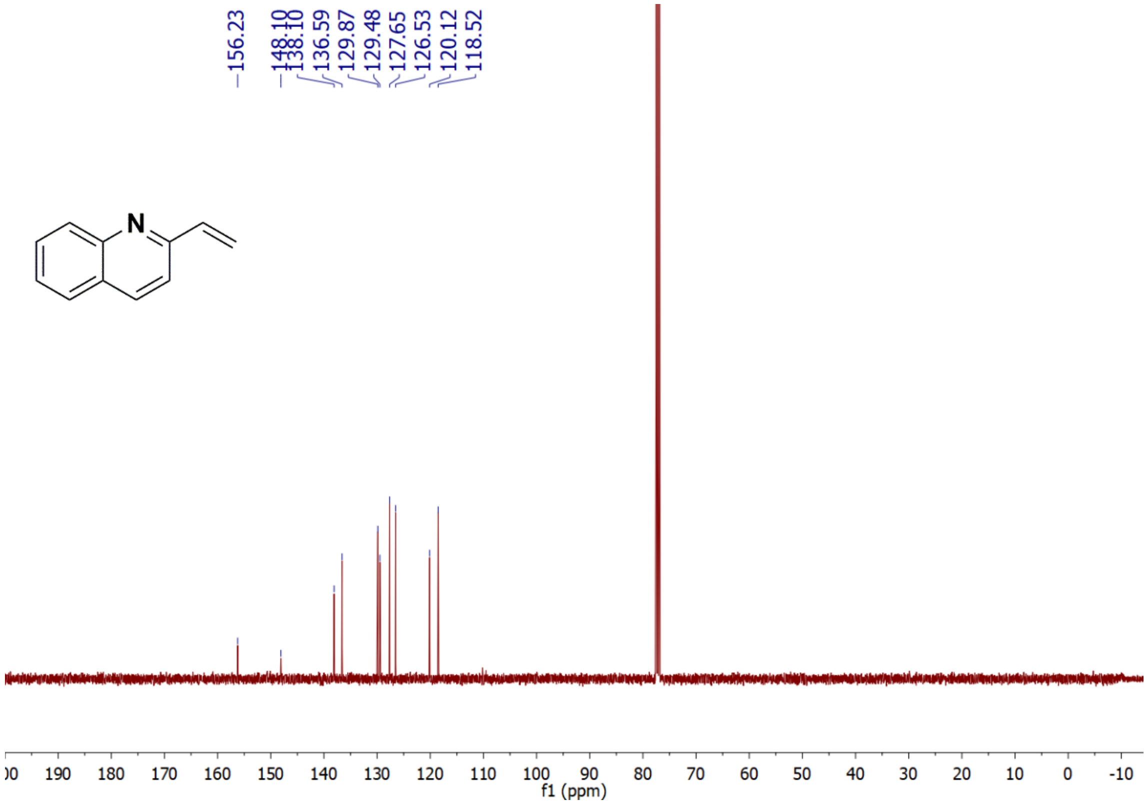

$^{13}C\{^{1}H\}$ NMR spectrum 2-vinylquinoline **(2aa)** in $CDCl_3$ at 150 MHz

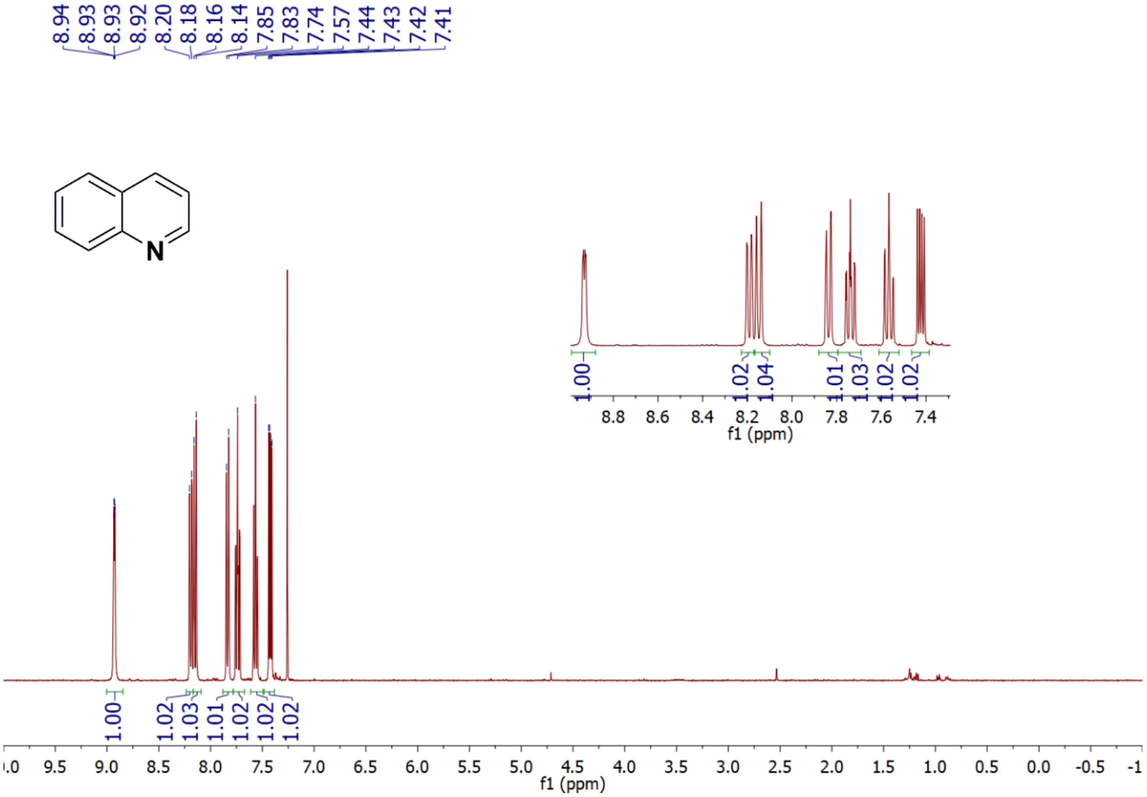

^{1}H NMR spectrum of **quinoline (2ab)** in $CDCl_3$ at 400 MHz

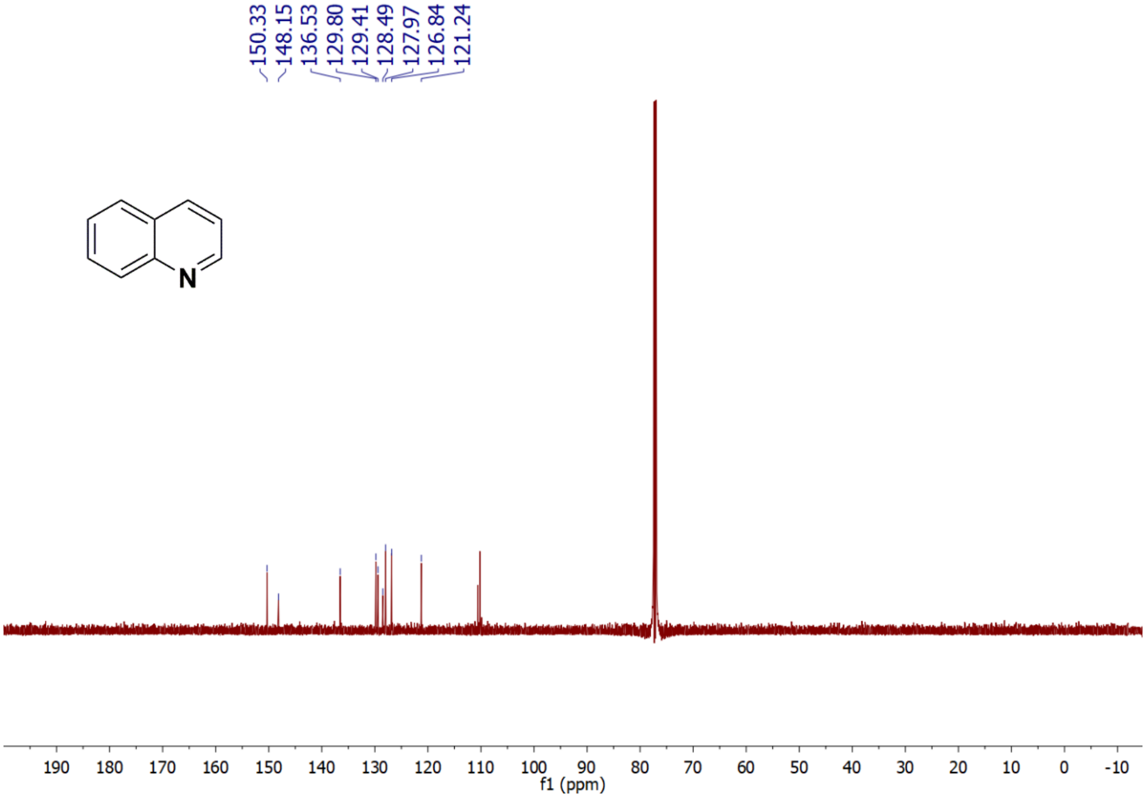

$^{13}C\{^{1}H\}$ NMR spectrum **quinoline (2ab)** in $CDCl_3$ at 150 MHz

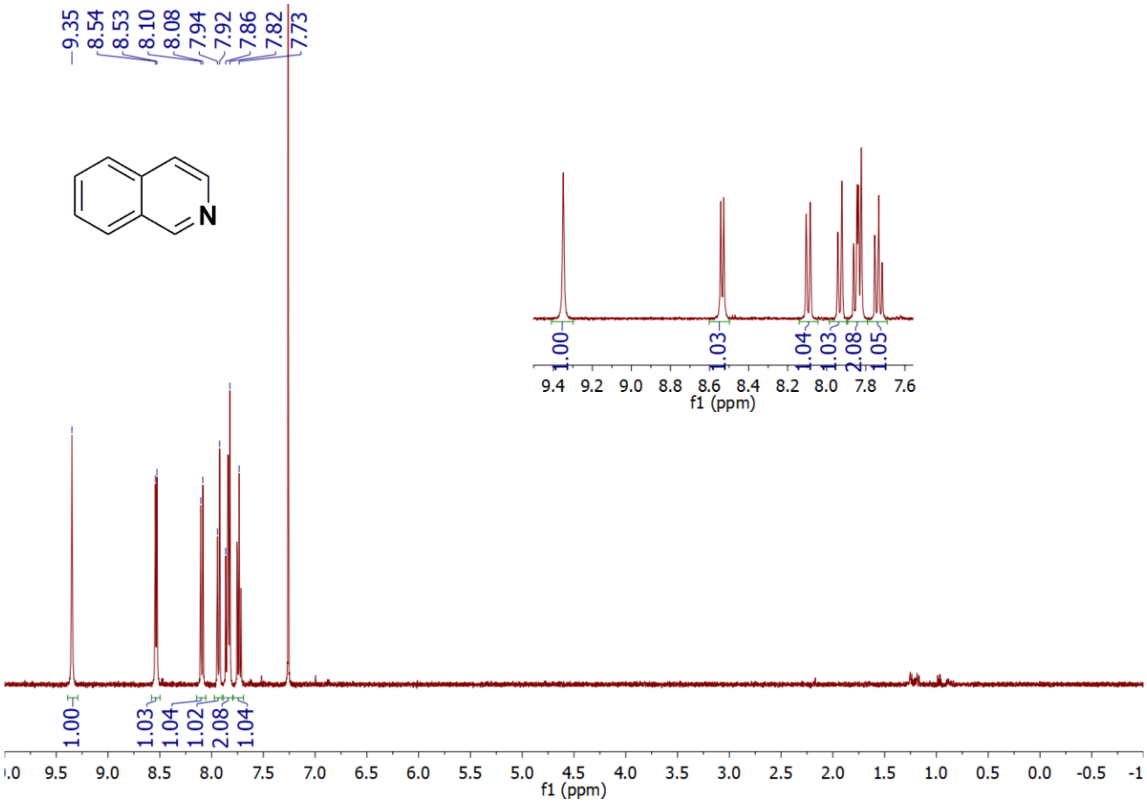

^{1}H NMR spectrum of **isoquinoline (2ac)** in $CDCl_3$ at 400 MHz

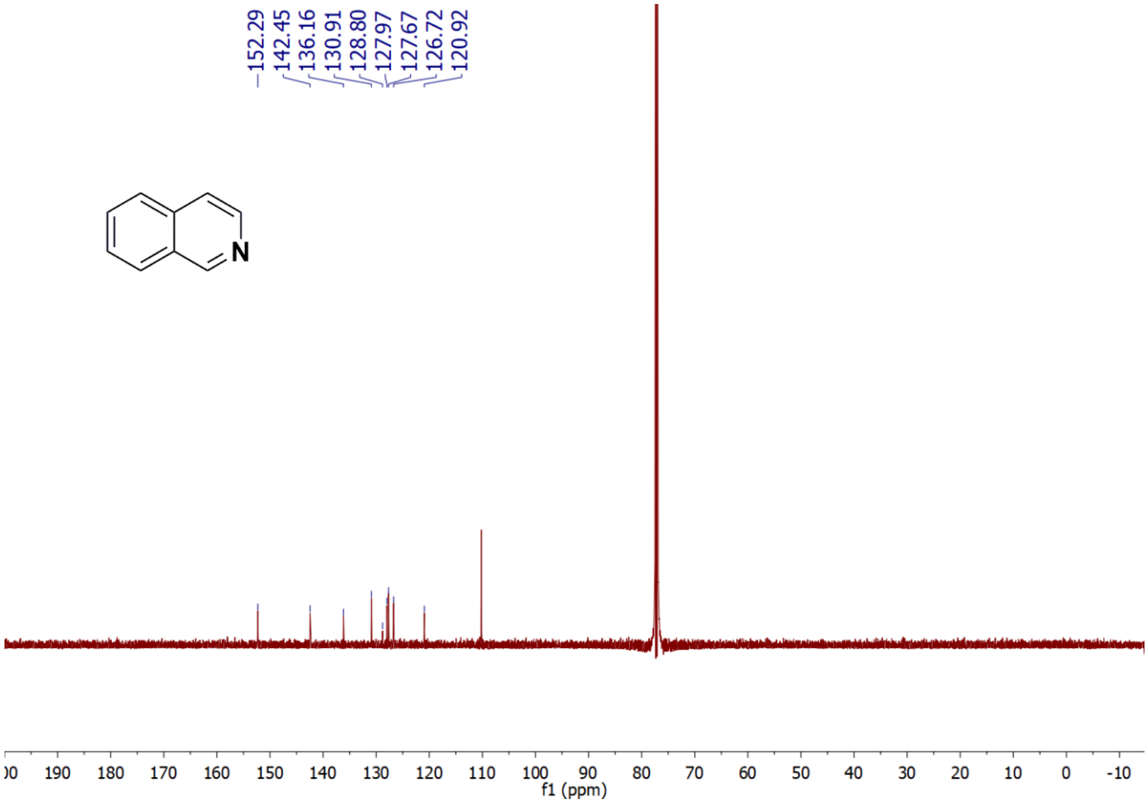

^{13}C{^{1}H} NMR spectrum **isoquinoline (2ac)** in $CDCl_3$ at 150 MHz

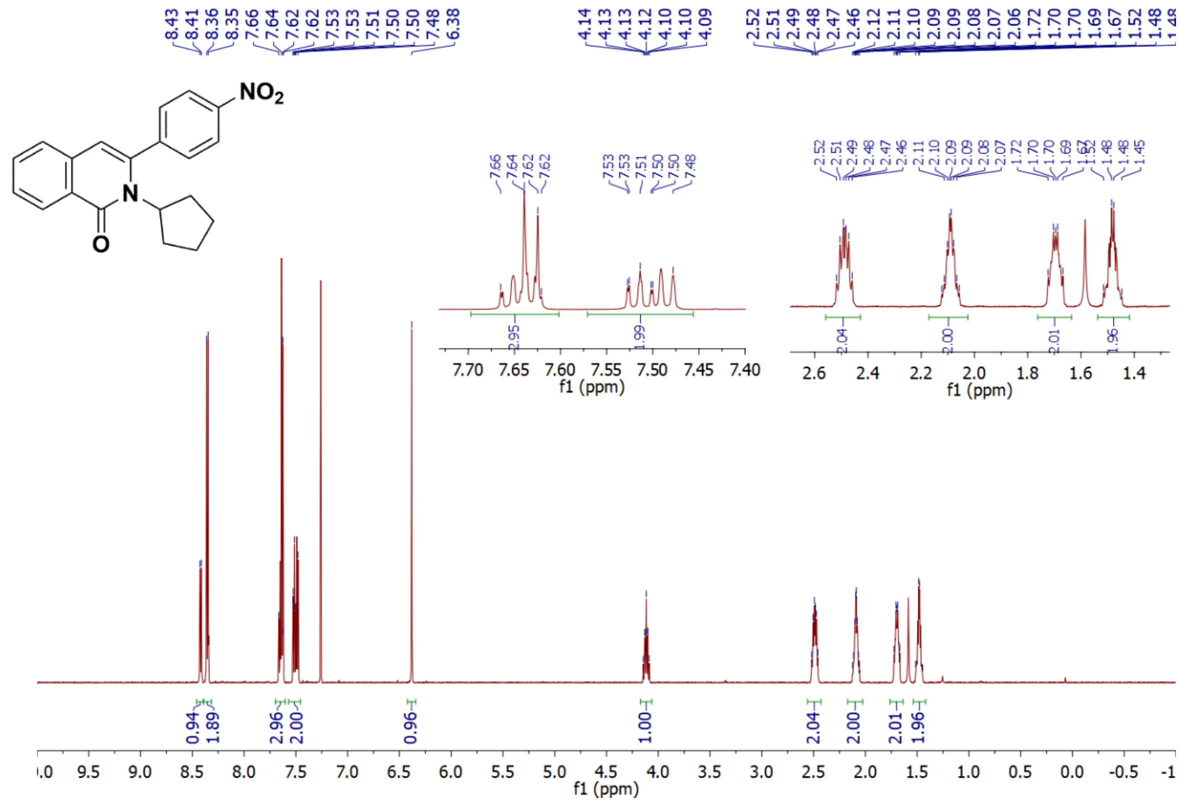

^{1}H NMR spectrum of **2-cyclopentyl-3-(4-nitrophenyl)-1(2*H*)-isoquinolinone (2ad)** in $CDCl_3$ at 600 MHz

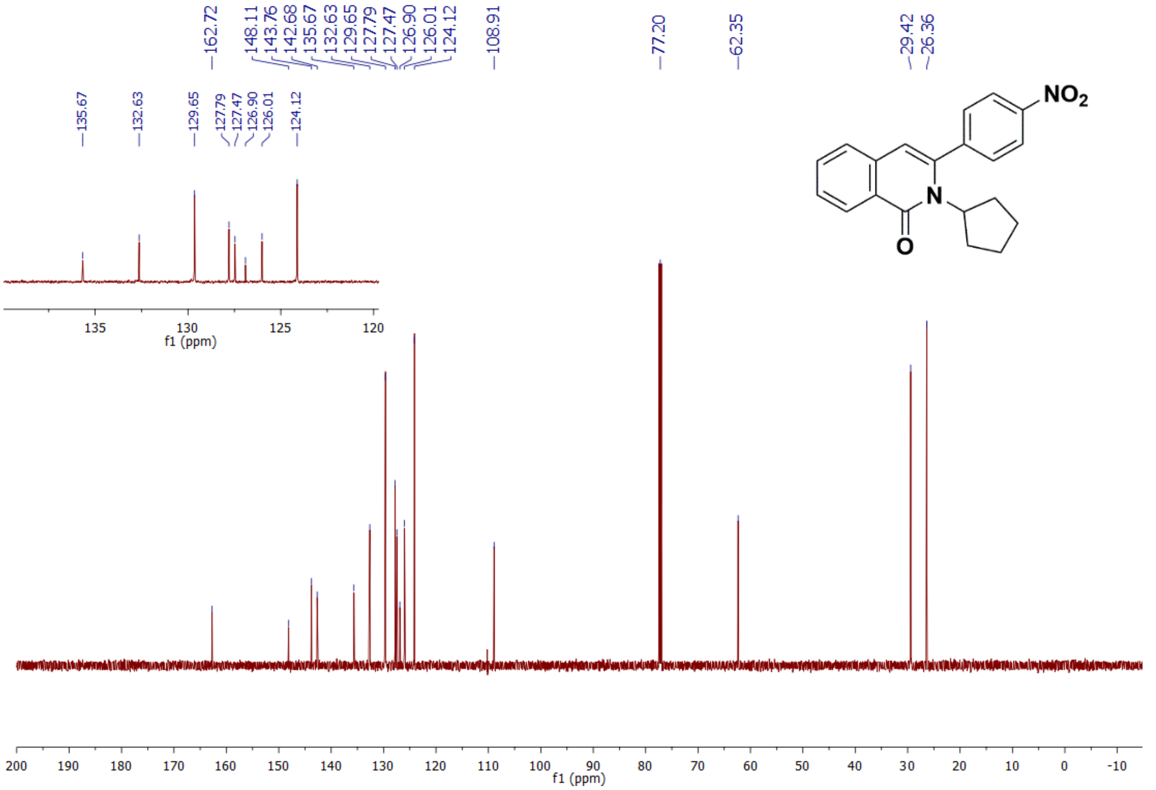

^{13}C{^{1}H} NMR spectrum of **2-cyclopentyl-3-(4-nitrophenyl)-1(2*H*)-isoquinolinone (2ad)** in $CDCl_3$ at 150 MHz

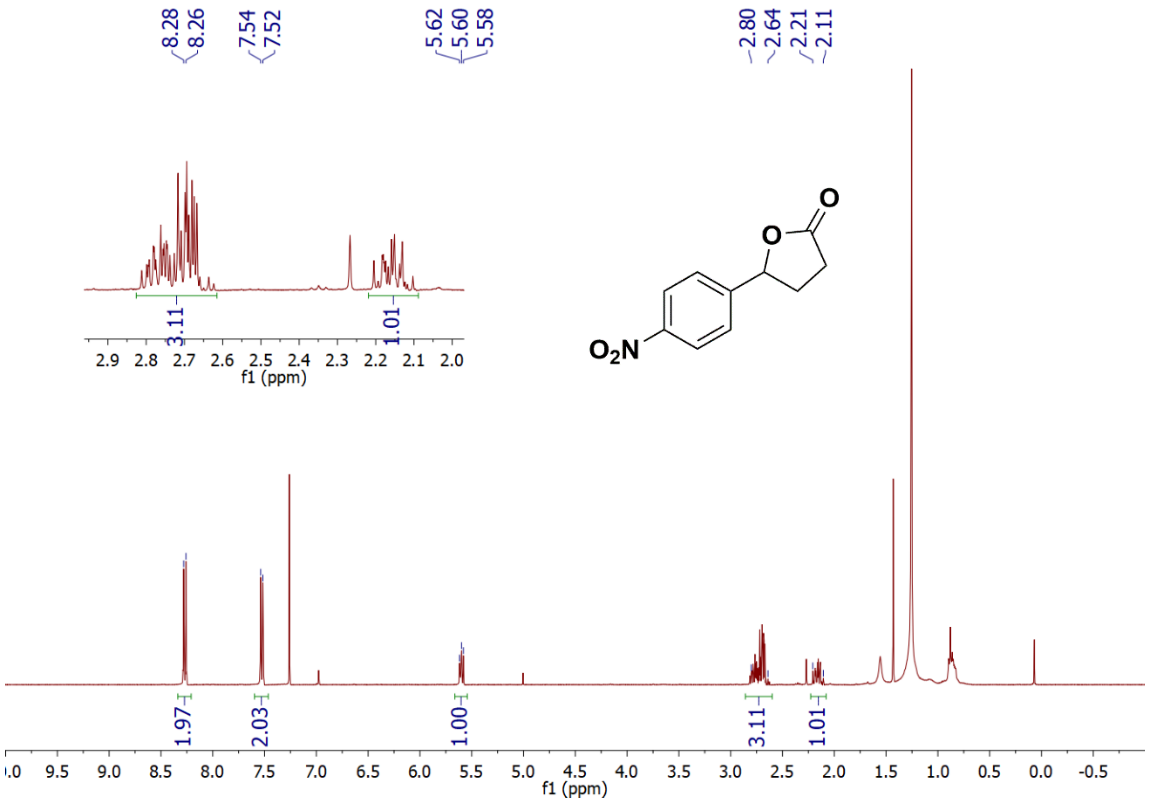

^{1}H NMR spectrum of **4-(4-nitrophenyl)-γ-butyrolactone (4)** in $CDCl_3$ at 400 MHz

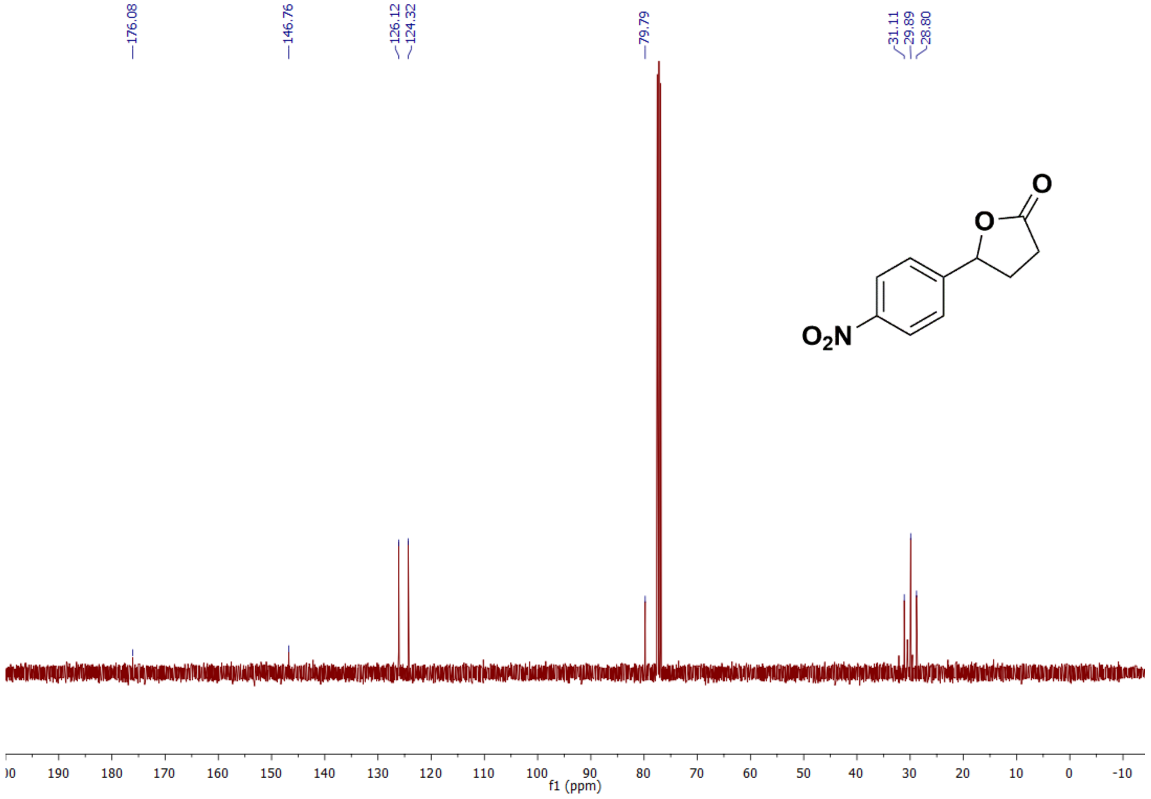

^{13}C{^{1}H} NMR spectrum **4-(4-nitrophenyl)-γ-butyrolactone (4)** in $CDCl_3$ at 100 MHz

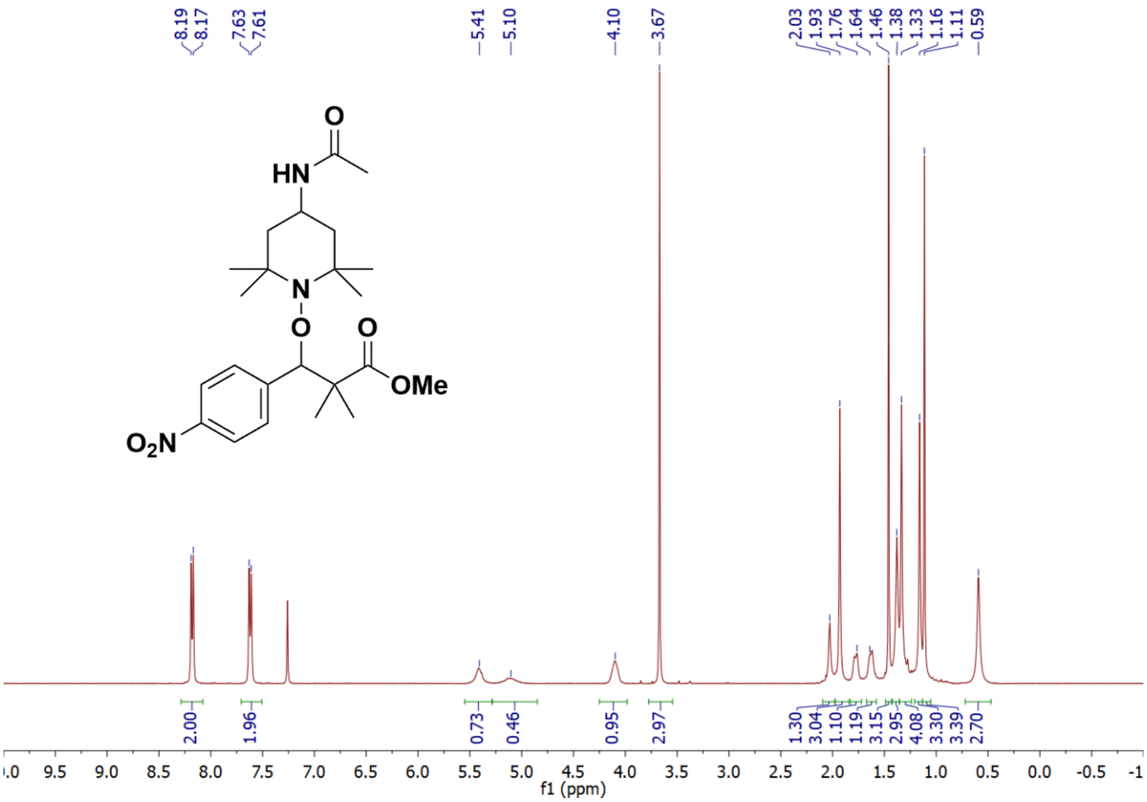

^{1}H NMR spectrum of **2,2-dimethyl-[3-[4-(acetamido)-2,2,6,6-tetramethyl-1-piperidinyl]oxy]-3-(4-nitrophenyl)methyl propionate (6)** in $CDCl_3$ at 600 MHz

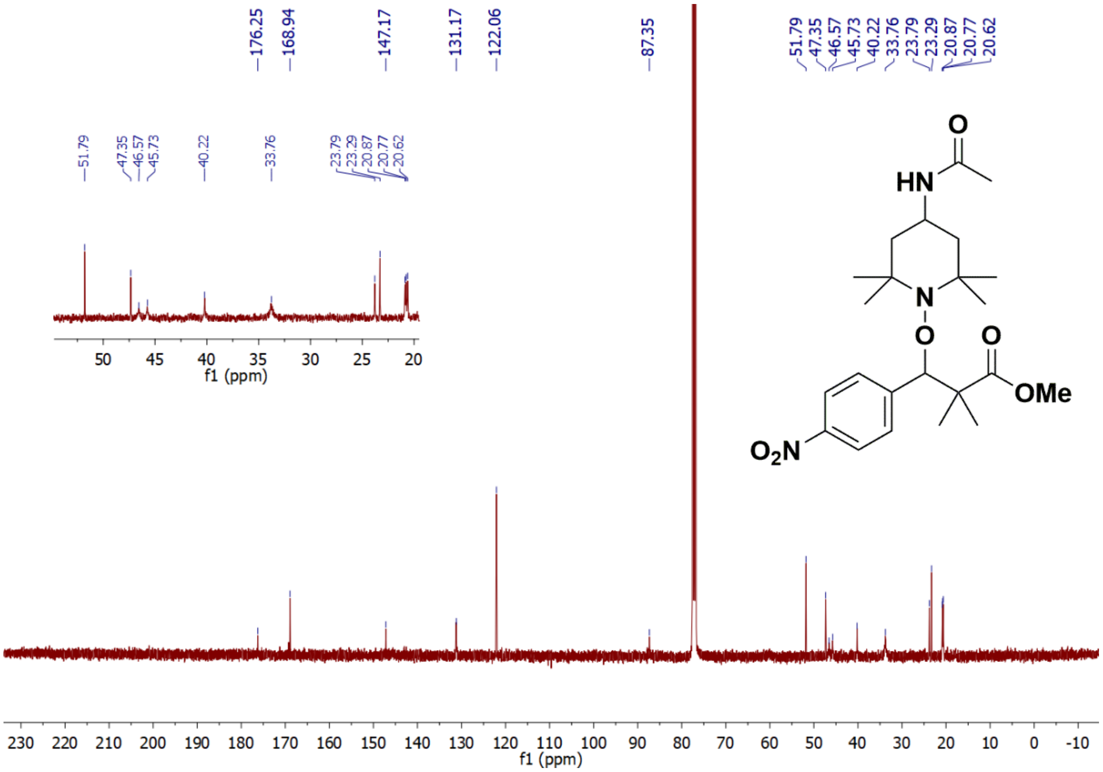

^{13}C{^{1}H} NMR spectrum **2,2-dimethyl-[3-[4-(acetamido)-2,2,6,6-tetramethyl-1-piperidinyl]oxy]-3-(4-nitrophenyl)methyl propionate (6)** in $CDCl_3$ at 150 MHz
